# Supplementary material for: A directly comparative two-gate case–control diagnostic accuracy study of the pure tone screen and HearCheck screener tests for identifying hearing impairment in school children
Source: BMJ Open. 2017 Jul 11;7(7):e017258. doi: 10.1136/bmjopen-2017-017258 (PMC5541595; doi:10.1136/bmjopen-2017-017258)
Supplement: Supplementary Appendix 1 [file bmjopen-2017-017258supp001.pdf]

Appendix

Variability in diagnostic accuracy across different scenarios

The primary analyses reported in the main text are for accuracy at ear level of the pure tone screen (PTS) and HearCheck Screener (HC) tests for distinguishing hearing impaired ears from non-impaired ears. Hearing impairment was defined as present when the PTA reference standard threshold was ≥30dB on at least one of the four frequencies (0.5 kHz, 1 kHz, 2 kHz and 4 kHz) and absent when the reference threshold was <30dB on all four frequencies. All impaired ears were used to calculate test sensitivity regardless of whether belonging to children recruited via audiology services (intended cases) or via schools (intending controls). In addition, we performed further exploratory analyses estimating accuracy of the tests in alternative scenarios:

a) when impairment was defined as present when the mean PTA threshold across the four frequencies was ≥30dB and absent when the mean threshold was <30dB,
b) when only impaired ears (children) that were recruited via audiology services were used to estimate sensitivity and when only impaired ears (children) that were recruited via schools were used to estimate sensitivity,
c) at child level for distinguishing between hearing impaired children and non-impaired children.

The primary definition of hearing impairment is stricter (i.e., the PTA reference standard is harder to pass) than the one based on mean hearing level across the 4 frequencies, since under the former the ear needs to pass on all 4 frequencies to pass overall.

The estimate of sensitivity based on only impaired ears belonging to children recruited via audiology services was carried out to quantify the ability of the tests to identify established hearing impairment. The estimate based on only impaired ears belonging to those recruited via schools was carried out to quantify the ability of the tests to identify impairment that has not previously been established.

The ear-level analyses were presented as primary as this reflects the intrinsic accuracy of the tests. Child-level analyses, however, have practical relevance because, regardless of whether just one ear or both ears are impaired, the child will be referred for diagnostic testing. To be included in the child-level analyses a child needed to provide full data on the tests and reference standard for both ears.

Table A1 reports the sensitivity and specificity of PTS and HC for each combination of: ear-level versus child-level analyses; definition of impairment (PTA score ≥30dB on at least one frequency versus mean PTA score ≥30dB across all four frequencies); and subset of impaired children used to calculate sensitivity (all children versus only children recruited from audiology services versus only children recruited from schools).

At ear level the sensitivity is generally higher (especially for the HC) when impairment is defined based on average hearing level across the four frequencies. This might be expected as this definition is easier to pass than our primary definition of impairment, thus resulting in only the more severely impaired ears being included in the impaired group and higher sensitivity. For the same reason the specificity is lower for both tests when impairment status is based on average hearing level across the frequencies presented under the PTA.

Restricting impaired ears in the analysis to only those belonging to children recruited via audiology services (intended cases) increased sensitivity relative to inclusion of all impaired ears. Again, this would be expected as ears of such children would be expected to have more severe hearing loss. Restricting impaired ears in the analysis to only those belonging to children recruited via schools (intended controls) results in lower sensitivity. This latter result is notable because the impaired ears of children with no previously identified hearing impairment are likely to be more representative of the spectrum of impairment in the type of child that we would predominantly want to identify in a school-based setting.

The child-level analyses generally provided a similar pattern of results to the ear-level analyses, except that the PTS test was markedly more sensitive in the child-level analyses when including only impaired children recruited via schools.

Table A1: Accuracy of Pure Tone Screen and HearCheck at ear level and child level across different definitions of impairment status and different subsets of impaired children based on whether recruited via audiology services (intended cases) or schools (intended controls)

**Reference standard**	**Subset of impaired children**	**Pure Tone Screen**		**HearCheck**	
		**Sensitivity**	**Specificity**	**Sensitivity**	**Specificity**
Ear-level analysis					
PTA score ≥30dB on at least one frequency	All children (primary analysis)	94.2%	82.2%	89.0%	86.5%
	Intended cases only	99.1%		97.2%	
	Intended controls only	83.3%		70.8%	
Average PTA score ≥30dB across frequencies	All children	95.7%	76.4%	94.8%	81.8%
	Intended cases only	98.9%		97.8%	
	Intended controls only	84.0%		84.0%	
Child-level analysis					
PTA score ≥30dB on at least one frequency	All children	95.9%	79.8%	88.7%	83.8%
	Intended cases only	98.3%		98.3%	
	Intended controls only	91.9%		73.0%	
Average PTA score ≥30dB across frequencies	All children	97.3%	72.7%	93.3%	78.2%
	Intended cases only	98.1%		98.1%	
	Intended controls only	95.2%		81.0%	
