# Supplementary material for: A directly comparative two-gate case–control diagnostic accuracy study of the pure tone screen and HearCheck screener tests for identifying hearing impairment in school children
Source: BMJ Open. 2017 Jul 11;7(7):e017258. doi: 10.1136/bmjopen-2017-017258 (PMC5541595; doi:10.1136/bmjopen-2017-017258)
Supplement: Author's manuscript [file bmjopen-2017-017258.draft_revisions.pdf]

BMJ Open

A directly comparative two-gate case-control diagnostic accuracy study of the pure tone screen and HearCheck Screener tests for identifying hearing impairment in school children

Journal:	*BMJ Open*
Manuscript ID	bmjopen-2017-017258
Article Type:	Research
Date Submitted by the Author:	11-Apr-2017
Complete List of Authors:	Ukoumunne, Obioha; University of Exeter Medical School, NIHR CLAHRC South West Peninsula (PenCLAHRC)<br>Hyde, Chris; University of Exeter, Exeter Test Group<br>Ozolins, Mara; Nottingham University, NIHR Nottingham Biomedical Research Centre<br>Zhelev, Zhivko; University of Exeter, University of Exeter Medical School<br>Errington, Sam; University of Nottingham, NIHR Nottingham Hearing Biomedical Research Unit<br>Taylor, Rod; University of Exeter, Peninsula Medical School<br>Benton, Claire; Nottingham University Hospitals NHS Trust, Nottingham Audiology Services<br>Moody, Joanne; Ida Darwin Hospital, Community Child Health<br>Cocking, Laura; Plymouth University, Peninsula Clinical Trials Unit at Plymouth University (PenCTU)<br>Watson, Julian; Public Involvement Representative<br>Fortnum, Heather; University of Nottingham, NIHR Nottingham Biomedical Research Centre
**Primary Subject Heading**:	Ear, nose and throat/otolaryngology
Secondary Subject Heading:	Communication, Ear, nose and throat/otolaryngology, Paediatrics
Keywords:	school entry hearing screen, diagnostic accuracy, pure tone screen, HearCheck Screener, pure tone audiogram, hearing impairment

SCHOLARONE™
Manuscripts

A directly comparative two-gate case-control diagnostic accuracy study of the pure tone screen and HearCheck Screener tests for identifying hearing impairment in school children

Author names and institutions

Obioha C Ukoumunne[1], Associate Professor, Medical Statistics

Chris Hyde[2], Professor, Public Health and Clinical Epidemiology

Mara Ozolins[3], Research Fellow, Hearing Research

Zhivko Zhelev[1], Research Fellow, Systematic Reviews

Sam Errington[3], Research Associate, Hearing Research

Rod S Taylor[2], Professor, Health Services Research

Claire Benton[4], Clinical Scientist (Audiology)

Joanne Moody[5], Clinical Scientist (Audiology)

Laura Cocking[6], Senior Data Manager

Julian Watson[7], Public Involvement Representative

Heather Fortnum[3], Associate Professor and Reader, Hearing Research

[1] NIHR CLAHRC South West Peninsula (PenCLAHRC), University of Exeter Medical School, South Cloisters, St Luke's Campus, Heavitree Road, Exeter, EX1 2LU, UK

[2] Institute of Health Research, University of Exeter Medical School, South Cloisters, St Luke's Campus, Heavitree Road, Exeter, EX1 2LU, UK

[3] NIHR Nottingham Hearing Biomedical Research Unit, University of Nottingham, Ropewalk House, 113 The Ropewalk, Nottingham, NG1 5DU, UK

[4] Nottingham Audiology Services, Nottingham University Hospitals, Ropewalk House, 113 The Ropewalk, Nottingham, NG1 5DU, UK

[5] Community Child Health, Ida Darwin Hospital, Fulbourn, Cambridge, CB21 5EE, UK

[6] Peninsula Clinical Trials Unit at Plymouth University (PenCTU), Plymouth University, ITTC Building 1, Plymouth Science Park, 1 Davy Road, Plymouth, PL6 8BX

[7] Public Involvement Representative

For peer review only - http://bmjopen.bmj.com/site/about/guidelines.xhtml

Corresponding author

Dr Obioha C Ukoumunne

Associate Professor in Medical Statistics

NIHR CLAHRC South West Peninsula

University of Exeter Medical School

South Cloisters

St Luke's Campus

Heavitree Road

Exeter EX1 2LU

UK

O.C.Ukoumunne@exeter.ac.uk

Word count (abstract): 300

Word count (main text): 3363

Number of tables: 4

Number of figures: 4

Key words: school entry hearing screen, diagnostic accuracy, pure tone screen, HearCheck Screener, pure tone audiogram, hearing impairment

For peer review only

For peer review only - http://bmjopen.bmj.com/site/about/guidelines.xhtml

Abstract

Objectives: This study directly compared the accuracy of two audiometry-based tests for screening school children for hearing impairment: the currently used test, pure tone screen, and a device newly applied to children, HearCheck Screener.

Design: Two test two-gate case-control diagnostic test accuracy study.

Setting and Participants: Hearing impaired children ("intended cases") aged 4-6 years were recruited between February 2013 and August 2014 from collaborating audiology services. Children with no previously identified impairment ("intended controls") were recruited from Foundation and Year 1 of schools between February 2013 and June 2014 in central England. The reference standard was pure tone audiometry. Tests were administered at Nottingham Hearing Biomedical Research Unit (NHBRU) or, for some intended cases only, in the participant's home.

Main outcome measures: Sensitivity and specificity of the pure tone screen and HearCheck tests based on pure tone audiometry result as reference standard.

Results: 315 children (630 ears) were recruited; 75 from audiology services and 240 from schools. Full test and reference standard data were obtained for 600 ears; 155 ears were classified as truly impaired and 445 as truly hearing based on the pure tone audiometry assessment. Sensitivity was estimated to be 94.2% (95% CI: 89.0% to 97.0%) for pure tone screen and 89.0% (95% CI: 82.9% to 93.1%) for HearCheck (difference = 5.2% favouring pure tone screen; 95% CI: 0.2% to 10.1%; p=0.02). Estimates for specificity were 82.2% (95% CI: 77.7% to 86.0%) for pure tone screen and 86.5% (95% CI: 82.5% to 89.8%) for HearCheck (difference = 4.3% favouring HearCheck; 95% CI: 0.4% to 8.2%; p = 0.02).

Conclusion: Pure tone screen was better than HearCheck with respect to sensitivity, but inferior with respect to specificity. As avoiding missed cases is arguably of greater importance for school entry screening, pure tone screen is probably preferable in this context.

Study registration: Current Controlled Trials: ISRCTN61668996

For peer review only

For peer review only - http://bmjopen.bmj.com/site/about/guidelines.xhtml

Strengths and limitations of the study

- A public involvement representative was a full member of the study team and contributed to the development, conduct and interpretation of the study.
- The audiometry-based screening tests, pure tone screen and HearCheck Screener, were directly compared in the same sample of children.
- The two-gate case-control study design used to identify cases and controls is known to be susceptible to bias.
- The estimates of accuracy of each test might be biased but estimates of comparative accuracy are unlikely to be affected by the design.

For peer review only

For peer review only - http://bmjopen.bmj.com/site/about/guidelines.xhtml

Introduction

Identification of permanent hearing impairment at the earliest possible age is crucial to the development of speech and language and for ensuring the best opportunities for educational achievement and quality of life(1). The highly sensitive and specific universal new-born hearing screen (UNHS) identifies the vast majority of children born with a hearing impairment(2). Due to acquisition, progression or late-onset of hearing impairment and geographical movement of families, however, a significant number of children remain to be identified with a permanent hearing impairment after the new-born period. The school entry screen (SES), a universal hearing screen when children start school, was established in 1955 and remains in place in many parts of the UK. It is considered a safeguard screen to identify hearing impairment.

The 2007 NIHR Health Technology Assessment (HTA)-funded evaluation of the cost-effectiveness of the school entry hearing screen in the UK(3) included a survey of practice which found that the audiometry-based pure tone screen (PTS) test(4) was used in all cases. The diagnostic accuracy studies identified by a review that was part of this evaluation found PTS to generally have higher sensitivity for minimal, mild and greater hearing impairments than alternative tests (tympanometry, otoscopy, transient-evoked otoacoustic emission tests, parent questionnaires, spoken word tests) for which evidence was identified(3). These comparisons were, however, indirect and highly susceptible to confounding. Furthermore, most of these accuracy studies were undertaken in populations where the prevalence of undetected hearing impairment was considerably greater than that likely to be encountered in a system where a universal new-born hearing screening programme is in place. The estimates of accuracy were also based on small sample sizes. A relatively new device, HearCheck Screener (HC)(5), also audiometry-based, came onto the market in 2005 as a tool for screening for hearing impairment in adults in a general practice setting. It is less comprehensive and flexible than PTS but has the potential to be a quicker test in the school setting. It has not previously been assessed as a tool for screening children in the UK.

The objective of this study was to compare the diagnostic accuracy of PTS and HC tests for hearing impairment of any type at or around school entry using full pure tone audiometry (PTA) as the reference standard(6). The study was part of a HTA-funded programme of

For peer review only - http://bmjopen.bmj.com/site/about/guidelines.xhtml

For peer review only

work with the wider aim of assessing the effectiveness and cost-effectiveness of the school entry hearing screen(7). The full study protocol is available from the authors on request.

Methods

Participants

This diagnostic test accuracy study used a directly comparative two-gate case-control design(8).

Intended cases

Hearing impaired children aged 4-6 years between February 2013 and August 2014 were identified by collaborating audiology services (centres) in central England. They had permanent sensorineural or conductive hearing impairment averaged across the four frequencies 0.5, 1, 2 and 4kHz, either bilaterally (average of 20-60dB HL) or unilaterally (any level ≥20dB HL). Children were identified by the paediatric audiologist in each centre. The reference standard was pure tone audiomentry (PTA), and potential recruits were excluded if there was no record of a PTA in the previous 12 months or planned for the following three months, and the family was unwilling to travel to their local service or to Nottingham to undergo the assessment. Eligible children for whom parents provided agreement to take part were invited to undergo the two screening tests (PTS and HC), either in their own homes, or at Nottingham Hearing Biomedical Research Unit (NHBRU), depending on their preference.

Intended controls

Children with no previously identified hearing impairment were recruited from the Foundation Year and Year 1 of schools in the Nottingham area (central England), between February 2013 and June 2014. The study researchers provided an agreed letter of invitation and information packs for the school. Children for whom agreement to take part was provided were invited to undergo the two screening tests and the PTA reference standard assessment at NHBRU.

Procedures

Written informed consent was obtained from the parent or legal guardian before the child entered the study. Test data for all children and reference standard data for all intended

For peer review only - http://bmjopen.bmj.com/site/about/guidelines.xhtml

controls were collected specifically for this study. For most of the intended cases the reference standard data were based on previous assessments otherwise unconnected to the study.

Pure tone screen test

Headphones were placed over the child's ears and then pure tones presented across the key frequencies for speech understanding in the order 1kHz, 2kHz, 4kHz and 0.5kHz. Each tone was held for 2 to 3 seconds, with staggered pauses. All four frequencies were tested in one ear before being tested in the other. To pass the screen in a given ear the child needed to respond to 2 out of 3 presentations of each frequency at 20dB HL to pass. The researcher was positioned to ensure they had a clear view of the child without giving any visual cues throughout the test. The child was instructed to place a ball onto a frame every time they heard a sound, however quiet. Hearing aids, glasses, hairbands and earrings were removed where relevant. A familiarisation tone (1kHz at 60dB HL) was presented to ensure the child had understood the instructions.

HearCheck Screener test

The HC screener was placed over the child's ear and an automatic sequence of pure tones played at three levels at each of the frequencies 1 kHz (55dB, 35dB and 20dB) and 3 kHz (75dB, 55dB, 35dB). To pass the screen in a given ear the child needed to respond to all six tones. The child indicated, usually by raising their hand, that they had heard each tone. The child was asked to remove hearing aids, and also glasses and earrings if necessary for a good fit. A disposable cardboard ear cover was put onto the HC for each ear. The HC was held against the first ear to be tested, often holding the child's head still with the free hand. The button was pressed and the first three tones were allowed to play for the first frequency. The button was then pressed again for the remaining three tones for the second frequency. The procedure was repeated on the other ear.

Reference standard

After intended cases had their appointment, the researcher phoned the audiologist, asking them to post their most recent PTA results to NHBRU. For intended controls PTA was carried out in NHBRU at the same session as the screening tests, using the audiometer in a sound-proofed booth with the child sat facing away from the equipment. PTA testing followed standard British Society of Audiology (BSA) recommended procedure(6) without

For peer review only - http://bmjopen.bmj.com/site/about/guidelines.xhtml

otoscopic examination or masking, for air conduction only. Hearing impairment was considered present when the PTA reference standard threshold was ≥30dB on at least one of the four frequencies (0.5 kHz, 1 kHz, 2 kHz and 4 kHz) and considered absent when the reference threshold was <30dB on all four frequencies.

Other procedural details

Equipment was calibrated as per manufacturer's instructions. There was generally less background noise than would be expected in schools. The researchers were trained in administering the PTA and PTS by the audiologists in the Children's Hearing Assessment Centre in Nottingham, using a mixture of observation, practice on children and feedback.

The order of administering the two screening tests and which researcher undertook them was determined randomly. For intended cases one researcher performed the PTS and another researcher performed the HC. For intended controls one researcher carried out both the screening tests and then another researcher performed the PTA measurement. We sought to blind the second researcher to the results of the first test(s) by asking them to leave the room. The PTA result obtained from the audiologist for intended cases was examined only after the results of the screening tests were known.

Ethical approval was granted by the West Midlands, Staffordshire Research Ethics Committee (Ref: 106333).

Statistical analysis

The target sample size was 80 hearing impaired children and 160 children with no hearing impairment. Eighty (80) impaired children is large enough to estimate a sensitivity of 80% with a margin of error of 10.4% based on the lower bound of the 95% confidence interval and 160 children without impairment is large enough to estimate a specificity of 80% with a margin of error of 7.0%. Accuracy was evaluated using the ear as the unit of analysis. In the main analysis, irrespective of intended case or control status, ears were defined as truly hearing impaired or not based on actual PTA reference standard results. Analyses were carried out using Stata statistical software (version 13.1). We reported the absolute difference in percentages between the PTS and HC for each of sensitivity and specificity with 95% confidence intervals and McNemar's test p-value (using the Stata command `mcc`). We used analytical methods that recognise the correlation between results of ears belonging to the same child. Details of further exploratory analyses are provided in the Appendix.

For peer review only - http://bmjopen.bmj.com/site/about/guidelines.xhtml

Public involvement

The research question originates from a call from the NIHR HTA funding stream to evaluate the diagnostic accuracy of hearing tests and cost-effectiveness of school entry hearing screening programmes. We recruited Julian Watson, a parent of a child who has experienced conductive hearing impairment, to be a full member of the study team and an author on this paper. His input included comments on information literature for participating parents; development of methodology and the conduct of the study (e.g., addressing recruitment challenges); attending study meetings; and critical comments and suggestions on the final study report and this paper. We also included on our study steering committee a representative from the National Deaf Children's Society. Parents of participating children were offered the opportunity to receive a lay summary of the findings at the end of the study. Almost all parents took up the offer and it was sent to them.

Results

Participants

Intended cases were recruited from 14 audiology services. We received 86 replies from 379 invitations sent by the audiologists. Eight children were ineligible, being outside the required age range. We were unable to contact one of the initial respondents, and we were unable to see a further two children due to researcher illness just before the close of recruitment. We recruited and tested the remaining 75 children (19.8% of those invited) (Figure 1).

Intended controls were recruited from 51 of the 164 schools in the Nottingham area that were invited by post to take part. The 51 schools between them gave information packs to the parents of 2787 children, of whom 291 (10.4%) replied, confirming they would like to participate. An additional 11 siblings of children who attended the appointment but who did not receive the invitation were in the correct age range and parents agreed for them to take part. Eight of the 302 invited children were subsequently found to be ineligible for the study (one was too old, six already had hearing problems identified, one replied after recruitment closed), 11 changed their minds about taking part, and we were unable to see 43 either because we could not make an appointment (mostly not contactable) or they did not attend the arranged appointment. The remaining 240 children were recruited as intended controls and seen for study appointments (Figure 2).

For peer review only - http://bmjopen.bmj.com/site/about/guidelines.xhtml

For peer review only

Table 1 summarises the demographic characteristics of participating children by whether they were recruited via audiology services (intended cases) or via schools (intended controls). The groups were similar with respect to gender and age.

Intended controls completed the tests and reference standard on the same day. For intended cases, reference standard data were already available prior to the tests being administered for 65 children and for the remaining 10 children a reference standard assessment took place after administering the PTS and HC. The median time interval between reference standard and test assessment was 16 weeks. There were no adverse events from performing the tests and the reference standard.

Number of ears with impaired or non-impaired hearing

Of the 630 recruited ears, 600 (95.2%) provided full data on the PTS and HC tests and scores for all four frequencies of the PTA reference standard and were included in the main analyses. Two hundred and ninety five (295) children provided full data on both ears and another 10 provided full data on just one ear. There were no indeterminate screening test or PTA results. The PTA reference standard categorised 155 ears as impaired and 445 as not impaired. The mean (SD) hearing level in dB at frequencies 0.5 kHz, 1 kHz, 2 kHz and 4 kHz, was 43.1 (21.0), 45.0 (22.5), 46.2 (25.0) and 49.0 (24.2), respectively, for impaired ears and 9.4 (7.4), 4.7 (7.5), 3.7 (6.6) and 4.9 (8.1), respectively, for hearing ears. One hundred and seven (107) of the impaired ears belonged to children recruited from audiology services and the remaining 48 ears belonging to children with no previously identified hearing loss.

Figures 3 and 4 present flowcharts that describe the number of impaired ears (based on PTA ≥30dB on at least one of the 4 frequencies) and hearing ears that passed and referred on the PTS and HC tests, respectively.

Sensitivity and specificity

Table 2 summarises the relationship between the PTS and HC test results separately for impaired ears, hearing ears and ears for which information on the reference standard was missing. The figures highlighted in grey indicate the impaired ears that were used in the calculation of sensitivity and the hearing ears used in the calculation of specificity.

Table 3 reports the sensitivity and specificity of the screening tests. The sensitivity was 94.2% for PTS and 89.0% for HC. The 95% confidence interval for sensitivity indicates that

For peer review only - http://bmjopen.bmj.com/site/about/guidelines.xhtml

we can be fairly certain that the true sensitivity is no lower than 89% for PTS and 83% for HC. The McNemar's test result (p = 0.02) indicates evidence that the true sensitivity is greater for PTS than for HC. The estimates of specificity were 82.2% for PTS and 86.5% for HC, with evidence provided by McNemar's test that the true specificity is higher for HC than PTS (p = 0.02).

False negatives

The mean (SD) hearing level across the 4 test frequencies on the PTA reference standard for the 19 ears that passed one or both of the screening tests but referred by the PTA was 28 (9) dB compared to 48 (21) dB for the remaining 136 impaired ears that referred on both PTS and HC. This indicates that impairment was less severe for the false negatives than the true positives.

Discussion

The main finding of our study is that PTS was better than HC with respect to sensitivity (5.2% in favour of PTS; 95% CI: 0.2%, 10.1%; p=0.02), but inferior with respect to specificity (4.3% in favour of HC; 95% CI: 0.4% to 8.2%; p = 0.02).

The two-gate diagnostic test accuracy study design employed is widely acknowledged to be open to bias in the assessment of accuracy(8). However, given the extremely low prevalence of hearing impairment in a school entry population, approximately 0.5%(7), this was felt to be the only feasible design. In a traditional accuracy study where the test and reference standard are administered to all participants identified from a single source ("single-gate") with no advance knowledge of their true disease status(8), 16,000 school children in the UK would need to have been recruited to identify our target of 80 cases of hearing impairment and so offer the same precision for measuring sensitivity. Also, we believe that although the bias might lead to an overestimate of accuracy for each test individually, it might have less impact on comparison of accuracy as both tests would be subject to any overestimation. Measuring PTS and HC accuracy in the near to ideal conditions in this study as opposed to the nosier circumstances that would prevail in schools is also likely to lead to inflation of accuracy of the tests individually.

For peer review only - http://bmjopen.bmj.com/site/about/guidelines.xhtml

We remain confident that there are no other studies that directly compare PTS with HC. Indeed there are very few directly comparative accuracy studies of any of the potential screening tests for hearing impairment(7). Our findings are consistent with indirect comparison of PTS with other tests which suggest that PTS is superior(3, 7). What this study adds is that when PTS is used in a standard manner and HC is used in the manner designed by the manufacturers, there is a trade-off between sensitivity and specificity and a threshold effect may be part of the apparent difference between the two tests. However, given that thresholds are fixed, particularly for HC, it is reasonable to consider which of PTS and HC in the conventional forms used in the study would be preferable in practice. Some further insight into this is given by reflecting on the absolute numbers of false positives and false negatives when the differences in accuracy are applied to a population with a prevalence of hearing impairment similar to one which might be observed in practice. This is done in Table 4 where the accuracy estimates are applied to a population of 10,000 with a prevalence of hearing impairment of 0.5% (i.e., 50 with impairment). In most tests used for screening and triage, there is a preference for avoiding false negatives, because it may take many years for "missed" individuals to re-engage with the health system, by which time the opportunity to successfully intervene may have been lost. However, as Table 4 shows the number of false positives (1771 and 1343 for PTS and HC, respectively) is so much larger than the number of false negatives (3 and 5 for PTS and HC, respectively), that it is reasonable to question whether the cumulative added costs of unnecessary testing in false positives have reached a point where they outweigh the cumulative benefits of avoiding a much smaller number of false negatives. This is particularly true where the nature of the hearing impairment is milder in the missed cases than in those who correctly tested positive, as we found in this study. We did, however, note in another component study of this programme of work that the number of screened children attending for diagnostic evaluation was much less than would be implied by test specificity, suggesting strongly that the number of false positives in a screening programme is much less than would be indicated by test specificity in isolation(7). This is because in a screening programme, those initially testing as impaired may have their screening result rechecked or reviewed before being finally sent for diagnostic evaluation. So the impact of false positives is overstated if one relies on test specificity in isolation rather than considering the specificity of the programme as a whole.

On balance, therefore, we retain the view that the reduced number of false negatives associated with PTS use (2 fewer per 10,000 children screened – Table 4) does outweigh the

For peer review only - http://bmjopen.bmj.com/site/about/guidelines.xhtml

advantage in terms of test specificity apparently offered by HC which has 428 fewer false positives per 10,000 screened. The implications for practice are thus that where school entry hearing screening is still being used or is under consideration, PTS would be the better screening tool. We do note, however, that recently concerns have been expressed about the likely cost-effectiveness of SES relative to a system reliant on ad hoc identification of possible hearing impairment and referral for diagnostic evaluation, although this is an early finding needing confirmation(7).

In terms of implications for research, whilst we note that this study gives robust information about the choice between PTS and HC, there are other alternative tests such as automated audiometry-based hearing screening systems installed on laptops or hand-held devices(9-14), otoacoustic emissions (OAEs)(15-19) and Automated Auditory Brainstem Response (AABR)(20). Although they have been the object of direct comparison of accuracy, further research is necessary to provide more robust evidence of their comparative performance, feasibility and cost-effectiveness in different country-specific contexts. Furthermore, we would suggest that if the arguments for the validity of comparative two-gate accuracy studies as used here are accepted this would be an appropriate and efficient means to evaluate relative accuracy in the future. Incorporating such direct comparisons into on-going systematic reviews of single test accuracy studies should also be anticipated. Finally, the work we have done here on accuracy of the hearing screening tests should be extended to estimate the accuracy of the school entry hearing screening programme itself.

For peer review only

References

1. Yoshinaga-Itano C., Coulter D., Thomson V. The Colorado Newborn Hearing Screening Project: effects on speech and language development for children with hearing loss. J Perinatol. 2000;20:S132–7.

2. Kennedy C., McCann D., Campbell M.J., Kimm L., Thornton R. Universal newborn screening for permanent childhood hearing impairment: an 8-year follow-up of a controlled trial. Lancet. 2005;336:660-2.

3. Bamford J., Fortnum H., Bristow K., Smith J., Vamvakas G., Davies L., et al. Current practice, accuracy, effectiveness and cost-effectiveness of the school entry hearing screen. Health Technol Assess. 2007;11(32).

4. Ewing A. The sweep frequency method of making screening tests of the hearing of school children. BMJ. 1955;4904:41–2.

5. Siemens. HearCheck Screener Userguide. 2012.

6. British Society of Audiology. Pure-tone air-conduction and bone-conduction threshold audiometry with and without masking. 2012.

7. Fortnum H., Ukoumunne O.C., Hyde C., Taylor R.S., Ozolins M., Errington S., et al. A programme of studies including assessment of diagnostic accuracy of school hearing screening tests and a cost-effectiveness model of school entry hearing screening programmes. Health Technol Assess. 2016;20(36).

8. Rutjes A.W.S., Reitsma J.B., Vandenbroucke J.P., Glas A.S., Bossuyt P.M.M. Case–control and two-gate designs in diagnostic accuracy studies. Clin Chem. 2005;51:1335 - 41.

9. Kam A.C., Li L.K., Yeung K.N., Wu W., Huang Z., Wu H., et al. Automated hearing screening for preschool children. Journal of Medical Screening. 2014;21:71-5.

10. Kam A.C.S., Gao H., Li L.K.C., Zhao H., Qiu S., Tong M.C.F. Automated hearing screening for children: A pilot study in China. International Journal of Audiology. 2013;52:855-60.

For peer review only

For peer review only - http://bmjopen.bmj.com/site/about/guidelines.xhtml

11. Mahomed-Asmail F., Swanepoel de W., Eikelboom R.H., Myburgh H.C., Hall J. Clinical validity of HearScreen smartphone hearing screening for school children. Ear and Hearing. 2016;37:e11-7.

12. McPherson B., Law M.M.S., Wong M.S.M. Hearing screening for school children: comparison of low-cost, computer-based and conventional audiometry. Child: Care, Health and Development. 2010;36:323-31.

13. Swanepoel D.W., Myburgh H.C., Howe D.M., Mahomed F., Eikelboom R.H. Smartphone hearing screening with integrated quality control and data management. International Journal of Audiology. 2014;53:841-9.

14. Wu H. A new hearing screening system for preschool children. International Journal of Pediatric Otorhinolaryngology. 2014;78:290-5.

15. Cloete T-L., Wilson W.J., Petersen L., Kathard H. Identifying a context-effective school hearing screening test: An emic/etic framework. International Journal of Audiology. 2015;54:605-12.

16. Georgalas C., Xenellis J., Davilis D., Tzangaroulakis A., Ferekidis E. Screening for hearing loss and middle-ear effusion in school-age children, using transient evoked otoacoustic emissions: a feasibility study. Journal of Laryngology and Otology. 2008;122:1299-304.

17. Lyons A., Kei J., Driscoll C. Distortion product otoacoustic emissions in children at school entry: a comparison with pure-tone screening and tympanometry results. Journal of the American Academy of Audiology. 2004;15:702-15.

18. Smiley D.F., Shapley K., Eckl D., Nicholson N. Comparison of pure-tone and distortion product otoacoustic emission screenings in school-age children. Journal of Educational Audiology. 2012;18:32-7.

19. Yin L., Bottrell C., Clarke N., Shacks J., Poulsen M.K. Otoacoustic emissions: a valid, efficient first-line hearing screen for preschool children. Journal of School Health. 2009;79:147-52.

For peer review only

20. Soares M., Nakazawa M., Ishikawa K., Sato T., Honda K. Hearing screening for Japanese children and young adults using the automated auditory brainstem response. Auris Nasus Larynx. 2014;41:17-21.

For peer review only

For peer review only - http://bmjopen.bmj.com/site/about/guidelines.xhtml

Footnotes

Acknowledgements

We would like to thank: the participating families and schools; the audiologists at each centre for sending out invitations for the study; Venessa Vas for helping to complete the screening; Elliot Carter, Brendan Lane and Paul Williams from PenCTU for developing the data entry websites; Brian Wainman and Mark Warner from PenCTU for entering data and assisting with the data; former project team member Vasilis Nikolaou; members of and observers on the study steering committee (John Bamford, John Fitzgerald, Vicki Kirwin, Kevin Munro, Kate Northstone, Karen Smith, Eldon Spackman, Maria Koufali, Stacey Arland, Angela Shone); and Jon Deeks for critical comments on the conduct and reporting of the study.

Author contributions

The protocol was developed and funding obtained by OCU, CH, RST, CB, JM and HF. HF was chief investigator with overall responsibility for the conduct of the study. OCU, CH, MO, ZZ, SE, RST, CB, JM, LC, JW and HF contributed to revisions of the design and conduct of the study. MO and SE managed the study and collected the diagnostic accuracy data. CB invited case children to the study. ZZ conducted an updated systematic review of the diagnostic accuracy of hearing screening tests. LC co-ordinated database design and development, data validation and data export. JW was the PPI representative on the study. OCU developed the statistical analysis plan which was critically revised by all authors. OCU undertook the analyses. OCU drafted the manuscript which was critically revised by all authors. OCU is the guarantor of the manuscript.

Declaration of competing interests

All authors have completed the Unified Competing Interest form at www.icmje.org/coi_disclosure.pdf (available on request from the corresponding author) and declare: no authors have support from any company for the submitted work; no authors have financial relationships with any organisations that might have an interest in the submitted work in the previous three years; no authors have any other relationships or activities that could appear to have influenced the submitted work.

For peer review only

Ethical Approval

Ethical approval was granted by the West Midlands, Staffordshire Research Ethics Committee (Ref: 106333). Written informed consent was obtained from the parent or legal guardian before the child entered the study.

Funding/support

The study was funded by the National Institute for Health Research Health Technology Assessment Programme (project no. 10/63/03). Obioha Ukoumunne, Zhivko Zhelev, Jaime Peters and Rod Taylor are supported by the National Institute for Health Research (NIHR) Collaboration for Leadership in Applied Health Research and Care South West Peninsula (PenCLAHRC). Chris Hyde is supported by the University of Exeter. Mara Ozolins and Sam Errington were supported by a grant from the NIHR Health HTA programme (10/63/03). Claire Benton is funded by Nottingham University Hospitals NHS Trust. Joanne Moody is funded by CCS NHS Trust and supported by Addenbrookes Hospital, Cambridge University Hospitals Foundation Trust. Heather Fortnum was funded by the University of Nottingham and supported by the NIHR NHBRU. The views and opinions expressed therein are those of the authors and do not necessarily reflect those of the HTA programme, the NHS, the NIHR or the Department of Health.

Role of the study sponsor and funder

Neither the funding body (National Institute for Health Research Health Technology Assessment Programme) nor the sponsor (University of Nottingham) had a role in the design of the study; collection, analysis and interpretation of the data; writing of the paper or the decision to submit it for publication.

Independence of researchers from funder

All researchers worked independently from the funder.

Data Access and responsibility

All authors had full access to all of the data (including statistical reports and tables) in the study and can take responsibility for the integrity of the data and the accuracy of the data analysis.

For peer review only

Transparency declaration

The lead author, Obioha Ukoumunne, affirms that the manuscript is an honest, accurate, and transparent account of the study being reported; that no important aspects of the study have been omitted; and that any discrepancies from the study as planned (and, if relevant, registered) have been explained.

Data sharing

Participant level data, the full dataset and statistical code are available from the corresponding author. Consent for this was not obtained but the presented data are anonymised and risk of identification is low.

Copyright

The Corresponding Author has the right to grant on behalf of all authors and does grant on behalf of all authors, a worldwide licence to the Publishers and its licensees in perpetuity, in all forms, formats and media (whether known now or created in the future), to i) publish, reproduce, distribute, display and store the Contribution, ii) translate the Contribution into other languages, create adaptations, reprints, include within collections and create summaries, extracts and/or, abstracts of the Contribution, iii) create any other derivative work(s) based on the Contribution, iv) to exploit all subsidiary rights in the Contribution, v) the inclusion of electronic links from the Contribution to third party material where-ever it may be located, and vi) licence any third party to do any or all of the above.

For peer review only

Table 1: Demographic characteristics of children by recruitment source

Characteristic	Recruited via audiology services (intended cases) (N = 75)	Recruited via schools (intended controls) (N = 240)
Male, n (%)	38 (51)	117 (49)
Age, mean (SD; range)	5.4 (0.9; 3.9 to 7.0)	5.4 (0.6; 4.0 to 6.9)
Ethnicity		
White, n (%)	61 (81)	189 (79)
Black, n (%)	2 (3)	14 (6)
Asian, n (%)	11 (15)	10 (4)
Mixed, n (%)	1 (1)	22 (9)
Other, n (%)	0 (0)	5 (2)

For peer review only

Table 2: Cross tabulation of Pure Tone Screen (PTS) versus HearCheck (HC) test results

		PTS test results			
		**Refer**	**Pass**	**Missing**	**Total**
		**Impaired**			
**HC**	**Refer**	136	2	1	139
**test**	**Pass**	10	7	0	17
**results**	**Missing**	0	0	0	0
	**Total**	146	9	1	156
		**Hearing**			
**HC**	**Refer**	34	26	0	60
**test**	**Pass**	45	340	0	385
**results**	**Missing**	0	1	0	1
	**Total**	79	367	0	446
		**Missing**			
**HC**	**Refer**	13	2	1	16
**test**	**Pass**	3	2	1	6
**results**	**Missing**	2	0	4	6
	**Total**	18	4	6	28

For peer review only - http://bmjopen.bmj.com/site/about/guidelines.xhtml

Table 3: Accuracy of Pure Tone Screen (PTS) and HearCheck (HC)

Measure	Pure Tone Screen estimate (95% CI)	HearCheck estimate (95% CI)	Difference in accuracy (PTS – HC) estimate (95% CI)	p value
**Sensitivity**	94.2% (89.0% to 97.0%)	89.0% (82.9% to 93.1%)	5.2% (0.2% to 10.1%)	0.02
**Specificity**	82.2% (77.7% to 86.0%)	86.5% (82.5% to 90.0%)	-4.3% (-8.2% to -0.4%)	0.02

For peer review only

For peer review only - http://bmjopen.bmj.com/site/about/guidelines.xhtml

Table 4: Frequency of test results per 10,000 screened in a hypothetical population

Test results	Test		Difference (PTS - HC)
	Pure Tone Screen (PTS)	HearCheck (HC)	
True positives	47	45	2
True negatives	8179	8607	
False positives	1771	1343	428
False negatives	3	5	

For peer review only

For peer review only - http://bmjopen.bmj.com/site/about/guidelines.xhtml

Figure 1: Numbers of intended case children in the diagnostic accuracy study

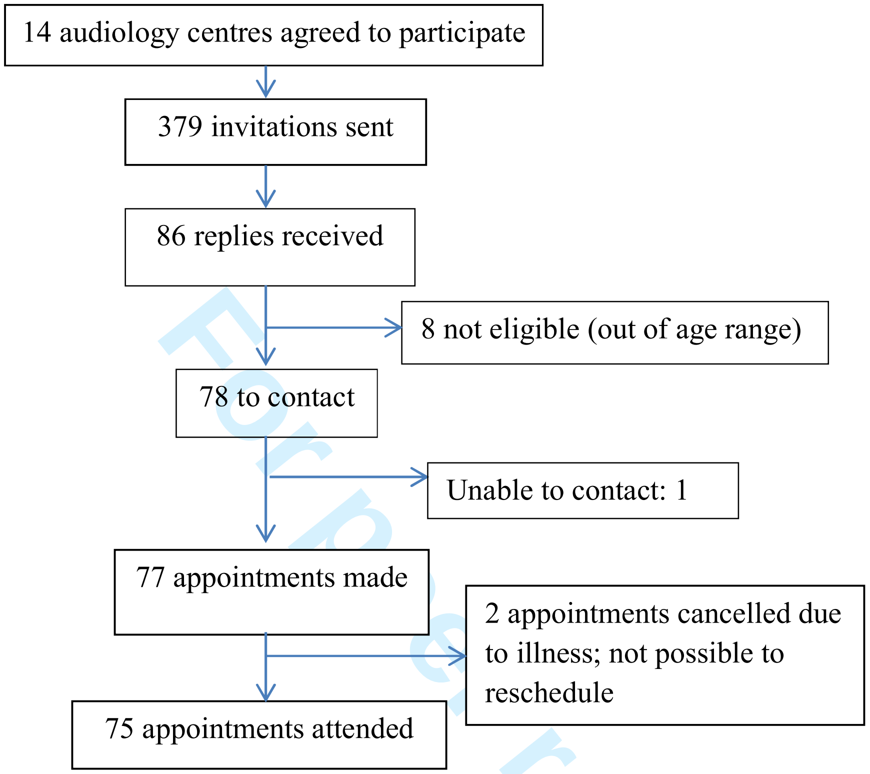

Figure 2: Numbers of intended control children in the diagnostic accuracy study

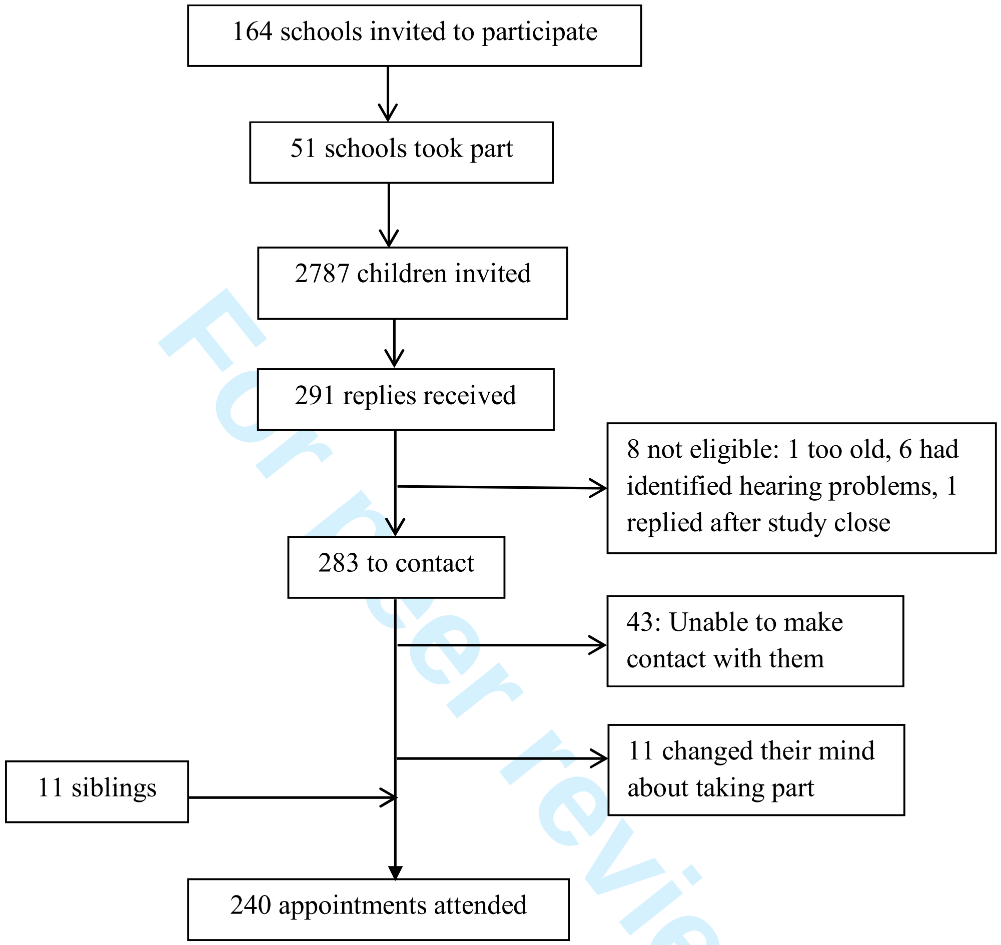

Figure 3: Pure Tone Screen (PTS) test results at ear level by hearing impairment status (PTA)

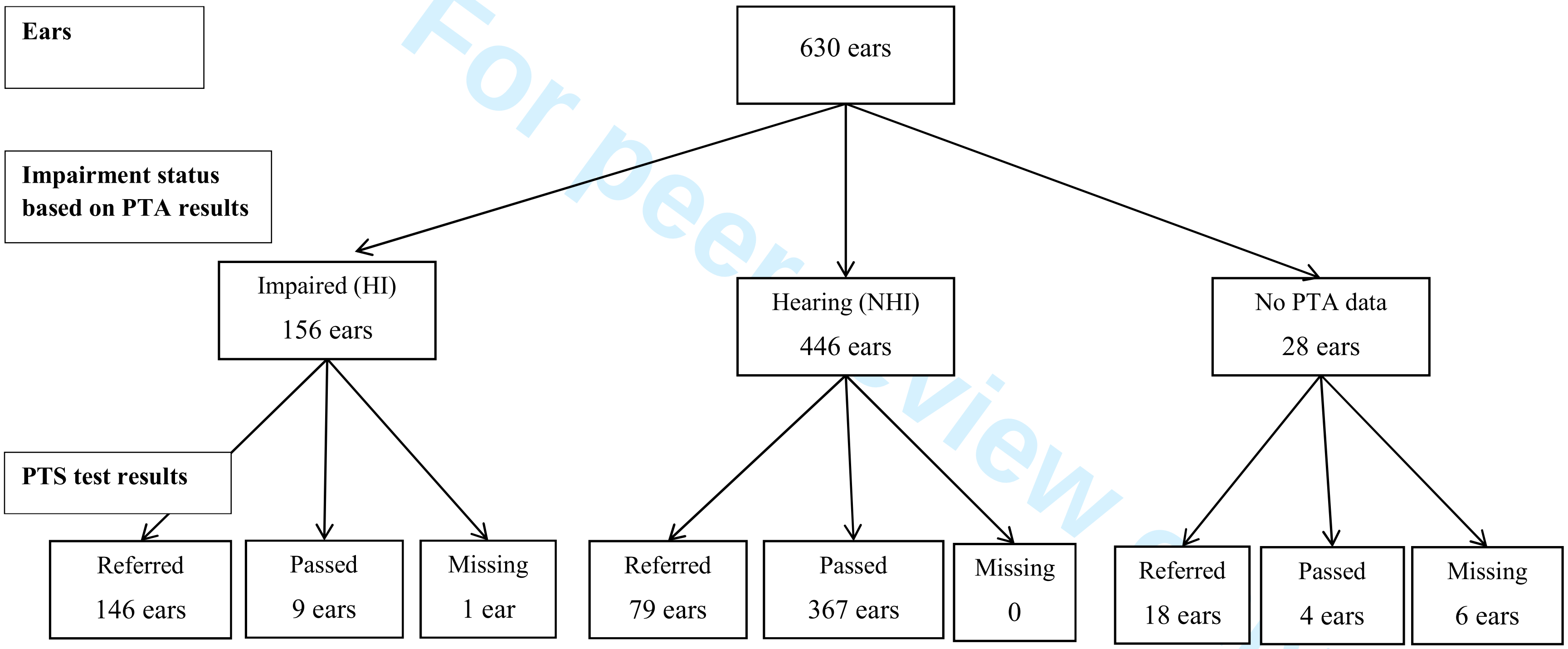

For peer review only - http://bmjopen.bmj.com/site/about/guidelines.xhtml

Figure 4: HearCheck (HC) test results at ear level by hearing impairment status (PTA)

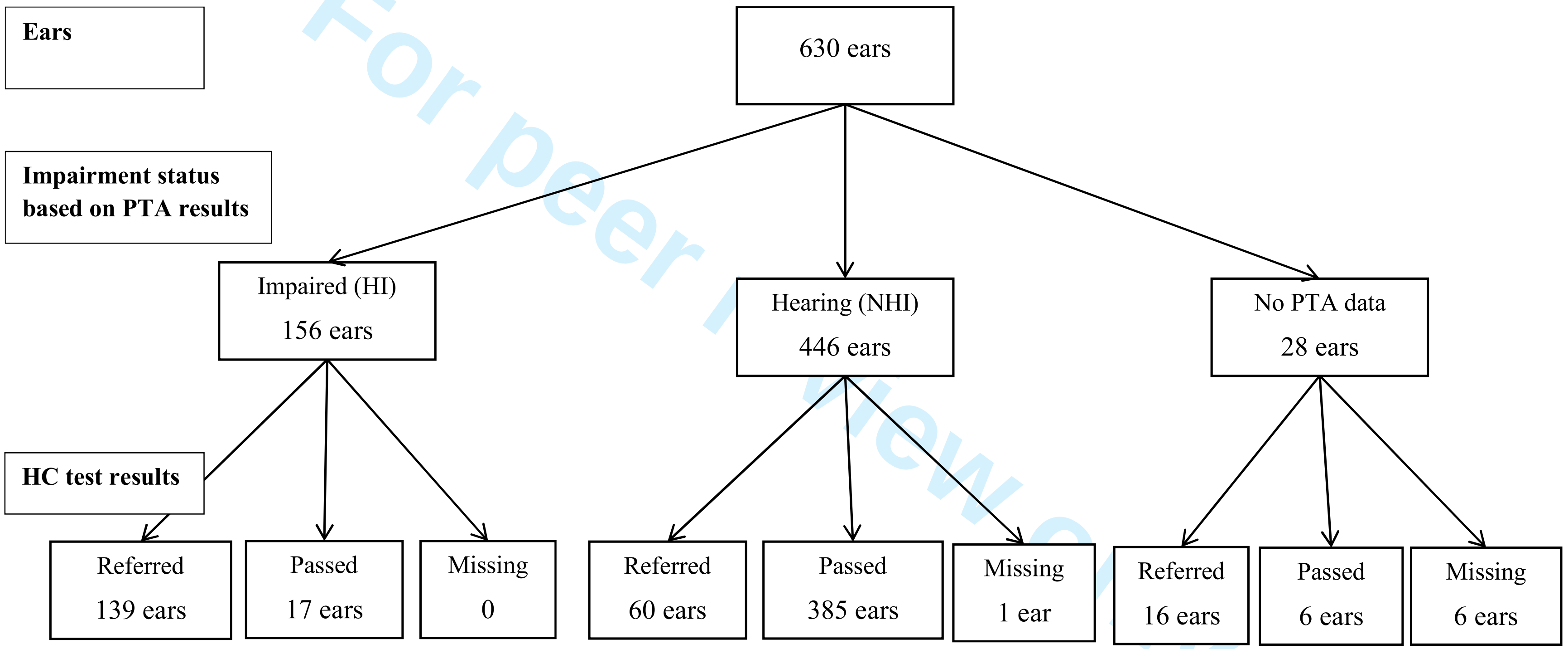

For peer review only - http://bmjopen.bmj.com/site/about/guidelines.xhtml

Appendix

Variability in diagnostic accuracy across different scenarios

The primary analyses reported in the main text is for accuracy at ear level of the pure tone screen (PTS) and HearCheck Screener (HC) tests for distinguishing hearing impaired ears from non-impaired ears. Hearing impairment was defined as present when the PTA reference standard threshold was ≥30dB on at least one of the four frequencies (0.5 kHz, 1 kHz, 2 kHz and 4 kHz) and absent when the reference threshold was <30dB on all four frequencies. All impaired ears were used to calculate test sensitivity regardless of whether belonging to children recruited via audiology services (intended cases) or via schools (intending controls). In addition, we performed further exploratory analyses estimating accuracy of the tests in alternative scenarios:

a) when impairment was defined as present when the mean PTA threshold across the four frequencies was ≥30dB and absent when the mean threshold was <30dB,
b) when only impaired ears (children) that were recruited via audiology services were used to estimate sensitivity and when only impaired ears (children) that were recruited via schools were used to estimate sensitivity,
c) at child level for distinguishing between hearing impaired children and non-impaired children.

The primary definition of hearing impairment is the stricter (i.e., the PTA reference standard is harder to pass) than the one based on mean hearing level across the 4 frequencies, since under the former the ear needs to pass on all 4 frequencies to pass overall.

The estimate of sensitivity based on only impaired ears belonging to children recruited via audiology services was carried out to quantify the ability of the tests to identify established hearing impairment. The estimate based on only impaired ears belonging to those recruited via schools was carried out to quantify the ability of the tests to identify impairment that has not previously been established.

The ear-level analyses were presented as primary as this reflects the intrinsic accuracy of the tests. Child-level analyses, however, have practical relevance because, regardless of whether just one ear or both ears are impaired, the child will be referred for diagnostic testing. To be included in the child-level analyses a child needed to provide full data on the tests and reference standard for both ears.

For peer review only

Table A1 reports the sensitivity and specificity of PTS and HC for each combination of: ear-level versus child-level analyses; definition of impairment (PTA score ≥30dB on at least one frequency versus mean PTA score ≥30dB across all four frequencies); and subset of impaired children used to calculate sensitivity (all children versus only children recruited from audiology services versus only children recruited from schools).

At ear level the sensitivity is generally higher (especially for the HC) when impairment is defined based on average hearing level across the four frequencies. This might be expected as this definition is easier to pass than our primary definition of impairment, thus resulting in only the more severely impaired ears being included in the impaired group and higher sensitivity. For the same reason the specificity is lower for both tests when impairment status is based on average hearing level across the frequencies presented under the PTA.

Restricting impaired ears in the analysis to only those belonging to children recruited via audiology services (intended cases) increased sensitivity relative to inclusion of all impaired ears. Again, this would be expected as ears of such children would be expected to have more severe hearing loss. Restricting impaired ears in the analysis to only those belonging to children recruited via schools (intended controls) results in lower sensitivity. This latter result is notable because the impaired ears of children with no previously identified hearing impairment are likely to be more representative of the spectrum of impairment in the type of child that we would predominantly want to identify in a school-based setting.

The child-level analyses generally provided a similar pattern of results to the ear-level analyses, except that the PTS test was markedly more sensitive in the child-level analyses when including only impaired children recruited via schools.

For peer review only

Table A1: Accuracy of Pure Tone Screen and HearCheck at ear level and child level across different definitions of impairment status and different subsets of impaired children based on whether recruited via audiology services (intended cases) or schools (intended controls)

Reference standard	Subset of impaired children	Pure Tone Screen		HearCheck	
		Sensitivity	Specificity	Sensitivity	Specificity
Ear-level analysis					
PTA score ≥30dB on at least one frequency	All children (primary analysis)	94.2%	82.2%	89.0%	86.5%
	Intended cases only	99.1%		97.2%	
	Intended controls only	83.3%		70.8%	
Average PTA score ≥30dB across frequencies	All children	95.7%	76.4%	94.8%	81.8%
	Intended cases only	98.9%		97.8%	
	Intended controls only	84.0%		84.0%	
Child-level analysis					
PTA score ≥30dB on at least one frequency	All children	95.9%	79.8%	88.7%	83.8%
	Intended cases only	98.3%		98.3%	
	Intended controls only	91.9%		73.0%	
Average PTA score ≥30dB across frequencies	All children	97.3%	72.7%	93.3%	78.2%
	Intended cases only	98.1%		98.1%	
	Intended controls only	95.2%		81.0%	

For peer review only - http://bmjopen.bmj.com/site/about/guidelines.xhtml

Section & Topic	No	Item	Reported on page #
**TITLE OR ABSTRACT**			
	**1**	Identification as a study of diagnostic accuracy using at least one measure of accuracy (such as sensitivity, specificity, predictive values, or AUC)	1, 3
**ABSTRACT**			
	**2**	Structured summary of study design, methods, results, and conclusions (for specific guidance, see STARD for Abstracts)	3
**INTRODUCTION**			
	**3**	Scientific and clinical background, including the intended use and clinical role of the index test	5
	**4**	Study objectives and hypotheses	5
**METHODS**			
Study design	**5**	Whether data collection was planned before the index test and reference standard were performed (prospective study) or after (retrospective study)	6
Participants	**6**	Eligibility criteria	6
	**7**	On what basis potentially eligible participants were identified (such as symptoms, results from previous tests, inclusion in registry)	6
	**8**	Where and when potentially eligible participants were identified (setting, location and dates)	6
	**9**	Whether participants formed a consecutive, random or convenience series	6
Test methods	**10a**	Index test, in sufficient detail to allow replication	7
	**10b**	Reference standard, in sufficient detail to allow replication	7, 8
	**11**	Rationale for choosing the reference standard (if alternatives exist)	no alternatives
	**12a**	Definition of and rationale for test positivity cut-offs or result categories of the index test, distinguishing pre-specified from exploratory	7
	**12b**	Definition of and rationale for test positivity cut-offs or result categories of the reference standard, distinguishing pre-specified from exploratory	7, 8
	**13a**	Whether clinical information and reference standard results were available to the performers/readers of the index test	8
	**13b**	Whether clinical information and index test results were available to the assessors of the reference standard	8
Analysis	**14**	Methods for estimating or comparing measures of diagnostic accuracy	8
	**15**	How indeterminate index test or reference standard results were handled	10
	**16**	How missing data on the index test and reference standard were handled	10
	**17**	Any analyses of variability in diagnostic accuracy, distinguishing pre-specified from exploratory	29, 30, 31
	**18**	Intended sample size and how it was determined	8
**RESULTS**			
Participants	**19**	Flow of participants, using a diagram	24, 25
	**20**	Baseline demographic and clinical characteristics of participants	10, 20
	**21a**	Distribution of severity of disease in those with the target condition	10
	**21b**	Distribution of alternative diagnoses in those without the target condition	10
	**22**	Time interval and any clinical interventions between index test and reference standard	10
Test results	**23**	Cross tabulation of the index test results (or their distribution) by the results of the reference standard	21, 26, 27
	**24**	Estimates of diagnostic accuracy and their precision (such as 95% confidence intervals)	10, 11, 22
	**25**	Any adverse events from performing the index test or the reference standard	10
**DISCUSSION**			
	**26**	Study limitations, including sources of potential bias, statistical uncertainty, and generalisability	11, 12, 13
	**27**	Implications for practice, including the intended use and clinical role of the index test	13
**OTHER INFORMATION**			
	**28**	Registration number and name of registry	3
	**29**	Where the full study protocol can be accessed	6
	**30**	Sources of funding and other support; role of funders	18

For peer review only

STARD 2015

AIM

STARD stands for "Standards for Reporting Diagnostic accuracy studies". This list of items was developed to contribute to the completeness and transparency of reporting of diagnostic accuracy studies. Authors can use the list to write informative study reports. Editors and peer-reviewers can use it to evaluate whether the information has been included in manuscripts submitted for publication.

EXPLANATION

A **diagnostic accuracy study** evaluates the ability of one or more medical tests to correctly classify study participants as having a **target condition.** This can be a disease, a disease stage, response or benefit from therapy, or an event or condition in the future. A medical test can be an imaging procedure, a laboratory test, elements from history and physical examination, a combination of these, or any other method for collecting information about the current health status of a patient.

The test whose accuracy is evaluated is called **index test.** A study can evaluate the accuracy of one or more index tests. Evaluating the ability of a medical test to correctly classify patients is typically done by comparing the distribution of the index test results with those of the **reference standard**. The reference standard is the best available method for establishing the presence or absence of the target condition. An accuracy study can rely on one or more reference standards.

If test results are categorized as either positive or negative, the cross tabulation of the index test results against those of the reference standard can be used to estimate the **sensitivity** of the index test (the proportion of participants *with* the target condition who have a positive index test), and its **specificity** (the proportion *without* the target condition who have a negative index test). From this cross tabulation (sometimes referred to as the contingency or "2x2" table), several other accuracy statistics can be estimated, such as the positive and negative **predictive values** of the test. Confidence intervals around estimates of accuracy can then be calculated to quantify the statistical **precision** of the measurements.

If the index test results can take more than two values, categorization of test results as positive or negative requires a **test positivity cut-off**. When multiple such cut-offs can be defined, authors can report a receiver operating characteristic (ROC) curve which graphically represents the combination of sensitivity and specificity for each possible test positivity cut-off. The **area under the ROC curve** informs in a single numerical value about the overall diagnostic accuracy of the index test.

The **intended use** of a medical test can be diagnosis, screening, staging, monitoring, surveillance, prediction or prognosis. The **clinical role** of a test explains its position relative to existing tests in the clinical pathway. A replacement test, for example, replaces an existing test. A triage test is used before an existing test; an add-on test is used after an existing test.

Besides diagnostic accuracy, several other outcomes and statistics may be relevant in the evaluation of medical tests. Medical tests can also be used to classify patients for purposes other than diagnosis, such as staging or prognosis. The STARD list was not explicitly developed for these other outcomes, statistics, and study types, although most STARD items would still apply.

DEVELOPMENT

This STARD list was released in 2015. The 30 items were identified by an international expert group of methodologists, researchers, and editors. The guiding principle in the development of STARD was to select items that, when reported, would help readers to judge the potential for bias in the study, to appraise the applicability of the study findings and the validity of conclusions and recommendations. The list represents an update of the first version, which was published in 2003.

More information can be found on http://www.equator-network.org/reporting-guidelines/stard.

For peer review only - http://bmjopen.bmj.com/site/about/guidelines.xhtml

The diagnostic accuracy of hearing tests and cost-effectiveness of school entry hearing screening programmes

Final Version 4.0
30th September 2013

For peer review only

**Short title:**	School entry hearing screening programmes
**Acronym:**	SES
**Trial Registration:**	N/A
**ISRCTN:**	ISRCTN61668996
**NRES reference:**	12/WM/0195
**Trial Sponsor:**	University of Nottingham
**Sponsor reference:**	**12064**
**Funding Source:**	NIHR Health Technology Assessment Programme 10/63/03

Ethics/R&D/CLRN reference: 106333

This protocol is confidential and the property of the University of Nottingham. No part of it may be transmitted, reproduced, published, or used by others persons without prior written authorisation from the University of Nottingham.
For peer review only - http://bmjopen.bmj.com/site/about/guidelines.xhtml

STUDY PERSONNEL AND CONTACT DETAILS

Sponsor: University of Nottingham
Contact name Mr Paul Cartledge
Head of Research Grants and Contracts
Research and Graduate Services
King's Meadow Campus
Lenton Lane
Nottingham
NG7 2NR

Chief investigator: Dr Heather Fortnum
Associate Professor and Reader in Hearing Research
NHBRU
Ropewalk House
113 The Ropewalk
Nottingham
NG1 5DU
Phone: 0115 8223601
Fax: 0115 8223618
Email heather.fortnum@nottingham.ac.uk

Co-investigators: Professor Rod Taylor
Professor in Health Services Research
Peninsula Medical School
Universities of Exeter and Plymouth
Veysey Building
Salmon Pool Lane
Exeter
EX2 4SG
rod.taylor@pms.ac.uk

Dr Obi Ukoumunne
Senior lecturer in medical statistics
PenCLAHRC
Veysey Building
Salmon Pool Lane
Exeter
EX2 4SG
obioha.ukoumunne@pcmd.ac.uk

Professor Chris Hyde
Prof of public health and clinical epidemiology
Peninsula College of Medicine and Dentistry
University of Exeter
Veysey Building
Salmon Pool Lane
Exeter

This protocol is confidential and the property of the University of Nottingham. No part of it may be transmitted, reproduced, published, or used by others persons without prior written authorisation from the University of Nottingham

For peer review only - http://bmjopen.bmj.com/site/about/guidelines.xhtml

EX2 4SG
'c.j.hyde@exeter.ac.uk'

Claire Benton
Clinical Scientist (Audiology)
Nottingham Audiology Service
Ropewalk House
113 The Ropewalk
Nottingham
NG1 5DU
Claire.benton@nuh.nhs.uk

Joanne Moody
Audiological Scientist
Cambridge University Hospital Foundation Trust
Addenbrookes Hospital
Hills Road
Cambridge
CB2 0QQ
joanne.moody@addenbrookes.nhs.uk

Ann Allardice
Head of service - interim
Health Visiting and School Nursing
Nottingham East

Park House Health and Social Care Centre
61 Burton Road
Nottingham
NG4 3DQ
ann.allardice@nottshc-chp.nhs.uk

Parent representatives: Julian and Sarah Watson
julian@juleswatson.co.uk
Sarah.C.Watson@nottingham.ac.uk

Study Statistician: Dr Obi Ukoumunne
Senior lecturer in medical statistics
PenCLAHRC
Veysey Building
Salmon Pool Lane
Exeter
EX2 4SG
obioha.ukoumunne@pcmd.ac.uk
Tel: 01392 726070

For peer review only

This protocol is confidential and the property of the University of Nottingham. No part of it may be transmitted, reproduced, published, or used by others persons without prior written authorisation from the University of Nottingham
For peer review only - http://bmjopen.bmj.com/site/about/guidelines.xhtml

Study Researchers:
Ms Mara Ozolins, Research Fellow
Ms Sam Catterick, Research Associate
NHBRU
Ropewalk House
113 The Ropewalk
Nottingham
NG1 5DU
Phone: 0115 8232827 / 8232607
Fax: 0115 8232618
Email mara.ozolins@nottingham.ac.uk / samantha.catterick@nottingham.ac.uk

Study Coordinating Centre:
NHBRU
Ropewalk House
113 The Ropewalk
Nottingham
NG1 5DU

This protocol is confidential and the property of the University of Nottingham. No part of it may be transmitted, reproduced, published, or used by others persons without prior written authorisation from the University of Nottingham.
For peer review only - http://bmjopen.bmj.com/site/about/guidelines.xhtml
For peer review only

SYNOPSIS

Title	The diagnostic accuracy of hearing tests and cost-effectiveness of school entry hearing screening programmes
Acronym	SES
Short title	School entry hearing screening programmes
Chief Investigator	Dr Heather Fortnum
Objectives	The overarching aims of this project (comprising 5 sub-studies S1-S5, also known as SES1-SES5) are evaluation of the diagnostic accuracy of hearing screening tests and the cost-effectiveness of screening for hearing impairment at school entry.<br>The specific research objectives of this project are:<br>• To determine and compare the diagnostic accuracy of two methods for screening for the identification of sensorineural or permanent conductive hearing impairment at or around school entry.<br>• To develop an existing SES economic model and synthesise the findings of the research in order to provide robust estimates of key parameters in the economic model beyond accuracy. In particular the yield and nature of hearing loss detected in a system with no SES; the yield, impact and costs of screen positive individuals in an SES system; and the costs of setting up an SES system.
Study Configuration	Case control study; prospective and retrospective observational cohort studies, health economic analyses:<br>S1: case control comparison of two screening methods.<br>S2: retrospective and prospective observational cohort study (Cambridge; no school screening)<br>S3: prospective observational cohort study (Nottingham; school screen); questionnaire on impact of screen<br>S4: costs of a school screen<br>S5: Health economic analysis and modelling
Setting	Secondary care, community, schools and research facility (NHBRU),
Sample size estimate	*(Applicable to study 1)* Eighty (80) case children will be selected from a range of centres in order to estimate the sensitivity of the screening tests. This sample is large enough to estimate a sensitivity of 75% with a margin of error of +/- 10% (based on a 95% confidence interval). One hundred and sixty (160) control children, recruited from schools, is a large enough sample to estimate a specificity of 90% with a margin of error of +/- 5% The margin of error for the estimated sensitivity and specificity will provide plausible ranges of values within which to test the stability of the results from the economic model (Study 5) to our assumptions about screening accuracy.
Number of participants	S1: 80 case children (aged 4-6 years) with a sensorineural or permanent conductive hearing loss either bilaterally (average of 20 to 60dBHL) or unilaterally (any level >=20dBHL) identified from collaborating audiology services, and 160 control children (aged 4-6 years) with no identified hearing loss recruited through Nottinghamshire schools

For peer review only

This protocol is confidential and the property of the University of Nottingham. No part of it may be transmitted, reproduced, published, or used by others persons without prior written authorisation from the University of Nottingham.
For peer review only - http://bmjopen.bmj.com/site/about/guidelines.xhtml

	S2: Data collection over a period of 7 years for 600-700 children referred per year to 2nd tier services for investigation of possible hearing loss and 20-30 children per year referred from 2nd tier to 3rd tier audiology services in Cambridgeshire for further investigation of possible sensorineural hearing loss. Data appropriate to the analysis will be extracted.<br><br>S3: Data collection of all referrals aged 3 to 6 years and 364 days to Nottingham audiology service for 24 months, plus 3 months for outstanding follow-up, Responders to a questionnaire from around 200 children and their families referred from the school entry hearing screening programme to Nottingham Audiology Services in a period of 24 months, once individual follow-up is complete.<br><br>S4: At least 4 schools will be recruited to take part in the study. Each school will be visited on one or more days according to routine practice by one screener accompanied by a researcher. All children in the appropriate classes who have parental consent to take part will be screened using both technologies (N~180 children).<br><br>S5: Health economic analysis and modelling with no study subjects involved.
Eligibility criteria	S1: Inclusion: Cases<br>Children aged between 4 and 6 years, with a sensorineural or permanent conductive hearing loss either bilaterally (average of 20 to 60dBHL) or unilaterally (any level >=20dBHL) confirmed by gold standard pure tone audiometry under headphones in sound–proofed rooms, identified from the service records of collaborating paediatric audiology services.<br>Inclusion: Controls<br>Children aged between 4 and 6 years, with no known hearing loss, identified through local Nottinghamshire primary schools.<br><br>S2:<br>Data will be included if they relate to children referred to the Cambridge service between October 1st 2007 and August 31st 2014 (follow-ups to 30th November 2014), who were referred by any source other than the newborn hearing screen. Children for whom one or more data items are missing will not be excluded at the data collection stage, but such missing data will be accounted for in analyses.<br><br>S3: Inclusion: All children aged 3 to 6 years and 364 days attending the paediatric audiology service in Nottingham, having been referred, from 1st September 2012 through to 31st August 2014 (follow-ups to 30th November 2014).<br>Exclusion: Children already identified with a permanent loss or under active management with the audiology service and for whom records of audiological assessments exist.<br><br>S4: Children will be included in the screening process following protocols and guidelines for parental consent normally administered by the service.
Description of interventions	S1 and S4: Hearing screening performed on one occasion using two methods. (i) currently standard pure-tone sweep screening under headphones, (ii) Siemens hand held ear level HearCheck hearing screening device.<br>S1: Pure-tone audiometry under headphones to assess detailed hearing level on one occasion for controls, also cases who have not had one in last 12 months<br>S3: self-completion questionnaire to parents

This protocol is confidential and the property of the University of Nottingham. No part of it may be transmitted, reproduced, published, or used by others persons without prior written authorisation from the University of Nottingham

For peer review only - http://bmjopen.bmj.com/site/about/guidelines.xhtml

Duration of study	1st August 2012 for 30 months to 31st January 2015, participants involved on a single occasion
Randomisation and blinding	S1: Each child will undergo two screening tests and control children will also have a gold standard hearing assessment and we will aim, as far as possible within available resources, to blind those undertaking the assessments to the results of the other assessments. The order of the two screening assessments will be randomised, with the gold standard last.<br>S4: the order of the two screening tests will be randomised where possible, without preventing the standard PTS test from being carried out.
Outcome measures	**S1:** "pass" or "refer" for the screening tests as defined by the protocol compared with the result of the gold-standard PTA (normal or refer)<br><br>**S2:** The primary outcomes are yield (the incidence of newly identified hearing loss in children) and age at referral. Secondary outcomes will be the referral source, pathway of care, number and types of assessments, interventions received, level of hearing loss, cause of hearing loss (when available).<br><br>**S3:** The primary outcome measures will be the yield (from the school screen), age at referral, and the costs both to the service and the families of referral through to definitive identification of hearing loss or discharge from follow-up. Secondary outcomes will be the referral source, pathway of care, number and type of assessments, interventions received, level of hearing loss, cause of hearing loss (when available).<br><br>**S4:** The primary outcome is the mean cost per child of implementing each of the two test technologies. Costs will include the staff type, grade and time taken in conducting the test plus the cost of the equipment. Outcome (pass/fail) will be recorded). Feedback from the school nurses on which screening method they prefer and why will also be sought.<br><br>**S5:** Estimation of incremental cost per case detected, and cost to families of being referred.
Statistical methods	S1: Sensitivity will be estimated for each test as the proportion of the case children that test positive and specificity will be estimated as the proportion of the control children that test negative, precision quantified using 95% confidence intervals. McNemar's test will be used to compare each of sensitivity and specificity between the two tests, reporting p-values.<br><br>S2: Quantitative characteristics summarised using means and standard deviations (or medians and inter-quartile ranges) and categorical characteristics summarised using percentages. Precision summarised using 95% confidence intervals.<br>S3: Descriptive methods as per S2. Additionally, yield (proportion diagnosed) will be compared between a non-screened sample (S2) and a routinely screened sample (S3) with a test and confidence intervals. Referral ages will be compared between S2 and S3 using box and whisker plots and survival curves, adjusting where appropriate for sex and deprivation (postcode).<br><br>In order to make inferences (from the questionnaire), costs will be summarised as means. Given the likely skewed nature of these outcomes, we will use the non-parametric bias corrected accelerated bootstrapping method (2000 replications) to validate the confidence intervals for the means. We will seek to estimate mean costs for referrals according to their outcome i.e. true positives, false positives and false negatives.

This protocol is confidential and the property of the University of Nottingham. No part of it may be transmitted, reproduced, published, or used by others persons without prior written authorisation from the University of Nottingham

For peer review only - http://bmjopen.bmj.com/site/about/guidelines.xhtml

	S4: The mean cost of the two tests will be compared using the paired t-test. Again, the bootstrap method will be used to validate the confidence intervals for the mean difference.<br>S5: Methods will include cost-effectiveness planes and curves.

For peer review only

This protocol is confidential and the property of the University of Nottingham. No part of it may be transmitted, reproduced, published, or used by others persons without prior written authorisation from the University of Nottingham.
For peer review only - http://bmjopen.bmj.com/site/about/guidelines.xhtml

ABBREVIATIONS

AE	Adverse Event
CI	Chief Investigator overall
CRF	Case Report Form
DMC	Data Monitoring Committee
GCP	Good Clinical Practice
ICF	Informed Consent Form
NDCS	National Children's Deaf Society
NHBRU	Nottingham Hearing Biomedical Research Unit
NHS	National Health Service
NICE	National Institute for Health and Clinical Excellence
NIHR	National Institute for Health Research
PIS	Participant Information Sheet
PSC	Project Steering Committee
PTA	Pure Tone Audiometry
REC	Research Ethics Committee
R&D	Research and Development department
SES	School Entry Hearing Screening
SNHL	Sensorineural Hearing Loss
UNHS	Universal Newborn Hearing Screening

For peer review only

This protocol is confidential and the property of the University of Nottingham. No part of it may be transmitted, reproduced, published, or used by others persons without prior written authorisation from the University of Nottingham.
For peer review only - http://bmjopen.bmj.com/site/about/guidelines.xhtml

TABLE OF CONTENTS

SYNOPSIS 5

ABBREVIATIONS 9

STUDY BACKGROUND INFORMATION AND RATIONALE 12

STUDY OBJECTIVES AND PURPOSE 13

PURPOSE 13
PRIMARY OBJECTIVE 13
SECONDARY OBJECTIVES 13

STUDY DESIGN 13

STUDY CONFIGURATION 13
Primary endpoint 13
Secondary endpoint 13
Safety endpoints 14
Stopping rules and discontinuation 14
RANDOMISATION AND BLINDING 14
Maintenance of randomisation codes and procedures for breaking code 14
STUDY MANAGEMENT 14
DURATION OF THE STUDY AND PARTICIPANT INVOLVEMENT 15
End of the Study 15
SELECTION AND WITHDRAWAL OF PARTICIPANTS 151516
Recruitment 151516
Eligibility criteria 17
Inclusion criteria 17
Exclusion criteria 17
Expected duration of participant participation 17
Removal of participants from therapy or assessments 17
Informed consent 18

STUDY TREATMENT AND REGIMEN 18

Details of procedures for all the studies are contained in working documents for each study. 22
Compliance 22
Criteria for terminating trial 22

STATISTICS 222223

Methods 222223
Sample size and justification 232324
Assessment of efficacy 24
Assessment of safety 24
Procedures for missing, unused and spurious data 24
Definition of populations analysed 25

ADVERSE EVENTS 25

ETHICAL AND REGULATORY ASPECTS 242425

For peer review only

This protocol is confidential and the property of the University of Nottingham. No part of it may be transmitted, reproduced, published, or used by others persons without prior written authorisation from the University of Nottingham.

For peer review only - http://bmjopen.bmj.com/site/about/guidelines.xhtml

ETHICS COMMITTEE AND REGULATORY APPROVALS 25
INFORMED CONSENT AND PARTICIPANT INFORMATION 25
RECORDS 25
Case Report Forms 26
Sample Labelling 27
Source documents 27
Direct access to source data / documents 262627
DATA PROTECTION 27

QUALITY ASSURANCE & AUDIT **27**

INSURANCE AND INDEMNITY 27
PROJECT CONDUCT 27
PROJECT DATA 28
RECORD RETENTION AND ARCHIVING 28
DISCONTINUATION OF THE PROJECT BY THE SPONSOR 28
STATEMENT OF CONFIDENTIALITY 28

PUBLICATION AND DISSEMINATION POLICY **29**

USER AND PUBLIC INVOLVEMENT **29**

STUDY FINANCES **29**

Funding source 29
Participant stipends and payments 29

SIGNATURE PAGES **30**

REFERENCES **30**

For peer review only

This protocol is confidential and the property of the University of Nottingham. No part of it may be transmitted, reproduced, published, or used by others persons without prior written authorisation from the University of Nottingham.
For peer review only - http://bmjopen.bmj.com/site/about/guidelines.xhtml

STUDY BACKGROUND INFORMATION AND RATIONALE

Identification of permanent hearing loss at the earliest possible age is crucial to maximise the development of speech and language and contribute to the best opportunities for educational achievement and quality of life [8]. The introduction of the highly sensitive and specific universal newborn hearing screening (UNHS) has led to the identification of the vast majority of children born with a hearing loss who undergo the screen [4,6]. However, not all children who will ultimately have a hearing loss are identifiable at birth. Data published in 2001 [3] reported an adjusted prevalence of hearing loss at age 3 of 1.07 per 1000 and a prevalence for children aged 9-15 of 2.05 per 1000. Thus, due to acquisition, progression or late-onset of the hearing loss and/or geographical movement of families, there remain a significant number of children with a hearing loss to be identified after the newborn period. The incidence of hearing loss in children after the neonatal period can occur at any time which means there is no optimum time for a further universal hearing screen. Following the introduction of UNHS, the universal distraction hearing test undertaken by health visitors at around 8 months of age was abandoned based on a lack of robust implementation and a low yield of cases [2,5]. The school entry screen (SES), however, remained in place in many parts of the UK and is considered as a "back-stop" screen to identify children as part of a "captive population at school entry. Identification of hearing impairment in children in the time between the newborn period and school entry is achieved through parental and professional awareness and a close follow-up of children who pass the neonatal screen but who are considered to be at risk [7]

In order to best provide a service for the identification of permanent childhood hearing loss whilst making best use of scarce NHS resources it is important to gather robust evidence to support particular cost-effective implementations of service delivery at times relevant to the aetiology of hearing loss and the child's development. There is no question that screening for hearing impairment at birth is efficient and cost-effective [4] but questions remain about the value of any further universal screen. A previous HTA-commissioned study to evaluate the SES undertaken by a number of the co-applicants of this project (HF, RT) [1] reported a survey of current practice, longitudinal data on yield, a systematic review of effectiveness and a model of cost-effectiveness. It concluded that there was insufficient, good quality data on which to base a decision about the value of the SES following the introduction of UNHS. The study did however report longitudinal data from a single district in London which indicated a small but significant number of children with a hearing impairment that was first identified via the SES in that particular population [7], and national survey data which reported examples of children not identified by other methods, for example, those who had moved into the country or who were highly mobile within it. One of the recommendations of the report was the need for comparative trials to compare the effectiveness and cost-effectiveness of alternative approaches to screening for identification of hearing impairment in the post-newborn period.

This proposal develops the findings of the previous HTA report [1] by gathering empirical data to address the questions posed by the funding call and to contribute to policy decisions on the future of the School entry hearing screen. The research question asks whether there should be a screening programme to identify permanent hearing loss in children when they start primary school. It asks if the cost of such a screen is appropriate for the outcomes achieved i.e. the number of children identified by this method compared with a system with no screen which is responsive to parental or professional concern. In addition it asks for a comparison of 2 different ways of doing the screen. One is the standard pure-tone sweep test whereby children listen to tones at frequencies between 500 and 4,000 Hz at specific levels usually 20-35 dBHL. The second screen uses a hand-held instrument, held at the child's ear that emits tones at 1,000 and 3,000 Hz at 20-75dBHL. In both situations the child indicates when they have heard the tones, by raising a hand, pressing a response button or other activity.

The questions are very relevant as previous research has shown that the number of children identified by this screen around age 5 (the yield) is low following the introduction of hearing screening at birth for all babies, and the subsequent widespread further development of a system that is responsive to professional and parental concerns at any age [1, 7]. We will address these questions with a series of 4 empirical studies which will feed into and inform an economic model of cost-effectiveness.

This protocol is confidential and the property of the University of Nottingham. No part of it may be transmitted, reproduced, published, or used by others persons without prior written authorisation from the University of Nottingham
For peer review only - http://bmjopen.bmj.com/site/about/guidelines.xhtml

STUDY OBJECTIVES AND PURPOSE

PURPOSE

The overarching aims of this project are evaluation of the diagnostic accuracy of hearing screening tests and the cost-effectiveness of screening for hearing impairment at school entry.

PRIMARY OBJECTIVE

To determine and compare the diagnostic accuracy of two methods for screening for the identification of sensorineural and permanent conductive hearing impairment at or around school entry i.e. pure tone sweep audiometry across 4 frequencies and 1 level, and the HearCheck pure tone screen with 2 fixed frequencies and 3 levels (S1)

SECONDARY OBJECTIVES

For a service with a routinely applied school entry hearing screen and a service with no SES, to compare the yield, referral age and source through assessment to intervention for permanent childhood hearing impairment and to measure the costs of referrals.

- To evaluate the effectiveness and cost effectiveness of screening for hearing impairment relative to no implementation of a universal screen at school entry through an economic model
- To explore the impact for the child and the family of a positive result from a screen (both true and false positives) resulting in referral for further assessment.
- To determine the resource costs in implementing the two alternative screening methods in primary schools
- To develop an existing SES economic model and synthesise the findings of studies 1-4 in order to provide robust estimates of key parameters in the economic model beyond accuracy, and to determine the cost-effectiveness of SES. In particular the yield and nature of hearing loss detected in a system with no SES; the yield, impact and costs of screen positive individuals in an SES system; and the costs of setting up an SES system

STUDY DESIGN

STUDY CONFIGURATION

The project involves data collection from the audiology services in Cambridge and Nottingham and from children invited by collaborating audiology departments in England. It comprises 5 studies (S1-S5)

S1 – Case-control study of diagnostic accuracy

S2, S3 and S4 – Cohort studies including both retrospective (S2) and prospective (S2, S3, S4) data collection

S5 – Health economic analyses and modelling

Primary endpoint for project

Pass or fail of the hearing screen and confirmation of any hearing loss present. (Accuracy of the screening tests) (S1)

Secondary endpoints for project

Yield, age at referral (these are primary outcomes for S2 and S3), referral source, number and type of assessments, interventions received, level and cause of hearing loss (S2, S3).

Impact on family (S3)

Cost effectiveness, pass or fail of tests & user preference (S4, S5)

Safety endpoints

This protocol is confidential and the property of the University of Nottingham. No part of it may be transmitted, reproduced, published, or used by others persons without prior written authorisation from the University of Nottingham

For peer review only - http://bmjopen.bmj.com/site/about/guidelines.xhtml

There are no risks to the participants in this project and adverse events are unlikely. None of the sound levels to be used are of a damaging level.

Stopping rules and discontinuation

Participants are free to withdraw from the study at any time without giving a reason.

RANDOMISATION AND BLINDING

S1 - Each child will undergo two screening tests and a gold standard hearing assessment (PTA) and we will aim, as far as possible within available resources, to blind those undertaking the assessments to the results of the other assessments. The staff based in the CTU in Plymouth will produce a randomisation list on paper which the researchers will use to determine for each participant the order of the tests, which ear is first for each test, and which researcher does which test. For the cases researchers will do one test each according to the list. For the controls one researcher will do both screening tests and the other will do the PTA. The PTA will always be done last so as not to influence the screening tests. Both researchers based in Nottingham will see the child at home or at the research facility in Nottingham. The screening assessments are automated and hence the influence of the person undertaking the screen is minimal and we feel will not lead to major or systematic bias. The researchers will be trained in implementing the tests.

It is not possible to blind the researchers to the knowledge of whether or not a child in Study 1 has a hearing impairment. Any child living out of Nottingham or wearing a hearing aid or exhibiting any hearing difficulty will be likely to be a 'case'. Indeed, to ensure the screening tests are assessed appropriately it is important to be aware that the child has hearing difficulties so that the researchers can be sure all instructions concerning the test are heard and understood.

S4 – the order of the two hearing screening tests will be randomised. The nurse may override this if they think that the child is unlikely to complete both tests, in which case the standard test (PTS) will be performed first to enable the school health check to be completed.

S2, S3, and S5 – randomisation is not appropriate for the data collection methodologies.

Maintenance of randomisation codes and procedures for breaking code

Randomisation relates only to the order in which the tests are completed.

STUDY MANAGEMENT

The Chief Investigator has overall responsibility for the study and shall oversee all study management from her base in Nottingham (NIHR Nottingham Hearing Biomedical Research Unit). She will be responsible for day to day management of the project working with the two researchers appointed.

The project steering committee (PSC) will:

- provide overall supervision for the project on behalf of the Sponsor and Funder and to ensure that the project is conducted to the rigorous standards set out in the Medical Research Council's (MRC) Guidelines for Good Clinical Practice;
- provide advice, through its chair, to the Chief Investigator(s), the Sponsor, the Funder, the Host Institution and the Contractor on all appropriate aspects of the trial;
- concentrate on progress of the trial, adherence to the protocol, patient safety and the consideration of new information of relevance to the research question;
- ensure appropriate ethics and other approvals are obtained in line with the project plan;
- agree substantial protocol amendments and provide advice to the investigators on all aspects of the trial.

This protocol is confidential and the property of the University of Nottingham. No part of it may be transmitted, reproduced, published, or used by others persons without prior written authorisation from the University of Nottingham.
For peer review only - http://bmjopen.bmj.com/site/about/guidelines.xhtml

Membership of the committee will comprise:

- an independent Chair from a different institution to members of the research team.
- two independent audiologists with relevant expertise
- two individuals to independently represent expertise in statistics, epidemiology and diagnostics.
- the Chief Investigator (HF) and the study statistician (RT or OU) (neither will have voting rights, and they will be excluded from closed sessions of the PSC where data are discussed).
- at least one individual who is able to contribute a patient and/or wider public perspective.
- a representative of the sponsor and a representative from the research network as observers.

The committee will meet twice a year for the duration of the project

For this particular project the role of a separate data monitoring committee is not straightforward. The project comprises a series of observational studies. It is unlikely with only 80 cases that any interim analyses would be appropriate and it is unlikely that any adverse events will occur. We propose that the PSC described above would monitor data quality and adverse events. Given the nature of research questions being addressed in this programme of work, interim data analyses are not required for safety or efficacy.

The data custodian will be the Chief Investigator.

DURATION OF THE STUDY AND PARTICIPANT INVOLVEMENT

The project started on 1st August 2012 and is due to complete on 31st January 2015 (30 months).

S1: each child will be involved on one occasion for screening tests and pure-tone audiometry (if appropriate). There will be no follow-up visits. Recruitment will begin in October 2012, or as soon as possible after then, and assessment will be complete by 31st August 2014. Non responses will be followed up by one reminder letter.

S2, S3: data collected from records by the audiologist. If the audiologists are short of time, the researchers may help with this which would mean they see the patient files in Cambridge/Nottingham or would need access to the hospital system.

S3: parents of children who fail a routine screening test (performed outside of the study) and who are referred to the audiology service will be sent a questionnaire for self-completion. Non-response will be followed up with one reminder. Questionnaires will be sent for all children referred to the service between November 2012 and September 2014, once their final outcome is known. If recipients of the questionnaire agree, their answers may be followed up by a telephone interview.

S4: children will be screened in their usual school using two methods. If the result of the standard screen is negative (fail) or unclear, the child will be re-screened and referred onward as appropriate according to usual practice. If a child does not pass the new Hearcheck method, the researchers will make a note of it as evaluation of the equipment and children will not be retested. The nurses will not record this information as the screen is not currently a validated method to test children's hearing. For this parental notification will not be necessary. Testing will take place in schools between September 2013 and July 2014 inclusive.

S5: no patient involvement

End of the Study

The end of the project will be when the last of the data has been entered into the model.

SELECTION AND WITHDRAWAL OF PARTICIPANTS

Recruitment

This protocol is confidential and the property of the University of Nottingham. No part of it may be transmitted, reproduced, published, or used by others persons without prior written authorisation from the University of Nottingham
For peer review only - http://bmjopen.bmj.com/site/about/guidelines.xhtml

Data will be gathered in the community (family homes), in secondary care (NHS audiology services), the research facility (NHBRU) and in primary schools.

The project concerns the identification of hearing loss in children between the ages of 4 and 6 years. Participants therefore are required to be such children.
S1: “Case” children as those aged 4-6 years at time of screening identified from collaborating audiology services in England as having a sensorineural or permanent conductive hearing loss. They will be identified by local services and we will ask a senior paediatric audiologist to contact families on behalf of the research team and to send them an invitation to take part in the study with a summary information sheet plus a separate pictorial information sheet for children. Once a parent has expressed interest in thier child taking part by returning a reply slip to the researchers, we will send them a full detailed information sheet containing all aspects pertaining to the study. This will enable parents to have another chance to consider taking part, before they come to an appointment. We have agreed commitment from the services in Nottingham, Sheffield, Leicester, Chesterfield, Derby, Mansfield and Lincoln. We will also approach audiology services in other areas as needed to reach recruitment targets.Some audiologists may choose to create a list once only, including some younger children (from age 2) who will reach the required age range within the time period of the study. Those wishing to take part, but who are too young at the time of invitation will be sent a holding letter. If the response rate to our invitation letters is low, we will post a reminder on websites of regional branches of the National Deaf Children’s Society (NDCS). This may give some parents a second opportunity to consent to their children participating. In addition we will ask audiologists from the collaborating centres to send out reminder letters to parents who have not responded to the initial invitation letters, which will give them a second opportunity to take part.

The children in the control group will be recruited from the reception and year 1 population of schools in Nottinghamshire by a letter of invitation either sent to parents at the time of routine school screening, or to all parents of a class of children at another time to be agreed with the school. Letters will be sent by the school.

S2 and S3: Data will be accessed by the member of the research team with clinical responsibility for the audiology service in Cambridge (JM) and Nottingham (CB). Data will be transferred to other members of the research team for analyses, identified by a study number, with hospital number removed. The researchers (conducting, but not analysing the study) may need to see the patient files in Cambridge/Nottingham, or PAS in order to help the audiologists with the data collection,
S3: the parents of children who have been referred following a failed school screening test will be sent questionnaires by the local paediatric audiology service on behalf of the research team. If they wish to complete the questionnaire they will be asked to return it directly to the research team. The questionnaire will give parents the option to take part in a follow-up telephone interview. Contact details will be provided voluntarily on a separate sheet to the main questionnaire so they can be destroyed later.

The letter of invitation for S1 and S3 will include a statement in locally common languages offering to translate the participant information sheets, and consent forms into these languages.

S4: It is routine practice for children to have their hearing checked at school in the term in which they become 5years old. Some will be tested in reception year and some in year 1. We will follow the routine practice of each participating school in notifying parents that this will be happening. Parents will be sent a letter from the research team. They can opt out of the research by returning the reply slip on our invitation letter to the school nurses (in an envelope provided).

For all studies it will be explained to the potential participant that entry into the project is entirely voluntary and that their treatment and care will not be affected by their decision. It will also be explained that they can withdraw at any time but attempts will be made to avoid this occurrence. In the event of their withdrawal it will be explained that their data collected so far cannot be erased and we will use the data in the final analyses where appropriate.

Eligibility criteria

This protocol is confidential and the property of the University of Nottingham. No part of it may be transmitted, reproduced, published, or used by others persons without prior written authorisation from the University of Nottingham.
For peer review only - http://bmjopen.bmj.com/site/about/guidelines.xhtml

Consent from parents will be sought for the children involved in study 1 and the opportunity to opt-out will be given for study 4. Any of the control children in study 1 and any of the children in study 4 could potentially have a hearing loss to be identified. The case children in study 1 will be known to have a hearing loss and their involvement in the study will provide no further information to contribute to their individual clinical management. The tests are easy to perform and relatively quick and will result in no side effects.

Inclusion criteria

S1: “Case” children as those aged 4-6 years at time of testing, identified from collaborating audiology services in England as having a sensorineural or permanent conductive hearing loss, averaged across 0.5, 1, 2, and 4kHz, either bilaterally (average of 20 - 60dBHL) or unilaterally (any level ≥20 dBHL). “Control” children will be aged between 4 and 6 years, with no known hearing loss, identified through local Nottinghamshire primary schools.

S2: Data will be included if they relate to children (between the ages of 3 years and 6 years 364 days, though data on all ages are collected by the service) referred to the Cambridge service between October 1st 2007 and 31st August 2014, who were referred by any source other than the newborn hearing screen. Children for whom one or more data items are missing will not be excluded at the data collection stage, but such missing data will be accounted for in analyses.

S3: Data related to all children aged 3 to 6 years and 364 days attending the paediatric audiology service in Nottingham, having been referred from 1st September 2012 through to 31st August 2014.

S4: Children will be included in the screening process in the term in which they reach the age of 5 years following protocols and guidelines for parental consent normally administered by the service.

Exclusion criteria

S1:

- Families identified by the audiology services will not be invited to take part if the responsible audiologist feels it would be inappropriate or cause added unnecessary burden e.g. seriously or terminally ill family member.
- Children of families who do not agree to take part.
- Case children who have no record of a PTA in the last 12 months and who are unwilling to travel to their local service or to Nottingham.
- Children who are unwell such that their illness would affect the results of the tests (unable to carry out the test)

S2: Children referred as a result of the newborn hearing screen

S3: Children already identified with a permanent loss or under active management with the audiology service and for whom records of audiological assessments exist.

S4: children for whom we receive an opt out reply slip.

Expected duration of participant participation

Study participants will be participating in the study for a single occasion of assessment.

Removal of participants from therapy or assessments

Participants may be withdrawn from the study either at their own request or at the discretion of the Investigator. The participants will be made aware that this will not affect their future care. Participants will be made aware (via the information sheet and consent form) that should they withdraw the data collected to date cannot be erased and may still be used in the final analysis.

Informed consent

S1: Consent from the parent or legal guardian will be sought for all participants. The Informed Consent Form will be signed and dated by the parent or legal guardian before the child enters the study. For case children, the local audiologist will post a summary information sheet to the parent or legal guardian, ensuring that the parent or legal guardian has sufficient time to consider whether the child should participate or not. For control children, the summary information sheet will be posted home

This protocol is confidential and the property of the University of Nottingham. No part of it may be transmitted, reproduced, published, or used by others persons without prior written authorisation from the University of Nottingham
For peer review only - http://bmjopen.bmj.com/site/about/guidelines.xhtml

from school with the invitation letters. The researchers will answer any questions that the parent or legal guardian has concerning study participation. Once a parent returns the reply slip expressing interest in their child taking part, we will send a full detailed information sheet for them to have another opportunity to decide whether to take part.
We shall also provide an age appropriate Participant Information Sheet for each child. This will take the form of a pictorial description of what will happen in the study that can be shared with the child and explained by the parent or legal guardian

Informed consent will be collected by the researcher from the parent or legal guardian of each child before they undergo any interventions related to the study. One copy of this will be kept by the parent or legal guardian, one will be kept by the researcher, and, for case children, a third will be retained in the child's hospital records.

Should there be any subsequent amendment to the final protocol, which might affect a participant's participation in the trial, continuing consent will be obtained using an amended Consent form which will be signed by the participant.

S3: consent to participate in the study will be implied by return of a completed questionnaire. For those returning the questionnaire, consent will also be sought to explore responses by telephone interview.

S4: Consent will not be sought from the parent of legal guardian for all children undergoing screening for hearing loss at school as we shall follow the routine practice of the school nurse teams which involves giving parents a chance to opt out of screening instead. We are not collecting identifiable information on the participants during this study.

STUDY TREATMENT AND REGIMEN

Participants will not receive any treatment. The intervention being assessed is the methodology to screen for hearing loss in children at the age at which they start school. As such there is no schedule of treatments

Each child who participates in studies 1 or 4 will be seen on one occasion with no individual, personal follow up. As such it is not appropriate to include a schematic diagram of trial design, procedures and stages, specifying the points of randomisation, baseline & intermediate visits, interim analyses, final visit + any follow-up contact/monitoring.

Issues of concomitant treatments are not applicable to this project.

To address the objectives this project consists of five studies, S1 to S5.

S1: A case-control study to look at which of 2 different implementations of the screen is more accurate in correctly identifying children with and without a hearing loss i.e. to assess the diagnostic accuracy. Both alternatives are implementations of using a pure tone listening task to screen for permanent childhood hearing loss. We will compare a traditional pure tone sweep across 4 frequencies (.5, 1, 2 and 4 kHz) at a level of 20 dBHL, with the HearCheck (Siemens) pure tone screener at 2 fixed frequencies: 1 kHz, with levels 55, 35 and 20 dBHL, & 3 kHz with levels 75, 55 and 35 dBHL).

80 case children (4-6 years) with a sensorineural or permanent conductive hearing loss either bilaterally (average 20 to 60dBHL) or unilaterally (any level >=20dBHL) identified from collaborating audiology services, and 160 control children with no identified hearing loss recruited through Nottinghamshire schools will undergo hearing screens with both implementations and the results compared with gold-standard pure-tone audiometry.

The "case" children will be identified by local services and we will ask a senior paediatric audiologist to contact families on behalf of the research team and to send them an invitation to take part in the study. Families who agree to take part will be invited to undergo the two screening tests either in their own homes or in the research facilities at NHBRU in Nottingham City Centre depending on their preference. In either location the screening tests will be performed to an identical protocol, by

This protocol is confidential and the property of the University of Nottingham. No part of it may be transmitted, reproduced, published, or used by others persons without prior written authorisation from the University of Nottingham
For peer review only - http://bmjopen.bmj.com/site/about/guidelines.xhtml

researchers trained in the procedures, in rooms that are quiet but not soundproofed. A reminder letter may be sent to some of the families who have not responded.

The children in the control group will be recruited from the reception and year 1 children (aged 4-6) through schools in Nottinghamshire by a letter of invitation either sent to parents at the time of routine school screening, or to all parents of a class of children at another time to be agreed with the school. Families who agree to take part will be invited to undergo the two screening tests in the research facilities at NHBRU in Nottingham City. The screening tests will be performed by researchers in the research team in rooms that are quiet but not sound-proofed. The children in the control group will need to attend a facility where PTA can be measured and hence the option to have the screening test at home is not possible. Thus, all control children will undergo full pure tone audiometry under headphones in sound proofed booths either in the Nottingham audiology service or in NHBRU. As for cases, the representativeness of the control group is important to obtaining as unbiased assessment of specificity as possible. The results obtained by testing the children stated will be generalisable to the population of children with "normal‟ hearing defined at an average of ≤20 dBHL and the group will contain a typical spectrum of educational levels and socio-economic deprivation in order to ensure that controls are not limited to children in whom the tests are likely to be easiest to conduct

Based on diagnostic accuracy data from a previous UK study of SES, this sample size is large enough to estimate a sensitivity of 75% with a margin of error of +/- 10%, and a specificity of 90% with precision of 5%.

What will happen for case children?
Case children will be tested on one occasion with the two versions of the screening test. The order of the tests will be randomised between children and each test will be undertaken by one of two researchers working together. These tests will be carried out in the child's home, or the research facility on Nottingham (NHBRU) subject to parent preference. If the child has not had a pure tone audiogram recorded in the past 12 months or scheduled in the following three months we will ask the parent and child to attend the research facility to record this gold-standard measure against which we will assess the two screening tests. If an audiogram has been recorded in the past 12 months or is scheduled in the following three months we will ask the local audiologist to provide those data to us, via a secure route.

What will happen for control children?
Control children will receive the same two screening tests but will require a recording of a pure tone audiogram (done after the screening tests). All tests for control children will be undertaken at the research facility in Nottingham (NHBRU). If the child does not pass the PTA (on one occasion) they will be referred to the audiology service: failure means a threshold of 30 dBHL in either ear (air conduction) at any frequency. Failure of the screening tests alone will not result in referral. The CRF contains a referral form.

The pure-tone sweep screening method.
The pure tone sweep methodology for screening is implemented in many different configurations throughout the country. The most commonly used frequencies are the four we will use (1, 2, 4 and 0.5 kHz) which assess hearing at speech frequencies and include 4 kHz as an assessment of high frequency hearing. Each child listens to tones through headphones and indicates (e.g. by pressing a response button) when they hear a tone. The tones are altered in frequency and delivered at specific levels to determine the lowest level at which the child can hear. Each ear is tested separately. We will use a "pass" level of 20 dBHL to enable direct comparison with the HearCheck screen for each child, but a 30 dBHL level for referral. For each frequency and each ear a pass will be hearing at least 2 out of 3 of the repeated tones (no need to present a third time if the tone is heard twice).
The HearCheck screening method
For the HearCheck the child is presented with 6 tones in total; 3 at 1 kHz at 55, 35 and 20 dBHL and 3 at 3 kHz at 75, 55 and 35 dBHL. Tones are delivered via a hand held machine held next to the child's ear using a disposable cardboard ear cover. The machine automatically generates tones at 2 frequencies and 3 levels. The child indicates when they have heard the tone. Each ear is tested separately. We are aiming to identify hearing loss of greater than 20dBHL, either bilaterally or

This protocol is confidential and the property of the University of Nottingham. No part of it may be transmitted, reproduced, published, or used by others persons without prior written authorisation from the University of Nottingham
For peer review only - http://bmjopen.bmj.com/site/about/guidelines.xhtml

unilaterally and hence a “fail” criteria for the HearCheck will be anything less than 6 tones heard for each ear.

Detailed procedures for each of the screening tests will be contained in working documents. This will improve standardisation and off-set to some extent the possibility of bias arising from not being able to blind the clinical staff administering the tests from whether the child has hearing loss or not.

Gold standard pure tone audiometry

We will assess the diagnostic accuracy of the two methods of screening assessed against the gold standard of pure-tone audiometry (PTA) under headphones in a sound proofed or sound attenuated room, without hearing aids for hearing impaired children. Tones are presented to the child at discrete frequencies (250 Hz, 500Hz, 1 kHz, 2 kHz, 4 kHz, 8 kHz) and levels variable by 5dBHL. The child indicates when they have heard the sound (e.g. by moving a coloured ball onto the stand). . This method will be carried out according to the BSA recommended procedures (see http://www.thebsa.org.uk/docs/Guidelines/BSA_RP_PTA_FINAL_24Sept11.pdf), without otoscopic exam or masking, air conduction only.

It will not be possible in all cases for the researchers assessing the children with the screening tests to be blind to the child’s hearing status. Any child living out of Nottingham or wearing a hearing aid or exhibiting any hearing difficulty will likely be in the case group. In fact, to ensure the screening tests are assessed appropriately it is important to be aware that the child has hearing difficulties so that the researchers can be sure all instructions concerning the test are heard and understood. The results of the tests will be recorded anonymously and entered into analytical software with no indication of the child’s hearing status or location and hence an element of blinding will be associated with the analyses.

Not all invited families will agree to participate. For the control children this is unlikely to be a problem and we will be able to continue to invite families from local schools until the required number have been tested. For the cases we anticipate that approximately 120 children will meet the criteria in the services who have agreed to collaborate (Nottingham, Mansfield, Chesterfield, Sheffield, Leicester, Derby and Lincoln). We shall require 70% of them to agree to take part to achieve a recruitment of 80 children. If this is not achieved we shall post a reminder on the NDCS websites, and also ask the audiologists to send one reminder letter to the parents. Then we will extend the invitation to collaborating centres outside the East Midlands.,. Children will only need to attend on one occasion so there should be no loss to follow-up.

S2: Retrospective and prospective cohort study of children referred, from Oct 2007, for hearing assessment to the 2nd and 3rd tier audiology service in Cambridgeshire which has had no formal hearing screen at school entry since 1997. We will measure the number of children referred, their age, referral source, type and level of loss and resource use.

We will assess the service for the area of Cambridge City, and South and East Cambridgeshire which has offered no formal hearing screen at school entry since 1997. In collaboration with the 2nd and 3rd tier audiology service we will analyse retrospective data for children referred to the service from whatever source excluding newborn hearing screening, providing they are geographically within the target areas for the period from 1st October 2007 (when records are available) to 31st August 2012 and prospective data for children referred, from 1st September 2012 to 31st August 2014. The data to be collected will include the child’s date of birth, date of referral, the source of the referral, the number and type of assessments and interventions, and the level and probable cause of any hearing loss, postcode, staff grade and time and equipment and interventions associated with the subsequent management and service delivery to children correctly identified as having a hearing loss (true positives) and those incorrectly identified as having a hearing loss (false positives). This healthcare utilisation will be collected on all referrals. Data on children aged 3 to 6 years and 364 days will be included in the analysis. Rates will be calculated from routine data for the population and ages at risk. Data will be collected in Cambridge by Miss Moody, a member of the research team with clinical responsibility for this population of children.

We will compare these data with the Nottingham and Nottinghamshire area which has an existing SES service (see Study 3 for details)

This protocol is confidential and the property of the University of Nottingham. No part of it may be transmitted, reproduced, published, or used by others persons without prior written authorisation from the University of Nottingham
For peer review only - http://bmjopen.bmj.com/site/about/guidelines.xhtml

There are no planned interventions in this study; it is an analysis of cohort data.

S3: Prospective analysis of the costs of management, including impact on families, following referral from the screen for children referred to Nottingham Audiology Service from the SES for two years (N ~200). Measures of the resource costs of referral through to definitive identification of hearing loss or discharge, including questionnaire measures of impact for families of true and false positive cases will contribute to the economic modelling. All children will have either been definitively identified as having a hearing loss or will have been discharged with no follow-up by 30th November 2014. We will address the issue of false negatives through a review of the literature on the impact of delayed identification.

Data collection:
To assess the resource implications for the tertiary audiology service of referrals from the School Entry Hearing Screen we shall collect data prospectively for 24 months of referrals aged 3 to 6 years 364 days for the paediatric audiology service based in Nottingham. This service provides for children referred from schools in Nottingham and Nottinghamshire and has a total caseload of approximately 100 referrals from the SES annually. We will collect staff grade and time and equipment and interventions associated with the subsequent management and service delivery to children correctly identified as having a hearing loss (true positives) and those incorrectly identified as having a hearing loss (false positives). This healthcare utilisation will be collected on all referrals. In order to translate resource use into monetary values, unit costs will be applied according to staff type and grade based on local or national costs and prices. These staff costs include indirect overheads (the costs of support services such as human resources, finance, and estates needed to carry out the service's main functions). Equipment and consumables will be costed using manufacturers‟ list prices and amortised over a 5-year lifetime. This element of the project is service evaluation and as such does not require REC approval and we will not be producing participant information sheets for approval by REC.

The data collected will also include the child's date of birth, date of referral, the source of the referral, the number and type of assessments and interventions, and the level and probable cause of their hearing loss, plus postcode; the same data will be collected as in Cambridge (S2) plus the date of the school screen.

Questionnaire:
To explore the resource implications and impact on families of referral from the School Entry Hearing Screen we shall undertake a questionnaire study of all families of children who are referred for further assessment following the SES. Some of these children will be true positives, i.e. they will have a confirmed permanent hearing loss, and many more will be false positive, i.e. identified by the screen but found to have no permanent hearing loss. The questionnaire will be distributed to relevant families by Ms Benton, a member of the research team with clinical responsibility for this population of children. It will seek details on the amount of time, travel, cost, inconvenience, and anxiety experienced by families in undergoing the screen follow up process. Families will be sent the questionnaire by post by the audiology service. Questionnaires will be coded and the codes related to personal details held only by the responsible clinician. This will ensure only those not responding are sent reminders. Non-responders will be sent one reminder. As part of the questionnaire, parents can consent to further questions about the referral process in a telephone interview; they will need to provide contact details to the researchers for this (on a separate sheet).

Delayed identification in children who pass the screen but who do have a hearing loss (false negatives) is a further consequence of concern, but is not one we believe can be dealt with as part of the data we propose to collect. We will address this via a review of the literature, looking specifically for robust reports of data exploring the impact on the family and on the child's development and education, of an unidentified or late identified hearing loss.

Interventions for the children referred from the SES will be the routine clinical interventions applied to investigate the child's possible hearing loss, including audiometry, tympanometry etc. as appropriate and decided by the audiologist concerned with the child's care. There will be no contact

This protocol is confidential and the property of the University of Nottingham. No part of it may be transmitted, reproduced, published, or used by others persons without prior written authorisation from the University of Nottingham
For peer review only - http://bmjopen.bmj.com/site/about/guidelines.xhtml

with the research team. Families recruited to take part in the questionnaire part of the study will be asked to complete paper questionnaires.

S4: Determination of resource use in proposed physical setting, and practicalities of implementation, of two forms of screening test in schools within Nottinghamshire.

Study 4 will allow us to collect data on the practical application of the two screening methods. In collaboration with the Health Visiting and School service for NHS-County Health Partnership we propose to implement both screens in a number of schools throughout the county representing a range of catchment populations. We shall involve a number of personnel over all three terms of the school year (September 2013 to July 2014) to ensure a range of measurements consistent with differing staff methods of implementation, differing child characteristics at different times (maturity and seasonal infections i.e. colds) and different screening conditions in schools. We shall measure the time taken to implement the screens in school situations following current guidelines, the pass/fail results of the tests and gather opinions on use of the tests from the school nurses. We shall collect routinely available data on the costs for the service as a whole, including staff, buildings, equipment, administration and travel. These costs should not vary with the screen being implemented in the schools.

Routinely, children are identified by schools as eligible to undergo the hearing screen. Their parents are informed of this routine service and notified by letter, with the right to opt out. We propose to send an additional letter at the same time informing parents of the research study, again providing them with a chance to return an opt out reply, for their child to undergo a second screen with the HearCheck device at the same time as the routine screen. Those children for whom their parent has consented will undergo both tests. The order of the tests will be randomised where the school nurse feels it will be appropriate to do so. The school nurses will perform the tests and the researchers will be present as observers to collect data on the time taken to carry out the hearing screens, pass/fail results of the two tests and any feedback provided by the school nurses.

S5: Cost data will be collected for resource use in screening and follow-up assessment, or assessment following referral, including staff time, travel, equipment and facilities for all studies. These data will contribute to the development of a model of cost-effectiveness together with data derived from the literature and existing NHS data sources. All data will be collated, checked, cleaned and analysed by Peninsula Clinical Trials Unit.

Details of procedures for all the studies are contained in working documents for each study.

Compliance

No long-term follow-up. Compliance applies to one visit only

Criteria for terminating trial

As a diagnostic accuracy study through which all participant children will undergo hearing screening with both methods and a pure-tone audiogram, there should be no reason for stopping the trial as a whole.

If during an assessment session a particular child becomes upset or uncomfortable he/she will be withdrawn from the study and arrangements made, if necessary, to formally test his or her hearing at a later date.

STATISTICS

Methods

Statistical analyses of projects S1-S4 and the decision analytic modelling in project S5 will be led by a team of three methodologists experienced in quantitative analysis and decision analytic modelling (RT,

This protocol is confidential and the property of the University of Nottingham. No part of it may be transmitted, reproduced, published, or used by others persons without prior written authorisation from the University of Nottingham
For peer review only - http://bmjopen.bmj.com/site/about/guidelines.xhtml

OU and CH) based at the Peninsula Medical School at the University of Exeter. A variety of software packages appropriate to the data being analysed will be used including Stata. Analytic methods and sample size rationale for each study is detailed below, and further details will be given in separate analysis plans for each of these studies.

Given the nature of research questions being addressed in this programme of work, interim data analyses are not required for safety or efficacy.

S1: Sensitivity will be estimated for each test as the proportion of the case children that test positive and specificity will be estimated as the proportion of the control children that test negative. The precision of these estimates will be quantified using 95% confidence intervals. Because this is a "paired design" in which two diagnostic tests are evaluated on the same sample, McNemar's test will be used to compare each of sensitivity and specificity between the two tests, reporting p-values. Confidence intervals for the difference in sensitivity/specificity (percentages) between the tests will be constructed.

S2: Quantitative characteristics of the Cambridge cohort will be summarised using means and standard deviations (or medians and inter-quartile ranges) and categorical characteristics will be summarised using percentages. The precision of the estimated mean yield and referral age will be summarised using 95% confidence intervals.

S3: The yield (number of referrals with a hearing loss divided by number of children) and mean referral age in the non-screened sample (S2) will be compared to the yield and mean referral age from populations that routinely screen at school entry (4-5 years) (S3) using box and whisker plots and survival curves, quantifying the extent to which yield may differ and diagnosis of hearing loss may occur sooner or later in non-screened populations. Analyses may be adjusted for potential differences in population characteristics of the two cohorts including sex, and deprivation (based on postcode).
In order to make inferences from the questionnaire, costs and impact outcomes will be summarised as means. Given the likely skewed nature of these outcomes, we will use the non-parametric bias corrected accelerated bootstrapping method (2000 replications) to validate the confidence intervals for the means. We will seek to estimate mean costs for referrals according to their outcome i.e., true positives, false positives and false negatives.

The main outcomes are the yield, the age of referral and referral source. The study will provide key data to estimate important parameters for the economic modelling in Study 5 (by describing the cost experience for true positives and false positives).

S4: The mean costs of the two tests will be compared using the paired t-test. Again, the bootstrap method will be used to validate the confidence interval of the mean difference.

S5: Results from the modelling of cost-effectiveness will be presented in a disaggregated format (outcomes, resource use, costs), and also in the form of a cost-effectiveness ratio. Results will include estimation of incremental cost per case detected, Where appropriate, results will include presentation of cost-effectiveness consistent with the reference case used by NICE. Where probabilistic modelling is undertaken, probabilistic sensitivity analysis will be presented. Results will include presentation of cost-effectiveness planes, and cost-effectiveness acceptability curves. Univariate and probabilistic multivariate sensitivity analyses will be conducted, to explore structural and parameter estimates of greatest concern, such as test accuracy. This will include consideration of bias associated with use of a case-control methodology in study 1. This would use estimates of relative diagnostic odds ratio obtained from empirical investigations of the effects of study design features on test accuracy to deflate the observed accuracy to that which might have been expected if a less biased estimate from a traditional test accuracy study was available.

Sample size and justification

S1: Eighty (80) case children will be selected from collaborating centres in order to estimate the sensitivity of the screening tests. This sample is large enough to estimate a sensitivity of 75% with a margin of error of +/- 10% (based on a 95% confidence interval) and 160 controls is a large enough

This protocol is confidential and the property of the University of Nottingham. No part of it may be transmitted, reproduced, published, or used by others persons without prior written authorisation from the University of Nottingham
For peer review only - http://bmjopen.bmj.com/site/about/guidelines.xhtml

sample to estimate a specificity of 90% with a margin of error of +/- 5% The margin of error for the estimated sensitivity and specificity will provide plausible ranges of values within which to test the sensitivity of the results from the economic model (Study 5) to our assumptions about screening accuracy.

S2: Data collection over a period of 7 years for 600-700 (ie 4200-4900 in total) children referred per year to 2nd tier services for investigation of possible hearing loss and 20-30 children per year (140-210) referred from 2nd tier to 3rd tier audiology services in Cambridgeshire for further investigation of possible sensorineural or permanent conductive hearing loss. Of those 20-30, 5-10 may be confirmed to have a SNHL.

Assuming the standard deviation of the age of referral is approximately 0.5 years (6 months), the 40 subjects we might recruit is a large enough number to estimate the mean age of referral with a margin of error of +/- 0.16 years (1.9 months) based on 95% confidence intervals.

S3: We expect 200 children to be referred from the SES to Nottingham Audiology Services in a period of 24 months. For the questionnaire survey we will look to invite all referred families to complete a questionnaire to provide a spread of experience and opinion. This study has not been formally powered on statistical inference. We are confident, however, that a sufficient number of those referred will answer the questionnaire to allow us to address the S3 research questions.

S4: We intend to recruit at least 4 schools to take part in the study through contact with the Head teacher through the Health Visiting and School Nursing Service of NHS-County Health Partnership. Each school will be visited on one or more days, according to routine practice by one screener and the researcher. All children in the appropriate classes who have parental consent to take part will be screened using both technologies. We anticipate this will provide a convenience sample of about 180 children. This study has not been formally powered on statistical inference. We are confident, however, that N=180 will allow us to address S4 research questions.

Assessment of efficacy
Not applicable.

Assessment of safety
Not applicable

Procedures for missing, unused and spurious data
Rigorous data collection and checking procedures will be put in place to minimise the amount of missing data and to minimise the collection and processing of erroneous data. For each project, the amount of missing data will be assessed and appropriate imputation methods, where appropriate, will be employed.

Definition of populations analysed
Various populations involved in this project are defined above.

ADVERSE EVENTS

The intervention to be assessed as part of the project protocol is a hand held device which emits tones to a maximum of 75 dBHL. All other assessments are part of routine service and will not be adapted in any way. None of the assessments will increase the hearing loss in any child who is hearing impaired nor will they trigger a hearing loss in a hearing child, when carried out according to protocol. Equipment will be checked each day before use. We do not foresee any adverse events, as defined, occurring in this project. If any serious or related adverse events do occur we will follow the University of Nottingham Standard Operating Procedures on reporting of adverse events; serious events will be reported to the Chief Investigator within 24 hours and, if related to the procedure, to the ethics committee, steering committee and sponsor within 7 days.

ETHICAL AND REGULATORY ASPECTS

This protocol is confidential and the property of the University of Nottingham. No part of it may be transmitted, reproduced, published, or used by others persons without prior written authorisation from the University of Nottingham
For peer review only - http://bmjopen.bmj.com/site/about/guidelines.xhtml

When testing children in their own homes in study 1 the University of Nottingham Lone working policy will be adhered to. Two researchers will be involved in all visits.

ETHICS COMMITTEE AND REGULATORY APPROVALS

The project will not be initiated before the protocol, informed consent forms and participant information sheets have received approval / favourable opinion from the Research Ethics Committee (REC), and the respective National Health Service (NHS) Research & Development (R&D) department. Should a protocol amendment be made that requires REC approval, the changes in the protocol will not be instituted until the amendment and revised informed consent forms and participant information sheets have been reviewed and received approval / favourable opinion from the REC and R&D departments. A protocol amendment intended to eliminate an apparent immediate hazard to participants may be implemented immediately providing that the REC are notified as soon as possible and an approval is requested. Minor protocol amendments only for logistical or administrative changes may be implemented immediately; and the REC will be informed.

The project will be conducted in accordance with the ethical principles that have their origin in the Declaration of Helsinki, 1996; the principles of Good Clinical Practice, and the Department of Health Research Governance Framework for Health and Social care, 2005.

INFORMED CONSENT AND PARTICIPANT INFORMATION

S1: The process for obtaining participant informed assent and parent / guardian informed consent will be in accordance with the REC guidance, and Good Clinical Practice (GCP) and any other regulatory requirements that might be introduced. The investigator or their nominee and the parent or legal guardian of the participant shall both sign and date the Informed Consent Form before the person can participate in the study – this is usually at the study visit, having previously received full information about the study.

The parent or legal guardian of the participant will receive a copy of the signed and dated forms and the original will be retained in the Project Master File. A second copy will be filed in the participant's audiological notes and a signed and dated note made in the notes that informed consent was obtained for the project (case children only).

The decision regarding participation in the study is entirely voluntary. The investigator or their nominee shall emphasize to them that consent regarding the child's participation in the project may be withdrawn at any time without penalty or affecting the quality or quantity of the child's future medical care, or loss of benefits to which the child and his/her family is otherwise entitled. No project-specific interventions will be done before informed consent has been obtained.

The investigator will inform the parent or legal guardian of the participant of any relevant information that becomes available during the course of the study, and will discuss with them, whether they wish to continue with the study. If applicable they will be asked to sign revised consent forms.

If the Informed Consent Form is amended during the study, the investigator shall follow all applicable regulatory requirements pertaining to approval of the amended Informed Consent Form by the REC and use of the amended form (including for ongoing participants).

S4: Parents will be provided with the opportunity to opt out of the research screen, which follows current practice for school hearing tests in the schools we shall be observing.

RECORDS

Case Report Forms

A case report form (CRF) will be generated for each child participating in the project or for whom data are collected from records. CRFs will be developed by the project researchers in liaison with the staff of PenCTU collaborating on the project.

Each form will be completed by the researchers involved in the study or by members of the research team. Each participant will be assigned a project identity code number for use on CRFs, other project

For peer review only

This protocol is confidential and the property of the University of Nottingham. No part of it may be transmitted, reproduced, published, or used by others persons without prior written authorisation from the University of Nottingham.
For peer review only - http://bmjopen.bmj.com/site/about/guidelines.xhtml

documents and the electronic database. The documents and the database will also contain the date of birth (dd/mm/yyyy) and postcode.

For study 1, generated data from the results of the screening tests and pure-tone audiometry will be collected by the researchers and entered onto CRFs using the identity codes described above. A separate password protected electronic database will be used to maintain links between personal information and these codes for the resolution of queries.

For studies 2 and 3, data will be collected from audiology records in Cambridge and Nottingham by members of the research team with clinical responsibility for the participants (JM and CB). Identity codes will be used as described above. Personal data linked to these codes will be held by the responsible clinician in a password protected database for the purposes of query resolution. Data will be anonymised before transfer for analyses to other members of the research team. If clinician time is short, the Nottingham-based researchers may help the clinicians to collect data which will mean the data are not anonymous at the point of collection. Data will also be collected using questionnaires; these data will be anonymous, except where parents opt for further questions by telephone, in which case they will be asked to provide contact details on a separate sheet.

For study 4, the data to be collected are practical or nurse opinion, i.e. times, costs and preferences, and will not identify individual participants.

Paper copies of CRFs will be stored in locked filing cabinets in offices that are locked when unoccupied in the NIHR Nottingham Hearing Biomedical Research Unit. All electronic databases will be password protected, accessible only by members of the research team. Data transferred to collaborators in Plymouth and Exeter will identify participants only by study number and will be encrypted before transfer.

CRFs will be treated as confidential documents and held securely in accordance with regulations. Members of the research team with clinical responsibility and the investigator will make a separate confidential record of the participant's name, date of birth, local hospital number or NHS number, and Project identity code number (the Project Recruitment Log), to permit identification of all participants enrolled in the project, in accordance with regulatory requirements and for follow-up as required.
CRFs shall be restricted to those personnel approved by the Chief or local Principal Investigator and recorded on the 'Project Delegation Log.'

All paper forms shall be filled in using black ballpoint pen. Errors shall be lined out but not obliterated by using correction fluid and the correction inserted, initialled and dated.
The Chief or local Principal Investigator shall sign a declaration ensuring accuracy of data recorded in the CRF.

Sample Labelling
Each participant will be assigned an identity code number for use on the consent forms and other study documents and the electronic database. The documents and database will also use date of birth (dd/mm/yyyy) and postcode.

Source documents
Consent forms, data extracted from current medical records, screening and hearing results and completed questionnaires are considered as source documents and shall be filed at the investigator's site. Each CRF will also completely serve as its own source data. Only project staff as listed on the Delegation Log shall have access to project documentation other than the regulatory requirements listed below.

Direct access to source data / documents
The CRF and all source documents, including progress notes and copies of medical test results shall made be available at all times for review by the Chief Investigator, Sponsor's designee and inspection by relevant regulatory authorities.

This protocol is confidential and the property of the University of Nottingham. No part of it may be transmitted, reproduced, published, or used by others persons without prior written authorisation from the University of Nottingham.
For peer review only - http://bmjopen.bmj.com/site/about/guidelines.xhtml

DATA PROTECTION

All project staff and investigators will endeavour to protect the rights of the project's participants to privacy and informed consent, and will adhere to the Data Protection Act, 1998. The CRF will only collect the minimum required information for the purposes of the project. CRFs will be held securely, in a locked room, and in a locked cupboard or cabinet. Access to the information will be limited to the project staff and investigators and relevant regulatory authorities (see above). Computer held data including the trial database will be held securely and password protected. All data will be stored on a secure dedicated web server. Access will be restricted by user identifiers and passwords (encrypted using a one way encryption method).
Information about the project in the participant's medical records / hospital notes will be treated confidentially in the same way as all other confidential medical information.

Electronic data will be backed up every 24 hours to both local and remote media in encrypted format.

QUALITY ASSURANCE & AUDIT

INSURANCE AND INDEMNITY

Insurance and indemnity for project participants and project staff is covered within the NHS Indemnity Arrangements for clinical negligence claims in the NHS, issued under cover of HSG (96)48. There are no special compensation arrangements, but trial participants may have recourse through the NHS complaints procedures.

The University of Nottingham as research Sponsor indemnifies its staff, research participants and research protocols with both public liability insurance and clinical trials insurance. These policies include provision for indemnity in the event of a successful litigious claim for proven non-negligent harm.

PROJECT CONDUCT

Project conduct will be subject to systems audit of the Project Master File for inclusion of essential documents; permissions to conduct the project; Project Delegation Log; CVs of project staff and training received; local document control procedures; consent procedures and recruitment logs; adherence to procedures defined in the protocol (e.g. inclusion / exclusion criteria, correct randomisation, timeliness of visits); adverse event recording and reporting; accountability of trial materials and equipment calibration logs.

The Project Coordinator, or where required, a nominated designee of the Sponsor, shall carry out a site systems audit at least yearly and an audit report shall be made to the Project Steering Committee.

PROJECT DATA

Monitoring of project data shall include confirmation of informed consent; source data verification; data storage and data transfer procedures; local quality control checks and procedures, back-up and disaster recovery of any local databases and validation of data manipulation. The Project Coordinator, or where required, a nominated designee of the Sponsor, shall carry out monitoring of project data as an ongoing activity.

Entries on CRFs will be verified by inspection against the source data. A sample of CRFs (10% or as per the study risk assessment) will be checked on a regular basis for verification of all entries made. In addition the subsequent capture of the data on the trial database will be checked. Where corrections are required these will carry a full audit trail and justification.

Project data and evidence of monitoring and systems audits will be made available for inspection by REC as required.

This protocol is confidential and the property of the University of Nottingham. No part of it may be transmitted, reproduced, published, or used by others persons without prior written authorisation from the University of Nottingham
For peer review only - http://bmjopen.bmj.com/site/about/guidelines.xhtml

See separate documents SES Monitoring and Audit Summary, and Summary and Procedures documents for each study.

RECORD RETENTION AND ARCHIVING

In compliance with the ICH/GCP guidelines, regulations and in accordance with the University of Nottingham Research Code of Conduct and Research Ethics, the Chief or local Principal Investigator will maintain all records and documents regarding the conduct of the study. These will be retained for at least 7 years or for longer if required. If the responsible investigator is no longer able to maintain the study records, a second person will be nominated to take over this responsibility.

The Project Master File and project documents held by the Chief Investigator on behalf of the Sponsor shall be finally archived at secure archive facilities at the University of Nottingham. This archive shall include all project databases and associated meta-data encryption codes.

DISCONTINUATION OF THE PROJECT BY THE SPONSOR

The Sponsor reserves the right to discontinue this project at any time for failure to meet expected enrolment goals, for safety or any other administrative reasons. The Sponsor shall take advice from the Project Steering Committee as appropriate in making this decision.

STATEMENT OF CONFIDENTIALITY

Individual participant medical information obtained as a result of this study is considered confidential and disclosure to third parties is prohibited with the exceptions noted above.
Participant confidentiality will be further ensured by utilising identification code numbers to correspond to treatment data in the computer files.

Such medical information may be given to the participant’s medical team and all appropriate medical personnel responsible for the participant’s welfare. If information is disclosed during the study that could pose a risk of harm to the participant or others, the researcher will discuss this with the CI and where appropriate report accordingly.

Data generated as a result of this project will be available for inspection on request by the participating physicians, the University of Nottingham representatives, the REC, local R&D Departments and the regulatory authorities.

PUBLICATION AND DISSEMINATION POLICY

The findings of the project will be disseminated via a report to the funder expected to be published as an HTA monograph in 2015-6. Additional publications will be submitted to peer reviewed journals during 2014-15. Oral and poster presentations will be submitted from 2013 through 2015 to national and international conferences with audiences of audiologists, paediatricians, otorhinolaryngologists, speech and language therapists and teachers. Participants will not be identified in any publications. Parents of children involved in Study 1 will be offered the opportunity to receive a short summary of the findings at the end of the study.

USER AND PUBLIC INVOLVEMENT

We have parents of a hearing impaired child in Nottingham as part of the research team (funded). The individuals will be fully involved, attending all research team meetings and will make specific contributions to the design of approaches and literature to families, design and content of questionnaires, and to contribute the patient perspective to all dissemination. We have a project steering group comprising external experts to advise on and oversee the programme of research. This group includes further lay representation in the form of representation from the National Deaf Children’s Society.

For peer review only

This protocol is confidential and the property of the University of Nottingham. No part of it may be transmitted, reproduced, published, or used by others persons without prior written authorisation from the University of Nottingham.
For peer review only - http://bmjopen.bmj.com/site/about/guidelines.xhtml

STUDY FINANCES

Funding source

This study is funded by the National Institute of Health Research Health Technology Assessment programme (NIHR HTA) reference 10/63/03.

Participant stipends and payments

Schools who agree to participate in study 1 will be entered into a prize draw to win £100. Participants in study 1 will be offered payment for their travel expenses to the research, as per standard NHBRU rates and procedures. In addition the funder has agreed to fund an allowance of £10* each for the 240 children taking part in Study 1 as compensation for their time and inconvenience. We propose to pay this in the form of a book token. *As of 1st Oct 2013 in line with a protocol amendment, the book token amount for the remaining children will be increased to £20.

Parents who complete and return a questionnaire to us for study 3 will be entered into a prize draw to win a £50 voucher of their choice.

For peer review only

This protocol is confidential and the property of the University of Nottingham. No part of it may be transmitted, reproduced, published, or used by others persons without prior written authorisation from the University of Nottingham.

For peer review only - http://bmjopen.bmj.com/site/about/guidelines.xhtml

SIGNATURE PAGES

Signatories to Protocol:

Chief Investigator: (name)__Heather Fortnum__

Signature:____ 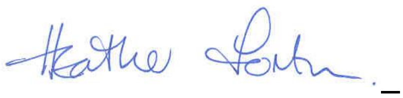 _

Date: 21 Dec 2012_

Co- investigator: (name) ________________________

Signature:__________________________________

Date: ___________

Trial Statistician: (name)

Signature:__________________________________

Date: ___________

REFERENCES

References

[1] Bamford J, Fortnum H, Bristow K, et al. Current practice, accuracy, effectiveness and cost-effectiveness of the school entry hearing screen. Health Technol Assess 2007;11(32)

[2] Bamford J, Uus K, Davis A. Screening for hearing loss in childhood: issues, evidence and current approaches in the UK. J Med Screen. 2005;12(3):119-24.

[3] Fortnum H, Summerfield Q, Marshall D, et al. Prevalence of permanent childhood hearing impairment in the United Kingdom and implications for universal neonatal hearing screening: questionnaire based ascertainment study. Br Med J, 2001;323:536-539

[4] Kennedy C, McCann D, Campbell MJ, Kimm L, Thornton R. Universal newborn screening for permanent childhood hearing impairment: an 8-year follow-up of a controlled trial. Lancet. 2005 Aug 20-26;366(9486):660-2.

[5] Russ SA, Poulakis Z, Wake M, Barker M, Rickards F, Jarman FC, Saunders K, Edwards G, Symons L, Oberklaid F The distraction test: the last word? J Paediatr Child Health. 2005 Apr;41(4):197-200

[6] van Dommelen P, van Straaten H, Verkerk P; the Dutch NICU Neonatal Hearing Screening

For peer review only

This protocol is confidential and the property of the University of Nottingham. No part of it may be transmitted, reproduced, published, or used by others persons without prior written authorisation from the University of Nottingham.

For peer review only - http://bmjopen.bmj.com/site/about/guidelines.xhtml

Working Group. Ten-year quality assurance of the nationwide hearing screening programme in Dutch neonatal intensive care units; Acta Paediatr. 2011 Feb 22. doi: 10.1111/j.1651-2227.2011.02230.x. [Epub ahead of print]
[7] Watkin PM, Baldwin M. Identifying deafness in early childhood: requirements after the newborn hearing screen. Arch Dis Child 2011; 96: 62-66
[8] Yoshinago-Itano C, Coulter D, Thomson V. The Colorado newborn hearing screening project: effects on speech and language development for children with hearing loss. J Perinatol 2000;20:S132-7

For peer review only

This protocol is confidential and the property of the University of Nottingham. No part of it may be transmitted, reproduced, published, or used by others persons without prior written authorisation from the University of Nottingham
For peer review only - http://bmjopen.bmj.com/site/about/guidelines.xhtml

HEALTH TECHNOLOGY ASSESSMENT

VOLUME 20 ISSUE 36 MAY 2016
ISSN 1366-5278

CrossMark click for updates

A programme of studies including assessment of diagnostic accuracy of school hearing screening tests and a cost-effectiveness model of school entry hearing screening programmes

Heather Fortnum, Obioha C Ukoumunne, Chris Hyde, Rod S Taylor, Mara Ozolins, Sam Errington, Zhivko Zhelev, Clive Pritchard, Claire Benton, Joanne Moody, Laura Cocking, Julian Watson and Sarah Roberts

NHS
National Institute for Health Research

DOI 10.3310/hta20360

For peer review only

For peer review only

A programme of studies including assessment of diagnostic accuracy of school hearing screening tests and a cost-effectiveness model of school entry hearing screening programmes

Heather Fortnum,[1] Obioha C Ukoumunne,[2] Chris Hyde,[3]* Rod S Taylor,[3] Mara Ozolins,[1] Sam Errington,[1] Zhivko Zhelev,[2] Clive Pritchard,[4] Claire Benton,[5] Joanne Moody,[6] Laura Cocking,[7] Julian Watson[8] and Sarah Roberts[4]

[1]National Institute for Health Research, Nottingham Hearing Biomedical Research Unit, Hearing and Otology Group, Division of Clinical Neuroscience, School of Medicine, University of Nottingham, Nottingham, UK
[2]National Institute for Health Research, Collaborations for Leadership in Applied Health Research and Care South West Peninsula, University of Exeter Medical School, Exeter, UK
[3]Institute of Health Research, University of Exeter Medical School, Exeter, UK
[4]Optimity Advisors, Kemp House, London, UK
[5]Nottingham Audiology Services, Nottingham University Hospitals, Nottingham, UK
[6]Cambridgeshire Community Services, Community Child Health, Ida Darwin Hospital, Fulbourn, Cambridge, UK
[7]Peninsula Clinical Trials Unit, Plymouth University Peninsula Schools of Medicine and Dentistry, Plymouth, UK
[8]Parent representative, Nottingham, UK

*Corresponding author

Declared competing interests of authors: Dr Fortnum and Professor Taylor were co-authors on the previous *Health Technology Assessment* (HTA) publication reporting evaluation of the school entry hearing screen [Bamford J, Fortnum H, Bristow K, Smith J, Vamvakas G, Davies L, et al. Current practice, accuracy, effectiveness and cost-effectiveness of the school entry hearing screen. *Health Technol Assess* 2007;**11**(32)]. Professor Taylor is chairperson of the National Institute for Health Research (NIHR) Health Services and Delivery Research researcher-led panel, March 2014–February 2016 (appointment extended to February 2018), and a member from 2013. He is also a member of NIHR Priority Research Advisory Methodology Group (PRAMG), August 2015–present, is a core member of NIHR HTA Themed Call Board, 2012–present and is a member of the core group of methodological experts for the NIHR Programme Grants for Applied Research programme, 2013–present.

Published May 2016
DOI: 10.3310/hta20360

For peer review only

For peer review only

For peer review only

This report should be referenced as follows:

Fortnum H, Ukoumunne OC, Hyde C, Taylor RS, Ozolins M, Errington S, *et al*. A programme of studies including assessment of diagnostic accuracy of school hearing screening tests and a cost-effectiveness model of school entry hearing screening programmes. *Health Technol Assess* 2016;**20**(36).

Health Technology Assessment is indexed and abstracted in *Index Medicus*/MEDLINE, *Excerpta Medica*/EMBASE, *Science Citation Index Expanded* (SciSearch®) and *Current Contents*®/ Clinical Medicine.

For peer review only

Health Technology Assessment

HTA/HTA TAR

ISSN 1366-5278 (Print)

ISSN 2046-4924 (Online)

Impact factor: 5.027

Health Technology Assessment is indexed in MEDLINE, CINAHL, EMBASE, The Cochrane Library and the ISI Science Citation Index.

This journal is a member of and subscribes to the principles of the Committee on Publication Ethics (COPE) (www.publicationethics.org/).

Editorial contact: nihredit@southampton.ac.uk

The full HTA archive is freely available to view online at www.journalslibrary.nihr.ac.uk/hta. Print-on-demand copies can be purchased from the report pages of the NIHR Journals Library website: www.journalslibrary.nihr.ac.uk

Criteria for inclusion in the *Health Technology Assessment* journal

Reports are published in *Health Technology Assessment* (HTA) if (1) they have resulted from work for the HTA programme, and (2) they are of a sufficiently high scientific quality as assessed by the reviewers and editors.

Reviews in *Health Technology Assessment* are termed 'systematic' when the account of the search appraisal and synthesis methods (to minimise biases and random errors) would, in theory, permit the replication of the review by others.

HTA programme

The HTA programme, part of the National Institute for Health Research (NIHR), was set up in 1993. It produces high-quality research information on the effectiveness, costs and broader impact of health technologies for those who use, manage and provide care in the NHS. 'Health technologies' are broadly defined as all interventions used to promote health, prevent and treat disease, and improve rehabilitation and long-term care.

The journal is indexed in NHS Evidence via its abstracts included in MEDLINE and its Technology Assessment Reports inform National Institute for Health and Care Excellence (NICE) guidance. HTA research is also an important source of evidence for National Screening Committee (NSC) policy decisions.

For more information about the HTA programme please visit the website: http://www.nets.nihr.ac.uk/programmes/hta

This report

The research reported in this issue of the journal was funded by the HTA programme as project number 10/63/03. The contractual start date was in August 2012. The draft report began editorial review in March 2015 and was accepted for publication in November 2015. The authors have been wholly responsible for all data collection, analysis and interpretation, and for writing up their work. The HTA editors and publisher have tried to ensure the accuracy of the authors' report and would like to thank the reviewers for their constructive comments on the draft document. However, they do not accept liability for damages or losses arising from material published in this report.

This report presents independent research funded by the National Institute for Health Research (NIHR). The views and opinions expressed by authors in this publication are those of the authors and do not necessarily reflect those of the NHS, the NIHR, NETSCC, the HTA programme or the Department of Health. If there are verbatim quotations included in this publication the views and opinions expressed by the interviewees are those of the interviewees and do not necessarily reflect those of the authors, those of the NHS, the NIHR, NETSCC, the HTA programme or the Department of Health.

© Queen's Printer and Controller of HMSO 2016. This work was produced by Fortnum *et al.* under the terms of a commissioning contract issued by the Secretary of State for Health. This issue may be freely reproduced for the purposes of private research and study and extracts (or indeed, the full report) may be included in professional journals provided that suitable acknowledgement is made and the reproduction is not associated with any form of advertising. Applications for commercial reproduction should be addressed to: NIHR Journals Library, National Institute for Health Research, Evaluation, Trials and Studies Coordinating Centre, Alpha House, University of Southampton Science Park, Southampton SO16 7NS, UK.

Published by the NIHR Journals Library (www.journalslibrary.nihr.ac.uk), produced by Prepress Projects Ltd, Perth, Scotland (www.prepress-projects.co.uk).

For peer review only

Health Technology Assessment Editor-in-Chief

Professor Hywel Williams Director, HTA Programme, UK and Foundation Professor and Co-Director of the Centre of Evidence-Based Dermatology, University of Nottingham, UK

NIHR Journals Library Editor-in-Chief

Professor Tom Walley Director, NIHR Evaluation, Trials and Studies and Director of the HTA Programme, UK

NIHR Journals Library Editors

Professor Ken Stein Chair of HTA Editorial Board and Professor of Public Health, University of Exeter Medical School, UK

Professor Andree Le May Chair of NIHR Journals Library Editorial Group (EME, HS&DR, PGfAR, PHR journals)

Dr Martin Ashton-Key Consultant in Public Health Medicine/Consultant Advisor, NETSCC, UK

Professor Matthias Beck Chair in Public Sector Management and Subject Leader (Management Group), Queen's University Management School, Queen's University Belfast, UK

Professor Aileen Clarke Professor of Public Health and Health Services Research, Warwick Medical School, University of Warwick, UK

Dr Tessa Crilly Director, Crystal Blue Consulting Ltd, UK

Dr Peter Davidson Director of NETSCC, HTA, UK

Ms Tara Lamont Scientific Advisor, NETSCC, UK

Professor Elaine McColl Director, Newcastle Clinical Trials Unit, Institute of Health and Society, Newcastle University, UK

Professor William McGuire Professor of Child Health, Hull York Medical School, University of York, UK

Professor Geoffrey Meads Professor of Health Sciences Research, Health and Wellbeing Research and Development Group, University of Winchester, UK

Professor John Norrie Health Services Research Unit, University of Aberdeen, UK

Professor John Powell Consultant Clinical Adviser, National Institute for Health and Care Excellence (NICE), UK

Professor James Raftery Professor of Health Technology Assessment, Wessex Institute, Faculty of Medicine, University of Southampton, UK

Dr Rob Riemsma Reviews Manager, Kleijnen Systematic Reviews Ltd, UK

Professor Helen Roberts Professor of Child Health Research, UCL Institute of Child Health, UK

Professor Jonathan Ross Professor of Sexual Health and HIV, University Hospital Birmingham, UK

Professor Helen Snooks Professor of Health Services Research, Institute of Life Science, College of Medicine, Swansea University, UK

Professor Jim Thornton Professor of Obstetrics and Gynaecology, Faculty of Medicine and Health Sciences, University of Nottingham, UK

Please visit the website for a list of members of the NIHR Journals Library Board: www.journalslibrary.nihr.ac.uk/about/editors

Editorial contact: nihredit@southampton.ac.uk

For peer review only

DOI: 10.3310/hta20360 HEALTH TECHNOLOGY ASSESSMENT 2016 VOL. 20 NO. 36

Abstract

A programme of studies including assessment of diagnostic accuracy of school hearing screening tests and a cost-effectiveness model of school entry hearing screening programmes

Heather Fortnum,[1] Obioha C Ukoumunne,[2] Chris Hyde,[3]* Rod S Taylor,[3] Mara Ozolins,[1] Sam Errington,[1] Zhivko Zhelev,[2] Clive Pritchard,[4] Claire Benton,[5] Joanne Moody,[6] Laura Cocking,[7] Julian Watson[8] and Sarah Roberts[4]

[1]National Institute for Health Research, Nottingham Hearing Biomedical Research Unit, Hearing and Otology Group, Division of Clinical Neuroscience, School of Medicine, University of Nottingham, Nottingham, UK
[2]National Institute for Health Research, Collaborations for Leadership in Applied Health Research and Care South West Peninsula, University of Exeter Medical School, Exeter, UK
[3]Institute of Health Research, University of Exeter Medical School, Exeter, UK
[4]Optimity Advisors, Kemp House, London, UK
[5]Nottingham Audiology Services, Nottingham University Hospitals, Nottingham, UK
[6]Cambridgeshire Community Services, Community Child Health, Ida Darwin Hospital, Fulbourn, Cambridge, UK
[7]Peninsula Clinical Trials Unit, Plymouth University Peninsula Schools of Medicine and Dentistry, Plymouth, UK
[8]Parent representative, Nottingham, UK

*Corresponding author C.J.Hyde@exeter.ac.uk

For peer review only

Background: Identification of permanent hearing impairment at the earliest possible age is crucial to maximise the development of speech and language. Universal newborn hearing screening identifies the majority of the 1 in 1000 children born with a hearing impairment, but later onset can occur at any time and there is no optimum time for further screening. A universal but non-standardised school entry screening (SES) programme is in place in many parts of the UK but its value is questioned.

Objectives: To evaluate the diagnostic accuracy of hearing screening tests and the cost-effectiveness of the SES programme in the UK.

Design: Systematic review, case–control diagnostic accuracy study, comparison of routinely collected data for services with and without a SES programme, parental questionnaires, observation of practical implementation and cost-effectiveness modelling.

Setting: Second- and third-tier audiology services; community.

Participants: Children aged 4–6 years and their parents.

Main outcome measures: Diagnostic accuracy of two hearing screening devices, referral rate and source, yield, age at referral and cost per quality-adjusted life-year.

© Queen's Printer and Controller of HMSO 2016. This work was produced by Fortnum *et al.* under the terms of a commissioning contract issued by the Secretary of State for Health. This issue may be freely reproduced for the purposes of private research and study and extracts (or indeed, the full report) may be included in professional journals provided that suitable acknowledgement is made and the reproduction is not associated with any form of advertising. Applications for commercial reproduction should be addressed to: NIHR Journals Library, National Institute for Health Research, Evaluation, Trials and Studies Coordinating Centre, Alpha House, University of Southampton Science Park, Southampton SO16 7NS, UK.

For peer review only - http://bmjopen.bmj.com/site/about/guidelines.xhtml

ABSTRACT

Results: The review of diagnostic accuracy studies concluded that research to date demonstrates marked variability in the design, methodological quality and results. The pure-tone screen (PTS) (Amplivox, Eynsham, UK) and HearCheck (HC) screener (Siemens, Frimley, UK) devices had high sensitivity (PTS $\geq$ 89%, HC $\geq$ 83%) and specificity (PTS $\geq$ 78%, HC $\geq$ 83%) for identifying hearing impairment. The rate of referral for hearing problems was 36% lower with SES (Nottingham) relative to no SES (Cambridge) [rate ratio 0.64, 95% confidence interval (CI) 0.59 to 0.69; $p < 0.001$]. The yield of confirmed cases did not differ between areas with and without SES (rate ratio 0.82, 95% CI 0.63 to 1.06; $p = 0.12$). The mean age of referral did not differ between areas with and without SES for all referrals but children with confirmed hearing impairment were older at referral in the site with SES (mean age difference 0.47 years, 95% CI 0.24 to 0.70 years; $p < 0.001$). Parental responses revealed that the consequences to the family of the referral process are minor. A SES programme is unlikely to be cost-effective and, using base-case assumptions, is dominated by a no screening strategy. A SES programme could be cost-effective if there are fewer referrals associated with SES programmes or if referrals occur more quickly with SES programmes.

Conclusions: A SES programme using the PTS or HC screener is unlikely to be effective in increasing the identified number of cases with hearing impairment and lowering the average age at identification and is therefore unlikely to represent good value for money. This finding is, however, critically dependent on the results of the observational study comparing Nottingham and Cambridge, which has limitations. The following are suggested: systematic reviews of the accuracy of devices used to measure hearing at school entry; characterisation and measurement of the cost-effectiveness of different approaches to the ad-hoc referral system; examination of programme specificity as opposed to test specificity; further observational comparative studies of different programmes; and opportunistic trials of withdrawal of SES programmes.

Trial registration: Current Controlled Trials ISRCTN61668996.

Funding: This project was funded by the NIHR Health Technology Assessment programme and will be published in full in *Health Technology Assessment*; Vol. 20, No. 36. See the NIHR Journals Library website for further project information.

For peer review only

For peer review only - http://bmjopen.bmj.com/site/about/guidelines.xhtml

DOI: 10.3310/hta20360 HEALTH TECHNOLOGY ASSESSMENT 2016 VOL. 20 NO. 36

Contents

List of tables xiii

List of figures xvii

List of abbreviations xix

Plain English summary xxi

Scientific summary xxiii

Chapter 1 Background and main questions **1**
Childhood hearing impairment and screening 1
Previous Health Technology Assessment study 3
Assessment of cost-effectiveness 3
Study aims and objectives 4
Structure of the project and the report 4
Patient and public involvement 6

Chapter 2 Update of the diagnostic accuracy systematic review **7**
Introduction 7
Objectives 7
Methods 7
Search strategy 7
Inclusion/exclusion criteria 8
Selection of studies and data extraction 8
Assessment of the methodological quality 9
Statistical analysis and data synthesis 9
Results 9
Search results and selection of studies 9
Characteristics of included studies 10
Methodological quality of included studies 15
Test accuracy 15
Discussion 22
Summary of the findings from the 2007 Health Technology Assessment report 22
Combining the results from the 2007 Health Technology Assessment report and the current update 23
Conclusion 25

Chapter 3 Diagnostic accuracy of the pure-tone screen and HearCheck screener for identifying hearing impairment in school children **27**
Introduction 27
Objectives 27
Methods 28
Design 28
Recruitment 28
Assessment 29
Screening tests 30
Data collection 31

© Queen's Printer and Controller of HMSO 2016. This work was produced by Fortnum *et al.* under the terms of a commissioning contract issued by the Secretary of State for Health. This issue may be freely reproduced for the purposes of private research and study and extracts (or indeed, the full report) may be included in professional journals provided that suitable acknowledgement is made and the reproduction is not associated with any form of advertising. Applications for commercial reproduction should be addressed to: NIHR Journals Library, National Institute for Health Research, Evaluation, Trials and Studies Coordinating Centre, Alpha House, University of Southampton Science Park, Southampton SO16 7NS, UK.

For peer review only - http://bmjopen.bmj.com/site/about/guidelines.xhtml

For peer review only

CONTENTS

Sample size calculation 32
Statistical analysis 32
Missing data 33
Results 33
Participants 33
Demographic characteristics 37
Time interval between screening test and reference standard for children nominally recruited as cases 38
Missing data and indeterminate results 38
Concordance between nominal case–control status and pure-tone audiometry reference standard classification 38
Accuracy of the pure-tone screen and HearCheck screener for identifying hearing impairment at the level of the ear 39
Accuracy of the pure-tone screen and HearCheck screener for identifying hearing impairment at the level of the child 43
Discussion 46
Strengths and limitations 47
Results in comparison with other studies 49

Chapter 4 False-negative results from screening tests **51**
Introduction 51
Objectives 52
Methods 52
Results 52
Literature review 52
Diagnostic accuracy study (see Chapter 3*)* 53
Discussion 55

Chapter 5 Comparison of a site with a school hearing screening programme (Nottingham) with a site without a school hearing screening programme (Cambridge) **57**
Introduction 57
Objectives 57
Methods 57
Background to study sites 57
Data collection 61
Statistical analysis 62
Results 62
Referral rate and yield 62
Discussion 66
Strengths and limitations 67

Chapter 6 Exploring the impact on the child and family of a child being referred by the school hearing screen: findings from a questionnaire survey **69**
Introduction 69
Objectives 69
Methods 69
Study population 69
Questionnaire 69
Data collection 69
Data analysis 70

For peer review only - http://bmjopen.bmj.com/site/about/guidelines.xhtml

DOI: 10.3310/hta20360 HEALTH TECHNOLOGY ASSESSMENT 2016 VOL. 20 NO. 36

Results 70
Quantitative data 70
Qualitative data 72
Discussion 77
Main findings 77
Strengths and weaknesses 77
Results in the context of other studies 78

Chapter 7 Observations of the practical implementation of screening tests for hearing in schools **79**
Introduction 79
Objectives 79
Methods 79
Sample size 80
Analysis 81
Results 81
Time taken 81
Observations of researchers and nurses 82
Discussion 83
Strengths and weaknesses 83
Results in the context of other studies 83

Chapter 8 Modelling cost-effectiveness of school entry hearing screening **85**
Introduction 85
Objectives 85
Methods 85
Overall approach 85
Decision-analytic structure 87
Initial model development 89
Model development incorporating new clinical data 90
Total referrals and cases of hearing impairment 91
Distribution of referrals over time 92
Management of hearing impairment 93
Sources of parameter inputs 93
Reviews of the literature 94
Referrals 94
Number of children with hearing impairment 95
Diagnostic accuracy 95
Probabilities of hearing impairment by severity 96
Management probabilities 96
Resource use and costs 96
Screening costs 96
Cost of travelling to appointments 97
Unit costs of the pure-tone screen and HearCheck screener 97
Management costs 99
Utilities 99
Modelling the potential costs and consequences of false-positive results 99
Anxiety 101

For peer review only

© Queen's Printer and Controller of HMSO 2016. This work was produced by Fortnum *et al.* under the terms of a commissioning contract issued by the Secretary of State for Health. This issue may be freely reproduced for the purposes of private research and study and extracts (or indeed, the full report) may be included in professional journals provided that suitable acknowledgement is made and the reproduction is not associated with any form of advertising. Applications for commercial reproduction should be addressed to: NIHR Journals Library, National Institute for Health Research, Evaluation, Trials and Studies Coordinating Centre, Alpha House, University of Southampton Science Park, Southampton SO16 7NS, UK.

For peer review only - http://bmjopen.bmj.com/site/about/guidelines.xhtml

CONTENTS

Results 101
Number of referrals to diagnostic evaluation and numbers diagnosed with hearing impairment 101
School entry hearing screening versus no screening: costs and quality-adjusted life-years 102
Sensitivity analysis 103
Discussion 104
Discussion of key assumptions 104
Strengths and weaknesses 105
Findings in comparison with other health economic evaluations 106

Chapter 9 Conclusions and recommendations **107**
Summary of findings 107
Systematic review of diagnostic accuracy (see Chapter 2*)* 107
Diagnostic accuracy study (see Chapter 3*)* 107
Assessment of false negatives (see Chapter 4*)* 107
Comparison of school entry hearing screening and non-school entry hearing screening services (see Chapter 5*)* 107
Survey of parents (see Chapter 6*)* 108
Practical implementation (see Chapter 7*)* 108
Cost-effectiveness of school entry hearing screening (see Chapter 8*)* 108
Discussion 108
Overall conclusions and recommendations 111
Implications for practice 111
Implications for research 111

Acknowledgements **113**

References **117**

Appendix 1 Executive summary from 2007 report **121**

Appendix 2 Systematic review **125**

Appendix 3 Diagnostic accuracy study: information **139**

Appendix 4 The relationship between the pure-tone screen and HearCheck screener results **159**

Appendix 5 Parent questionnaire **165**

Appendix 6 Practical implementation of screening tests for hearing in schools: information **171**

For peer review only

For peer review only - http://bmjopen.bmj.com/site/about/guidelines.xhtml

DOI: 10.3310/hta20360 HEALTH TECHNOLOGY ASSESSMENT 2016 VOL. 20 NO. 36

List of tables

TABLE 1 Study characteristics: sampling and participants 10

TABLE 2 Study characteristics: index test and reference standard 11

TABLE 3 Results from the methodological quality assessment 16

TABLE 4 Diagnostic accuracy outcomes 20

TABLE 5 Sensitivities and specificities of all studies evaluating questionnaires and included in the 2007 HTA report and the current update 23

TABLE 6 Sensitivities and specificities of all studies evaluating audiometry-based tests and included in the 2007 HTA report and the current update 24

TABLE 7 Sensitivities and specificities of all studies evaluating TEOAE and included in the 2007 HTA report and the current update 24

TABLE 8 Numbers of invitations, replies and consents by centre 35

TABLE 9 Demographic characteristics of recruited children 37

TABLE 10 Time interval in weeks between administering the screening tests (PTS and HC) and the reference standard (PTA) for children nominally recruited as cases only ($n = 75$) 38

TABLE 11 Cross-tabulation of the PTS vs. HC test results: impaired ears defined as those with a PTA score of ≥ 30 dB on at least one of the four frequencies – analyses include all impaired ears regardless of whether belonging to children nominally recruited as cases or controls 42

TABLE 12 Accuracy of the PTS and HC screener in ear-level analyses: impaired ears defined as those with a PTA score of ≥ 30 dB on at least one of the four frequencies – analyses include all impaired ears regardless of whether belonging to children nominally recruited as cases or controls 42

TABLE 13 Accuracy of the PTS and HC screener at ear level across different definitions of impairment status and using different subsets of impaired ears based on whether they belong to children nominally recruited as cases or controls 43

TABLE 14 Cross-tabulation of the PTS vs. HC test results: impaired children defined as those with a PTA score of ≥ 30 dB on at least one of the four frequencies in at least one ear – analyses include all children regardless of whether nominally recruited as case or control 45

TABLE 15 Accuracy of the PTS and HC screener in child-level analyses: impaired children defined as those with a PTA score of ≥ 30 dB on at least one of the four frequencies in at least one ear – analyses include all impaired children regardless of whether nominally recruited as case or control 45

For peer review only

© Queen's Printer and Controller of HMSO 2016. This work was produced by Fortnum *et al.* under the terms of a commissioning contract issued by the Secretary of State for Health. This issue may be freely reproduced for the purposes of private research and study and extracts (or indeed, the full report) may be included in professional journals provided that suitable acknowledgement is made and the reproduction is not associated with any form of advertising. Applications for commercial reproduction should be addressed to: NIHR Journals Library, National Institute for Health Research, Evaluation, Trials and Studies Coordinating Centre, Alpha House, University of Southampton Science Park, Southampton SO16 7NS, UK.

For peer review only - http://bmjopen.bmj.com/site/about/guidelines.xhtml

TABLE 16 Accuracy of the PTS and HC tests at child level across different definitions of impairment status and using different subsets of impaired children based on whether they were nominally recruited as cases or controls 46

TABLE 17 Two-by-two table of the PTS and HC test results for hypothetical screening population of 10,000 children where the prevalence of impairment is 0.5% and given the child-level accuracy estimates 47

TABLE 18 Comparison of referral and yield rates (as at last appointment) between Nottingham and Cambridge expressed per 1000 children per year 63

TABLE 19 Percentage of referred children who were subsequently confirmed as cases (at last appointment), stratified by site and, for Nottingham, whether or not referral was via SES 63

TABLE 20 Comparison of the mean age of referral (in years) between Nottingham and Cambridge 63

TABLE 21 Characteristics of children referred 64

TABLE 22 Characteristics of confirmed cases of hearing loss 65

TABLE 23 Parental anxiety about school hearing screen referral 71

TABLE 24 What parents think about the school entry hearing screen: thematic analysis 72

TABLE 25 Time taken (minutes) to do screening tests and comparison between the PTS and HC screener 81

TABLE 26 Advantages and disadvantages of the PTS and the HC screener as observed when used by school nurses 82

TABLE 27 Ratings of three nurses on the practical implementation of the PTS and HC screener in schools on 20 occasions – scoring from 0 (low) to 10 (maximum) 82

TABLE 28 Key data points derived from clinical studies 91

TABLE 29 Screening results: persons per hypothetical cohort of 10,000 children screened 92

TABLE 30 Diagnostic accuracy 92

TABLE 31 Percentage of children being referred to diagnostic evaluation by year: base case 93

TABLE 32 Distribution of referrals by type of referral 93

TABLE 33 Data sources used for the current model in comparison with the 2007 HTA report 94

TABLE 34 False negatives under the PTS and HC tests 95

For peer review only

For peer review only - http://bmjopen.bmj.com/site/about/guidelines.xhtml

DOI: 10.3310/hta20360 HEALTH TECHNOLOGY ASSESSMENT 2016 VOL. 20 NO. 36

TABLE 35 Distribution of hearing impairment states 96

TABLE 36 Distributions of management types 97

TABLE 37 Unit costs of the PTS and HC tests 98

TABLE 38 Unit costs of all non-screening forms of identification 99

TABLE 39 NHS management-related reference costs used in the model 100

TABLE 40 Utilities used in the model 100

TABLE 41 Rate at which children are referred to diagnostic evaluation with audiologist in each model arm: base case 101

TABLE 42 Rate at which children are diagnosed in each model arm (based on a hypothetical 10,000 population) 101

TABLE 43 Undiscounted costs 102

TABLE 44 Discounted costs 102

TABLE 45 Quality-adjusted life-years for children with hearing impairment 102

TABLE 46 Incremental costs and QALYs (discounted) 103

TABLE 47 Cross-tabulation of the PTS vs. HC results for ear-level analysis: analyses include only impaired ears that belong to children recruited as cases – impaired ears defined as those with a PTA score of ≥ 30 dB on at least one of the four frequencies 159

TABLE 48 Cross-tabulation of the PTS vs. HC test results for ear-level analyses: analyses include only impaired ears that belong to children recruited as controls – impaired ears defined as those with a PTA score of ≥ 30 dB on at least one of the four frequencies 160

TABLE 49 Cross-tabulation of the PTS vs. HC test results for ear-level analyses: analyses include all impaired ears regardless of whether belong to children recruited as case or control – impaired ears defined as those with an average PTA score of ≥ 30 dB across the four frequencies 160

TABLE 50 Cross-tabulation of the PTS vs. HC test results for ear-level analyses: analyses include only impaired ears that belong to children recruited as cases – impaired ears defined as those with an average PTA score of ≥ 30 dB across the four frequencies 161

TABLE 51 Cross-tabulation of the PTS vs. HC test results for ear-level analyses: analyses include only impaired ears that belong to children recruited as controls – impaired ears defined as those with an average PTA score of ≥ 30 dB across the four frequencies 161

For peer review only

© Queen's Printer and Controller of HMSO 2016. This work was produced by Fortnum *et al.* under the terms of a commissioning contract issued by the Secretary of State for Health. This issue may be freely reproduced for the purposes of private research and study and extracts (or indeed, the full report) may be included in professional journals provided that suitable acknowledgement is made and the reproduction is not associated with any form of advertising. Applications for commercial reproduction should be addressed to: NIHR Journals Library, National Institute for Health Research, Evaluation, Trials and Studies Coordinating Centre, Alpha House, University of Southampton Science Park, Southampton SO16 7NS, UK.

For peer review only - http://bmjopen.bmj.com/site/about/guidelines.xhtml

LIST OF TABLES

TABLE 52 Cross-tabulation of the PTS vs. HC test results for child-level analyses: analyses include only impaired children who were nominally recruited as cases – impaired children defined as those with an PTA score of ≥ 30 dB on at least one of the four frequencies 162

TABLE 53 Cross-tabulation of the PTS vs. HC test results for child-level analyses: analyses include only impaired children who were nominally recruited as controls – impaired children defined as those with a PTA score of ≥ 30 dB on at least one of the four frequencies 162

TABLE 54 Cross-tabulation of the PTS vs. HC test results for child-level analyses: analyses include all impaired children regardless of whether recruited as case or control – impaired children defined as those with an average PTA score of ≥ 30 dB across the four frequencies 163

TABLE 55 Cross-tabulation of the PTS vs. HC test results for child-level analyses: analyses include only impaired children who were nominally recruited as cases – impaired ears defined as those with an average PTA score of ≥ 30 dB across the four frequencies 163

TABLE 56 Cross-tabulation of the PTS vs. HC test results for child-level analyses: analyses include only impaired children who were nominally recruited as controls – impaired ears defined as those with an average PTA score of ≥ 30 dB across the four frequencies 164

For peer review only

For peer review only - http://bmjopen.bmj.com/site/about/guidelines.xhtml

DOI: 10.3310/hta20360 HEALTH TECHNOLOGY ASSESSMENT 2016 VOL. 20 NO. 36

List of figures

FIGURE 1 Planned participant flow 5

FIGURE 2 Flow diagram of the selection process 9

FIGURE 3 Forest plot of sensitivity and specificity of different types of hearing screening tests 18

FIGURE 4 Summary ROC plot of different hearing screening tests 19

FIGURE 5 The HC screener 30

FIGURE 6 Numbers of case children in the diagnostic accuracy study 34

FIGURE 7 Numbers of control children in the diagnostic accuracy study 37

FIGURE 8 Nominal recruitment status at the ear level and the reference standard classification by or of hearing impairment status based on a PTA score of ≥ 30 dB on at least one of the four frequencies 40

FIGURE 9 Nominal recruitment status at the child level and the reference standard classification by or of hearing impairment status based on a PTA score of ≥ 30 dB on at least one of the four frequencies for at least one ear 40

FIGURE 10 Pure-tone screen results at the ear level by hearing impairment status based on a PTA score of ≥ 30 dB on at least one of the four frequencies 41

FIGURE 11 HearCheck screener test results at the ear level by hearing impairment status based on a PTA score of ≥ 30 dB on at least one of the four frequencies 41

FIGURE 12 Pure-tone screen test results at the child level by hearing impairment status based on a PTA score of ≥ 30 dB on at least one of the four frequencies in at least one ear 44

FIGURE 13 HearCheck test results at the child level by hearing impairment status based on a PTA score of ≥ 30 dB on at least one of the four frequencies in at least one ear 44

FIGURE 14 False negatives from diagnostic accuracy study (PTA average criterion: average of four frequencies is ≥ 30 dB 54

FIGURE 15 Pathway for Nottingham patients 59

FIGURE 16 Pathway for Cambridge patients 60

FIGURE 17 Decision tree representation of SES from original bid 86

FIGURE 18 Basic decision tree structure 87

© Queen's Printer and Controller of HMSO 2016. This work was produced by Fortnum *et al.* under the terms of a commissioning contract issued by the Secretary of State for Health. This issue may be freely reproduced for the purposes of private research and study and extracts (or indeed, the full report) may be included in professional journals provided that suitable acknowledgement is made and the reproduction is not associated with any form of advertising. Applications for commercial reproduction should be addressed to: NIHR Journals Library, National Institute for Health Research, Evaluation, Trials and Studies Coordinating Centre, Alpha House, University of Southampton Science Park, Southampton SO16 7NS, UK.

For peer review only - http://bmjopen.bmj.com/site/about/guidelines.xhtml

FIGURE 19 Decision tree: screening arm 88

FIGURE 20 Transitory hearing impairment 88

FIGURE 21 Sensorineural or permanent conductive hearing impairment 89

For peer review only

For peer review only - http://bmjopen.bmj.com/site/about/guidelines.xhtml

DOI: 10.3310/hta20360 HEALTH TECHNOLOGY ASSESSMENT 2016 VOL. 20 NO. 36

List of abbreviations

AABR	automated auditory brainstem response
CCS	Cambridgeshire Community Services
CHAC	Children's Hearing Assessment Centre
CHQS	Chinese Hearing Questionnaire for School Children
CI	confidence interval
CRF	case report form
DEA	diagnostic evaluation with an audiologist
ENT	ear, nose and throat
GP	general practitioner
HC	HearCheck
HI	hearing impaired
HL	hearing level
HTA	Health Technology Assessment
ICER	incremental cost-effectiveness ratio
ID	identifier
IQR	interquartile range
NHBRU	Nottingham Hearing Biomedical Research Unit
NHI	not hearing impaired
NHSP	Newborn Hearing Screening Programme
NICE	National Institute for Health and Care Excellence
NIHR	National Institute for Health Research
OAE	otoacoustic emission
OME	otitis media with effusion
PenCTU	Peninsula Clinical Trials Unit
PSA	probabilistic sensitivity analysis
PTA	pure-tone audiometry
PTS	pure-tone screen
QALY	quality-adjusted life-year
QUADAS	quality assessment of diagnostic accuracy studies
ROC	receiver operating characteristic
SD	standard deviation
SES	school entry screen(ing)
SNPC	sensorineural or permanent conductive
TEOAE	transient-evoked otoacoustic emission
UNHS	Universal Newborn Hearing Screening
WTE	whole-time equivalent

© Queen's Printer and Controller of HMSO 2016. This work was produced by Fortnum *et al.* under the terms of a commissioning contract issued by the Secretary of State for Health. This issue may be freely reproduced for the purposes of private research and study and extracts (or indeed, the full report) may be included in professional journals provided that suitable acknowledgement is made and the reproduction is not associated with any form of advertising. Applications for commercial reproduction should be addressed to: NIHR Journals Library, National Institute for Health Research, Evaluation, Trials and Studies Coordinating Centre, Alpha House, University of Southampton Science Park, Southampton SO16 7NS, UK.

For peer review only - http://bmjopen.bmj.com/site/about/guidelines.xhtml

For peer review only

DOI: 10.3310/hta20360 HEALTH TECHNOLOGY ASSESSMENT 2016 VOL. 20 NO. 36

Plain English summary

Discovering if a child has problems with their hearing is important for development and education. Screening babies when they are born identifies most problems but hearing problems can start at any age. Many parts of the UK screen children for hearing problems when they start school but others have stopped doing this. We wanted to see whether having the screen was better or worse than not having it. We also wanted to see what was the best test to use.

We compared the screening test that is most commonly used, the pure-tone screen (PTS) (Amplivox, Eynsham, UK), with a hand-held device, the HearCheck screener (Siemens, Frimley, UK), which might be quicker to use but has not been used in schools before.

We found the two tests were equally good at finding children with hearing problems but that school nurses preferred to use the PTS.

We compared an area that has a school hearing screening programme (Nottingham) with an area that does not (Cambridge). We found that more children were referred for further testing in the area that did not have the screen but there was little evidence of a difference between the areas in terms of finding the children with problems.

We found that not having the screen when children start school is as good as having it in terms of finding children with problems and is probably better value but there has to be another system in place to find children. This usually depends on parents, schools and health professionals noticing promptly when a child might have hearing problems and referring them.

For peer review only

© Queen's Printer and Controller of HMSO 2016. This work was produced by Fortnum *et al.* under the terms of a commissioning contract issued by the Secretary of State for Health. This issue may be freely reproduced for the purposes of private research and study and extracts (or indeed, the full report) may be included in professional journals provided that suitable acknowledgement is made and the reproduction is not associated with any form of advertising. Applications for commercial reproduction should be addressed to: NIHR Journals Library, National Institute for Health Research, Evaluation, Trials and Studies Coordinating Centre, Alpha House, University of Southampton Science Park, Southampton SO16 7NS, UK.

For peer review only - http://bmjopen.bmj.com/site/about/guidelines.xhtml

For peer review only

DOI: 10.3310/hta20360 HEALTH TECHNOLOGY ASSESSMENT 2016 VOL. 20 NO. 36

Scientific summary

Background

Identification of permanent hearing impairment at the earliest possible age is crucial to maximise the development of speech and language, and contribute to the best opportunities for educational achievement and quality of life. Approximately 1 in every 1000 children in the UK is born with a permanent bilateral hearing impairment > 40 dB (average across four frequencies: 0.5, 1, 2 and 4 kHz) and a further 0.6 per 1000 has a unilateral impairment. This equates to 800 children per year born with a permanent bilateral hearing impairment (moderate or greater) and 500 with a unilateral impairment. The introduction of the highly sensitive and specific Universal Newborn Hearing Screening (UNHS) programme has led to the identification of the vast majority of children born with a hearing impairment who undergo the screen. However, not all children who will ultimately have a hearing impairment are identifiable at birth.
The adjusted prevalence of permanent hearing impairment > 40 dB (average of 0.5, 1, 2 and 4 kHz) at age 3 years is reported as 1.07 per 1000 and the prevalence for children aged 9–15 years as 2.05 per 1000. Thus, because of acquisition, progression or late onset of hearing impairment and/or geographical movement of families, there remains a significant number of children to be identified with a permanent hearing impairment after the newborn period. The onset of hearing impairment in children can occur at any time, which means there is no optimum time for a further universal hearing screen. The universal distraction hearing test, established in the UK in the 1950s and undertaken by health visitors at around 8 months of age, was abandoned following the introduction of UNHS, based on a lack of robust implementation and a low yield of cases. Without formal screening between the newborn period and school entry, identification of hearing impairment in children is achieved through parental and professional awareness and a close follow-up of children who pass the neonatal screen but are considered to be at risk. A universal hearing screen when children start school, the school entry screening (SES) programme, was established in 1955 and remains in place in many parts of the UK. It is considered as a 'back-stop' screen to identify children as part of a 'captive population' at school entry.

Objectives

The overarching aims of this project were to evaluate the diagnostic accuracy of hearing screening tests and the cost-effectiveness of screening for hearing impairment at school entry in the UK.

The specific research objectives of this project were:

- to update the latest systematic review of diagnostic accuracy of tests used for SES, summarising the literature that has been published since the previous review and drawing together the evidence from the previous review and the updated review
- to estimate and compare the diagnostic accuracy of the pure-tone screen (PTS) (Amplivox, Eynsham, UK), and HearCheck (HC) screener (Siemens, Frimley, UK) tests for discriminating between children with a hearing impairment (of any type) and children with no hearing impairment, using pure-tone audiometry (PTA) results as the reference standard
- to investigate the impact of a potential false-negative result by reviewing the literature on the impact of false-negative results from screening tests and describing children with false-negative screening results in the diagnostic accuracy study

For peer review only

© Queen's Printer and Controller of HMSO 2016. This work was produced by Fortnum *et al.* under the terms of a commissioning contract issued by the Secretary of State for Health. This issue may be freely reproduced for the purposes of private research and study and extracts (or indeed, the full report) may be included in professional journals provided that suitable acknowledgement is made and the reproduction is not associated with any form of advertising. Applications for commercial reproduction should be addressed to: NIHR Journals Library, National Institute for Health Research, Evaluation, Trials and Studies Coordinating Centre, Alpha House, University of Southampton Science Park, Southampton SO16 7NS, UK.

For peer review only - http://bmjopen.bmj.com/site/about/guidelines.xhtml

- to compare children referred for investigation of suspected hearing impairment in a geographical area that applies a routine SES programme (Nottingham) with those referred in an area with no routine SES programme (Cambridge) with respect to the number of referrals, the age at referral, the source of referral, the route through assessment to intervention, the number of children ultimately identified to have a hearing impairment (yield) and the nature of hearing impairment identified
- to determine the impact, both psychological and economic, for the child and the family of the child being referred for further assessment following SES (both true and false positives)
- to determine the time resource in implementing either of the two alternative screening methods (PTS and HC) in primary schools and to elicit the views of the school nurses implementing the screening tests
- to refine an existing SES economic model {from the 2007 Health Technology Assessment (HTA) report [Bamford J, Fortnum H, Bristow K, Smith J, Vamvakas G, Davies L, *et al.* Current practice, accuracy, effectiveness and cost-effectiveness of the school entry hearing screen. *Health Technol Assess* 2007;**11**(32)]} and assess the cost-effectiveness of the SES programme
- to estimate the health-related quality of life, costs and utilities of the SES programme compared with no screening, and of the PTS compared with HC screener, with comparisons based on cost per quality-adjusted life-year (QALY) gained.

Methods

In order to explore and summarise the existing literature we updated the review of diagnostic test accuracy reported in a previous HTA report and reviewed the literature on false-negative rates in hearing screening.

For children with a known hearing impairment and for children assumed to have no hearing impairment we compared the diagnostic accuracy of two screening methods administered at or around the time children start school. These were the established and widely used PTS (which is applied using headphones) and HC screener (a hand-held PTS). We used PTA as a reference standard.

The yield, referral age and route through assessment to intervention for childhood hearing impairment were assessed for a paediatric audiology service that implements a routine universal SES programme (Nottingham) and one that does not (Cambridge) by collecting data prospectively for all children aged between 3 years and 6 years 364 days.

We surveyed parents of children referred from the SES programme in Nottingham via a postal questionnaire to assess the impact for the child and the family of a positive result from a screen (both true and false positives).

We determined the time spent in implementing either of the two screening tests in primary schools and explored the practical issues involved and the views of nurses conducting the screening tests.

The component data from each study were used to refine an existing SES economic model, providing robust estimates of key parameters beyond accuracy of SES to be assessed; in particular, the yield and nature of hearing impairment detected in a system with no SES progamme; the yield, consequences and costs of screen-positive individuals in an SES programme; and the costs of setting up a SES programme.

For peer review only - http://bmjopen.bmj.com/site/about/guidelines.xhtml

DOI: 10.3310/hta20360 HEALTH TECHNOLOGY ASSESSMENT 2016 VOL. 20 NO. 36

Results

The updated review of diagnostic accuracy studies confirms the conclusion from the 2007 HTA report that research to date demonstrates marked variability in the design, methodological quality and results. Robust conclusions about the performance of individual test types for use in SES cannot be drawn. It was found that:

- Parental questionnaires had the poorest diagnostic accuracy compared with all other tests.
- The findings from the new audiometry-based studies evaluating computer-based devices and the HC screener reported higher and more consistent specificity but lower and widely varying sensitivity estimates compared with the sweep PTA studies included in the original report.
- Studies evaluating transient-evoked otoacoustic emissions reported variable sensitivity with wide confidence intervals (CIs), whereas specificity estimates were relatively high and more consistent.
- The study evaluating the automated auditory brainstem response reported high sensitivity and specificity.

The review included studies from countries with and without an established UNHS system and with very different systems of health-care delivery. The generalisability of the findings to other situations, including the UK NHS system, is likely to be limited.

The findings of our diagnostic accuracy study indicate that the PTS and HC devices have a high level of sensitivity (PTS ≥ 89%, HC ≥ 83%) and specificity (PTS ≥ 78%, HC ≥ 83%) for identifying hearing impairment at the level of the ear. These conclusions appear robust, the child-level analyses indicating similar levels of sensitivity and specificity.

From our review of the existing literature and data from the diagnostic accuracy study, we are unable to quantify the effect of false-negative results for the PTS or HC screener, but were able to confirm that the rate was extremely low. Of the 16 ears in our diagnostic study (total $n = 630$) that passed one or both of the screening tests but were referred by the PTA measure, only four were confirmed to have a hearing impairment at diagnostic evaluation and all were mild.

There was strong evidence that the rate of referral for hearing problems is lower when a SES programme is present. The referral rate was 36% lower in Nottingham (SES) relative to Cambridge (no SES) (rate ratio 0.64, 95% CI 0.59 to 0.69; $p < 0.001$).

There was little evidence that the yield of confirmed cases differs between areas with and without a SES programme (rate ratio 0.82, 95% CI 0.63 to 1.06; $p = 0.12$); a higher proportion of referred children were subsequently confirmed to be hearing impaired in the area with a SES programme (17.0% in Nottingham vs. 10.6% in Cambridge).

The mean age of referral was nearly identical between areas with and without a SES programme when looking at all referrals, but for children who were subsequently confirmed as having a hearing impairment there was strong evidence that the children in the site with a screening programme are older at referral (mean age difference 0.47 years, 95% CI 0.24 to 0.70 years; $p < 0.001$).

We found from our survey of parents of children referred by the SES programme in Nottingham that the consequences of the referral process for parents and children, including false positives, are minor. The difference for parents whose child is referred by the SES programme is that they may have had no concerns prior to the screening test.

We demonstrated minimal differences between the PTS and HC screener in terms of time taken to conduct each examination and practical issues. Testing covered a range of schools throughout the school year and thus we suggest the findings might be generalisable beyond the Nottingham schools.

© Queen's Printer and Controller of HMSO 2016. This work was produced by Fortnum *et al.* under the terms of a commissioning contract issued by the Secretary of State for Health. This issue may be freely reproduced for the purposes of private research and study and extracts (or indeed, the full report) may be included in professional journals provided that suitable acknowledgement is made and the reproduction is not associated with any form of advertising. Applications for commercial reproduction should be addressed to: NIHR Journals Library, National Institute for Health Research, Evaluation, Trials and Studies Coordinating Centre, Alpha House, University of Southampton Science Park, Southampton SO16 7NS, UK.

For peer review only - http://bmjopen.bmj.com/site/about/guidelines.xhtml

Our economic modelling showed that SES is unlikely to be cost-effective and, using base-case assumptions, is dominated by a no screening strategy. This is consistent with the observed results of the clinical studies, which suggest that cases of hearing impairment are identified in similar numbers but at a younger average age in the absence of SES.

Two situations where SES might be cost-effective were identified. In the first situation, a reduction in the number of referrals associated with SES or, conversely, an increase in referrals without SES, can give a cost-effectiveness ratio for the no screening option above the National Institute for Health and Care Excellence (NICE) £30,000 per QALY benchmark. This is supported by the observation from our clinical study that the referral rate (and by assumption, potential false positive rate) was lower in the site where SES had been in place for many years. However, in order for this to be the case, the reduction in referrals would need to be attributable to SES and there is considerable uncertainty about this. The second situation is subject to still greater uncertainty and requires referrals to happen more quickly with screening than is observed from our study comparing SES and non-SES sites.

Conclusions

In the context of the UK NHS, and similar health-care systems, SES using screening tests, such as the PTS and HC screener, is unlikely to be effective in increasing the number of cases of hearing impairment identified and lowering the average age at which these cases were identified. SES is also unlikely to be cost-effective when judged against the benchmarks normally used by NICE, relative to a system entirely reliant on ad-hoc referral when a suspicion of hearing impairment is raised.

Implications for practice

Although our finding of the lack of cost-effectiveness of SES may be considered as a reason to withdraw SES where it is currently being practised, we would highlight aspects of the results that suggest caution. First, we have shown that there are at least two scenarios in which it may be cost-effective. Second, our findings are very dependent on findings in the two specific areas (Nottingham and Cambridge) that were used here, and our conclusions from comparing areas with a SES programme and without a SES programme may not be generalisable to other areas. Third, the cost-effectiveness of SES depends on how effective (or ineffective) the 'no SES system' is. This in turn is highly dependent on the effectiveness of ad-hoc identification and referral for a diagnostic evaluation with an audiologist (DEA), which is not only largely unknown, but likely to be variable. It seems plausible that SES may have greater potential to be cost-effective where ad-hoc identification and referral is less well developed than in a system where it is well established. If withdrawal of the SES programme is to be considered it needs to be carefully managed to ensure that the ad-hoc referral system is working effectively. Health professionals, school and nursery staff, and parents who would then be responsible for referral of children about whom there were concerns in the school entry year may need to be reminded to be more vigilant for signs of hearing impairment.

Implications for research

Systematic reviews of the accuracy of devices, which might be used to measure hearing in children at around school entry age, should continue to be pursued.

Characterising and measuring the cost-effectiveness of different approaches to the ad-hoc referral system with a view to optimising it should be undertaken.

Examination of the process by which concern, or referral from SES, is converted into DEAs would be useful to inform further research on what determines programme specificity (as opposed to test specificity).

For peer review only - http://bmjopen.bmj.com/site/about/guidelines.xhtml

DOI: 10.3310/hta20360 HEALTH TECHNOLOGY ASSESSMENT 2016 VOL. 20 NO. 36

We should improve understanding of why the referral rate varies across different sites and determine if this is related to the presence of SES. Further observational studies similar to our comparison between Nottingham and Cambridge could be undertaken, albeit recognising the difficulty of matching the geographical areas.

Further research to better quantify the impact of referral, particularly with respect to anxiety, and whether or not all referrals are affected to the same degree as respondents in our study may be required, particularly if it appears that overall effectiveness and cost-effectiveness could be critically dependent on the costs and disutility experienced by false positives.

If withdrawal of SES is contemplated in particular settings, this could be used as an opportunity for further data collection; in particular where the pattern of referrals and cases was known over many years in the run up to withdrawal, any change in pattern of referrals/cases could be very useful evidence confirming the lack of effectiveness and cost-effectiveness of SES, or challenging it. More formally, if SES cessation is being contemplated in many areas, a randomised trial of withdrawal of SES services could be designed using referrals and hearing impairment cases identified as outcomes.

Trial registration

This trial is registered as ISRCTN61668996.

Funding

Funding for this study was provided by the Health Technology Assessment programme of the National Institute for Health Research.

For peer review only

© Queen's Printer and Controller of HMSO 2016. This work was produced by Fortnum *et al.* under the terms of a commissioning contract issued by the Secretary of State for Health. This issue may be freely reproduced for the purposes of private research and study and extracts (or indeed, the full report) may be included in professional journals provided that suitable acknowledgement is made and the reproduction is not associated with any form of advertising. Applications for commercial reproduction should be addressed to: NIHR Journals Library, National Institute for Health Research, Evaluation, Trials and Studies Coordinating Centre, Alpha House, University of Southampton Science Park, Southampton SO16 7NS, UK.

For peer review only - http://bmjopen.bmj.com/site/about/guidelines.xhtml

For peer review only

DOI: 10.3310/hta20360 HEALTH TECHNOLOGY ASSESSMENT 2016 VOL. 20 NO. 36

Chapter 1 Background and main questions

Childhood hearing impairment and screening

Identification of permanent hearing impairment at the earliest possible age is crucial to maximise the development of speech and language and contribute to the best opportunities for educational achievement and quality of life.[1] Approximately 1 in every 1000 children born in the UK has a permanent bilateral hearing impairment of > 40 dB (average across four frequencies: 0.5, 1, 2 and 4 kHz) and a further 0.6 per 1000 has a unilateral impairment.[2] This equates to 800 children per year born with a permanent bilateral hearing impairment (moderate or greater) and 500 with a unilateral impairment. The introduction of the highly sensitive and specific Universal Newborn Hearing Screening (UNHS) programme has led to the identification of the vast majority of children born with a hearing impairment who undergo the screen.[3,4] However, not all children who will ultimately have a hearing impairment are identifiable at birth. Data published in 2001[5] reported an adjusted prevalence of permanent hearing impairment of > 40 dB (average of 0.5, 1, 2 and 4 kHz) at age 3 years of 1.07 per 1000 and a prevalence for children aged 9–15 years of 2.05 per 1000. Thus, because of acquisition, progression or late onset of hearing impairment and/or geographical movement of families, there remain a significant number of children to be identified with a permanent hearing impairment after the newborn period. The onset of hearing impairment in children after the newborn period can occur at any time, which means there is no optimum time for a further universal hearing screen. The universal distraction hearing test, established in the UK in the 1950s and undertaken by health visitors at around 8 months of age, was abandoned following the introduction of UNHS, based on a lack of robust implementation and a low yield of cases.[6,7] Identification of hearing impairment in children in the time between the newborn period and school entry is achieved through parental and professional awareness and a close follow-up of children who pass the neonatal screen but are considered to be at risk.[8] A universal hearing screen when children start school, the school entry screening (SES) programme, was established in 1955 and remains in place in many parts of the UK. It is considered as a 'back-stop' screen to identify children as part of a 'captive population' at school entry. Note: SES always refers to the hearing screening in this report.

A number of studies and reviews[2,9–12] have explored evidence for the value of the SES programme but without clear conclusions. Research has shown that the number of children identified by this screen around age 5 years (the yield) has decreased following the introduction of UNHS, and widespread development of a system that is responsive to professional and parental concerns at any age.[8,12] The SES programme is no longer universally applied. Bamford *et al.*[12] reported in 2007 that one in eight services had stopped offering the screen by 2005 and it is more likely that others have stopped since 2005 than that services have reinstated the screen. There are no guidelines on standard methodology nationally and procedures are variable. However, despite a lack of evidence of its value, many support its continuation as a 'back-stop' to identify an acknowledged small number of children who would otherwise not be identified with the consequent effect on their development of speech and language.

Childhood hearing impairment can be permanent (usually sensorineural although permanent conductive impairment also occurs) and will not improve, or transient (also referred to as conductive) and will usually fluctuate and can get better.

A sensorineural impairment occurs when the inner ear (cochlea) or auditory nerve does not function properly; this can be caused by many factors. As mentioned, the majority of children with sensorineural impairments will have been identified by the newborn screening programme. However, there is the possibility that a child has a progressive impairment that, at birth, was minimal and therefore enabled them to pass the newborn screen. It is also possible for a child to develop a sensorineural impairment. Sensorineural impairments are permanent and require management to ensure that the child has access to language; management is usually to fit children with hearing aids or cochlear implants.

© Queen's Printer and Controller of HMSO 2016. This work was produced by Fortnum *et al.* under the terms of a commissioning contract issued by the Secretary of State for Health. This issue may be freely reproduced for the purposes of private research and study and extracts (or indeed, the full report) may be included in professional journals provided that suitable acknowledgement is made and the reproduction is not associated with any form of advertising. Applications for commercial reproduction should be addressed to: NIHR Journals Library, National Institute for Health Research, Evaluation, Trials and Studies Coordinating Centre, Alpha House, University of Southampton Science Park, Southampton SO16 7NS, UK.

For peer review only - http://bmjopen.bmj.com/site/about/guidelines.xhtml

BACKGROUND AND MAIN QUESTIONS

Many more children will have a transient hearing impairment at some point in their childhood than will have a permanent impairment. A conductive loss occurs when there is a problem with the outer or middle ear, such as impacted wax in the ear canal or a build up of fluid in the middle ear [otitis media with effusion (OME), which usually is associated with colds and respiratory tract infections]. Conductive impairments are usually temporary, unless there is a malformation of the outer or middle ear, which can lead to a permanent impairment. Prevalence is greatest in the first year of life and again at around age 4–5 years. For children < 6 years, 80% will experience OME.[13,14] Most episodes of OME get better in a couple of months but a small proportion (3–4%) can be persistent and severe and lead to problems with behaviour, communication and progress at school. It is therefore important to identify those children with an ongoing problem, as their hearing impairment needs management to ensure that they can access spoken language clearly. The recommended management options are initially to watch and wait. Owing to the fact that the majority of these transient impairments will resolve spontaneously, most children will be observed for 3 months before any active management takes place, then the usual options are surgical insertion of grommets if the child's hearing impairment meets national recognised criteria,[15] or for them to be fitted with a hearing aid.

Some children with permanent sensorineural impairments may also have an additional transient conductive impairment that would require management.

Screening tests for hearing impairment identify any child who has a hearing impairment. The tests do not discriminate between a permanent impairment and a transient impairment. They are also able to identify a hearing impairment in one ear or in both ears. Thus, all children with a hearing impairment, whether permanent or transient, bilateral or unilateral, should be identified by the screening test if it is 100% sensitive.

Testing children between the ages of 4 and 6 years can be difficult. Most tests require the engagement of the child in a simple task, such as raising their hand or putting a ball on a stick. Some children may find it difficult to maintain their attention throughout the test and this can give rise to spurious results. It is important that the screener/tester has experience in working with children to enable them to identify when their attention could be affecting the results of the test and be able to change their own behaviour accordingly to engage with the child. At any age it is possible that some children may not be able to co-operate with the testing because of specific learning or behavioural needs.

The pure-tone screen (PTS) (Amplivox, Eynsham, UK) test involves placing headphones over the child's ears and then presenting pure tones across the key frequencies for speech understanding (0.5, 1, 2 and 4 kHz). The child needs to indicate by a simple action that they have heard a sound. The screen works on the basis that a child needs to hear two out of three presentations of each frequency at 20 dB hearing level (HL) in each ear to pass the screen.

The HearCheck (HC) screener (Siemens, Frimley, UK) is placed over the child's ear and an automatic sequence of pure tones is played once at each of three levels at each of the frequencies 1 kHz (55 dB, 35 dB and 20 dB) and 3 kHz (75 dB, 55 dB, 35 dB), which is six tones in total. The child needs to indicate, usually by raising their hand that they have heard each tone.

Any child identified by a screening test as having a possible hearing impairment should be referred to audiology services, but many children at school entry have a transient conductive impairment and hence it is the case that many children referred will ultimately be found to have no permanent impairment.

The commonly used screening method of the PTS can identify all types of impairment. Specifically including tympanometry would indicate a problem with the middle ear, but only by including both air and bone conduction thresholds from pure-tone audiometry (PTA), masked where necessary, could a conductive impairment be indicated.

For peer review only - http://bmjopen.bmj.com/site/about/guidelines.xhtml

DOI: 10.3310/hta20360 HEALTH TECHNOLOGY ASSESSMENT 2016 VOL. 20 NO. 36

This is important because there are conflicting opinions on the target group of children to be identified by SES. Should it be designed to only identify children with a permanent impairment for whom intervention is a priority, or should it be designed to identify any impairment to ensure every child is assessed appropriately and intervention provided for all who would benefit, regardless of the permanence of the condition? For this report, in both the analyses of diagnostic accuracy in *Chapter 3* and in the analyses of yield in *Chapter 5*, we have considered identification of any type of impairment as the outcome for assessing the screening tests or the screening programme as a whole.

Previous Health Technology Assessment study

A previous National Institute for Health Research (NIHR) Health Technology Assessment (HTA) programme-commissioned study to evaluate SES by Bamford *et al.*[12] reported a survey of current practice, longitudinal data on yield, a systematic review of effectiveness of SES (which included a systematic review of the diagnostic accuracy of screening tests) and an economic model estimating cost-effectiveness. The 2007 HTA report[12] concluded that there was insufficient good-quality data on which to base a decision about the value of SES following the introduction of UNHS. The executive summary of the report is included as *Appendix 1*.

However, the 2007 study did report longitudinal data from a single district in London which indicated a small but significant number of children with a permanent hearing impairment first identified via SES in that particular population,[8] and national survey data which reported examples of children not identified by other methods.

One of the recommendations of the 2007 HTA report[12] was the need for trials to compare the effectiveness and cost-effectiveness of alternative approaches to the identification of a post-newborn screen for permanent hearing impairment. Studies concerned with the relative accuracy (in terms of sensitivity and specificity) of alternative screening tests are difficult to compare and are often flawed by differing referral criteria and differing case definitions. The 2007 HTA report[12] identified 25 publications reporting studies of alternative screens or tests for screening at school entry. These data indicate that, using full PTA as the reference standard, the PTS test appears to have high sensitivity and high specificity for minimal, mild and greater hearing impairments – better than alternative tests for which evidence was identified. Spoken word tests were reported with acceptable levels of sensitivity and specificity but are variable in their implementation. Otoacoustic emissions (OAEs), tympanometry, acoustic reflectometry, parental questionnaires and otoscopy were reported with either variable or poor sensitivity and specificity. Only one study, published in 1980,[16] has compared screening with no screening and the results were inconclusive.

Assessment of cost-effectiveness

In order to best provide a service for the identification of permanent childhood hearing impairment while making best use of scarce NHS resources, it is important to gather robust evidence to support particular cost-effective implementations of service delivery at times relevant to the aetiology of hearing impairment and the child's development. There is no question that screening for hearing impairment at birth is efficient and cost-effective,[3] but the value of any further universal screen remains uncertain. Aside from the minimal and very weak evidence for the effectiveness and cost-effectiveness of different implementations of SES reported by the 2007 HTA report[12] we are not aware of literature on the resource implications of different implementations or different technologies. A version of the HC screener has been evaluated as a screening tool in children in only one published paper,[17] but it did not report any resource use.

© Queen's Printer and Controller of HMSO 2016. This work was produced by Fortnum *et al.* under the terms of a commissioning contract issued by the Secretary of State for Health. This issue may be freely reproduced for the purposes of private research and study and extracts (or indeed, the full report) may be included in professional journals provided that suitable acknowledgement is made and the reproduction is not associated with any form of advertising. Applications for commercial reproduction should be addressed to: NIHR Journals Library, National Institute for Health Research, Evaluation, Trials and Studies Coordinating Centre, Alpha House, University of Southampton Science Park, Southampton SO16 7NS, UK.

For peer review only - http://bmjopen.bmj.com/site/about/guidelines.xhtml

Study aims and objectives

The two overarching aims of this project were to evaluate the diagnostic accuracy of hearing screening tests and to assess the cost-effectiveness of screening for hearing impairment at school entry. The specific research objectives of this project were:

- To determine and compare the diagnostic accuracy of two screening methods used to identify hearing impairment at or around school entry. These are the widely established PTS (which is applied using headphones) and the HC screener, a hand-held PTS.
- To investigate the impact of a potential false-negative result.
- To update the 2007 HTA report[12] systematic review of diagnostic accuracy of tests used for SES.
- To assess the yield, referral age and route through assessment to intervention for childhood hearing impairment and measure the costs of referrals for a service that employs a routine SES programme and for a service that does not.
- To determine the impact, both psychological and economic, on the child and the family of the child being referred for further assessment following the school entry hearing screen (both true and false positives).
- To determine the resource costs in implementing either of the two alternative screening methods in primary schools and to elicit the views of the school nurses implementing the screening tests.
- To refine an existing SES economic model (from the 2007 HTA report[12]) and to assess the cost-effectiveness of SES.
- To provide estimates of the yield and nature of hearing impairment detected in a system with no SES system; the yield, consequences and costs of screen-positive individuals in a SES system; and the costs of setting up a SES system.

This study thus addressed the question of whether or not there should be a screening programme to identify permanent hearing impairment in children when they start primary school. It assessed if the cost of such a screen is appropriate for the outcomes achieved, that is, the number of children identified by this method compared with a system with no screen, which is responsive to parental or professional concern, along with comparisons of diagnostic accuracy of two different ways of doing the screen. Based on the findings, we make recommendations to contribute to decisions regarding the continued implementation of SES and the form that implementation should take.

Structure of the project and the report

The project comprised four primary studies, a questionnaire survey and two systematic reviews. The planned participant flow for the four studies is illustrated in *Figure 1*.

Chapter 2 reports results from a systematic review undertaken to update the 2007 HTA report[12] review of diagnostic accuracy of tests used for SES.

Chapter 3 reports results from the study that assessed the diagnostic accuracy of two methods of screening for the identification of hearing impairment for children aged 4–6 years. The PTS (four frequencies and one level) was compared with the HC screener (two frequencies and three levels) with respect to sensitivity and specificity.

Chapter 4 reports results from a systematic review of the issues around false negatives in screening for hearing impairment, and from the diagnostic accuracy study described above.

Chapter 5 reports and compares outcomes, including yield and age at referral, for an area where SES is in place (Nottingham) and an area where there has been no SES since 1997 (Cambridge).

For peer review only - http://bmjopen.bmj.com/site/about/guidelines.xhtml

DOI: 10.3310/hta20360 HEALTH TECHNOLOGY ASSESSMENT 2016 VOL. 20 NO. 36

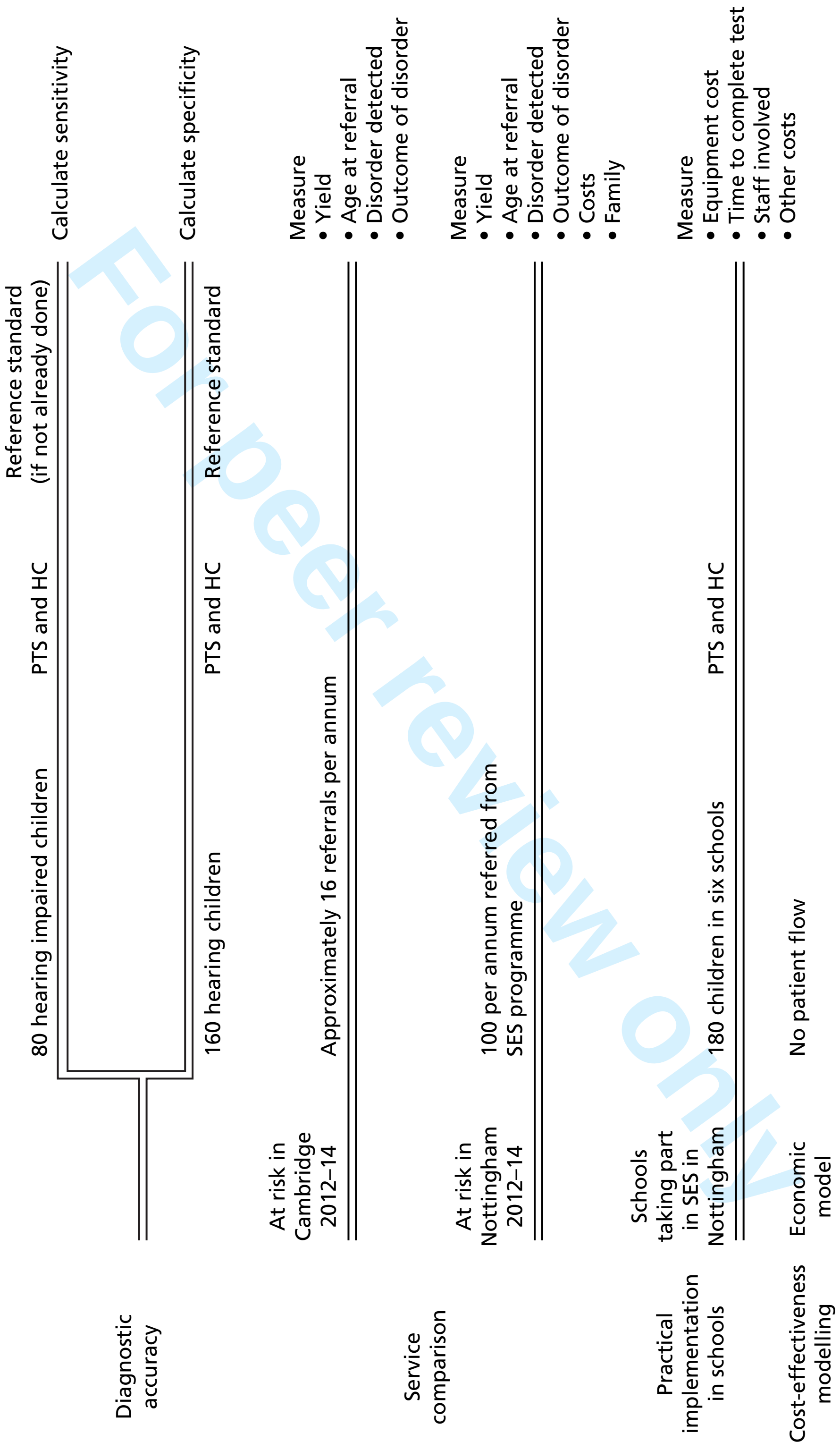

FIGURE 1 Planned participant flow.

© Queen's Printer and Controller of HMSO 2016. This work was produced by Fortnum *et al.* under the terms of a commissioning contract issued by the Secretary of State for Health. This issue may be freely reproduced for the purposes of private research and study and extracts (or indeed, the full report) may be included in professional journals provided that suitable acknowledgement is made and the reproduction is not associated with any form of advertising. Applications for commercial reproduction should be addressed to: NIHR Journals Library, National Institute for Health Research, Evaluation, Trials and Studies Coordinating Centre, Alpha House, University of Southampton Science Park, Southampton SO16 7NS, UK.

For peer review only - http://bmjopen.bmj.com/site/about/guidelines.xhtml

For peer review only

Chapter 6 reports results from a questionnaire survey of parents of children referred to audiology services in Nottingham from SES. Data on parent experience are reported.

Chapter 7 reports results of a study of the practical implementation of the two screening tests in a primary school environment.

Chapter 8 reports results from the cost-effectiveness model of SES. Data from *Chapters 3*, *5*, *6* and *7* informed the parameters in the model.

Finally, *Chapter 9* summarises the discussion points from each part of the project and makes conclusions and recommendations.

Patient and public involvement

We acknowledge the importance of the involvement of members of the public in all health research. This project evaluated a screening system for children and input from parents to the development and interpretation of the research question was very important. Recruiting such a person proved challenging, as parents have childcare responsibilities and/or employment considerations, which mean they have little time to spare and commit to a 2.5-year project. Having advertised and discussed the project with several parents, most of whom were unable to commit the time, we recruited the parent of a child who had experienced conductive hearing impairment during his early school years. This parent became a full member of the research team (JW).

He provided comments on research literature for parents, including the questionnaire used for the survey of parents of children referred to audiology services from SES. He also contributed to the development of methodology, offering advice on how to deal with lower-than-expected recruitment. He attended all the meetings of the research team, either in person or via the telephone, and contributed to discussions of the findings, presentation of results and development of recommendations. The project addressed various issues concerned with the identification of hearing impairment in children, and we recognise that individual parents may have experience of only one part of the service. Nonetheless, they bring the lay perspective to research design and, as an individual, represent other parents. To access input from a wider range of parents we recruited a representative from the National Deaf Children's Society to the project steering group. Based on the guidelines issued by INVOLVE (the organisation funded by NIHR to support public involvement in NHS, public health and social care research) we provided reimbursement to the parent joining the research team for their input of an average of 1 day per month to advise the design and attend research meetings. In addition all travel costs were reimbursed.

For peer review only

For peer review only - http://bmjopen.bmj.com/site/about/guidelines.xhtml

DOI: 10.3310/hta20360 HEALTH TECHNOLOGY ASSESSMENT 2016 VOL. 20 NO. 36

Chapter 2 Update of the diagnostic accuracy systematic review

Introduction

This chapter presents an update of the systematic review of the diagnostic test accuracy undertaken in the 2007 HTA report.[12] That report identified 25 publications reporting studies of alternative screens or tests for screening at school entry, showing that, using full PTA as the reference standard, the PTS test appears to have high sensitivity and high specificity for minimal, mild and greater hearing impairments – better than alternative tests for which evidence was identified. Other tests (spoken word tests, OAEs, tympanometry, acoustic reflectometry, parental questionnaires and otoscopy) had either variable or poor sensitivity and specificity. Using additional evidence published since the 2007 HTA report, the aim of this update was to produce an updated summary of diagnostic test accuracy.

Objectives

To update the 2007 HTA report systematic review of diagnostic accuracy of tests used for SES. This work summarises the literature that has been published since the previous review and draws together the evidence from the previous review and the updated review.

Methods

Search strategy

The search strategy used and published in the 2007 HTA report on SES[12] was reviewed and updated by an information specialist in the Peninsula Technology Assessment Group and re-run to identify studies published in the period January 2005 (search cut-off date of 2007 report, May 2005) to July 2014 (see *Appendix 2*). The following electronic databases were searched: The Cochrane Library (via Wiley) (Cochrane Database of Systematic Reviews, Cochrane Central Register of Controlled Trials, Database of Abstracts of Reviews of Effects), MEDLINE and MEDLINE In-Process & Other Non-Indexed Citations (via Ovid), EMBASE (via Ovid), Cumulative Index to Nursing and Allied Health Literature (via Ovid), PsycINFO (via Ovid), Science Citation Index (via Web of Science), Education Resources Information Center and ongoing trial databases (National Research Register, ClinicalTrials.gov and Research Findings Register).

For peer review only

© Queen's Printer and Controller of HMSO 2016. This work was produced by Fortnum *et al.* under the terms of a commissioning contract issued by the Secretary of State for Health. This issue may be freely reproduced for the purposes of private research and study and extracts (or indeed, the full report) may be included in professional journals provided that suitable acknowledgement is made and the reproduction is not associated with any form of advertising. Applications for commercial reproduction should be addressed to: NIHR Journals Library, National Institute for Health Research, Evaluation, Trials and Studies Coordinating Centre, Alpha House, University of Southampton Science Park, Southampton SO16 7NS, UK.

For peer review only - http://bmjopen.bmj.com/site/about/guidelines.xhtml

Inclusion/exclusion criteria

The inclusion and exclusion criteria were identical to those of the 2007 HTA report[12] unless stated otherwise:

- Study design: we included all primary diagnostic accuracy studies regardless of their specific design.
- Population: we included children 4–6 years old. We excluded studies with a discrete age range that was completely outside our criteria (e.g. 1–3 years or 7–10 years). If a study included 4- to 6-year-old children but the age range was too wide (e.g. 6–19 years) and the results for different age categories were not reported separately, the study was also excluded from the review. We included, however, studies that at least partially covered but slightly exceeded the 4–6 years age range (e.g. 5–10 years) provided they met all other inclusion criteria. Whenever relevant and possible, the age of the included children is noted in the list of excluded full-text studies (see *Appendix 2*).
- Screening tests or programmes: studies that evaluated one or more of the following hearing screening tests were included: sweep PTA, single-frequency PTA, transient-evoked otoacoustic emissions (TEOAE), distortion product otoacoustic emissions, questionnaires, otoadmittance tests, tympanometry, reflectometry and speech audiometry. (Note: sweep PTA and PTS are alternative terms for the same procedure. Where publications have used the term sweep PTA this description has been maintained.) Tests had to have been undertaken in either a primary school or the community [e.g. community clinic, family home or general practitioner (GP) surgery]. This could include hearing screening as a component of a multifaceted screen, such as a school entry medical examination.
- Comparator: no hearing screening or hearing screening based on different tests or test protocols. We did not exclude studies without a comparator (studies that evaluated a single screening test) as long as they measured the performance of the evaluated test against an acceptable reference standard.
- Reference standard: we included all studies that assessed the accuracy of the evaluated test(s) against a reference standard that included PTA.
- Outcomes: the 2007 HTA review[12] had a wider scope and also included studies that reported (1) the screen performance, that is, uptake (the number of children who actually received the screen) and yield (the number of cases identified); and (2) screen effectiveness, that is, language skills, health-related quality of life, communication skills, social interaction and educational performance. The current update focused on diagnostic accuracy only and included studies that reported the diagnostic accuracy of the evaluated test(s), regardless of whether or not the reported data were sufficient to reconstruct two-by-two table(s).
- Language: no language restrictions were applied to the search and selection of studies.

Selection of studies and data extraction

After removing the duplicates, all records identified by the electronic searches were screened independently by two reviewers (HF and ZZ) at title and abstract level. Full-text copies were obtained for all publications identified as potentially relevant by at least one of the reviewers. Their suitability for inclusion in the review was assessed by one reviewer (ZZ) and checked by a second reviewer (CH) against the criteria specified above. Data were extracted from included studies by one reviewer (ZZ), entered into an Excel 2010 (Microsoft Corporation, Redmond, WA, USA) spreadsheet and checked by a second reviewer (CH). Disagreements in the selection of studies and data extraction were resolved through discussion. Data were extracted from studies published in a language other than English with the help of a translator.

For peer review only

For peer review only - http://bmjopen.bmj.com/site/about/guidelines.xhtml

DOI: 10.3310/hta20360 HEALTH TECHNOLOGY ASSESSMENT 2016 VOL. 20 NO. 36

Assessment of the methodological quality

Although a new version of the quality assessment of diagnostic accuracy studies (QUADAS) tool used in the 2007 HTA review[12] is now available, the difference between the two versions is mainly in the structure and process of customising the tool and does not concern the contents of the actual checklist.[18,19] Therefore, we assessed the methodological quality of the included studies using the original QUADAS checklist from the 2007 HTA review. This allowed for a direct comparison between the quality of the studies included in the 2007 report and in the current update. However, we provided more specific definitions of some QUADAS items that were not explicitly defined in the original checklist (see *Appendix 2*).

Statistical analysis and data synthesis

Whenever possible, two-by-two tables were used to report the numbers of true-positive, false-positive, false-negative and true-negative results. The sensitivity and specificity of the index test (the test under evaluation) were calculated using The Cochrane Collaboration's Review Manager (RevMan) software version 5.3 (The Cochrane Collaboration, The Nordic Cochrane Centre, Copenhagen, Denmark). The same software was used to create forest plots of the paired sensitivity and specificity estimates and to plot the true-positive rate (sensitivity) versus the false-positive rate (1 - specificity) in receiver operating characteristic (ROC) space. Heterogeneity was investigated visually by examining the forest plots and the ROC plot, and a decision was made whether or not to pool the results across studies. As standard funnel plots and tests for publication bias are not recommended in systematic reviews of diagnostic accuracy studies, we did not investigate publication bias.[20]

For peer review only

Results

Search results and selection of studies

The electronic searches returned 892 papers after removal of duplicates. Screening at title or/and abstract level led to 39 citations being identified as potentially relevant. A further seven citations were found through hand-searching.

We obtained full-text copies of 45 of these studies and failed to obtain one.[21] After full-text evaluation 10 studies were selected for inclusion in the review.[17,22–30] *Figure 2* details the selection process while the reasons for exclusion of full-text papers are given in *Appendix 2*.

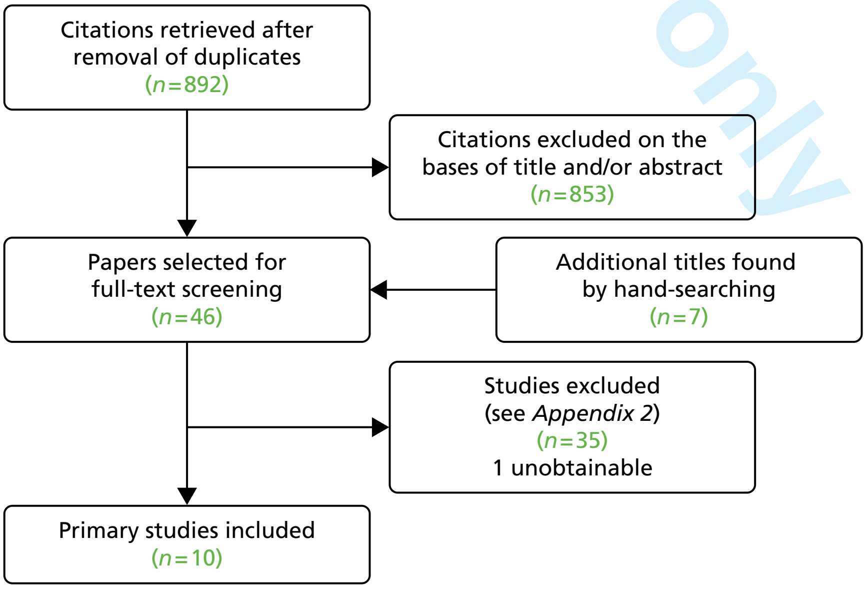

FIGURE 2 Flow diagram of the selection process.

© Queen's Printer and Controller of HMSO 2016. This work was produced by Fortnum *et al.* under the terms of a commissioning contract issued by the Secretary of State for Health. This issue may be freely reproduced for the purposes of private research and study and extracts (or indeed, the full report) may be included in professional journals provided that suitable acknowledgement is made and the reproduction is not associated with any form of advertising. Applications for commercial reproduction should be addressed to: NIHR Journals Library, National Institute for Health Research, Evaluation, Trials and Studies Coordinating Centre, Alpha House, University of Southampton Science Park, Southampton SO16 7NS, UK.

For peer review only - http://bmjopen.bmj.com/site/about/guidelines.xhtml

Characteristics of included studies

The characteristics of the included studies are summarised in *Tables 1* and *2*. Briefly, 10 primary studies were included in this update.[17,22–30] One was published before 2005[24] but was included here as it had been missed by the original 2007 HTA report.[12] The other nine studies were published in the period 2005–2014.[17,22,23,25–30] Four of them were conducted in China[22,23,27,30] and one in each of the following countries: Brazil,[25] Greece,[29] Japan,[26] Kenya,[24] the Philippines[17] and the USA.[28]

TABLE 1 Study characteristics: sampling and participants

Source and country	Sampling and inclusion criteria	Exclusion criteria	Number of children (% male)	Mean age in years, (SD) or range
Bu *et al.*,[22] 2005, China	Convenience sample of children grade 1–6 studying at one primary school	No exclusion criteria (except for a lack of consent)	317 (49.5)	9.43 (1.79)
Georgalas *et al.*,[29] 2008, Greece	Convenience sample of primary school children (6–12 years old) in one geographical area	No exclusion criteria (except for a lack of consent)	86 (52.0)	8.7 (2.0)
Gloria-Cruz *et al.*,[17] 2013, Philippines	Convenience sample of grade one elementary school children from three metropolitan schools	No exclusion criteria (except for a lack of consent)	418 (56.9)	8.6 (n/a)
Li *et al.*,[23] 2009, China	Convenience sample of children grade 1–6 studying at one rural school	No exclusion criteria (except for a lack of consent)	154 (n/a)	9.3 (1.7)
McPherson *et al.*,[30] 2010, China	Convenience sample of children 6–8 years old from a mainstream urban primary school	No exclusion criteria (except for a lack of consent) but no children with known cognitive or hearing impairments took part	80 (62.5)	7.04 (0.74)
Newton *et al.*,[24] 2001, Kenya	Convenience sample of children attending nursery schools and child health clinics in districts with audiologically trained ENT officers	No exclusion criteria	735 (49.1)	5.2 (n/a); range 2.21–7.5
Samelli *et al.*,[25] 2011, Brazil	Convenience sample of children 2–10 years old living in an underserved metropolitan area with no UNHS	None specified	214 (59.3)	Range 2–10
Soares *et al.*,[26] 2014, Japan	Convenience sample of children 3–5 years old from a mainstream kindergarten (unclear if children from the nearby special school for hearing impaired children were also included in this age group)	Children unable to lie down still for several minutes (unable to complete procedure); lack of consent	115 (n/a)	Range 3–5
Wu *et al.*, 2014,[27] China	Random sample (5%, randomisation procedure not described) of 6288 eligible children enrolled from 41 kindergartens from a district area with a total of 106 kindergartens	Children who refused to participate or had learning disabilities	312 (52.2)	5.06 (0.72)
Yin *et al.*, 2009,[28] USA	Convenience sample of children 2–6 years old who are socioeconomically at risk (14% special education students) attending preschools in a large, urban, metropolitan school district	No exclusion criteria specified (except for the lack of consent) but no children previously identified with hearing impairment took part	135 (n/a)	Range 2–6

ENT, ear nose and throat; n/a, not available; SD, standard deviation.

For peer review only

For peer review only - http://bmjopen.bmj.com/site/about/guidelines.xhtml

DOI: 10.3310/hta20360 HEALTH TECHNOLOGY ASSESSMENT 2016 VOL. 20 NO. 36

TABLE 2 Study characteristics: index test and reference standard

Source and country	Index test(s)	Setting (index test)	Test administrator (index test)	Cut-off (index test)	Reference standard	Setting (reference standard)	Test administrator (reference standard)	Definition of hearing impairment
Bu *et al.*, 2005,[22] China	Questionnaire (CHQS)	Home	Parents/carers	No prespecified cut-off	Otoscopy, tympanometry and PTA	Same as TEOAE	Otolaryngologists, audiologists and nurses	Tympanogram type 'B' or an average threshold across four frequencies (0.5, 1, 2 and 4 kHz) > 20 dB HL in either ear
	TEOAE (Madsen Celesta 503 connected to a laptop)	School; quiet but not sound-treated room during normal attendance hours; ambient noise monitored	Otolaryngologists, audiologists and nurses	Pass: SNR values (an average of 1.5–4 kHz) of at least 3 dB and whole-wave reproducibility of at least 50%				
Georgalas *et al.*,[29] 2008, Greece	TEOAE (ILO 92 recorder). (Also, in combination with tympanometry but results not reported)	School; partially sound-proofed rooms	Otolaryngologists	Pass: if the TEOAE spectrum was recorded at least 3 dB above the noise floor and halfway across the frequency bands of 2–3 kHz and 3–4 kHz	Otoscopy, tympanometry and PTA	Same as for the index test	Same as for the index test	Average threshold > 25 dB across 0.5, 1, 2 and 4 kHz in either ear (> 30 dB also used but no full data reported)
Gloria-Cruz *et al.*,[17] 2013, Philippines	Audiometry (Siemens HC Navigator)	School; quiet but not sound-treated room; ambient noise monitored	Not specified but probably an audiologist	Pass: green light; refer: yellow or red light (separate results for red and yellow were also reported)	PTA	Same as for the index test	Audiologist	> 40 dB at 0.5, 1, 2 or 4 kHz in either ear
Li *et al.*[23] 2009, China	Questionnaire (CHQS-II)	Home	Parents/carers	No prespecified cut-off	Otoscopy, tympanometry and PTA	School; quiet but not sound-treated room during normal attendance hours; ambient noise monitored	Medical doctors and audiologists, under the supervision of an otolaryngologist	Evidence of OME or 'B'-type tympanogram or threshold > 40 dB at 0.5, 1, 2 or 4 kHz in either ear
McPherson *et al.*,[30] 2010, China	Audiometry (Home Audiometer Software version 1.83)	School; quiet but not sound-treated room during non-attendance days; ambient noise monitored	Speech and hearing science undergraduates who had received 12 hours' training	Refer: > 40 dB at 0.5, 1, 2 or 4 kHz in either ear; results also reported after excluding 0.5 kHz data	PTA	Same as for the index test	Same as for the index test (test administrators randomly assigned each time)	> 40 dB HL at 0.5, 1, 2 or 4 kHz in either ear

continued

© Queen's Printer and Controller of HMSO 2016. This work was produced by Fortnum *et al.* under the terms of a commissioning contract issued by the Secretary of State for Health. This issue may be freely reproduced for the purposes of private research and study and extracts (or indeed, the full report) may be included in professional journals provided that suitable acknowledgement is made and the reproduction is not associated with any form of advertising. Applications for commercial reproduction should be addressed to: NIHR Journals Library, National Institute for Health Research, Evaluation, Trials and Studies Coordinating Centre, Alpha House, University of Southampton Science Park, Southampton SO16 7NS, UK.

For peer review only - http://bmjopen.bmj.com/site/about/guidelines.xhtml

TABLE 2 Study characteristics: index test and reference standard (*continued*)

Source and country	Index test(s)	Setting (index test)	Test administrator (index test)	Cut-off (index test)	Reference standard	Setting (reference standard)	Test administrator (reference standard)	Definition of hearing impairment
Newton *et al.*,[24] 2001, Kenya	Questionnaire	Home, nursery, child health clinic	Three modes: (1) a nursery teacher interviewing parents; (2) parents/carers; (3) a community nurse interviewing parents	Refer: one or more answers suggesting hearing impairment or uncertainty (question 5 excluded from analysis)	ENT examination including otoscopy and PTA	Quietest possible room (not sound treated); ambient noise measured whenever possible	ENT officers trained to perform PTA	Average threshold (0.5, 1 and 4 kHz) > 40 dB bilaterally
Samelli *et al.*,[25] 2011, Brazil	Questionnaire	Home, kindergarten, school or health unit	Evaluators (unspecified)	Refer: score ≥ 6 (ROC optimised); two additional cut-offs established to distinguish between conductive and sensorineural hearing impairment	Otoscopy, tympanometry and PTA	Sound-attenuated testing room	Evaluators (unspecified)	> 15 dB HL (0.25–8 kHz), tympanogram type B, C, As, or Ad and/or an absence of acoustic reflexes with one or both ears
Soares *et al.*,[26] 2014, Japan	AABR (MB11 BERA-phone®, MAICO Diagnostic GmbH, Berlin, Germany)	Kindergarten; quiet room (no further details); ambient noise monitored	Audiologist	Pass: a 'pass' line on the device's graphic screen; refer: when 180 seconds elapsed without achieving pass line	PTA (preceded by ENT examination)	Same as for the index test	Audiologist from the institution in which the children belonged	> 25 dB HL at frequencies between 0.5 and 4 kHz
Wu *et al.*,[27] 2014, China	Audiometry (Smart Hearing)	School; quiet but not sound-treated room; ambient noise measured	Screening personnel (unspecified) who had some training	Refer: > 30 dB HL at 1, 2 and 4 kHz in either ear (plus children who failed the guidance stage)	Otoscopy, tympanometry, PTA (standard or play) and distortion product OAEs	Children's Hearing and Speech Centre	Specialists at the centre (unspecified)	Hearing impairment: mild (26–40 dB HL), moderate (41–60 dB HL), severe (61–80 dB HL), or profound (> 80 dB HL) based on the average value of threshold at 0.5, 1, 2 and 4 kHz

For peer review only

For peer review only - http://bmjopen.bmj.com/site/about/guidelines.xhtml

DOI: 10.3310/hta20360 HEALTH TECHNOLOGY ASSESSMENT 2016 VOL. 20 NO. 36

Source and country	Index test(s)	Setting (index test)	Test administrator (index test)	Cut-off (index test)	Reference standard	Setting (reference standard)	Test administrator (reference standard)	Definition of hearing impairment
Yin *et al.*,[28] 2009, USA	TEOAE (Otodynamics Echo Port ILO 288)	Preschool; quiet but not sound-treated room	Nurses and a paediatrician who had 1 hour of 'hands-on' training	Pass: automatically indicated when a TEOAE response was obtained for three of five frequency range with TEOAE being 5 dB above noise floor<br><br>Refer: no TEOAE present or did not pass the required number of frequencies	PTA	Unspecified	School audiologists	No response at any frequency (1, 2, 4 kHz) to 25 dB HL pure tone

AABR, automated auditory brainstem response; CHQS, Chinese Hearing Questionnaire for School Children; ENT, ear, nose and throat; SNR, signal-to-noise ratio; TEOAE, transient-evoked otoacoustic emission.

© Queen's Printer and Controller of HMSO 2016. This work was produced by Fortnum *et al.* under the terms of a commissioning contract issued by the Secretary of State for Health. This issue may be freely reproduced for the purposes of private research and study and extracts (or indeed, the full report) may be included in professional journals provided that suitable acknowledgement is made and the reproduction is not associated with any form of advertising. Applications for commercial reproduction should be addressed to: NIHR Journals Library, National Institute for Health Research, Evaluation, Trials and Studies Coordinating Centre, Alpha House, University of Southampton Science Park, Southampton SO16 7NS, UK.

For peer review only - http://bmjopen.bmj.com/site/about/guidelines.xhtml

Although the majority of the included children were at or around school entry age, there was marked variability in terms of age range. Children's age ranged from 2[25,28] to 13 years[23] with the mean age ranging from 5.1 years [standard deviation (SD) 0.7 years][27] to 9.4 years (SD 1.8 years)[22] (three studies[25–27] did not report mean age and only gave age range as an inclusion criterion). The studies were relatively balanced in terms of participants' gender, the proportion of males ranging from 48.4%[26] to 62.5%[30] (two papers[23,28] did not report details). Apart from lack of consent, most studies did not specify any other exclusion criteria. One study[26] excluded children who were unable to lie down still for several minutes, which was a necessary requirement for completing the screen procedure; one study[27] excluded children with learning disabilities and two studies[26,30] commented that no children with known cognitive or hearing impairments were included but it was unclear if this had been a prespecified exclusion criterion. Seven studies[17,22–25,27,30] were conducted in countries without established UNHS.

For peer review only

Studies evaluated the performance of questionnaires ($n = 4$), audiometry ($n = 3$), TEOAE ($n = 3$) and automated auditory brainstem response (AABR) ($n = 1$). AABR is usually used to screen infants and was not listed in the initial inclusion criteria. However, we decided to include the study, as it evaluated, probably for the first time, the use of AABR in SES. Two studies[22,23] evaluated different versions of the Chinese Hearing Questionnaire for School Children (CHQS). Only one study[22] compared directly the performance of two different tests, a questionnaire and TEOAE, and one study[29] evaluated a combination of TEOAE and tympanometry but did not report the results as adding tympanometry had not improved performance.

Questionnaires were completed by parents or carers in two studies,[22,23] either by parents/carers or by teachers or community nurses interviewing parents (three different testing conditions) in one study[24] and by unspecified evaluators in one study.[25] All other index tests were performed either by audiologists, otolaryngologists or other professional staff who had some preliminary training. With the exception of questionnaires, all other tests were performed at school or a community health centre in a quiet but not sound-treated room and the ambient noise level was monitored.

All studies except those evaluating questionnaires used a prespecified positivity threshold to define 'pass' and 'refer' outcomes. Data-driven selection of a threshold to achieve optimal performance (best sensitivity and/or best specificity) could lead to overly optimistic diagnostic accuracy estimates and the test is likely to perform worse when the same threshold is used in an independent sample of patients.[31]

The reference standard was a combination of otoscopy, tympanometry and audiometry in four studies,[22,23,25,29] otoscopy, tympanometry, audiometry and OAEs in one study,[27] ear, nose and throat (ENT) examination including audiometry in two studies,[24,26] and audiometry only in three studies.[17,28,30] The tests were performed by audiologists and otolaryngologists, usually in quiet but not sound-treated rooms with monitored ambient noise.

The mean prevalence of the target condition across all studies that reported outcomes at the level of the individual was 10.8% (SD 12.6%) and ranged from 0.7%[28] to 46.7%.[25] The prevalence in the only study that reported ear-level outcomes was 4.8%.[17] These numbers should, however, be treated with caution, as the studies varied in their pass/refer criteria and included convenience samples drawn from populations likely to be different in terms of prevalence and spectrum of hearing impairments.

For peer review only - http://bmjopen.bmj.com/site/about/guidelines.xhtml

DOI: 10.3310/hta20360 HEALTH TECHNOLOGY ASSESSMENT 2016 VOL. 20 NO. 36

Methodological quality of included studies

The definitions of the assessment criteria are given in *Appendix 2* and the results from the methodological quality assessment are summarised in *Table 3*. All studies had a prospective cross-sectional single-gate design. 'Single-gate' is a term introduced by Rutjes *et al.*[32] to describe diagnostic accuracy studies in which a single sample drawn from the target population receives the index test and the reference standard to allow calculation of the test's sensitivity and specificity. In contrast, in 'two-gate' designs sensitivity and specificity are calculated separately based on two different samples of participants, that is, one with and one without the target condition. The two-gate design is prone to spectrum bias as the mix of participants in the two samples combined is unlikely to be representative of the target population.[32] Therefore, studies using a single-gate design are considered to be of better methodological quality.

Although all included studies had a single-gate design, none of them included a representative sample of a relevant target population defined for the purpose of this review as 4- to 6-year-old children (± 1 year) at or around school entry stage who have no high-risk features (such as Down syndrome, cytomegalovirus infection or meningitis) and have been tested and found to have no hearing impairment at birth. Therefore, the studies are likely to suffer from a selection bias and to have limited applicability to the UK context because of the following methodological issues. First, some studies included children younger or older than the defined 4–6 years age range, which reflects, to some extent, the fact that school entry age varies across countries. Second, seven of the studies[17,22–25,27,30] were conducted in countries without established UNHS, which is likely to impact on the prevalence and spectrum of hearing impairments in the included children. Third, most of the studies included small self-selected samples recruited from a single locality and, therefore, may not be representative, even when drawn from a relevant target population.

The execution of the index test(s) and the reference standard were reported in sufficient detail in the majority of the studies. In five studies,[17,24,26,28,30] however, the reference standard was suboptimal and did not meet the quality criterion 'PTA + tympanometry'. The time between the performance of the index test and the reference standard was < 1 month in four studies,[17,22,23,30] was > 1 month in one study[28] and was not reported in five studies.[24–27,29] Blinding of the index test evaluators to the results of the reference standard was not reported in three studies[22,26,29] and blinding of the reference standard evaluators to the results of the index test was not reported in five studies.[22,23,26,27,29] Those criteria that were consistently met across the studies were questions 5–9 (see *Appendix 2*) concerning the application of the same reference standard to the whole or random sample, regardless of the index test result (verification bias); the independence of the reference standard from the index test (incorporation bias) and the description of index test and reference standard in sufficient detail to allow replication. According to the criteria for calculating a total quality score published in the 2007 HTA report,[12] three studies[25,26,29] were of 'moderate' quality (total score 7–9) and the remaining seven studies[17,22–24,27,28,30] were of 'good' quality (total score of > 9).

Test accuracy

Given the significant heterogeneity in the study characteristics and the reported test accuracy estimates (see *Table 2* and *Figure 3*) we considered quantitative synthesis inappropriate and, instead, summarised the performance of different test types in tables and figures (*Figures 3* and *4*, and *Table 4*).

Parental questionnaires

Four studies[22–25] reported the diagnostic accuracy of questionnaires, two of which evaluated different versions of the same tool (CHQS).[22,23] All questionnaires had been independently validated and the authors reported satisfactory reliability. None of the studies used a prespecified positivity threshold, which means that the reported diagnostic accuracy might be exaggerated. The two studies evaluating CHQS reported results from a range of different scores[23] or a range of sensitivity and specificity values obtained for different individual questions and combinations.[22] The results included in the forest plot and the ROC curve plot (see *Figures 3* and *4*) for these two studies correspond to positivity thresholds that resulted in the best overall performance: a total score of > 1 for the study by Li *et al.*,[23] and sensitivity of 56% and specificity of 60% read-off the ROC plot in the paper for the study by Bu *et al.*[22]

© Queen's Printer and Controller of HMSO 2016. This work was produced by Fortnum *et al.* under the terms of a commissioning contract issued by the Secretary of State for Health. This issue may be freely reproduced for the purposes of private research and study and extracts (or indeed, the full report) may be included in professional journals provided that suitable acknowledgement is made and the reproduction is not associated with any form of advertising. Applications for commercial reproduction should be addressed to: NIHR Journals Library, National Institute for Health Research, Evaluation, Trials and Studies Coordinating Centre, Alpha House, University of Southampton Science Park, Southampton SO16 7NS, UK.

For peer review only - http://bmjopen.bmj.com/site/about/guidelines.xhtml

TABLE 3 Results from the methodological quality assessment

	Study	Representative spectrum?	Selection criteria described?	Acceptable reference standard?	Acceptable time between tests?	Whole/random sample received the reference standard?	Same reference standard?	Reference standard and index test independent?
Study 1	Bu *et al.*,[22] 2005	No	Yes	Yes	Yes	Yes	Yes	Yes
Study 2	Georgalas *et al.*,[29] 2008	No	No	Yes	Not clear	Yes	Yes	Yes
Study 3	Gloria-Cruz *et al.*,[17] 2013	No	Yes	No	Yes	Yes	Yes	Yes
Study 4	Li *et al.*,[23] 2009	No	Yes	Yes	Yes	Yes	Yes	Yes
Study 5	McPherson *et al.*,[30] 2010	No	Yes	No	Yes	Yes	Yes	Yes
Study 6	Newton *et al.*,[24] 2001	No	Yes	No	Not clear	Yes	Yes	Yes
Study 7	Samelli *et al.*,[25] 2011	No	No	Yes	Not clear	Yes	Yes	Yes
Study 8	Soares *et al.*,[26] 2014	No	No	No	Not clear	Yes	Yes	Yes
Study 9	Wu *et al.*,[27] 2014	No	Yes	Yes	Not clear	Yes	Yes	Yes
Study 10	Yin *et al.*,[28] 2009	No	Yes	No	No	Yes	Yes	Yes

For peer review only

For peer review only - http://bmjopen.bmj.com/site/about/guidelines.xhtml

DOI: 10.3310/hta20360 HEALTH TECHNOLOGY ASSESSMENT 2016 VOL. 20 NO. 36

Index test described?	Reference standard described?	Blinded interpretation of index test?	Blinded interpretation of reference standard?	Same clinical data available as in practice?	Uninterpretable/ intermediate results reported?	Withdrawals explained	Total score (number of 'yes')	
Yes	Yes	Not clear	Not clear	Not clear	Yes	Yes	10	Study 1
Yes	Yes	Not clear	Not clear	Not clear	Yes	Yes	8	Study 2
Yes	Yes	Yes	Yes	Not clear	Yes	Yes	11	Study 3
Yes	Yes	Yes	Not clear	Yes	Yes	Yes	12	Study 4
Yes	Yes	Yes	Yes	Yes	Not clear	Not clear	10	Study 5
Yes	Yes	Yes	Yes	Yes	Yes	Yes	11	Study 6
Yes	Yes	Yes	Yes	Yes	Not clear	Not clear	9	Study 7
Yes	Yes	Not clear	Not clear	Not clear	Yes	Yes	7	Study 8
Yes	Yes	Yes	Not clear	Yes	Yes	Yes	11	Study 9
Yes	Yes	Yes	Yes	Yes	Yes	Yes	11	Study 10

For peer review only

© Queen's Printer and Controller of HMSO 2016. This work was produced by Fortnum *et al.* under the terms of a commissioning contract issued by the Secretary of State for Health. This issue may be freely reproduced for the purposes of private research and study and extracts (or indeed, the full report) may be included in professional journals provided that suitable acknowledgement is made and the reproduction is not associated with any form of advertising. Applications for commercial reproduction should be addressed to: NIHR Journals Library, National Institute for Health Research, Evaluation, Trials and Studies Coordinating Centre, Alpha House, University of Southampton Science Park, Southampton SO16 7NS, UK.

For peer review only - http://bmjopen.bmj.com/site/about/guidelines.xhtml

Questionnaire

Study	TP	FP	FN	TN	Sensitivity (95% CI)	Specificity (95% CI)
Bu 2005[22]	9	120	7	181	0.56 (0.30 to 0.80)	0.60 (0.54 to 0.66)
Li 2009[23]	10	63	5	76	0.67 (0.38 to 0.88)	0.55 (0.46 to 0.63)
Newton 2001[24]	13	182	0	540	1.00 (0.75 to 1.00)	0.75 (0.71 to 0.78)
Samellie 2011[25]	44	15	56	99	0.44 (0.34 to 0.54)	0.87 (0.79 to 0.92)

Audiometry

Study	TP	FP	FN	TN	Sensitivity (95% CI)	Specificity (95% CI)
Gloria-Cruz 2013[17]	9	20	30	762	0.23 (0.11 to 0.39)	0.97 (0.96 to 0.98)
McPherson 2010[30]	7	6	2	65	0.78 (0.40 to 0.97)	0.92 (0.83 to 0.97)
Wu 2014[27]	6	22	10	274	0.38 (0.15 to 0.65)	0.93 (0.89 to 0.95)

TEOAE

Study	TP	FP	FN	TN	Sensitivity (95% CI)	Specificity (95% CI)
Bu 2005[22]	12	6	4	295	0.75 (0.48 to 0.93)	0.98 (0.96 to 0.99)
Georgalas 2008[29]	9	27	1	49	0.90 (0.55 to 1.00)	0.64 (0.53 to 0.75)
Yin 2009[28]	1	8	0	126	1.00 (0.03 to 1.00)	0.94 (0.89 to 0.97)

AABR

Study	TP	FP	FN	TN	Sensitivity (95% CI)	Specificity (95% CI)
Soares 2014[26]	11	6	0	98	1.00 (0.72 to 1.00)	0.94 (0.88 to 0.98)

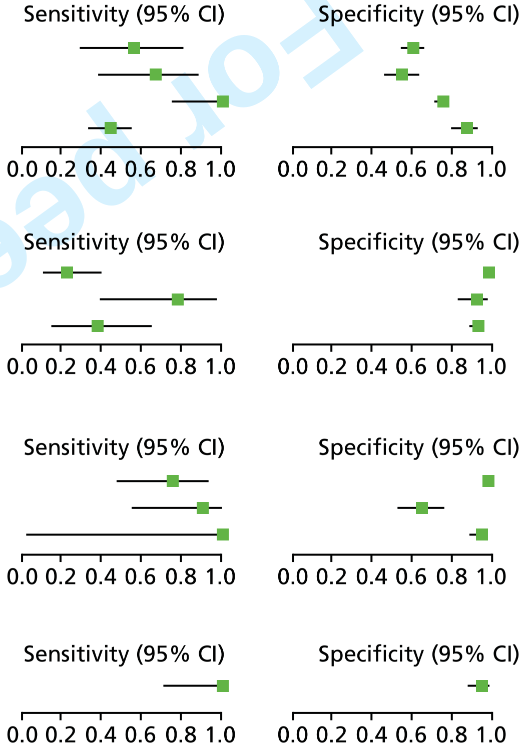

FIGURE 3 Forest plot of sensitivity and specificity of different types of hearing screening tests. Please note that the results for Bu *et al.*[22] are based on reading off the ROC plot in the paper and do not refer to a particular positivity threshold; the results for Li *et al.*[23] are based on '1+' answers suggesting hearing impairment; and the results for McPherson *et al.*[30] are based on the data set that did not include results from 0.5 kHz. CI, confidence interval; FN, false negative; FP, false positive; TN, true negative; TP, true positive.

NIHR Journals Library www.journalslibrary.nihr.ac.uk
For peer review only - http://bmjopen.bmj.com/site/about/guidelines.xhtml

DOI: 10.3310/hta20360 HEALTH TECHNOLOGY ASSESSMENT 2016 VOL. 20 NO. 36

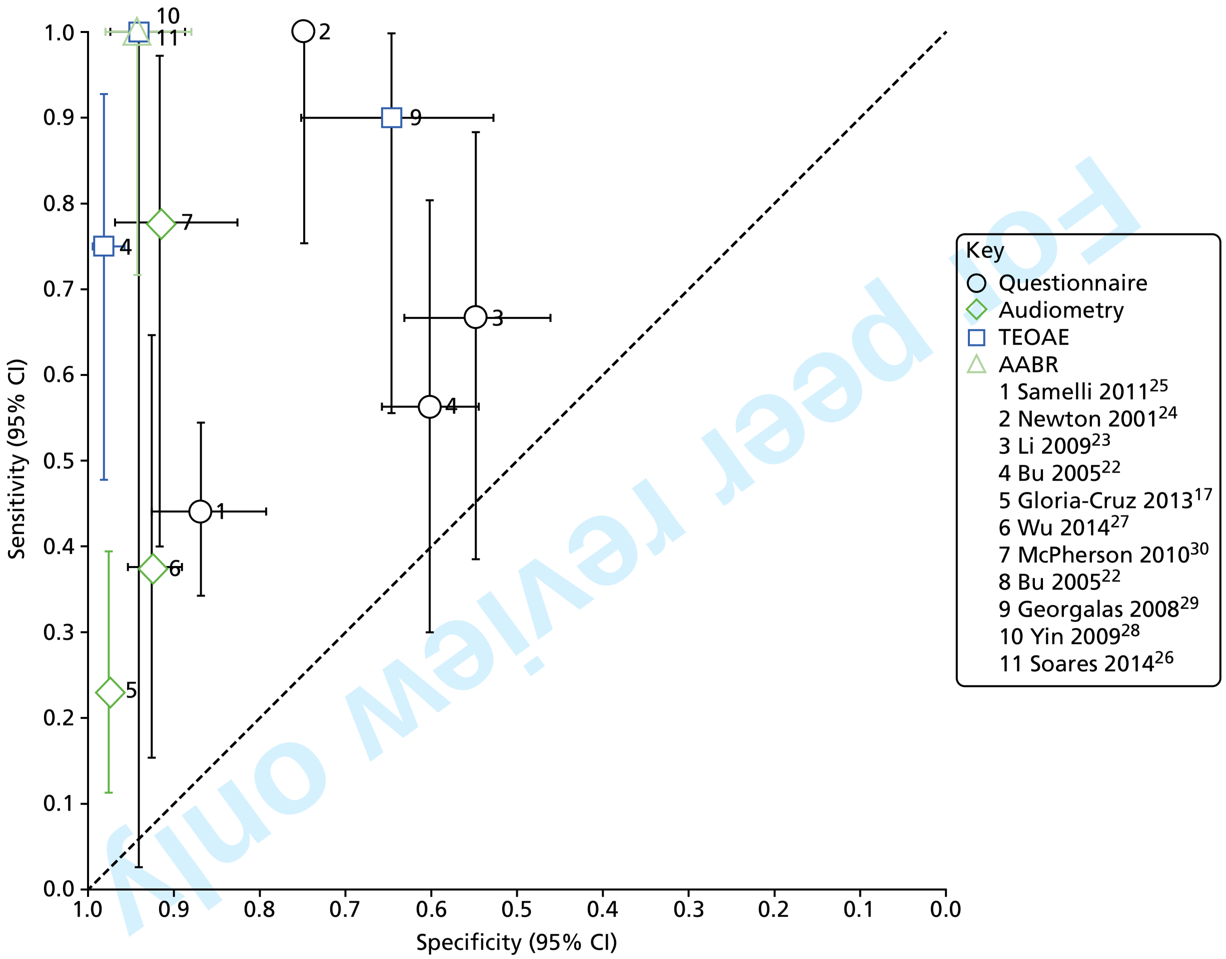

FIGURE 4 Summary ROC plot of different hearing screening tests. Please note that the results for Bu *et al.*[22] are based on reading off the ROC plot in the paper and do not refer to a particular positivity threshold; the results for Li *et al.*[23] are based on '1+' answers suggesting hearing impairment; and the results for McPherson *et al.*[30] are based on the data set that did not include results from 0.5 kHz. CI, confidence interval.

© Queen's Printer and Controller of HMSO 2016. This work was produced by Fortnum *et al.* under the terms of a commissioning contract issued by the Secretary of State for Health. This issue may be freely reproduced for the purposes of private research and study and extracts (or indeed, the full report) may be included in professional journals provided that suitable acknowledgement is made and the reproduction is not associated with any form of advertising. Applications for commercial reproduction should be addressed to: NIHR Journals Library, National Institute for Health Research, Evaluation, Trials and Studies Coordinating Centre, Alpha House, University of Southampton Science Park, Southampton SO16 7NS, UK.

For peer review only - http://bmjopen.bmj.com/site/about/guidelines.xhtml

TABLE 4 Diagnostic accuracy outcomes

Study and country	Index test cut-off	Total number	TP	FP	FN	TN	Prevalence %	Sensitivity % (95% CI)	Specificity % (95% CI)	Withdrawals and uninterpretable results
Questionnaire										
Bu *et al.*,[22] 2005, China	Single questions	317	n/a	n/a	n/a	n/a	5.05	Range 7–42	Range 76–99	None reported; response rate to questionnaire was 61%
	Unspecified cut-off (based on the ROC plot reported in the paper)	317	9	120	7	181	5.05	56 (30 to 80)	60 (54 to 66)	As above
Li *et al.*[23] 2009, China	Scores of ≥ 1 to ≥ 5	154	n/a	n/a	n/a	n/a	9.74	Range 0–67	Range 55–100	None reported; questionnaire's response rate was 100% (after being distributed via students randomly selected by teachers)
	Score of ≥ 1	154	10	63	5	76	9.74	0.67 (0.38 to 0.88)	0.55 (0.46 to 0.63)	As above
Newton *et al.*,[24] 2001, Kenya	Score of ≥ 1 (question 5 excluded)	735	13	182	0	540	1.77	100 (75 to 100)	75 (71 to 78)	Response rate to questionnaire was 88%; PTA could not be obtained for 22 children who were excluded from analysis
Samelli *et al.*,[25] 2011, Brazil	Score of ≥ 6	214	44	15	56	99	46.72	44 (34 to 54)	87 (79 to 92)	None reported; response rate also not reported
Audiometry										
Gloria-Cruz *et al.*,[17] 2013, Philippines	Pass: green; refer: yellow/red	821 (ears)	9	20	30	762	4.75 (ears)	23 (11 to 39)	97 (96 to 98)	73% coverage of the entire population under study: 7 out of 418 children excluded as not available for testing; one child had only one ear tested
McPherson *et al.*,[30] 2010, China	> 40 dB any frequency	80	10	35	0	35	12.5	100 (69 to 100)	50 (38 to 62)	None reported; coverage of entitled children also not reported
	> 40 dB, 0.5 kHz excluded	80	7	6	2	65	11.25	78 (40 to 97)	92 (83 to 97)	
Wu *et al.*,[27] 2014, China	> 30 dB at 1, 2 or 4 kHz	312	6	22	10	274	5.13	38 (15 to 65)	93 (89 to 95)	76 children refused to take part; eight children with learning disabilities were excluded

For peer review only - http://bmjopen.bmj.com/site/about/guidelines.xhtml

For peer review only

DOI: 10.3310/hta20360 HEALTH TECHNOLOGY ASSESSMENT 2016 VOL. 20 NO. 36

Study and country	Index test cut-off	Total number	TP	FP	FN	TN	Prevalence %	Sensitivity % (95% CI)	Specificity % (95% CI)	Withdrawals and uninterpretable results
TEOAE										
Bu *et al.*,[22] 2005, China	See *Table 2*	317	12	6	4	295	5.05	75 (48 to 93)	98 (96 to 99)	As above
Georgalas *et al.*,[29] 2008, Greece	See *Table 2*	86	9	27	1	49	11.63	90 (55 to 100)	64 (53 to 75)	196 students enrolled but only 86 received PTA owing to financial constraints (selection criteria not clear)
Yin *et al.*,[28] 2009, USA	See *Table 2*	135	1	8	0	126	74	100 (3 to 100)	94 (89 to 97)	142 students enrolled in the diagnostic cohort of whom seven were excluded (two special education students refused TEOAE, three non-special education refused or were unable to do PTA, two students could not be tested using TEOAE owing to complete cerumen impaction)
AABR										
Soares *et al.*,[26] 2014, Japan	n/a	115	11	6	0	98	9.57	100 (72 to 100)	94 (88 to 98)	17 individuals (34 ears) excluded from the sample owing to failure to get good impedance or accurate PTA; and five ears (from the 163 included individuals) were also excluded

CI, confidence interval; FN, false negative; FP, false positive; n/a, not available; TN, true negative; TP, true positive.

© Queen's Printer and Controller of HMSO 2016. This work was produced by Fortnum *et al.* under the terms of a commissioning contract issued by the Secretary of State for Health. This issue may be freely reproduced for the purposes of private research and study and extracts (or indeed, the full report) may be included in professional journals provided that suitable acknowledgement is made and the reproduction is not associated with any form of advertising. Applications for commercial reproduction should be addressed to: NIHR Journals Library, National Institute for Health Research, Evaluation, Trials and Studies Coordinating Centre, Alpha House, University of Southampton Science Park, Southampton SO16 7NS, UK.

For peer review only - http://bmjopen.bmj.com/site/about/guidelines.xhtml

Across all studies, sensitivity ranged from 44% to 100% and specificity from 55% to 87% (see *Figure 3*). The ROC plot in *Figure 4* clearly shows that the performance of the questionnaires was poor, with three of the studies,[22,23,25] close to the line of no effect (the dotted diagonal line), which indicates accuracy no better than expected due to chance. Only the study conducted by Newton *et al.*[24] showed relatively good overall accuracy with excellent sensitivity (100%) and moderate specificity (75%). However, the definition of hearing impairment in this study was bilateral hearing impairment of > 40 dB and three of the four children with unilateral hearing impairment of > 40 dB were missed by the questionnaire. Including these children in the analysis brings the sensitivity down to 82% while specificity remains the same (75%).

Audiometry

The diagnostic accuracy of audiometry was evaluated in three studies: two evaluated computer-based audiometers[27,30] and one a hand-held device.[17] Sensitivity ranged from 23% to 78% and specificity from 92% to 97% (see *Figure 3*). McPherson *et al.*[30] reported two sets of results – before and after excluding the data for 0.5 kHz – which they considered problematic owing to possible interference from ambient noise. With the 0.5 kHz data included, sensitivity was 100% and specificity was 50%; excluding 0.5 kHz data led to a marked decrease in sensitivity (78%) and increase in specificity (92%). The forest plot and the ROC plot (see *Figures 3* and *4*) show that, based on the studies included here, portable audiometry tools have much better and more consistent specificity, but variable sensitivity, noting, however, that there is a considerable difference between the technologies evaluated, particularly between the computer-based audiometers and the hand-held HC screening device.

Transient-evoked otoacoustic emissions

Three studies[22,28,29] evaluated the accuracy of TEOAE, one of which compared its performance with that of a questionnaire (see *Figures 3* and *4*).[22] Sensitivity ranged from 75% to 100% and specificity from 64% to 98%. However, the sensitivity estimate reported by Yin *et al.*[28] had a very wide confidence interval (CI) (3% to 100%), indicating considerable statistical uncertainty. The comparative study found that the diagnostic accuracy of TEOAE was superior to that of the questionnaire with sensitivity of 75% versus 56% and specificity of 98% versus 60%. The sensitivity estimates of the two tests, however, had overlapping CIs, suggesting that the result might be due to chance rather than real superiority.

Automated auditory brainstem response

Only one study[26] evaluated the diagnostic accuracy of AABR as a screening test for school entry children. This study was conducted in Japan, included children 3–5 years old and the evaluated device, MB11 BERA-phone® (MAICO Diagnostic GmbH, Berlin, Germany), was operated by an audiologist. The study had the lowest quality score of all included studies (see *Table 3*) because of a failure to provide a clear description of the selection process and to report important aspects of the study design, such as time between index test and reference standard, blinding and availability of clinical data to test administrators. The test showed very high performance with 100% sensitivity and 94% specificity but the sensitivity estimate had a relatively wide 95% CI (72% to 100%) owing to a small sample size and a low event rate.

Discussion

Summary of the findings from the 2007 Health Technology Assessment report

The 2007 HTA report[12] included 25 primary studies reporting the performance of a wide range of hearing screening tests, including parental questionnaires, impedance audiometry/tympanometry, spoken word tests, otoscopy, audiometry, TEOAE and other tests and combinations of tests. The overall conclusion was that evidence was of unacceptable variability in terms of methodological quality and study characteristics and, as a result, drawing strong conclusions about the performance of different tests was not possible.

For peer review only - http://bmjopen.bmj.com/site/about/guidelines.xhtml

DOI: 10.3310/hta20360 HEALTH TECHNOLOGY ASSESSMENT 2016 VOL. 20 NO. 36

With those caveats taken into account and including only the subset of studies that used PTA as a reference standard, the findings from the 2007 HTA report suggested that: sweep PTA had high sensitivity and specificity; spoken word tests had acceptable levels of sensitivity and specificity; TEOAE had high specificity but somewhat lower sensitivity; tympanometry and acoustic reflectometry had variable sensitivity and specificity; parental questionnaires and otoscopy had poor sensitivity and specificity; and there was insufficient evidence to comment on the accuracy of combinations of tests (p. 48).

Combining the results from the 2007 Health Technology Assessment report and the current update

The studies identified in the current update provide additional test accuracy data for the following three categories of tests evaluated in the original HTA review: parental questionnaires, sweep PTA tests and TEOAE. Below we discuss the combined evidence from the two sets of studies for each category of tests.

Parental questionnaires

In the 2007 HTA report[12] three studies[33–35] examined the accuracy of parental questionnaires and reported sensitivities ranging from 34% to 71% and specificities ranging from 52% to 95%. In the update we included four studies[22–25] evaluating questionnaires that reported sensitivities and specificities in the range of 44–100% and 55–87%, respectively (*Table 5*). As none of them used a prespecified positivity threshold, the reported test accuracy estimates are likely to be overoptimistic and the performance of the evaluated questionnaires worse when applied in practice. The study by Newton *et al.*[24] reported very high sensitivity but the definition of hearing impairment was very restrictive and may not be appropriate for most circumstances. Taking into account the methodological limitations of the studies and the marked heterogeneity in their results, the combined evidence from the original 2007 HTA report and the current update suggest that, on the whole, parental questionnaires have poor diagnostic accuracy and may not be suitable for mass screening, especially in the context of established UNHS and sensitised educational and health-care systems.

Audiometry-based tests

Five evaluations reported in four studies[36–39] included in the original 2007 HTA report and three[17,27,30] in the current update evaluated the accuracy of audiometry-based tests. The original five studies, all assessing pure-tone sweep devices, reported sensitivities ranging from 86% to 100% and specificities from 65% to 99%. The three studies in the update reported sensitivities in the range of 23% to 78% and specificities 92% to 97% (*Table 6*). McPherson *et al.*,[30] achieved high specificity (92%) only after the results from the 0.5 kHz frequency were excluded from the analysis. This was a post-hoc decision made to reduce the interference from background noise. With this caveat noted, the new studies reported higher and more consistent specificity but lower and widely varying sensitivity estimates compared with the

TABLE 5 Sensitivities and specificities of all studies evaluating questionnaires and included in the 2007 HTA report[12] and the current update

Report	Study	Sensitivity, % (95% CI)	Specificity, % (95% CI)
Current update	Bu *et al.*, 2005[22]	56 (30 to 80)	60 (54 to 66)
	Li *et al.*, 2009[23]	67 (38 to 88)	55 (46 to 63)
	Newton *et al.*, 2001[24]	100 (75 to 100)	75 (71 to 78)
	Samelli *et al.*, 2011[25]	44 (34 to 54)	87 (79 to 92)
2007 HTA report[12]	Gomes and Lichtig, 2005[33]	71 (n/a)	64 (n/a)
	Olusanya, 2001[34]	34 (n/a)	95 (n/a)
	Hammond *et al.*, 1997[35]	56 (n/a)	52 (n/a)

n/a, not available.

© Queen's Printer and Controller of HMSO 2016. This work was produced by Fortnum *et al.* under the terms of a commissioning contract issued by the Secretary of State for Health. This issue may be freely reproduced for the purposes of private research and study and extracts (or indeed, the full report) may be included in professional journals provided that suitable acknowledgement is made and the reproduction is not associated with any form of advertising. Applications for commercial reproduction should be addressed to: NIHR Journals Library, National Institute for Health Research, Evaluation, Trials and Studies Coordinating Centre, Alpha House, University of Southampton Science Park, Southampton SO16 7NS, UK.

For peer review only - http://bmjopen.bmj.com/site/about/guidelines.xhtml

TABLE 6 Sensitivities and specificities of all studies evaluating audiometry-based tests and included in the 2007 HTA report[12] and the current update

Report	Study	Sensitivity, % (95% CI)	Specificity, % (95% CI)
Current update	Gloria-Cruz *et al.*, 2013[17]	23 (11 to 39)	97 (96 to 98)
	McPherson *et al.*, 2010[30]	78 (40 to 97)[a]	92 (83 to 97)
	Wu *et al.*, 2014[27]	38 (15 to 65)	93 (89 to 95)
2007 HTA report[12]	Sabo *et al.*, 2000[36]	87 (n/a)	80 (n/a)
	Orlando and Frank, 1987[37]	Range 82–100[b]	Range 65–90[b]
	Orlando and Frank, 1987[37]	Range 91–100[b]	Range 97–98[b]
	FitzZaland and Zink, 1984[38]	93 (n/a)	99 (n/a)
	Holtby *et al.*, 1997[39]	86 (n/a)	70 (n/a)

n/a, not available.
a After excluding the results from 0.5 kHz.
b Same study and for different age groups ranging from 4 to 6 years old.

original studies. When combined, the results from the two sets of studies are more difficult to interpret, as there is a marked heterogeneity in both sensitivity and specificity. However, given the gap of > 10 years between the two sets of studies and the technical differences between the evaluated devices, it might be more reasonable to interpret the results from the new studies separately instead of combining them with those from the 2007 HTA review.[12]

Transient-evoked otoacoustic emissions

Only two of the studies included in the original 2007 HTA report[12] evaluated the accuracy of TEOAE.[36,40] The first reported sensitivity of 63% and specificity of 91%, and the second one reported sensitivities ranging from 67% to 100% and specificities from 80% to 98% depending on the 'refer' criterion used. In comparison, three studies[22,28,29] were included in the current update, reporting sensitivities from 75% to 100% and specificities from 64% to 98%. *Table 7* illustrates the heterogeneity in the results. Although the new studies reported higher sensitivities, the CIs were very wide, indicating considerable statistical uncertainty. With the exception of Georgalas *et al.*[29] the specificity estimates across all studies were higher and more consistent, suggesting a relatively low false-positive rate. We could not identify obvious reasons for the low specificity reported by Georgalas *et al.*[29] However, this was a small, poorly reported study with considerable methodological limitations (total quality score = 8) and its results should be interpreted with caution.

TABLE 7 Sensitivities and specificities of all studies evaluating TEOAE and included in the 2007 HTA report[12] and the current update

Report	Study	Sensitivity, % (95% CI)	Specificity, % (95% CI)
Current update	Bu *et al.*, 2005[22]	75 (48 to 93)	98 (96 to 99)
	Georgalas *et al.*, 2008[29]	90 (55 to 100)	64 (53 to 75)
	Yin *et al.*, 2009[28]	100 (3 to 100)	94 (89 to 97)
2007 HTA report[12]	Sabo *et al.*, 2000[36]	63 (n/a)	91 (n/a)
	Nozza *et al.*, 1997[40]	67–100 (n/a)	80–98 (n/a)

n/a, not available.

For peer review only - http://bmjopen.bmj.com/site/about/guidelines.xhtml

DOI: 10.3310/hta20360 HEALTH TECHNOLOGY ASSESSMENT 2016 VOL. 20 NO. 36

Other tests

None of the studies included in the original HTA report assessed the accuracy of AABR and none of the studies included in the update assessed the accuracy of spoken word tests, otoscopy, the audiometric Rinne test and reflectometry. Although one study[29] evaluated the accuracy of tympanometry in combination with TEOAE it reported results only for TEOAE as adding tympanometry had failed to improve performance.

Conclusion

This updated review confirms the conclusion from the 2007 HTA report[12] that, because of a marked variability in the design, methodological quality and the results of the existing studies, it is not possible to draw strong conclusions about the performance of individual test types for use in SES.

Moreover, there were significant differences in the technical characteristics of some of the evaluated devices even when they belonged to the same screening modality (e.g. in the audiometry-based category there were two computer-based devices, one of which involves joysticks, etc., to help children respond, whereas the third one was a hand-held device). This is not surprising given that the studies included in the 2007 HTA and the current update span a period of 30 years. Interpreting their results together, even in the context of a narrative synthesis, requires careful consideration of the differences between the evaluated tests, which, in some cases, might be too great to justify such an approach. Combining the results from more modern devices with those from older ones and making general conclusions based on all the studies included in a particular category may be inappropriate. The performance of currently available tests is of interest to policy-makers.

With these caveats in mind, the findings from the current update, interpreted in the context of the 2007 HTA report,[12] could be summarised as follows:

- We were able to identify a limited number of studies that provided additional diagnostic accuracy data for only the following categories of hearing screening tests: parental questionnaires, audiometry-based tests, TEOAE and AABR. No studies evaluating AABR were included in the 2007 HTA report.[12]
- Questionnaires had the poorest diagnostic accuracy compared with all other tests. The only study that directly compared a questionnaire with another test (TEOAE) supported this finding.[22]
- Audiometry-based tests had high specificity but variable sensitivity.
- Studies evaluating TEOAE reported variable sensitivity with wide CIs, whereas specificity estimates were relatively high and more consistent (with the exception of one study).[29]
- The study evaluating AABR reported high sensitivity and specificity.

The majority of the studies were conducted in countries without an established UNHS system and with variable health-care arrangements and, therefore, the reported results may have limited applicability to the UK context characterised with a well-established UNHS system, sensitised educational system and highly accessible and responsive health care.

For peer review only

© Queen's Printer and Controller of HMSO 2016. This work was produced by Fortnum *et al.* under the terms of a commissioning contract issued by the Secretary of State for Health. This issue may be freely reproduced for the purposes of private research and study and extracts (or indeed, the full report) may be included in professional journals provided that suitable acknowledgement is made and the reproduction is not associated with any form of advertising. Applications for commercial reproduction should be addressed to: NIHR Journals Library, National Institute for Health Research, Evaluation, Trials and Studies Coordinating Centre, Alpha House, University of Southampton Science Park, Southampton SO16 7NS, UK.

For peer review only - http://bmjopen.bmj.com/site/about/guidelines.xhtml

For peer review only

DOI: 10.3310/hta20360 HEALTH TECHNOLOGY ASSESSMENT 2016 VOL. 20 NO. 36

Chapter 3 Diagnostic accuracy of the pure-tone screen and HearCheck screener for identifying hearing impairment in school children

Introduction

In the survey of practice reported in the 2007 HTA report[12] the test used for the hearing screen was in all cases the PTS but there was a wide variety of implementations of this, with different frequencies, pass criteria and retest protocols. Studies concerned with the relative accuracy (in terms of sensitivity and specificity) of alternative screening tests are difficult to compare and often flawed by their use of different referral criteria and case definitions. As reviewed in *Chapter 2*, the 2007 HTA report[12] identified 25 publications reporting comparative trials of alternative screens or tests for screening at school entry. Most studies were undertaken in populations where the prevalence of undetected hearing impairment was considerably greater than that likely to be encountered in a system where a UNHS programme has been introduced. These published data indicate that, using full PTA as the reference standard, the PTS test appears to have high sensitivity and high specificity for minimal, mild and greater hearing impairments; better than alternative tests for which evidence was identified. Spoken word tests were reported with acceptable levels of sensitivity and specificity but are variable in their implementation. OAEs, tympanometry, acoustic reflectometry, parental questionnaires and otoscopy were reported, with either variable or poor accuracy.

A new device, the HC screener, came onto the market in 2005 as a tool for screening for hearing impairment in adults in a general practice setting. It is hand held and has an automatic presentation of a series of tones at two frequencies and three levels: 1 kHz at 55, 35 and 20 dB HL, and 3 kHz at 75, 55 and 35 dB HL. It has potential to be a quicker test in the school setting but it has not previously been assessed as a tool for screening in children.

Objectives

In this chapter we use a two-gate case–control design[32] to:

- estimate the diagnostic accuracy of the PTS and HC tests for discriminating between children with a hearing impairment (of any type) and children with no hearing impairment, using PTA results as the reference standard
- compare the diagnostic accuracy between the PTS and HC methods.

Measures of diagnostic accuracy are reported at the level of the ear and at the level of the child. From the outset we considered the ear-level analysis to be primary to the objectives addressed in this chapter because it directly addresses the question of the accuracy of the tests for discriminating between hearing and impaired ears. The child-level accuracy estimates are more relevant, however, for informing the refinement of the existing economic model of the benefits of a SES programme, reported in *Chapter 8*.

For peer review only

© Queen's Printer and Controller of HMSO 2016. This work was produced by Fortnum *et al.* under the terms of a commissioning contract issued by the Secretary of State for Health. This issue may be freely reproduced for the purposes of private research and study and extracts (or indeed, the full report) may be included in professional journals provided that suitable acknowledgement is made and the reproduction is not associated with any form of advertising. Applications for commercial reproduction should be addressed to: NIHR Journals Library, National Institute for Health Research, Evaluation, Trials and Studies Coordinating Centre, Alpha House, University of Southampton Science Park, Southampton SO16 7NS, UK.
For peer review only - http://bmjopen.bmj.com/site/about/guidelines.xhtml

Methods

Design

This study used a directly comparative two-gate ('case–control') design,[32] with separate sources (gates) used to recruit those with the target condition (hearing impairment – cases) and those without (no hearing impairment – controls). Case children were recruited through audiology services in England whereas the control children were recruited largely from Nottinghamshire primary schools. The implications of this are discussed later in this chapter (see *Discussion*).

Recruitment

Cases were children aged 4–6 years recruited between February 2013 and August 2014 who were identified by collaborating audiology services [in Nottingham, Sheffield, Leicester (City and County), Chesterfield, Derby, Mansfield, Lincoln, Birmingham, Huntingdon, Bradford, Rotherham and Doncaster] and who had permanent sensorineural or conductive hearing impairment averaged across the four frequencies 0.5, 1, 2 and 4 kHz, either bilaterally (average of 20–60 dB HL) or unilaterally (any level ≥ 20 dB HL). The senior paediatric audiologist in each centre drew up a list of all children meeting the definition criteria. Audiologists were given stamped envelopes to address and post to potential participants and agreed together with the researchers when to send the letters. Each identified family was sent a letter on behalf of the research team inviting them to take part in the study, together with information about the study. Children were not invited to take part if they were unwell such that their illness would affect the results of the tests or if the responsible audiologist felt it would be inappropriate or cause added unnecessary burden (e.g. seriously or terminally ill family member). Parents willing to take part replied directly to the research team. Eligible children for whom agreement was provided to take part were invited to undergo the two screening tests, either in their own homes, or at Nottingham Hearing Biomedical Research Unit (NHBRU), depending on their preference. Researchers sent holding letters if necessary for children < 4 years old, explaining that an appointment would be made for them after their fourth birthday. The reference standard definition of hearing impairment was based on PTA results, thus case children were also excluded if there was no record of a PTA in the previous 12 months or planned for the following 3 months, and if the family was unwilling to travel to their local service or to Nottingham to undergo the assessment.

Control children, defined as having no previously identified hearing impairment, were recruited from the Foundation Year and Year 1 of schools in the Nottingham area, between February 2013 and June 2014. The study researchers provided a letter of invitation and information pack for the school to distribute to all parents of children in the aforementioned year groups; invitation methods were agreed with the school. Children for whom agreement to take part was provided were invited to undergo the two screening tests and a PTA at NHBRU. Children needed to have the PTA measured in a soundproofed room, and hence the option to have the screening test at home was not possible.

To help ensure that the cases and controls were representative of the sources from which they were drawn, the audiologists and researchers were advised to not just pick those whom they thought might be easy to test. For all children the invitation pack contained an invitation letter, a one-page summary information sheet, a pictorial information sheet for the children and a pre-paid return envelope. Full participant information sheets were sent to the parent (either via e-mail or post) once an appointment was made (see *Appendix 3*) (the full sheet was originally included in the invitation pack, but a revision was made in December 2013, to be less overwhelming for the parent and to save paper).

Parents of all children involved were offered the opportunity to receive a short summary of the findings at the end of the study. A £20 book token (revised from £10 after 7 months of recruitment in an attempt to improve recruitment) was given to each child as a thank you for his/her time and inconvenience. Travel expenses to NHBRU were reimbursed in line with University of Nottingham standard policy. Schools that participated in the recruitment of control children were entered into a prize draw with a chance to win £100.

For peer review only - http://bmjopen.bmj.com/site/about/guidelines.xhtml

DOI: 10.3310/hta20360 HEALTH TECHNOLOGY ASSESSMENT 2016 VOL. 20 NO. 36

Assessment

Once a reply slip was received, the researcher contacted the parent (via telephone, e-mail or letter) to make an appointment either at NHBRU (case and control children) or at their home (case children only). They were also asked about access issues or translator needs. Parents were usually reminded about the appointment by telephone or e-mail on the day prior to the appointment. After case children had their appointment, the researcher phoned the audiologist, asking them to post their most recent PTA results to NHBRU. This could be a PTA measured in the previous 12 months or one that is scheduled to be measured in the following 3 months.

For all appointments the researcher checked that the parent had all the study information, explained what would happen at the appointment and how that fitted into the project, and answered any questions they might have. The consent form was explained and written consent obtained. The researcher emphasised that participation was voluntary and consent regarding the child's participation in the project could be withdrawn at any time without penalty or affecting the quality or quantity of the child's future medical care, or loss of benefits to which the child and his/her family was otherwise entitled. It was explained that in the event of withdrawal, their data collected so far could not be erased and that consent would be sought to use the data in the final analyses where appropriate. The informed consent form was signed and dated by the parent or legal guardian and researcher before the child entered the study – usually at the start of the screening appointment. Copies of the consent form were kept by the parent or legal guardian, the researchers, and, for case children only, in the child's hospital records (see *Appendix 3*).

Data collection was planned prospectively in advance of administering the tests and reference standard to the children. Background details [visit date, school for control children, hospital for case children, date of birth, postcode, background sound level, gender, ethnicity, medical conditions on the day (including respiratory infections)] were recorded on the case report form (CRF) and testing was then administered.

The audiometer (used for the PTS testing for all children and for the PTA for control children) and HC device were checked each day, by listening to the tones at the testing frequencies, to ensure they were audible. Valid calibration was undertaken in the previous 12 months for the audiometer and sound level meter and in the previous 3 years for the HC screener according to manufacturers' recommendations. Headphones were cleaned with a disinfectant wipe before each child was tested and new disposable cups were used for the HC screener. Items likely to cause noise (e.g. mobile phones, washing machines, televisions) were turned off where possible, and the level of background noise was measured with a sound level meter. Although, for control children, the door of the room in which the two screening tests were conducted was left open, and sometimes siblings were present, the ambient noise conditions were much quieter than rooms that are usually available in schools for conducting the hearing screen. For case children, where the screens were conducted at home, attempts were made to minimise the noise levels.

The researchers were trained in administering the PTA and PTS tests by the audiologists in the Children's Hearing Assessment Centre (CHAC) in Nottingham, using a mixture of observation, practice with children and feedback. Further familiarisation with the equipment and procedures was gained by testing staff from the research department.

The order of administering the two screening tests and which researcher undertook them were each determined by separate lists based on computer-generated simple (unrestricted) random numbers. Each test was administered to both ears. For case children, one researcher performed the PTS and another researcher performed the HC screen. For control children, one researcher carried out both the screening tests and then another researcher performed the PTA measurement. An effort was made to blind the second researcher to the results of the first test(s) by asking them to leave the room. The PTA result for case children obtained from their audiologist (measured within 12 months before or 3 months after the study visit) was examined only after the result of the screening tests were known.

© Queen's Printer and Controller of HMSO 2016. This work was produced by Fortnum *et al.* under the terms of a commissioning contract issued by the Secretary of State for Health. This issue may be freely reproduced for the purposes of private research and study and extracts (or indeed, the full report) may be included in professional journals provided that suitable acknowledgement is made and the reproduction is not associated with any form of advertising. Applications for commercial reproduction should be addressed to: NIHR Journals Library, National Institute for Health Research, Evaluation, Trials and Studies Coordinating Centre, Alpha House, University of Southampton Science Park, Southampton SO16 7NS, UK.

For peer review only - http://bmjopen.bmj.com/site/about/guidelines.xhtml

Screening tests

Pure-tone screen

A traditional PTS tests across four frequencies (0.5, 1, 2 and 4 kHz), which cover the majority of the frequencies contained in speech, was undertaken. The researcher was positioned to ensure that they had a clear view of the child without giving any visual cues throughout the test. The child was instructed to place a ball onto a frame every time they heard a sound, even if the sound was quiet. Hearing aids, glasses, hairbands and earrings were removed where relevant. The headphones were placed over the child's ears, adjusting the fit if necessary. A familiarisation tone (1 kHz at 60 dB HL) was presented as a practice to ensure that the child had understood the instructions. Up to three tones were presented at a single level (20 dB HL) for each of the frequencies in the order 1 kHz, 2 kHz, 4 kHz and 0.5 kHz. Each tone was held for 2–3 seconds, with staggered pauses between tones to reduce expectation. For each frequency and ear, at least two out of three responses to a tone was considered to be a pass at that frequency. One response from three presentations or zero responses from two presentations of the tone was considered to be a refer result. If the first two presentations were both passed at a given frequency or both referred at a given frequency the tone was not presented a third time. On the CRF a tick was used to indicate response (i.e. child placed a ball on the frame) and a cross for no response. Failure to hear at any frequency for a given ear was considered an overall refer result for that ear. All four frequencies were tested in one ear before changing to the other ear. For children who did not appear to hear any of the tones, the 20 dB HL tones were interspersed with sounds of greater intensity to check attention.

HearCheck screener

The HC[41] screener (*Figure 5*) automatically generates six tones in total – one tone for each combination of frequency and level: 1 kHz at 55, 35 and 20 dB HL and 3 kHz at 75, 55 and 35 dB HL. The tasks and test were explained, in particular that there was one tone much louder than the others. Children were asked to raise a hand when they heard a tone. The response method for the HC screen had to be quick to perform as there was only a very short automated gap between the tones, and was also chosen to be different from that for the PTS to keep the child's interest. For some children a different response method (e.g. tap the table) was used if they did not seem able to raise their hand for whatever reason. The child was asked to remove hearing aids, and also glasses and earrings if necessary for a good fit. A disposable cardboard ear cover was put onto the HC screener for each ear. The HC screener was held against the ear to be tested, often holding the child's head still with the free hand. The button was pressed and the first three tones were allowed to play for the first frequency. The button was then pressed again for the remaining three tones for the second frequency. The procedure was repeated on the other ear. A tick was recorded on the CRF for a response, and a cross for no response. The refer criterion for each ear is anything less than all six tones being responded to.

For peer review only

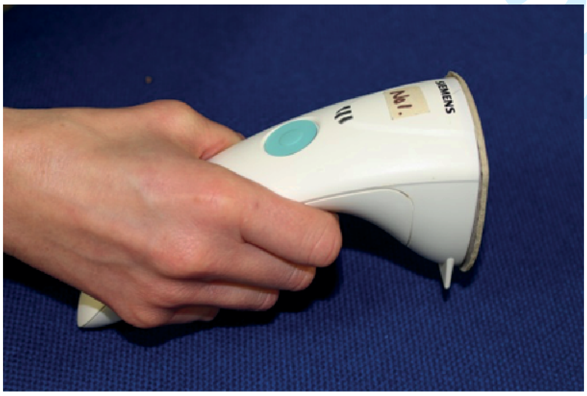

FIGURE 5 The HC screener.

For peer review only - http://bmjopen.bmj.com/site/about/guidelines.xhtml

DOI: 10.3310/hta20360 HEALTH TECHNOLOGY ASSESSMENT 2016 VOL. 20 NO. 36

Pure-tone audiometry reference standard

The PTA used to define the reference standard was administered in the study to all children recruited as controls. For control children, PTA was carried out in NHBRU at the same session as the screening tests were administered, using the audiometer in a soundproofed booth with the child sat facing away from the equipment. The parent was able to watch through an observation window. Instructions were given and then the headphones were put on. For familiarisation, 1 kHz tones at 60 dB HL were presented in one ear with further explanation provided if needed. Testing followed standard British Society of Audiology recommended procedure[42] without otoscopic examination or masking, for air conduction only. The testing tones were administered in the order 1, 2, 4, 8, 0.5 and 0.25 kHz for each ear. Sounds were presented for approximately 2 seconds with variable pauses between them. Each frequency was first presented at 40 dB HL. If not heard then the intensity was increased to 60 or 80 dB if needed. If still not heard then the equipment and the child's concentration were checked. If the participant heard the tone, then intensity was decreased by 10 dB HL, then if not heard it was increased by 5 dB HL. This stepwise approach was repeated until a threshold was found (defined as the quietest sound heard twice as the intensity was increased) for each frequency. The procedure was repeated in the second ear. The child indicated hearing the tone using the response button connected to the audiometer. If the child did not understand the use of the response button, then stacking bricks were occasionally used. The results were then printed out (only possible if the response button was used) and the audiometer results were transcribed into the CRF. The child identifier (ID) was written on the print out and attached to the CRF.

If a child decided that they did not want to continue with the testing during an assessment session, for whatever reason, he/she could be withdrawn from the study.

As the children recruited as controls had no previously identified hearing impairment, it was expected that they would pass the PTA. However, if the child was found to have a threshold of ≥ 30 dB HL at any frequency then, regardless of screening results, their parents were asked if they would like a referral to CHAC for further audiological testing. If such a referral was made this was indicated on the CRF and patient details were obtained. A letter and leaflet about the referral were given to the parents with an explanation that they would be contacted in the next 2–3 weeks with an appointment time. A copy of the CRF was passed to CHAC who made the appointment.

For children recruited as cases, a suitable PTA (administered between 12 months before and 3 months after the appointment) was obtained from the audiologist after the appointment and the data were added to the CRF. The original PTA result (with name, hospital number and other IDs removed and study ID added) was attached to the CRF.

Data collection

Each page of the CRF for each child was identified with the participant identity code, date of birth and partial postcode. The researchers entered the data from the CRFs into the study database. Copies of the CRFs were then sent to Peninsula Clinical Trials Unit (PenCTU) for second data entry and consistency checks. Emerging queries were noted to the Nottingham researchers and corrected where appropriate. Recruitment logs (for children nominally recruited as cases and controls, audiologist and school), the password-protected appointment spreadsheet and progress documents were completed as appropriate. Consent forms, reply slips and CRFs were filed in a secure place at NHBRU and then archived in a secure facility.

© Queen's Printer and Controller of HMSO 2016. This work was produced by Fortnum *et al.* under the terms of a commissioning contract issued by the Secretary of State for Health. This issue may be freely reproduced for the purposes of private research and study and extracts (or indeed, the full report) may be included in professional journals provided that suitable acknowledgement is made and the reproduction is not associated with any form of advertising. Applications for commercial reproduction should be addressed to: NIHR Journals Library, National Institute for Health Research, Evaluation, Trials and Studies Coordinating Centre, Alpha House, University of Southampton Science Park, Southampton SO16 7NS, UK.

For peer review only - http://bmjopen.bmj.com/site/about/guidelines.xhtml

Sample size calculation

The target sample size was 80 hearing impaired (HI) case children and 160 not hearing impaired (NHI) control children. Eighty HI children is a large enough sample to estimate a sensitivity of 80% with a margin of error of 10.4% based on the lower bound of the 95% CI; 160 NHI children is large enough to estimate a specificity of 80% with a margin of error of 7.0%. The margin of error for these estimates provides plausible ranges of values within which to test the sensitivity of the results from the economic model to our assumptions about screening accuracy (see *Chapter 8*).

Statistical analysis

The characteristics of the children were summarised separately for those nominally recruited as case children (i.e. with impairment) and those nominally recruited as control children (without impairment), using mean and SD for age, and numbers and percentages for gender and ethnicity. For case children, the time interval in weeks between measurement of the PTA reference standard and administration of the screening tests was summarised using the mean with SD and median with interquartile range (IQR).

The criteria used to define recruitment of case children were based on information available from the PTA. The children who were recruited as controls had no known hearing impairment. However, it was possible for children nominally recruited as cases to pass some elements of the reference standard and for children nominally recruited as controls to receive a refer result from the reference standard. Evaluation of the diagnostic accuracy of the screening tests was based on using the PTA reference standard, and not the nominal recruitment status of the children (i.e. it was not based on the 'gate' through which they were recruited). We classified those who were referred by PTA as HI and those who passed PTA as hearing (or NHI), regardless of whether they were nominally recruited as cases or controls. The discordance between nominal case–control status and the PTA reference standard classification is summarised later.

The accuracy of each of the screening test results was assessed in relation to the PTA reference standard classification based on analyses at the level of individual ear and at the level of individual child. From the outset we considered the ear-level analysis to be primary to the objectives addressed in this chapter because it directly addresses the question of the accuracy of the tests for discriminating between hearing and impaired ears. We also present child-level analyses, however, because the resulting accuracy estimates from these are most relevant for the economic model reported in *Chapter 8*. For the child-level analyses hearing was considered impaired (HI) if they had at least one impaired ear on the PTA reference standard and considered to have been referred by a given screening test if at least one of their ears was referred by the test.

When examining the relationship between the screening test results and the reference standard, separate sets of analyses were carried out for two different definitions of hearing impairment status. Under the primary definition, impairment was considered present when the PTA reference standard threshold was ≥ 30 dB on at least one of the four frequencies examined (0.5 kHz, 1 kHz, 2 kHz and 4 kHz) and considered absent when the reference threshold was < 30 dB on all four frequencies. Secondary analyses were carried out under the alternative definition where impairment was considered present when the mean PTA threshold across the four frequencies was ≥ 30 dB and considered absent when the mean threshold was < 30 dB. The primary definition is the stricter of the two.

For peer review only

For peer review only - http://bmjopen.bmj.com/site/about/guidelines.xhtml

DOI: 10.3310/hta20360 HEALTH TECHNOLOGY ASSESSMENT 2016 VOL. 20 NO. 36

Separate sets of analyses relating the screening test result and the reference standard classification were also conducted based on the inclusion of different subsets of impaired ears (or children). In the primary analysis all HI ears (or children) were included regardless of whether nominally recruited as a case child or as a control child. In two further sets of secondary analyses we included only impaired ears (or children) for those nominally recruited as cases and then included only impaired ears (or children) for those nominally recruited as controls.

Cross-tabulation was used to report the test results (refer vs. pass) for the PTS and HC screen in relation to each other, separately for the groups of ears (or children) that were classified on the PTA reference standard as impaired (to compare sensitivity between tests), hearing (to compare specificity between tests) and missing. Sensitivity, specificity, positive likelihood ratio and negative likelihood ratio were reported for the PTS and HC screening tests for ear-level and child-level analyses and all scenarios defined by the above definitions of impairment. Sensitivity is the percentage of impaired ears (or children) that are referred by the test; specificity is the percentage of hearing ears (or children) that pass the test; positive likelihood ratio is the ratio of the percentages that are referred by the screening test between the impaired and hearing groups; and negative likelihood ratio is the ratio of the percentages that pass the screening test between the impaired and hearing groups. For the ear-level analyses, correlation between the results for ears of a given child was accounted for when reporting CIs for sensitivity and specificity by fitting null logistic regression models (i.e. with no covariates) to the binary test result outcome with information sandwich ('robust') estimates of standard error, with the resulting log odds and 95% CI converted to the proportions (percentage) scale. When estimating sensitivity the model was fitted using only impaired ears and when estimating specificity using only hearing ears. The test result outcome was coded 1 for refer and 0 for pass when estimating sensitivity and vice versa when estimating specificity.

We report the absolute difference in percentages between the PTS and HC screen for both sensitivity and specificity with 95% CIs and McNemar's test *p*-value [using the mcc command in Stata software (version 13.1, StataCorp LP, College Station, TX, USA)]. The calculation of the CI and *p*-value recognise the pairing of the PTS and HC test results within ear (or within child). This was done for ear-level and child-level analyses and all scenarios defined by the above definitions of impairment. Unlike when estimating the accuracy of each test, when comparing accuracy between tests for the ear-level analysis there was no need to allow for the correlation between results related to ears from the same children because the estimated difference in sensitivity (and specificity) between the PTS and HC tests is based on information within ears. An alternative analysis in which the data structure was reshaped so that there were four rows per child (PTS result for the left ear, HC result for the left ear, PTS result for the right ear and HC result for the right ear) and logistic regression models fitted to the test result outcome (coded 1 for pass and 0 for refer) on the predictor test type (coded 1 for PTS and 0 for HC) to estimate the difference between test types in accuracy, taking account of the correlation of test results for ears that belong to the same child, provided identical CIs and *p*-values to those reported here.

Missing data

To be included in the main ear-level analysis the ear needed to have provided data on both the screening tests and provided hearing-level data on all four frequencies presented under the PTA reference standard. It was possible for a given child, therefore, to provide only one ear that was used in the ear-level analyses. To be included in the child-level analysis the child had to provide full data on the PTS and HC tests and the PTA reference standard for both ears. Analyses were carried out using Stata statistical software.

Results

Participants

Nominal case children were recruited from 14 centres. The original eight centres in the East Midlands were: Nottingham, Leicester City, Leicester County, Sheffield, Derby, Lincoln, Mansfield and Chesterfield. Owing to recruitment difficulties a further six centres were added: Bradford, Rotherham, Huntingdon,

© Queen's Printer and Controller of HMSO 2016. This work was produced by Fortnum *et al.* under the terms of a commissioning contract issued by the Secretary of State for Health. This issue may be freely reproduced for the purposes of private research and study and extracts (or indeed, the full report) may be included in professional journals provided that suitable acknowledgement is made and the reproduction is not associated with any form of advertising. Applications for commercial reproduction should be addressed to: NIHR Journals Library, National Institute for Health Research, Evaluation, Trials and Studies Coordinating Centre, Alpha House, University of Southampton Science Park, Southampton SO16 7NS, UK.

For peer review only - http://bmjopen.bmj.com/site/about/guidelines.xhtml

Birmingham Children's Hospital, Birmingham Heartlands Hospital and Doncaster. From 379 invitations sent by the audiologists, a total of 86 replies were received, a response rate of 23%. Eight children were not eligible, being outside the required age range. We were unable to contact one of the initial respondents, and we were unable to see a further two children due to researcher illness just before the close of recruitment. We recruited and tested the remaining 75 children. See the flow diagram (*Figure 6*) below. Details of recruitment by centre are given in *Table 8*.

Nominal control children were recruited from 51 of the 164 schools in the Nottingham area that were invited by post to take part (response rate of 31% at school level). Twenty-three of the participating schools agreed to take part only after a follow-up telephone call to discuss the information received.

The 51 schools, between them, gave information packs to the parents of 2787 children, of whom 291 (10.4%; median reply rate per school of 9.1%, IQR 4.6–14.9%, range 0–28.6%) replied, confirming they would like to participate. Seven of the schools that agreed to take part did not recruit any children. An additional 11 siblings of children who attended the appointment but who did not receive the invitation were in the correct age range and parents agreed for them to take part, bringing the number of children initially indicating that they wanted to participate to 302. Eight of these children were subsequently found to be ineligible for the study (one was too old, six already had hearing problems identified and one replied after recruitment closed), 11 changed their minds about taking part and we were unable to see 43 either because we could not make an appointment (mostly not contactable) or because they did not attend the arranged appointment.

The remaining 240 children were recruited as nominal controls and seen for study appointments (*Figure 7*).

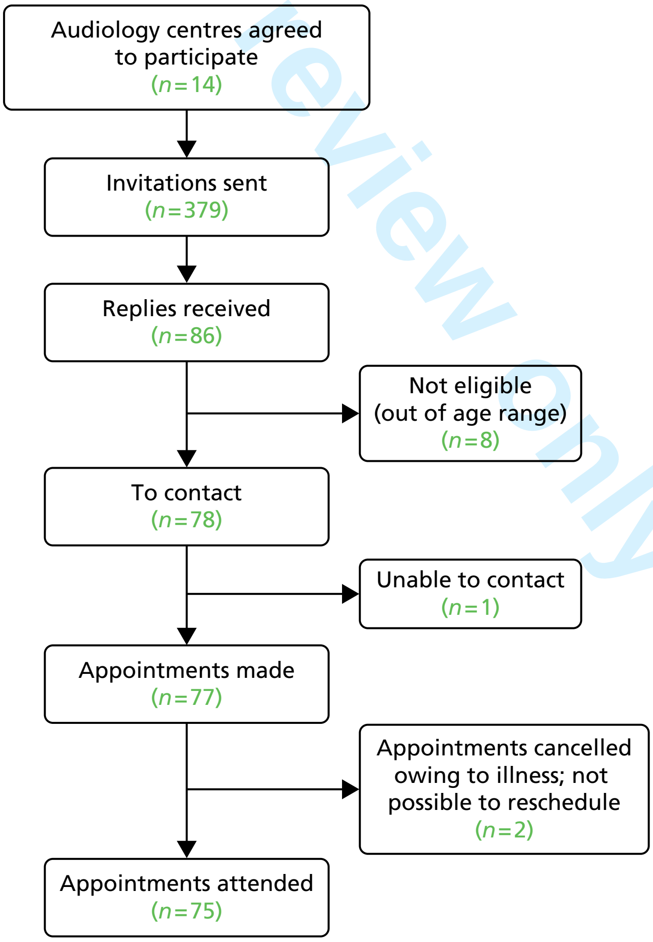

FIGURE 6 Numbers of case children in the diagnostic accuracy study.

For peer review only

For peer review only - http://bmjopen.bmj.com/site/about/guidelines.xhtml

DOI: 10.3310/hta20360 HEALTH TECHNOLOGY ASSESSMENT 2016 VOL. 20 NO. 36

TABLE 8 Numbers of invitations, replies and consents by centre

Audiology clinic	Number of invitations sent		Date posted to audiologist	Number of replies received	Ineligible (wrong age)	Eligible children not seen	Number tested	Child did not complete the tests
Nottingham	Original posting	39	8 February 2013	17	3	0	14	1
	Reminders	27	April 2014					
	Newly identified[a]	0	N/A					
Leicester County	Original posting	8	6 February 2013	2	0	0	2	0
	Reminders	4	15 December 2013					
	Newly identified	0	15 December 2013					
Sheffield	Original posting	47	5 March 2013	7	1	0	6	0
	Reminders	9	6 January 2013					
	Newly identified	1	6 January 13					
Derby	Original posting	35	May 2013	13	1	1	11	0
	Reminders	N/A	January 2014					
	Newly identified	21	January 2014					
Lincoln	Original posting	18	June 2013	9	0	0	9	1
	Reminders	8	24 March 2014					
	Newly identified	7	24 March 2014					
Leicester City	Original posting	16	June 2013	7	0	0	7	0
	Reminders	8	April 2014					
	Newly identified	28	April 2014					
Mansfield	Original posting	20	July 2013	5	0	0	5	1
	Reminders	14	March 2014					
	Newly identified	4	March 2014					

continued

For peer review only

© Queen's Printer and Controller of HMSO 2016. This work was produced by Fortnum *et al.* under the terms of a commissioning contract issued by the Secretary of State for Health. This issue may be freely reproduced for the purposes of private research and study and extracts (or indeed, the full report) may be included in professional journals provided that suitable acknowledgement is made and the reproduction is not associated with any form of advertising. Applications for commercial reproduction should be addressed to: NIHR Journals Library, National Institute for Health Research, Evaluation, Trials and Studies Coordinating Centre, Alpha House, University of Southampton Science Park, Southampton SO16 7NS, UK.

For peer review only - http://bmjopen.bmj.com/site/about/guidelines.xhtml

TABLE 8 Numbers of invitations, replies and consents by centre (*continued*)

Audiology clinic	Number of invitations sent		Date posted to audiologist	Number of replies received	Ineligible (wrong age)	Eligible children not seen	Number tested	Child did not complete the tests
Chesterfield	Original posting	16	July 2013	5	2	0	3	0
	Reminders	14	January 2014					
	Newly identified	6	January 2014					
Bradford	Original posting	37	8 January 2014	7	1	1	5	0
	Reminders	30	March 2014					
	Newly identified	0	March 2014					
Rotherham	Original posting	17	February 2014	2	0	0	2	0
	Reminders	15	June 2014					
	Newly identified	0	N/A					
Birmingham (Heartlands)	Original posting	18	28 January 2014	5	0	0	5	0
	Reminders	13	March 2014					
	Newly identified	0	March 2014					
Huntingdon	Original posting	8	16 January 2014	2	0	0	2	0
	Reminders	6	June 2014					
	Newly identified	0	N/A					
Birmingham (Children's Hospital)	Original posting	24	12 February 2014	3	0	0	3	0
	Reminders	21	June 2014					
	Newly identified	0	N/A					
Doncaster	Original posting	9	26 March 2014	2	0	1	1	1
	Reminders	7	July 2014					
	Newly identified	0	N/A					
Total		379		86	8	3	75	3

N/A, not applicable.
a To boost recruitment, a second search was made during the study, to identify new referrals or younger children not previously identified.

For peer review only - http://bmjopen.bmj.com/site/about/guidelines.xhtml

For peer review only

DOI: 10.3310/hta20360 HEALTH TECHNOLOGY ASSESSMENT 2016 VOL. 20 NO. 36

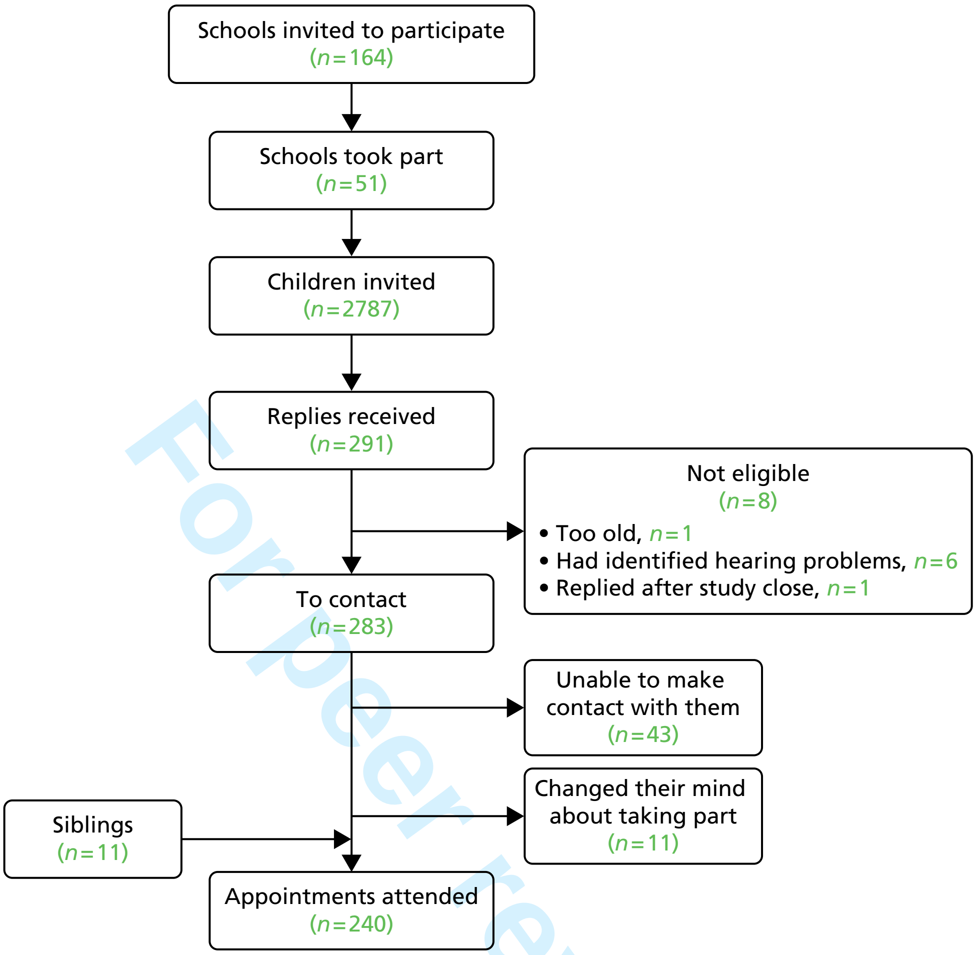

FIGURE 7 Numbers of control children in the diagnostic accuracy study.

For peer review only

Demographic characteristics

Table 9 summarises the demographic characteristics of children who attended appointments (315) by whether they were nominally recruited as cases (75 children) or controls (240 children). The groups were similar with respect to gender and age. Four-fifths of each group was categorised as white on ethnicity. The nominal case group had a higher percentage of Asians (15% vs. 4%) and a lower percentage of mixed ethnicity children (1% vs. 9%) than the nominal control group.

TABLE 9 Demographic characteristics of recruited children

Characteristic	Recruited as case child (*n* = 75)	Recruited as control child (*n* = 240)
Male, *n* (%)	38 (51)	117 (49)
Age (years), mean (SD, range)	5.4 (0.9, 3.9–7.0)	5.4 (0.6; 4.0–6.9)
Ethnicity		
White, *n* (%)	61 (81)	189 (79)
Black, *n* (%)	2 (3)	14 (6)
Asian, *n* (%)	11 (15)	10 (4)
Mixed, *n* (%)	1 (1)	22 (9)
Other, *n* (%)	0 (0)	5 (2)

© Queen's Printer and Controller of HMSO 2016. This work was produced by Fortnum *et al.* under the terms of a commissioning contract issued by the Secretary of State for Health. This issue may be freely reproduced for the purposes of private research and study and extracts (or indeed, the full report) may be included in professional journals provided that suitable acknowledgement is made and the reproduction is not associated with any form of advertising. Applications for commercial reproduction should be addressed to: NIHR Journals Library, National Institute for Health Research, Evaluation, Trials and Studies Coordinating Centre, Alpha House, University of Southampton Science Park, Southampton SO16 7NS, UK.

For peer review only - http://bmjopen.bmj.com/site/about/guidelines.xhtml

Time interval between screening test and reference standard for children nominally recruited as cases

Table 10 summarises the time interval between when the screening tests were administered and when the PTA reference standard was measured for the nominal cases, reporting the difference in weeks. Separate rows summarise the time interval for the 65 nominal cases for which reference standard data were available before the tests were administered, and for the 10 nominal cases who completed the screening tests before the reference standard. The absolute time interval is summarised for all 75 nominal cases. The median absolute time interval was 16 weeks. The reference standard was completed within the criterion period of 12 months (52 weeks) before to 3 months (13 weeks) after the screening tests for 73 children. For each of the remaining two children a reference standard measure was available prior to the 52-week cut-off (65 and 63 weeks before the screening test) and another was available for each at 24 weeks after the screening test. The two measures for each child showed very similar results and indicated a stable hearing impairment by both definitions and the children were therefore included in the analyses. As described in the methods section the children nominally recruited as controls completed the tests and the reference standard on the same day.

Missing data and indeterminate results

Of the 630 recruited ears, 600 (95.2%) provided full data on the PTS and HC tests and scores for all four frequencies of the PTA reference standard and were included in the main analyses. Sixty-two (82.7%) of the 75 children recruited as cases and 233 (97.1%) of the 240 children recruited as controls (total = 295/315, 93.7%), provided full data on the tests and reference standard. There were no indeterminate screening test or PTA results. For children recruited as cases it was usually the PTA that was part missing; however, one child refused to do the HC test, another child refused to do the PTS test and one child had no physical ear on one side. For those recruited as controls, PTA and PTS results were missing for two participants due to equipment failure. There were a further two children with autism who were unable to complete the tests and the remainder were children who refused to do or complete other tests mainly because of lack of concentration or finding the headphones too tight.

Concordance between nominal case–control status and pure-tone audiometry reference standard classification

A unique feature of the two-gate ('case–control') design used for this accuracy study is that the criteria used to recruit children as cases and controls were not the same as the reference standard classification (using the PTA) that was used to define presence of the target condition when evaluating the screening tests. In other words the nominal target condition status is not necessarily consistent with the status based on application of the reference standard. For children nominally recruited as cases, hearing status was based on audiology notes. The criterion for inclusion was a bilateral impairment up to an average of 60 dB HL or a unilateral impairment of any level. Consequently, although nominally recruited as case or control children, some of the case children had no hearing impairment when assessed on the PTA reference standard and some of the control children were categorised as having impairment in the child-level analyses. Furthermore, and of particular relevance to the fact that the primary analyses are conducted at the level of the ear, there were case children for whom the PTA reference standard defined them as having the target condition in one ear but not the other (i.e. had unilateral impairment). There were, therefore, ears that were categorised and analysed based on the reference standard as HI that belonged to children nominally recruited as controls and ears that were categorised and analysed as NHI that belonged to children nominally recruited as cases.

TABLE 10 Time interval in weeks between administering the screening tests (PTS and HC) and the reference standard (PTA) for children nominally recruited as cases only ($n = 75$)

Time interval in weeks	*n*	Mean (SD)	Median (IQR)	Range
Reference standard performed first	65	19.3 (14.2)	17.3 (7.4–24.4)	0.7–63.3
Screening test performed first	10	8.9 (7.0)	9.5 (1.6–11.9)	1.1–24.1
Absolute interval for all children	75	17.9 (13.9)	16.3 (6.9–24.1)	0.7–63.3

For peer review only - http://bmjopen.bmj.com/site/about/guidelines.xhtml

DOI: 10.3310/hta20360 HEALTH TECHNOLOGY ASSESSMENT 2016 VOL. 20 NO. 36

Figure 8 indicates the number of ears belonging to participants nominally recruited as cases and controls that under the reference standard PTA were classified as HI, classified as NHI or for which there were insufficient data to classify the ear (i.e. PTA data were missing for that ear). The figure is based on the PTA reference standard definition of impairment as having a HL of ≥ 30 dB on at least one of the four frequencies (the primary definition). Twenty-six of the 150 ears of children nominally recruited as cases were classified on the reference standard as hearing and 48 of the 480 ears of children nominally recruited as controls were classified as impaired. Similar data are summarised at the level of child in *Figure 9*. Two of the 75 children who were nominally recruited as cases were classified on the reference standard as hearing. The pure-tone audiograms provided by the child's local audiology service did not match the inclusion criteria but the children were tested within the study because, as per the protocol, the research team did not access the PTA until after the screening tests were undertaken. Of the 240 children who were nominally recruited as controls 37 were classified as impaired. We suggest that parents who had concerns about their child's hearing were more likely to take up the invitation to take part.

Accuracy of the pure-tone screen and HearCheck screener for identifying hearing impairment at the level of the ear

Figures 10 and *11* present flow charts that describe the number of impaired ears (based on a PTA score of ≥ 30 dB on at least one of the four frequencies) and hearing ears that passed and referred on the PTS and HC tests, respectively. *Table 11* summarises the relationship between the PTS test results and the HC test results separately for impaired ears, hearing ears and ears for which information on the reference standard was missing. The figures highlighted in green indicate the numbers that were used in the calculation of sensitivity and specificity. The relationship between the PTS and HC results is summarised for the other five scenarios (see *Tables 47–51*, *Appendix 4*).

Table 12 reports the sensitivity and specificity of the screening tests for the ear level. The sensitivity was 94.2% for the PTS and 89.0% for the HC screen. The 95% CIs for sensitivity indicate that we can be fairly certain that the true sensitivity is no lower than 89% for the PTS but could be as low as 83% for the HC screen. The McNemar's test result ($p = 0.02$) indicates evidence that the true sensitivity is greater for the PTS than for the HC screener. The difference of 5% in sensitivity implies that for every 20 impaired ears tested the PTS would correctly identify an extra ear as having a hearing problem compared with using the HC screener. The corresponding values for specificity were 82.2% for the PTS and 86.5% for the HC screener, with evidence provided by McNemar's test that the true specificity is higher for the HC screener than the PTS ($p = 0.02$).

Table 13 reports the sensitivity and specificity for each combination of definition of impairment (PTA score of ≥ 30 dB on at least one frequency vs. a mean PTA score of ≥ 30 dB across all four frequencies) and subset of impaired ears used to calculate sensitivity (all ears, ears of children nominally recruited as cases and ears of children nominally recruited as controls). The results for the primary analysis for which the reference standard is a PTA score of ≥ 30 dB on at least one frequency and all ears are used to calculate sensitivity is shown on the top row. The sensitivity is generally higher (especially for the HC screener) when impairment is defined based on average HL across the four frequencies. This might be expected, as the primary definition of impairment is more stringent and thus more likely to result in less severe impaired ears being included in the impaired group. For the same reason the specificity is lower for both tests when impairment status is based on average HL across the frequencies presented under the PTA. Restricting impaired ears in the analysis to only those belonging to children recruited as cases results in increased sensitivity relative to inclusion of all ears. Again, this would be expected as ears of such children would be expected to have more severe hearing loss. Restricting impaired ears in the analysis to only those belonging to children recruited as controls results in lower sensitivity. Although only a sensitivity analysis, this latter result is notable because the impaired ears of children nominally recruited as controls may be more representative of the spectrum of impairment in the type of child that the screen would predominantly want to identify in a school-based setting (i.e. children with less severe hearing impairment).

© Queen's Printer and Controller of HMSO 2016. This work was produced by Fortnum *et al.* under the terms of a commissioning contract issued by the Secretary of State for Health. This issue may be freely reproduced for the purposes of private research and study and extracts (or indeed, the full report) may be included in professional journals provided that suitable acknowledgement is made and the reproduction is not associated with any form of advertising. Applications for commercial reproduction should be addressed to: NIHR Journals Library, National Institute for Health Research, Evaluation, Trials and Studies Coordinating Centre, Alpha House, University of Southampton Science Park, Southampton SO16 7NS, UK.

For peer review only - http://bmjopen.bmj.com/site/about/guidelines.xhtml

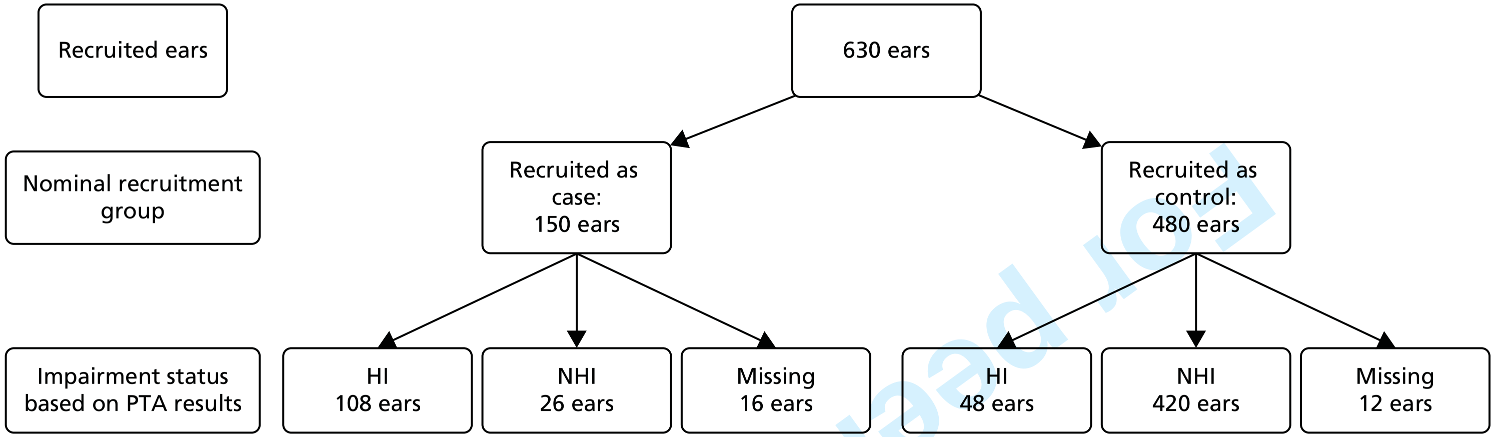

FIGURE 8 Nominal recruitment status at the ear level and the reference standard classification by or of hearing impairment status based on a PTA score of ≥ 30 dB on at least one of the four frequencies.

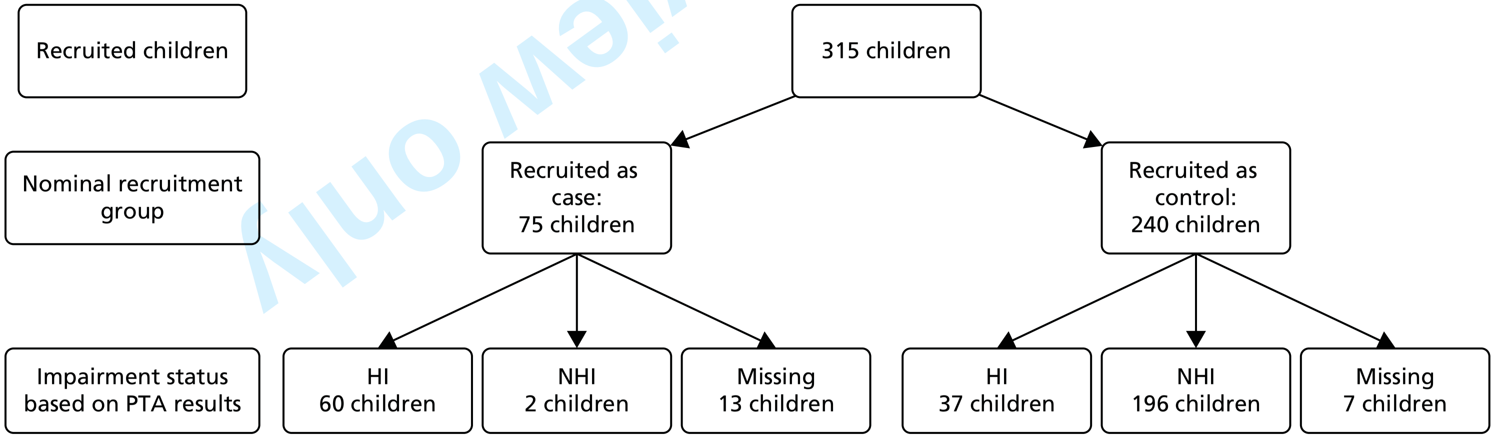

FIGURE 9 Nominal recruitment status at the child level and the reference standard classification by or of hearing impairment status based on a PTA score of ≥ 30 dB on at least one of the four frequencies for at least one ear.

For peer review only - http://bmjopen.bmj.com/site/about/guidelines.xhtml

DOI: 10.3310/hta20360 HEALTH TECHNOLOGY ASSESSMENT 2016 VOL. 20 NO. 36

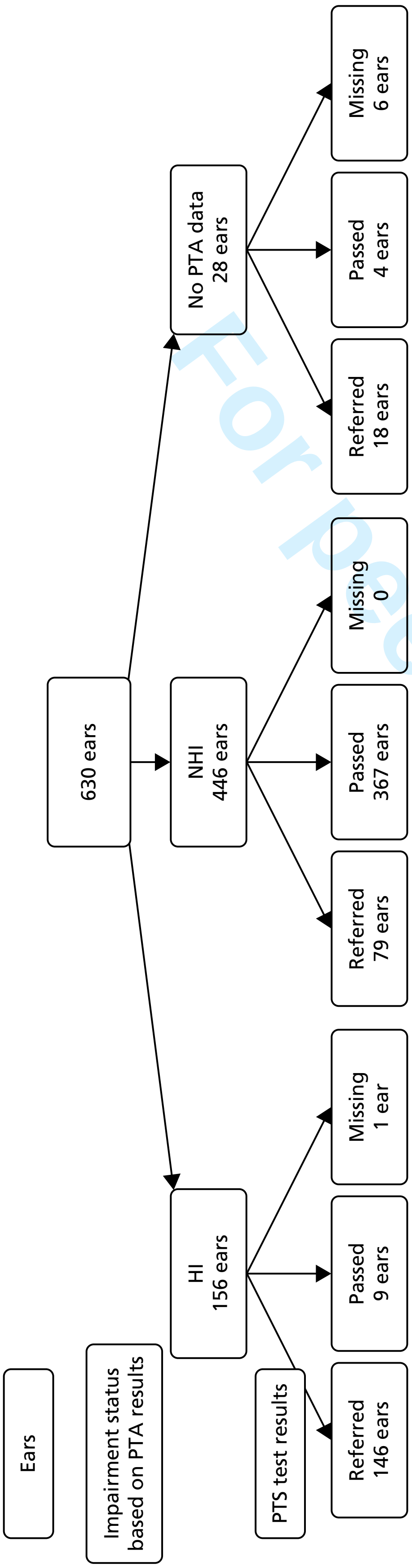

FIGURE 10 Pure-tone screen results at the ear level by hearing impairment status based on a PTA score of ≥ 30 dB on at least one of the four frequencies.

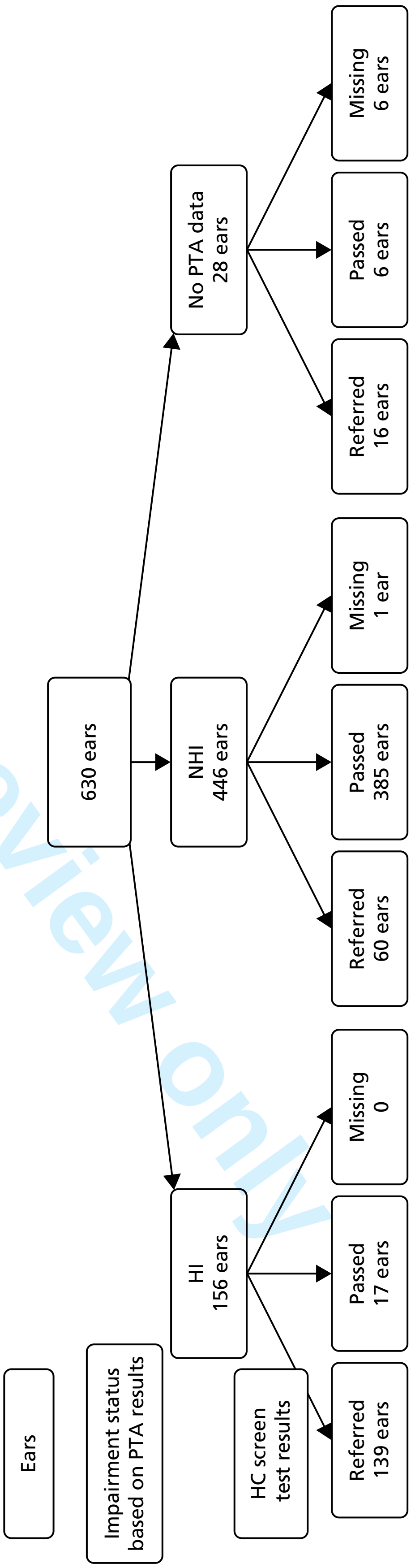

FIGURE 11 HearCheck screener test results at the ear level by hearing impairment status based on a PTA score of ≥ 30 dB on at least one of the four frequencies.

For peer review only

© Queen's Printer and Controller of HMSO 2016. This work was produced by Fortnum *et al.* under the terms of a commissioning contract issued by the Secretary of State for Health. This issue may be freely reproduced for the purposes of private research and study and extracts (or indeed, the full report) may be included in professional journals provided that suitable acknowledgement is made and the reproduction is not associated with any form of advertising. Applications for commercial reproduction should be addressed to: NIHR Journals Library, National Institute for Health Research, Evaluation, Trials and Studies Coordinating Centre, Alpha House, University of Southampton Science Park, Southampton SO16 7NS, UK.

For peer review only - http://bmjopen.bmj.com/site/about/guidelines.xhtml

TABLE 11 Cross-tabulation of the PTS vs. HC test results: impaired ears defined as those with a PTA score of ≥ 30 dB on at least one of the four frequencies – analyses include all impaired ears regardless of whether belonging to children nominally recruited as cases or controls

		PTS test results			
		Refer	Pass	Missing	Total
Impaired ears (by reference standard)					
HC test results	Refer	136	2	1	139
	Pass	10	7	0	17
	Missing	0	0	0	0
	Total	146	9	1	156
Hearing ears (by reference standard)					
HC test results	Refer	34	26	0	60
	Pass	45	340	0	385
	Missing	0	1	0	1
	Total	79	367	0	446
Missing ears (no reference standard)					
HC test results	Refer	13	2	1	16
	Pass	3	2	1	6
	Missing	2	0	4	6
	Total	18	4	6	28

Shading indicates the numbers that were used in the calculation of sensitivity and specificity.

TABLE 12 Accuracy of the PTS and HC screener in ear-level analyses: impaired ears defined as those with a PTA score of ≥ 30 dB on at least one of the four frequencies – analyses include all impaired ears regardless of whether belonging to children nominally recruited as cases or controls

	PTS	HC	Difference in accuracy (PTS – HC)	
Measure	Estimate (95% CI)	Estimate (95% CI)	Estimate (95% CI)	*p*-value
Sensitivity	94.2% (89.0% to 97.0%)	89.0% (82.9% to 93.1%)	5.2% (0.2% to 10.1%)	0.02
Specificity	82.2% (77.7% to 86.0%)	86.5% (82.5% to 90.0%)	–4.3% (–8.2% to –0.4%)	0.02
Positive likelihood ratio	5.31	6.60		
Negative likelihood ratio	0.07	0.13		

For peer review only

For peer review only - http://bmjopen.bmj.com/site/about/guidelines.xhtml

DOI: 10.3310/hta20360 HEALTH TECHNOLOGY ASSESSMENT 2016 VOL. 20 NO. 36

TABLE 13 Accuracy of the PTS and HC screener at ear level across different definitions of impairment status and using different subsets of impaired ears based on whether they belong to children nominally recruited as cases or controls

		PTS		HC	
Reference standard	Subset of impaired ears	Sensitivity	Specificity	Sensitivity	Specificity
PTA score of ≥ 30 dB on at least one frequency	All (primary analysis)	94.2%	82.2%	89.0%	86.5%
	Nominal cases	99.1%	82.2%	97.2%	86.5%
	Nominal controls	83.3%	82.2%	70.8%	86.5%
Average PTA score of ≥ 30 dB across frequencies	All	95.7%	76.4%	94.8%	81.8%
	Nominal cases	98.9%	76.4%	97.8%	81.8%
	Nominal controls	84.0%	76.4%	84.0%	81.8%

Accuracy of the pure-tone screen and HearCheck screener for identifying hearing impairment at the level of the child

Figures 12 and *13* contain flow charts that describe the number of hearing-impaired children (defined based on a PTA score of ≥ 30 dB on at least one of the four frequencies in either ear) and hearing children who passed and referred on the PTS and HC tests, respectively. *Table 14* summarises the relationship between the PTS test results and the HC test results for hearing-impaired children, hearing children and children who did not provide full data on the reference standard. The numbers highlighted in green were used to calculate sensitivity and specificity. The relationship between the PTS and HC results is summarised for the other five scenarios (see *Tables 52–56*, *Appendix 4*). *Table 15* reports the sensitivity and specificity of the screening tests at child level. The sensitivity estimates (95.9% for the PTS and 88.7% for the HC screener) were similar to those reported for the ear-level analyses. The McNemar's test ($p = 0.02$) indicates that the true sensitivity is greater for the PTS than for the HC screener. The difference of 7% in sensitivity implies that for every 14 impaired children tested the PTS would correctly identify an extra child as having a hearing problem compared with using the HC screener. Specificity at the child level was higher for the HC screener than for the PTS (83.8% vs. 79.8%) but there was little evidence of a true difference ($p = 0.18$).

Table 16 reports the sensitivity and specificity for each combination of definition of impairment (PTA score of ≥ 30 dB on at least one frequency vs. an average PTA score of ≥ 30 dB across all four frequencies) and subset of hearing-impaired children used to calculate sensitivity (all children, children nominally recruited as cases and children nominally recruited as controls). The results for the primary analysis in which the reference standard is a PTA score of ≥ 30 dB on at least one frequency for at least one ear and all impaired children are used to calculate sensitivity are shown on the top row. The pattern of results is similar to the ear-level analyses. The use of less stringent passing criteria for the reference standard (average PTA score of ≥ 30 dB across the four frequencies presented) generally results in higher sensitivity and markedly lower specificity. Restricting the sample to impaired children who were nominally recruited as cases resulted in higher sensitivity and restricting the sample to impaired children who were nominally recruited as controls resulted in lower sensitivity.

© Queen's Printer and Controller of HMSO 2016. This work was produced by Fortnum *et al.* under the terms of a commissioning contract issued by the Secretary of State for Health. This issue may be freely reproduced for the purposes of private research and study and extracts (or indeed, the full report) may be included in professional journals provided that suitable acknowledgement is made and the reproduction is not associated with any form of advertising. Applications for commercial reproduction should be addressed to: NIHR Journals Library, National Institute for Health Research, Evaluation, Trials and Studies Coordinating Centre, Alpha House, University of Southampton Science Park, Southampton SO16 7NS, UK.

For peer review only - http://bmjopen.bmj.com/site/about/guidelines.xhtml

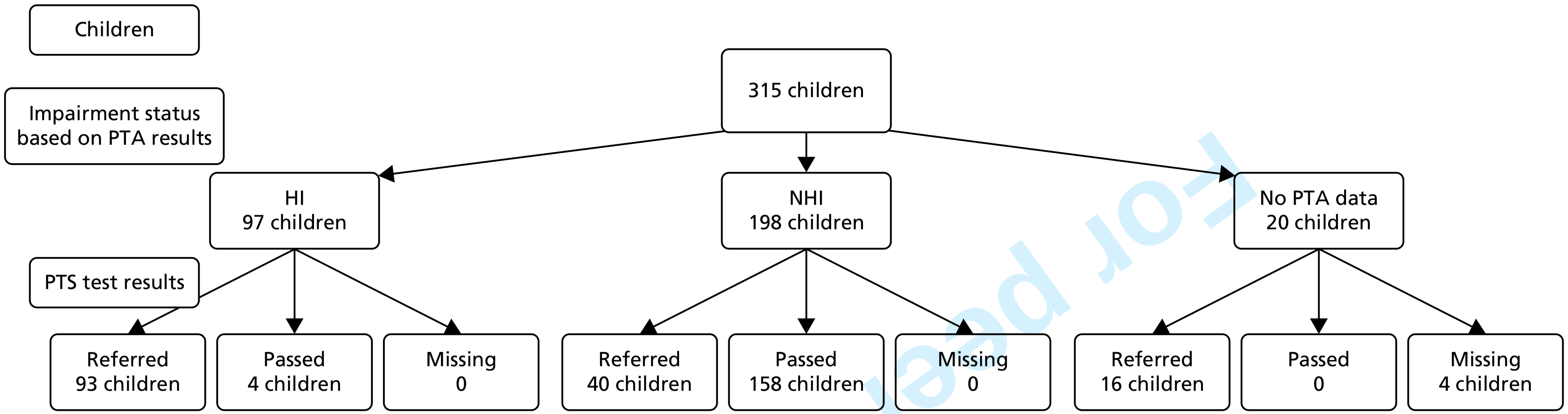

FIGURE 12 Pure-tone screen test results at the child level by hearing impairment status based on a PTA score of ≥ 30 dB on at least one of the four frequencies in at least one ear.

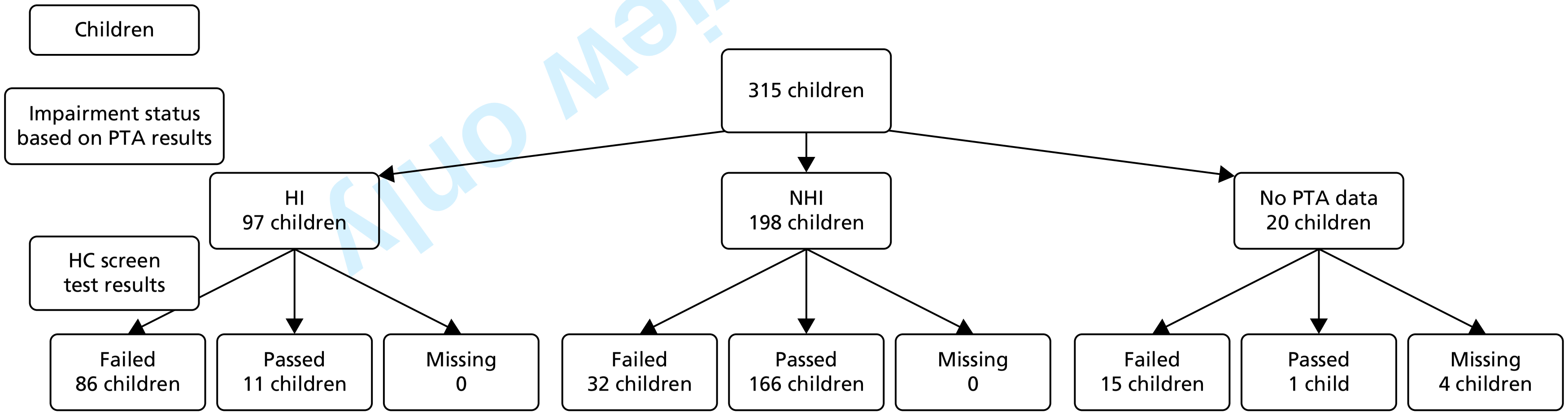

FIGURE 13 HearCheck test results at the child level by hearing impairment status based on a PTA score of ≥ 30 dB on at least one of the four frequencies in at least one ear.

For peer review only - http://bmjopen.bmj.com/site/about/guidelines.xhtml

DOI: 10.3310/hta20360 HEALTH TECHNOLOGY ASSESSMENT 2016 VOL. 20 NO. 36

TABLE 14 Cross-tabulation of the PTS vs. HC test results: impaired children defined as those with a PTA score of ≥ 30 dB on at least one of the four frequencies in at least one ear – analyses include all children regardless of whether nominally recruited as case or control

		PTS test results			
		Refer	Pass	Missing	Total
Impaired (by reference standard)					
HC test results	Refer	85	1	0	86
	Pass	8	3	0	11
	Missing	0	0	0	0
	Total	93	4	0	97
Hearing (by reference standard)					
HC test results	Refer	18	14	0	32
	Pass	22	144	0	166
	Missing	0	0	0	0
	Total	40	158	0	198
Missing (no ears reference standard)					
HC test results	Refer	14	0	1	15
	Pass	1	0	0	1
	Missing	1	0	3	4
	Total	16	0	4	20

Shading indicates the numbers that were used in the calculation of sensitivity and specificity.

TABLE 15 Accuracy of the PTS and HC screener in child-level analyses: impaired children defined as those with a PTA score of ≥ 30 dB on at least one of the four frequencies in at least one ear – analyses include all impaired children regardless of whether nominally recruited as case or control

	PTS	HC	Difference in accuracy (PTS – HC)	
Measure	Estimate (95% CI)	Estimate (95% CI)	Estimate (95% CI)	*p*-value
Sensitivity	95.9% (89.8% to 98.9%)	88.7% (80.6% to 94.2%)	7.2% (0.3% to 14.1%)	0.02
Specificity	79.8% (73.5% to 85.2%)	83.8% (78.0% to 88.7%)	–4.0% (–10.5% to 2.3%)	0.18
Positive likelihood ratio	4.75	5.49		
Negative likelihood ratio	0.05	0.14		

© Queen's Printer and Controller of HMSO 2016. This work was produced by Fortnum *et al.* under the terms of a commissioning contract issued by the Secretary of State for Health. This issue may be freely reproduced for the purposes of private research and study and extracts (or indeed, the full report) may be included in professional journals provided that suitable acknowledgement is made and the reproduction is not associated with any form of advertising. Applications for commercial reproduction should be addressed to: NIHR Journals Library, National Institute for Health Research, Evaluation, Trials and Studies Coordinating Centre, Alpha House, University of Southampton Science Park, Southampton SO16 7NS, UK.

For peer review only - http://bmjopen.bmj.com/site/about/guidelines.xhtml

TABLE 16 Accuracy of the PTS and HC tests at child level across different definitions of impairment status and using different subsets of impaired children based on whether they were nominally recruited as cases or controls

		PTS		HC	
Reference standard	Subset of impaired children	Sensitivity	Specificity	Sensitivity	Specificity
PTA score of ≥ 30 dB on at least one frequency	All (primary analysis)	95.9%	79.8%	88.7%	83.8%
	Nominal cases	98.3%	79.8%	98.3%	83.8%
	Nominal controls	91.9%	79.8%	73.0%	83.8%
Average PTA score of ≥ 30 dB across frequencies	All	97.3%	72.7%	93.3%	78.2%
	Nominal cases	98.1%	72.7%	98.1%	78.2%
	Nominal controls	95.2%	72.7%	81.0%	78.2%

Discussion

In this chapter we have estimated and compared the diagnostic accuracy of the PTS and HC tests for discriminating between children with and without hearing impairment (sensorineural, permanent conductive or transient) basing our reference standard on the results of PTA. Our primary definition of hearing impairment was a threshold of ≥ 30 dB on at least one of the four frequencies presented for the PTA assessment. Diagnostic accuracy was primarily assessed at the level of ear, as this chapter is principally concerned with differentiating impaired ears from hearing ears, but child-level analyses were also reported, as those estimates are most relevant for the economic analyses reported in *Chapter 8*. Our primary analysis included ears and children regardless of whether they had nominally been recruited as cases or controls. The inclusion of all ears/children is appropriate for assessing the extent to which the screening tests can differentiate the presence of hearing impairment from no hearing impairment across all children. However, the secondary analyses that include only impaired ears/children who were nominally recruited as controls may provide estimates of accuracy that are more pertinent for assessing the value of such tests in the context of a SES programme, where we are looking to identify children with previously unsuspected impairment.

Confidence intervals for the accuracy measures at ear level indicate that the sensitivity is likely to be no lower than 89% for the PTS and 83% for the HC screener, and that the specificity is no lower than 78% for the PTS and 83% for the HC screener. There was evidence at the 5% level that the sensitivity is greater for the PTS than the HC screener and that the specificity is greater for the HC screener than for the PTS. Considering only the results presented in this chapter and not taking into account other issues considered in the cost-effectiveness analyses presented later in this report (see *Chapter 8*), the value of the SES tests rests on the minimum accuracy level that makes them worthwhile in practice. The choice between the screening methods hinges on the importance of the test being sensitive relative to the importance of it being specific – in other words whether false negatives (children with a hearing impairment who pass the screening tests) are a more important problem than false positives (children with no hearing impairment referred by the screening tests). Reducing the false-negative fraction (i.e. increasing sensitivity) is more salient because, ultimately, the priority is identifying impaired children rather than confirming the status of children without impairment. On this basis one might lean towards the PTS as the 'better' test. It should be noted that the additional impaired children who are identified as a result of the increased sensitivity are likely to be at the less severe end of impairment and that there are therefore diminishing returns in improved identification.

For peer review only - http://bmjopen.bmj.com/site/about/guidelines.xhtml

DOI: 10.3310/hta20360 HEALTH TECHNOLOGY ASSESSMENT 2016 VOL. 20 NO. 36

Another consideration in the interpretation of the accuracy estimates is to take account of the effect of the prevalence of hearing impairment. This is particularly important in diagnostic case–control studies where the study prevalence of hearing loss is artificially determined (*Table 17*). For this we have assumed a prevalence of hearing loss typical of the pre and early school period as identified for the purposes of the health economic model in *Chapter 8* – 47 in every 10,000 children, which we have rounded up to 50 in every 10,000 children to simplify the 2 x 2 table. We applied this prevalence to the primary analysis accuracy estimates at child level for a PTA score of ≥ 30 dB on at least one frequency, PTS 95.9% sensitivity and 79.8% specificity, and HC screener 88.7% sensitivity and 83.8% specificity.

This allows quantification of the benefit of the PTS relative to the HC screener arising from improved sensitivity in context, with four additional true positives identified for every 10,000 children screened – the number needed to screen to achieve one additional true positive is 2500. It also emphasises that where the prevalence of hearing impairment is low, the effect of changes in specificity is amplified. The specificity obtained for the screening tests suggests that considerable numbers of false-positive tests [referrals for a diagnostic evaluation with an audiologist (DEA) who turn out not to have hearing impairment] will be generated – 1612 in the case of the HC screener and 2010 in the case of the PTS. This might argue that specificity should be the metric that decides whether the PTS or the HC screener is preferable. However, as is discussed further in *Chapter 8*, the actual number of referrals observed in a health-care system with SES in place is considerably less than would be predicted considering just the specificity of the screening test in isolation (see *Table 21*).

Analyses directly comparing the results of the PTS and HC screener revealed some discordance in the pass/refer classification, that is, some children who passed the PTS were referred by the HC screener, and vice versa. This suggests that the tests may be making different types of errors and that the best aspects of both tests could be combined into a new or hybrid test that is more accurate than either of the individual existing methods. As well as using different methods, they also have a basic difference in that PTS tests four frequencies at one level and the operator has the option to repeat tones if he/she feels that the child, for instance, has lost concentration whereas the HC tests two frequencies at three levels and is automated. Each frequency is presented only once to the child and there is some regularity to the presentation of tones such that the next tone could be anticipated. The only tested frequency that the two tests have in common is 1 kHz. The PTS is applied under headphones, which means that the ear not being tested has some barrier to any sound being presented to the test ear. The HC screener is applied through a close-fitting cardboard cup to one ear but the other ear has no sound protection and might hear the sound applied to the test ear.

Strengths and limitations

The study has a number of strengths. It was carefully designed and conducted, and data analysis was rigorous and included a number of secondary (sensitivity) analyses across different definitions of impairment. A sample size calculation was conducted a priori and the recruited sample yielded narrower CIs (and thus less uncertainty) for the accuracy estimates than the estimates from the economic model in the 2007 report.[12]

TABLE 17 Two-by-two table of the PTS and HC test results for hypothetical screening population of 10,000 children where the prevalence of impairment is 0.5% and given the child-level accuracy estimates

	PTS analysis			HC analysis		
	Reference standard result			Reference standard result		
Test result	Refer	Pass	Total	Refer	Pass	Total
Refer	48	2010	2058	44	1612	1656
Pass	2	7940	7942	6	8338	8344
Total	50	9950	10,000	50	9950	10,000

© Queen's Printer and Controller of HMSO 2016. This work was produced by Fortnum *et al.* under the terms of a commissioning contract issued by the Secretary of State for Health. This issue may be freely reproduced for the purposes of private research and study and extracts (or indeed, the full report) may be included in professional journals provided that suitable acknowledgement is made and the reproduction is not associated with any form of advertising. Applications for commercial reproduction should be addressed to: NIHR Journals Library, National Institute for Health Research, Evaluation, Trials and Studies Coordinating Centre, Alpha House, University of Southampton Science Park, Southampton SO16 7NS, UK.

For peer review only - http://bmjopen.bmj.com/site/about/guidelines.xhtml

A weakness of the study is that it estimated and compared the diagnostic accuracy between the PTS and HC screener methods using a two-gate ('case–control') design.[32] Under this approach those with the target condition (HI) and those without (NHI) are sampled from separate sources. The impaired children were largely identified via audiology services and the hearing children were largely recruited through schools. The two-gate design contrasts with the single-gate (or cohort) design where both those with and without the target condition are sampled from a single source without any knowledge of their target condition status. The more traditional single-gate design would have been preferable, but the challenge with this approach is that, because the prevalence of hearing impairment is so low, a large number of children would need to be recruited to the study in order to ensure that there is a sufficient number with the target condition. Fewer than 1 in 2000 children screened at school entry will have sensorineural hearing impairment[8] and 160,000 school children would need to be approached and tested in order to identify the target of 80 cases originally proposed in this study. The anticipated size of the single-gate design test accuracy study challenges both its feasibility and value for money in this context. This is not an issue for the two-gate case–control design used here. The two-gate design, however, is known to be more prone to biased estimates of accuracy largely resulting from the fact that those with and without the target condition are sampled from separate sources rather than drawn from a single defined population. This is problematic when estimating the accuracy of each test but is a less salient issue when comparing accuracy between hearing screening tests since any biases operating will be experienced equally for each test assessed. With respect to differential accuracy, conclusions on whether one hearing screen is better than the other should remain robust.

The criteria used to recruit children in each gate of the design were not used as the reference standard to categorise the children according to impairment status. A consequence of this is that some of the participants who were nominally recruited as controls were categorised as having a hearing impairment on the PTA reference standard. Cases were recruited as having a bilateral or unilateral hearing impairment and hence some ears were categorised as hearing on the PTA reference standard. In this event, this was a strength of the study as the HI group included children with established hearing impairment as well as children with no previously identified hearing impairment. The latter group may have levels of impairment severity that are more representative of the type of children who one might seek to identify with hearing impairment using a school entry hearing screen. The recruitment of such children enabled us to carry out sensitivity analyses based on whether impaired children were known cases (and therefore had a permanent hearing impairment) or were not previously identified with a problem (and therefore probably included children with transient hearing impairment).

A higher-than-anticipated number of children nominally recruited as controls were shown to have a hearing impairment on PTA measurement. We suggest this may be caused by a bias towards participation in those families who had some concerns about their child's hearing and therefore saw the study as a means by which their child could be tested, even though Nottingham operates an open referral system offering an appointment without a referral from a GP.

For the children nominally recruited as controls (which made up most of the hearing group) the screening tests and the reference standard were administered on the same day. The children nominally recruited as cases (mostly children in the HI group), however, generally had the reference standard administered at a different time to the test; up to 62 weeks before the test and up to 24 weeks after the test. The HL of children with permanent hearing impairment, however, is likely to be stable or worsening and it is not possible for children who have permanent sensorineural hearing impairment at one point to not have that hearing impairment later on.

For peer review only - http://bmjopen.bmj.com/site/about/guidelines.xhtml

DOI: 10.3310/hta20360 HEALTH TECHNOLOGY ASSESSMENT 2016 VOL. 20 NO. 36

Results in comparison with other studies

Several studies assessing the accuracy of the PTS were identified in the 2007 HTA report[12] (see *Table 6*) and no accuracy studies of the PTS were identified in the update review. The sensitivity from these earlier studies ranged from 82% to 100% and specificity from 65% to 99%. Thus, the accuracy obtained in this study, sensitivity 94.2% (95% CI 89.0% to 97.0%) and specificity 82.2% (95% CI 77.7% to 86.0%) is consistent with the previous findings, and shows no evidence of the overestimation of accuracy, which is theoretically predicted by using a case–control methodology. This provides reassurance that the accuracy estimate obtained for the HC screener, particularly the relative accuracy of the HC screener against the PTS, is also robust, as relative accuracy is less likely to be vulnerable to biases associated with study design. This is helpful as an additional accuracy study was identified for a HC device relative to reference standard of PTA in the update review. Gloria-Cruz *et al.*[17] estimated sensitivity as 23% (95% CI 11% to 39%) and specificity as 97% (95% CI 96% to 98%). The study was conducted in the Philippines in a convenience sample of Grade 1 elementary school children from three metropolitan schools with a mean age of 7.6 years. The prevalence of hearing loss was approximately 5% (39/821). The circumstances are thus very different from our consideration of SES in the context of the UK, which is likely to explain the marked difference in accuracy between the estimates. Additionally, the devices were not identical in each study. The device used by Gloria-Cruz *et al.*[17] (HC navigator) used three frequencies rather than two and a higher threshold to define hearing impairment, which would decrease sensitivity and increase specificity.

As well as impacting on the validity of our accuracy estimates, comparison with other studies also impacted on our decision-making about the choice of accuracy estimates to be used in the base case for the health economic evaluation. In the current protocol the 2007 HTA study estimates were identified as the source of estimates for our base-case analysis. This decision seems to be justified both in terms of the internal validity of the estimates and their applicability relative to alternative sources of accuracy data identified.

For peer review only

© Queen's Printer and Controller of HMSO 2016. This work was produced by Fortnum *et al.* under the terms of a commissioning contract issued by the Secretary of State for Health. This issue may be freely reproduced for the purposes of private research and study and extracts (or indeed, the full report) may be included in professional journals provided that suitable acknowledgement is made and the reproduction is not associated with any form of advertising. Applications for commercial reproduction should be addressed to: NIHR Journals Library, National Institute for Health Research, Evaluation, Trials and Studies Coordinating Centre, Alpha House, University of Southampton Science Park, Southampton SO16 7NS, UK.

For peer review only - http://bmjopen.bmj.com/site/about/guidelines.xhtml

For peer review only

DOI: 10.3310/hta20360 HEALTH TECHNOLOGY ASSESSMENT 2016 VOL. 20 NO. 36

Chapter 4 False-negative results from screening tests

Introduction

An important issue to be addressed in any research on population identification is how many people with the target condition are not identified, that is, the false negatives from any test, screen or awareness programme. Such data are expensive to collect, involving whole population follow-up and only realistic in longitudinal whole-cohort studies.

In this particular screening scenario, true and false positives are relatively easy to confirm. A child who is referred by the screen and is subsequently found to have a hearing impairment is a true positive and a child who is referred by the screen but is subsequently found to have no hearing impairment is a false positive.

It is also possible for a child to pass the screen and be therefore a true or false negative. A true negative is a child who passes the screen and is found to have no hearing impairment. A false negative would be a child who passes the screen when they in fact did have a hearing impairment.

Delayed identification in children who pass the hearing screen but who do have a hearing impairment (false negatives) is a concern. The challenge is how to identify false negatives. As children who pass the screen are not routinely followed up, only by evaluating every child in the SES programme at a diagnostic centre could one assess the numbers who are true negatives and those who are false negatives.

With the numbers involved this is clearly not feasible. Instead noting children who are referred for audiological assessment at any time after they have undergone and passed the screen could derive an estimate of the false negatives. However, even if such children passed the screen and are subsequently found to have a hearing impairment there can never be any certainty that the hearing impairment was present on the day the child was screened. If it is a sensorineural impairment, it could have been acquired or have progressed beyond a level identifiable by the screen. If it is a transient conductive impairment, by its very nature, it is very likely not to have been present at the time of screening. Only a permanent conductive impairment caused by anatomical pathology could be presumed to have been present on both occasions.

One way to address these issues is through a review of the literature, looking specifically for robust reports of data that explore the impact on the family and the child's development and education, of an unidentified or late-identified hearing impairment. However, such studies are likely to be rare for the reasons outlined above.

In addition, from the data collected in the diagnostic accuracy study (see *Chapter 3*) we can report the number of false negatives, that is, children who passed either of the screening tests and were found to be HI by the reference standard (see *Chapter 5*). This was a departure from our original protocol, added at the suggestion of our study steering group.

The key issue to be addressed by the review and the data is the size of any reduction or increase in the number of children identified late in a system with and without SES, and how much benefit is likely to accrue through their earlier identification and management.

© Queen's Printer and Controller of HMSO 2016. This work was produced by Fortnum *et al.* under the terms of a commissioning contract issued by the Secretary of State for Health. This issue may be freely reproduced for the purposes of private research and study and extracts (or indeed, the full report) may be included in professional journals provided that suitable acknowledgement is made and the reproduction is not associated with any form of advertising. Applications for commercial reproduction should be addressed to: NIHR Journals Library, National Institute for Health Research, Evaluation, Trials and Studies Coordinating Centre, Alpha House, University of Southampton Science Park, Southampton SO16 7NS, UK.

For peer review only - http://bmjopen.bmj.com/site/about/guidelines.xhtml

Objectives

- To review literature on the impact of false-negative results from screening tests.
- To describe children with false-negative screening results in the diagnostic accuracy study.

Methods

The following electronic databases were searched from inception to May 2014: The Cochrane Library (via Wiley), MEDLINE and MEDLINE In-Process & Other Non-Indexed Citations (via Ovid), EMBASE (via Ovid), PsycINFO (via Ovid) and Science Citation Index (via Web of Science). Using the search terms 'false negative' and 'hearing' identified a total of 173 titles. Using broad inclusion criteria of reference to hearing screening, sensitivity, specificity and/or false negatives, two of the authors (HF and CH) reviewed the titles and selected 26 papers for further consideration of abstracts. Applying the same criteria to the abstracts, 13 papers were identified for full review.

From an additional search using the terms 'negligence' and 'hearing' we identified 88 titles of which three abstracts were identified for full review, one of which had previously been included. We therefore sought 15 papers[3,7,8,26,43–53] for full review. All but one[45] (published in 1981) were published between 1996 and 2014.

Data collected for children participating in the diagnostic accuracy study (see *Chapter 3*) who passed the screening tests but had a refer result on the reference standard were extracted and tabulated.

Results

Literature review

No studies were identified that reported false-negative data for the school entry hearing screen. Four of the studies reported comparisons of two different screening tests without reference standards,[44–47] two of the studies (identified from the search on negligence) reported no cases owing to missed diagnosis of hearing impairment,[48,49] and three included general discussion of the value of hearing screens beyond the neonatal period.[8,50,51] These nine studies were not reviewed further.

Geal-Dor *et al.*[52] reviewed the results of neonatal screening at birth and behavioural screening at 7–9 months for 1545 children in Israel. They identified 58 children who had passed both screening tests or had passed the only screening test they underwent who were later referred for audiological assessment between the ages of 1 month and 4 years. Thirty-four had age-appropriate thresholds. Of these, 18 were discharged and 16 belonged to high-risk groups and were followed up. All of the remaining 24 children had a conductive impairment. No cases of permanent hearing impairment were identified.

In a further sample of 49 children identified with permanent hearing impairment, the results of the two screening tests were reviewed. Although eight children passed one screen and were referred by the other, only one child passed both screening tests, a conservative false-negative rate of 2% (1/49).

In an 8-year follow-up of the cohort of 21,279 babies enrolled in the Wessex (UK) trial of neonatal screening,[3] two of the 31 children identified by the age of 8–10 years with a permanent bilateral hearing impairment ≥ 40 dB had passed the neonatal screen (false-negative rate 6%). Of the 28,172 children who did not undergo neonatal screening, 35 children were identified with a permanent bilateral hearing impairment ≥ 40 dB and six of these had passed the distraction screening test at 7–9 months (false-negative rate 17%). The authors note that seven children (who had failed early screens) had evidence of progression of hearing impairment and speculate that the eight children who passed the screens might also have a progressive impairment, implying that the negative results may not have been false negatives.

For peer review only - http://bmjopen.bmj.com/site/about/guidelines.xhtml

DOI: 10.3310/hta20360 HEALTH TECHNOLOGY ASSESSMENT 2016 VOL. 20 NO. 36

Lo *et al.*[53] compared a parental questionnaire with PTA results in 6- to 7-year-old Chinese children in Hong Kong. They report that parents were able to identify only 20% of the cases of OME (no permanent hearing impairments were identified).

A birth cohort of 49,335 children in Australia with no risk factors for hearing impairment at birth underwent the distraction test at 7–9 months and were followed up at age 6 years.[7] Of 45,078 children who passed the distraction test eight were later identified to have a bilateral hearing impairment > 40 dB HL and fitted with hearing aids. The authors report a false-negative rate of 0.02% (8/45,078). If we assume those who failed the distraction test had a hearing impairment, the true false-negative rate would be 0.19% (8/4257 + 8); however, this assumption cannot be validated.

A study of college students (> 18 years) in the USA[43] noted that the false-negative rate was higher when the reference standard included all frequencies rather than the frequencies at which the screening was performed. However, they also note that the major reason for this was the presence of hearing impairment at high frequency (6 kHz) in these young adults, possibly caused by leisure noise. This is an important issue and is particularly relevant for this project when evaluating the HC screener (1 and 3 kHz) against gold standard PTA at 0.5, 1, 2 and 4 kHz.

Soares *et al.*[26] compared the results of a new AABR screener against PTA in children and young adults aged from 3 to 22 years in Japan. For the pre-school children aged 3–5 years the false-negative rate was zero but increased to 3% (3/108) for ages 6–17 years and 12% (2/17) for 18- to 22-year-olds.

Diagnostic accuracy study (see Chapter 3)

For control children in the diagnostic accuracy study, if a screening test (either PTS or HC) was passed but the PTA result indicated a hearing impairment, the result of the screening test is defined as a false-negative result. Sixteen ears of 16 children met this definition. Each ear underwent two screening tests, so the 16 ears underwent 32 screening tests. Of these, 22 tests were passed (false negatives) and 10 tests gave a refer results (true positives).

Of the 22 false-negative test results, eight passed only the HC, two passed only the PTS and six passed both screening tests (*Figure 14*).

Of the six ears that passed both screening tests, all had a PTA score of ≥ 30 dB on at least one of the four frequencies, 0.5, 1, 2, or 4 kHz, and three of those had a PTA score of ≥ 30 dB averaged across the four frequencies.

Of the eight ears that passed the HC but referred on the PTS, all had a PTA score of ≥ 30 dB on at least one of the four frequencies, 0.5, 1, 2, or 4 kHz, and two of those had a PTA score of ≥ 30 dB averaged across the four frequencies.

Of the two ears that passed the PTS but referred on the HC, both had a PTA score of ≥ 30 dB on at least one of the four frequencies, 0.5, 1, 2, or 4 kHz, and one of those had a PTA score of ≥ 30 dB averaged across the four frequencies.

All children for whom there was a refer result on the PTA (regardless of screening test results) were offered referral for further assessment. Of the 16 children with false-negative screening test results on one ear, one child was not referred because the result was considered to be because of a lack of concentration and one did not attend the appointment. Of the remaining 14, 10 were found to have no hearing impairment and discharged, presumably because a transient hearing impairment had resolved. Three children who passed the HC test but referred on the PTS test were found to have mild impairments and the one child who passed both screening tests was found to have a mild impairment.

© Queen's Printer and Controller of HMSO 2016. This work was produced by Fortnum *et al.* under the terms of a commissioning contract issued by the Secretary of State for Health. This issue may be freely reproduced for the purposes of private research and study and extracts (or indeed, the full report) may be included in professional journals provided that suitable acknowledgement is made and the reproduction is not associated with any form of advertising. Applications for commercial reproduction should be addressed to: NIHR Journals Library, National Institute for Health Research, Evaluation, Trials and Studies Coordinating Centre, Alpha House, University of Southampton Science Park, Southampton SO16 7NS, UK.

For peer review only - http://bmjopen.bmj.com/site/about/guidelines.xhtml

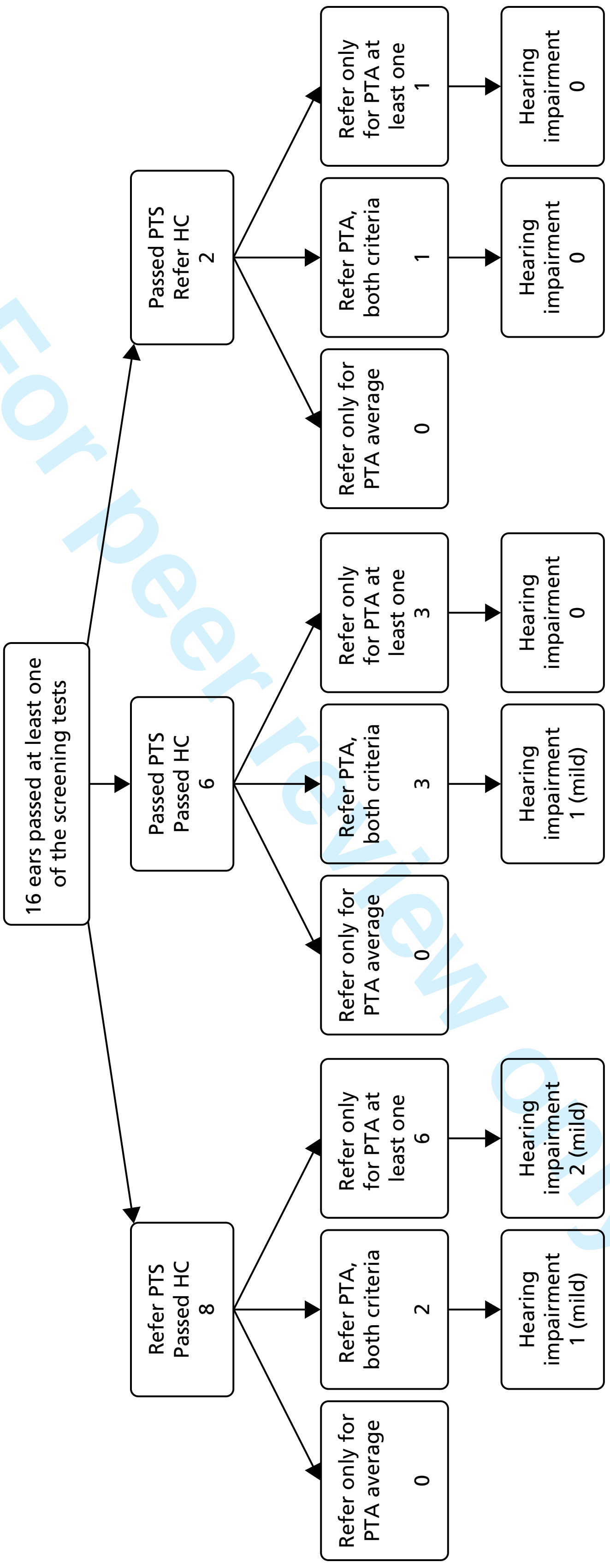

FIGURE 14 False negatives from diagnostic accuracy study (PTA average criterion: average of four frequencies is ≥ 30 dB. PTA at least one criterion: at least one of four frequencies is ≥ 30 dB).

For peer review only

For peer review only - http://bmjopen.bmj.com/site/about/guidelines.xhtml

DOI: 10.3310/hta20360 HEALTH TECHNOLOGY ASSESSMENT 2016 VOL. 20 NO. 36

Discussion

We were unable to identify studies that reported false-negative data from the school entry hearing screen. Across five different screening settings (neonatal at birth, distraction test at 8 months, parent questionnaire at 6–7 years, AABR at age 3–22 years, and the PTS at age 18–49 years), false-negative rates varied from 0.2% to 20%. All of the studies included in this review note the difficulty in assessing false negatives across evaluations separated in time for a condition that can fluctuate (transient conductive impairments) or progress or is of later onset (sensorineural impairments). The presence of a hearing impairment at a later time in an individual who passed a screening test at an earlier time can never be determined with certainty to be a false-negative result of the screen.

For the children nominally recruited as controls in the diagnostic accuracy study, the screening tests and the reference standard were conducted on the same day and the false-negative results could be considered to be more certain. The results of the further assessment which was conducted some weeks later indicate that most of the impairments identified by the reference standard on the day of the screening tests were transient and improved before the child attended the later appointment.

Owing to the lack of robust published data on false-negative results from SES, we did not take the results of this review further in terms of the economic modelling (see *Chapter 8*).

For peer review only

© Queen's Printer and Controller of HMSO 2016. This work was produced by Fortnum *et al.* under the terms of a commissioning contract issued by the Secretary of State for Health. This issue may be freely reproduced for the purposes of private research and study and extracts (or indeed, the full report) may be included in professional journals provided that suitable acknowledgement is made and the reproduction is not associated with any form of advertising. Applications for commercial reproduction should be addressed to: NIHR Journals Library, National Institute for Health Research, Evaluation, Trials and Studies Coordinating Centre, Alpha House, University of Southampton Science Park, Southampton SO16 7NS, UK.

For peer review only - http://bmjopen.bmj.com/site/about/guidelines.xhtml

For peer review only

DOI: 10.3310/hta20360 HEALTH TECHNOLOGY ASSESSMENT 2016 VOL. 20 NO. 36

Chapter 5 Comparison of a site with a school hearing screening programme (Nottingham) with a site without a school hearing screening programme (Cambridge)

Introduction

One alternative to the standard SES programme would be to not have a programme at all. The systematic review in the 2007 HTA report[12] described one poor quality study that compared screening with no screening, but the results were inconclusive.[16] The updated systematic review reported here in *Chapter 2* failed to identify any further studies.

In 2005, one in eight of all services in the UK responsible for implementing a universal school entry hearing screen were no longer doing so.[12] Approximately half of those services ran no screen and the remaining half offered a targeted screen for children identified as most at risk. The reasons given for not running a universal screen were based mainly on practical issues, including lack of resources, rather than any research-based evidence. There is no evidence whether implementing a screen at school entry leads to a heightened awareness and hence no failure to identify hearing impairment in children, or whether it results in children, who would otherwise be identified by the screen, slipping through the net and ultimately either never being identified or being identified much later in their school career.

In this chapter we compare two areas of the UK, one with a standard SES programme (Nottingham) and one without such a programme (Cambridge).

Objectives

- To compare children referred for investigation of suspected hearing impairment in a geographical area that applies routine SES (Nottingham) with a service with no routine SES (Cambridge) with respect to the number of referrals, the age at referral, the source of referral, the route through assessment to intervention, the number of children ultimately identified to have a hearing impairment (yield) and the nature of hearing impairment identified.

Methods

Routine data were analysed to compare data on referrals between a site with SES (Nottingham) and a site without SES (Cambridge).

Background to study sites

Site with a school screen (Nottingham)

The CHAC is a third-tier service and is part of Nottingham Audiology Services within Nottingham University Hospitals NHS Trust. There is no separate second-tier audiology service for children within Nottingham, therefore, all children referred are seen within the same service. CHAC provides services for all children up to the age of 16 years (19 years if in special education) within the catchment area of Nottingham City, Rushcliffe, Erewash, Ashfield, Broxtowe and Gedling. It also accepts referrals from surrounding areas for

© Queen's Printer and Controller of HMSO 2016. This work was produced by Fortnum *et al.* under the terms of a commissioning contract issued by the Secretary of State for Health. This issue may be freely reproduced for the purposes of private research and study and extracts (or indeed, the full report) may be included in professional journals provided that suitable acknowledgement is made and the reproduction is not associated with any form of advertising. Applications for commercial reproduction should be addressed to: NIHR Journals Library, National Institute for Health Research, Evaluation, Trials and Studies Coordinating Centre, Alpha House, University of Southampton Science Park, Southampton SO16 7NS, UK.

For peer review only - http://bmjopen.bmj.com/site/about/guidelines.xhtml

For peer review only

specialised services. The service is led by a band 8a Clinical Scientist (CB) and has six whole-time equivalent (WTE) audiological staff ranging from band 6 to 8a. CHAC has an open referral policy and therefore accepts referrals from parents as well as from all professionals. It works closely alongside the ENT Department at Nottingham University Hospitals and children move between the two services depending on their needs. The service has around 3400 new referrals a year with a total number of 5900 patient appointments on average.

The service assesses children's hearing according to national and local protocols and provides counselling and advice to parents with any concerns regarding their child's hearing. If a child is identified as having a transient conductive impairment, they are referred onto ENT as appropriate after monitoring. For those children identified as having a permanent impairment and those children with a transient conductive impairment whose parent(s) choose not to have surgical management, the service provides hearing aids and ongoing habilitation (*Figure 15*).

All schools within the Nottingham City and County authorities carry out the school entry hearing screen as part of the school entry health screen.

Site with no school screen (Cambridge)

The Cambridge service is a second-tier service provided by Cambridge Community Paediatric Audiology Service, which is part of the Cambridgeshire Community Services (CCS) NHS Trust. It receives approximately 1800 new referrals per year with a total of approximately 2400 appointments annually. The service provides second-tier hearing assessment for children aged 7 months to 16 years (19 years if in special education) and information and education to carers and professionals. The catchment area covers children living in, attending school in or with a GP in Cambridge City, or South and East Cambridgeshire. The service is provided by two community paediatricians (total 1.3 WTE, one of which is clinical lead for the service), one WTE band 7 lead clinical scientist (Audiology) (JM), members from the Addenbrooke's Paediatric Audiological Team who provide sessional coverage, and administrative support provided by members of the Clinical Support Team attached to Children's Services within CCS.

The service is provided within family-friendly environments located in the community. A community paediatrician and an audiologist usually run each clinic by working together, using their own areas of expertise, to look at the whole child.

Referrals are made to the service from GPs, child and family nurse team/health visitors, speech and language therapists, paediatricians, education, social services, and the Newborn Hearing Screening Programme (NHSP). When a chronic or permanent hearing impairment is identified the service aim is to facilitate further assessment and management within third-tier services, that is, the tertiary audiology service at Addenbrooke's Hospital, the ENT department at Addenbrooke's Hospital, and speech and language therapy.

The role of the service is to assess children's hearing, according to national and local protocols, for children referred due to carer or professional concern. It also includes assessment of children who have been identified to be 'at risk' by the NHSP despite clear responses on the screen. The main condition seen in the second tier is transient conductive hearing impairment associated with middle ear effusion or 'glue ear'. The second tier provides carers and professionals with information and education regarding this type of hearing impairment and good strategies for supporting the child. If the middle ear effusion or conductive hearing impairment is persistent there are agreed written protocols for referring these children to the ENT Department at Addenbrooke's Hospital. If a child is identified as having a sensorineural (unilateral or bilateral) hearing impairment, the child will be referred to the Tertiary Paediatric Audiology Service at Addenbrooke's Hospital for diagnostic assessment and management; this includes any child where it has not been possible to exclude a sensorineural hearing impairment. Referrals to the second tier are often made in order to exclude a hearing impairment as a contributing factor to educational, behavioural and/or speech and language concerns. This includes children who may go on to be identified as being on the autistic spectrum. A diagrammatic representation of the pathway of care for Cambridge is shown in *Figure 16*.

For peer review only

For peer review only - http://bmjopen.bmj.com/site/about/guidelines.xhtml

DOI: 10.3310/hta20360 HEALTH TECHNOLOGY ASSESSMENT 2016 VOL. 20 NO. 36

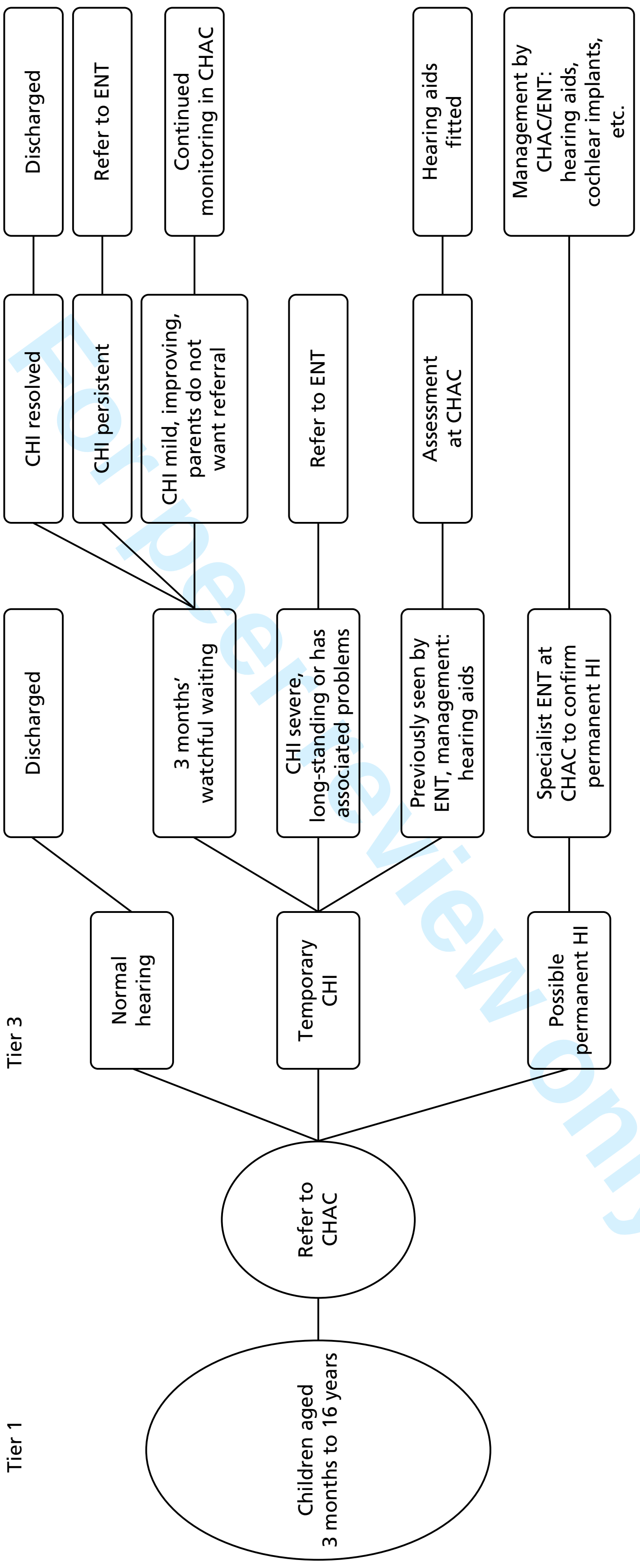

FIGURE 15 Pathway for Nottingham patients. CHI, conductive hearing impairment.

For peer review only

© Queen's Printer and Controller of HMSO 2016. This work was produced by Fortnum *et al.* under the terms of a commissioning contract issued by the Secretary of State for Health. This issue may be freely reproduced for the purposes of private research and study and extracts (or indeed, the full report) may be included in professional journals provided that suitable acknowledgement is made and the reproduction is not associated with any form of advertising. Applications for commercial reproduction should be addressed to: NIHR Journals Library, National Institute for Health Research, Evaluation, Trials and Studies Coordinating Centre, Alpha House, University of Southampton Science Park, Southampton SO16 7NS, UK.

For peer review only - http://bmjopen.bmj.com/site/about/guidelines.xhtml

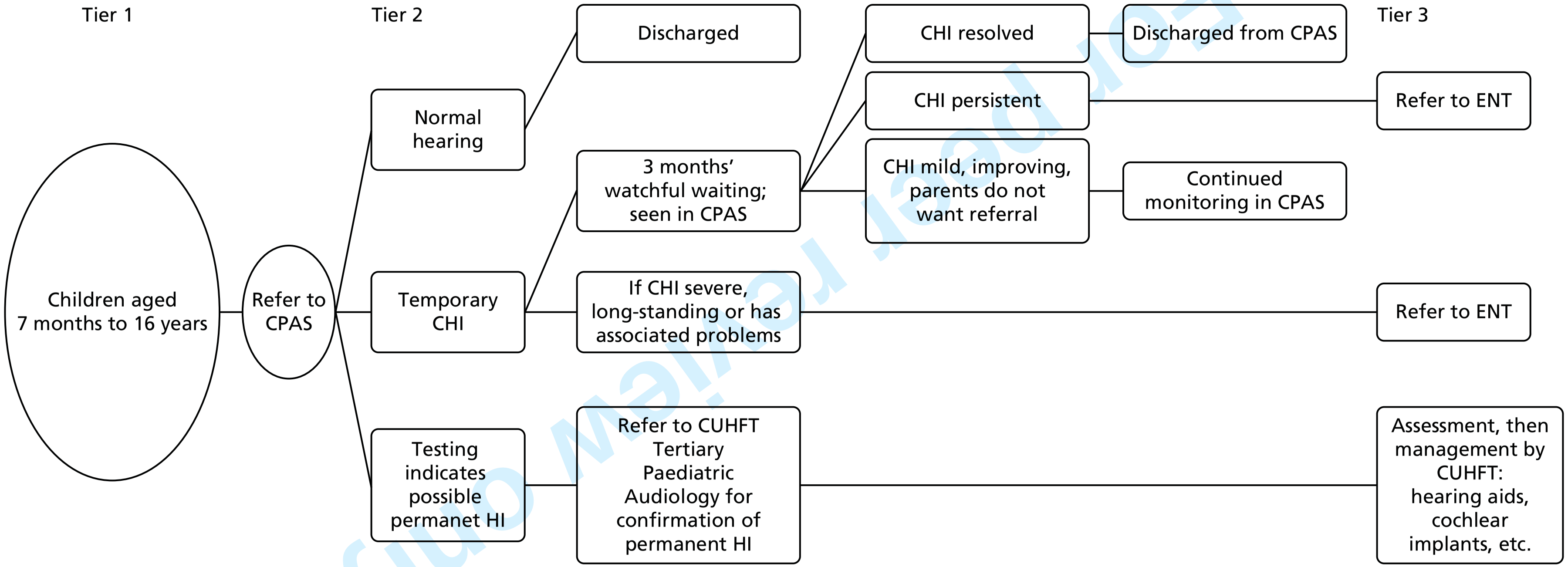

FIGURE 16 Pathway for Cambridge patients. CPAS, Community Paediatric Audiology Service; CHI, conductive hearing impairment; CUHFT, Cambridge University Hospitals Foundation Trust (third-tier hospital).

For peer review only - http://bmjopen.bmj.com/site/about/guidelines.xhtml

DOI: 10.3310/hta20360 HEALTH TECHNOLOGY ASSESSMENT 2016 VOL. 20 NO. 36

There has been no SES in Cambridge City, South and East Cambridgeshire since 1997, when the health visitor distraction testing was abolished in this area. Reasons for stopping included cost, variability of practice and lack of strong guidance from the Department of Health on what should be provided for SES and how it should be implemented. Community Paediatric Audiology in CCS was set up in 1997 with a strong campaign to first-tier services (GP, speech and language therapists, etc.) informing them that there would be no routine hearing tests in Cambridgeshire and emphasising the importance of referring to second-tier services if there are parental and professional concerns. This involved letters to all GP practices and health visiting teams, and speech therapy services outlining the changes in provision of routine hearing tests, and posters in GP practices and speech therapy clinics, highlighting the presenting symptoms of glue ear.

Data collection

Data were collected for children aged between 3 years and 6 years 364 days who were referred to Nottingham paediatric audiology or Cambridge audiology services by any source other than the UNHS. All referrals between 1 September 2012 and 30 June 2014 were included. Data on follow-up appointments that took place up to 30 September 2014 were included.

We originally proposed to explore retrospective data collected in Cambridge from 2007 to 2012. However, as those data were not collected with the objectives of the research in mind, much information was missing and it was decided that analyses would not add constructively to the project.

The collaborating audiologists for the areas of Nottingham (site with SES) and Cambridge City, and South and East Cambridgeshire (site without SES) collected data on referrals. Further data were collected via questionnaire from parents of children referred from the SES programme to the Nottingham service (see *Chapter 6*). Prospective data were processed using a database built by PenCTU.

Audiologists entered data from patient notes. In Nottingham, the waiting list co-ordinator identified eligible children, and audiologists within the service with permission to access patient records entered the data. In Cambridge assistance was also provided by one of the Nottingham researchers. Data were entered for the complete care pathway, from referral through to discharge (or 30 September 2014, whichever occurred first) including: date of birth; postcode; date of referral; location of clinic; referral source (GP, health visitor, school screen, etc.) date of appointment(s); type of assessment (e.g. visual reinforcement audiometry, play audiometry in the soundfield, PTA); result of assessment [normal bilateral, normal soundfield (better ear) thresholds, unilateral sensorineural, bilateral sensorineural, unilateral conductive, bilateral conductive, mixed bilateral, incomplete]; tympanometry results; hearing thresholds/minimal response levels; probable cause of impairment (if known); end of care (yes/no); and outcome [discharge, referral to ENT, referred for diagnostic confirmation (Cambridge only), hearing aids fitted (Nottingham only)].

Each child's records were accessed by audiologists authorised to look at them, hence consent was not required from individual patients. Anonymised data were entered onto the database. These procedures received ethical approval. Referral data were checked and corrected for implausible values. Staff time to undertake a planned second data extraction check of 10% was severely restricted by the pressure of service delivery. Undertaking this data check was not possible with the staff resources available without causing a significant delay to the study reporting. Copies of the questionnaires were sent to PenCTU for second data entry and double entry data checking.

© Queen's Printer and Controller of HMSO 2016. This work was produced by Fortnum *et al.* under the terms of a commissioning contract issued by the Secretary of State for Health. This issue may be freely reproduced for the purposes of private research and study and extracts (or indeed, the full report) may be included in professional journals provided that suitable acknowledgement is made and the reproduction is not associated with any form of advertising. Applications for commercial reproduction should be addressed to: NIHR Journals Library, National Institute for Health Research, Evaluation, Trials and Studies Coordinating Centre, Alpha House, University of Southampton Science Park, Southampton SO16 7NS, UK.

For peer review only - http://bmjopen.bmj.com/site/about/guidelines.xhtml

Statistical analysis

The yield, rate of referral and age at referral were compared between the site with a SES programme (Nottingham) and the site without a SES programme (Cambridge).

Yield was defined as the number of children between their third and seventh birthdays identified as having hearing impairment whose date of referral was between 1 September 2012 and 30 June 2014 (the study period) per 1000 person-years at risk. Hearing impairment included transient conductive and permanent sensorineural or conductive hearing impairments. The rate of referral was defined as number of referrals for suspected hearing impairment in the same period per 1000 person-years at risk. Some referrals resulted in more than one appointment. Children for whom the outcome of the last appointment was further referral or hearing aid were considered to have hearing impairment and included in the numerator in the calculations for yield; children discharged at the last appointment were considered to have no hearing impairment. In order to calculate the denominator for yield and the referral rate, the population size in each site was obtained from the Office for National Statistics mid-2013 estimates[54] of the population who were aged 3, 4, 5 or 6 years in the study sites. The Nottingham site included referrals from the local authorities of Nottingham, Erewash, Ashfield, Broxtowe, Gedling and Rushcliffe; the Cambridge site included Cambridge, East Cambridgeshire and South Cambridgeshire. The number of person-years observed was calculated by multiplying the population size by the number of days during the study period (668 days) and dividing by the mean number of days in a year (365.25 days). The two sites were compared with respect to yield and referral rate using the rate ratio, reported with 95% CI and *p*-value.

The *t*-test was used to compare the mean age at referral between the Nottingham and Cambridge sites (1) for all initial referrals; and (2) for confirmed HI cases only.

We report the percentage of referrals that resulted in the identification of HI cases for: (1) Nottingham referrals that were via a school screen; (2) Nottingham referrals that were via any other source (e.g. GP, speech therapist); and (3) Cambridge referrals.

Finally, we report the median (IQR) level of hearing impairment in dB at each of four frequencies (0.5, 1, 2 and 4 kHz) in each ear and the source of referral (using numbers and percentages) for both Nottingham and Cambridge. These variables were summarised for all referrals and then for the subset of referrals that resulted in the identification of children with impaired hearing.

Analyses were carried out using Stata statistical software.

Results

Referral rate and yield

There were 1702 referrals in Nottingham (21.9 referrals per 1000 person-years) and 1108 in Cambridge (34.4 referrals per 1000 person-years); the referral rate in Nottingham was two-thirds that of Cambridge (rate ratio 0.64, 95% CI 0.59 to 0.69; $p < 0.001$) (*Table 18*). Hearing impairment was confirmed in 195 children in Nottingham (yield of 2.51 cases per 1000 person-years) and 98 children in Cambridge (3.04 cases per 1000 person-years). There was little evidence that the yield is different between Nottingham and Cambridge (rate ratio 0.82, 95% CI 0.64 to 1.06; $p = 0.12$). Confirmed hearing loss cases made up 17.0% of referred children in Nottingham (25.2% of children who were referred via SES and 14.9% of children referred via other sources) and 10.6% of referred children in Cambridge (*Table 19*).

For peer review only

For peer review only - http://bmjopen.bmj.com/site/about/guidelines.xhtml

DOI: 10.3310/hta20360 HEALTH TECHNOLOGY ASSESSMENT 2016 VOL. 20 NO. 36

TABLE 18 Comparison of referral and yield rates (as at last appointment) between Nottingham and Cambridge expressed per 1000 children per year

Outcome	Nottingham estimate	Cambridge estimate	Nottingham relative to Cambridge		
			Rate ratio	95% CI	*p*-value
Number of referred children per 1000 children per year	21.9	34.4	0.64	0.59 to 0.69	< 0.001
Number of confirmed cases (yield) per 1000 children per year	2.51	3.04	0.82	0.64 to 1.06	0.12

TABLE 19 Percentage of referred children who were subsequently confirmed as cases (at last appointment), stratified by site and, for Nottingham, whether or not referral was via SES

Site/referral source	% (*n*/*N*)	95% CI
Nottingham – referred via school screen	25.2% (60/238)	19.8% to 31.2%
Nottingham – referred via other source	14.9% (135/907)	12.6% to 17.4%
Cambridge	10.6% (98/923)	8.7% to 12.8%

The mean age of referral was 4.7 years for both the Nottingham and Cambridge sites, but the mean age at referral for children who were subsequently confirmed as HI was higher in Nottingham than Cambridge (5.0 years vs. 4.5 years; mean difference 0.47 years, 95% CI 0.24 to 0.70 years; $p < 0.001$) (*Table 20*).

The characteristics are summarised in *Table 21* for all referred children and in *Table 22* for children confirmed as HI cases, separately for each of the Nottingham and Cambridge sites. In Nottingham 21.5% of all referrals and 30.8% of the confirmed HI cases were originally referred via SES. Other key sources of referral in Nottingham were ENT consultants (23.6% of confirmed cases), parents (11.8% of confirmed cases), GPs (10.8% of confirmed cases) and health visitors (10.8% of confirmed cases). In the Cambridge site the key sources of referral for confirmed HI cases were GPs (64.3%), health visitors (21.4%) and speech therapist (12.2%).

TABLE 20 Comparison of the mean age of referral (in years) between Nottingham and Cambridge

Participants	Nottingham		Cambridge		Difference (Nottingham – Cambridge)		
	n	Mean, years (SD)	*n*	Mean, years (SD)	Mean, years	95% CI, years	*p*-value
All referred children	1702	4.70 (1.01)	1108	4.66 (1.08)	0.04	–0.04 to 0.11	0.37
Confirmed cases only	195	4.97 (0.96)	98	4.51 (0.94)	0.47	0.24 to 0.70	< 0.001

© Queen's Printer and Controller of HMSO 2016. This work was produced by Fortnum *et al.* under the terms of a commissioning contract issued by the Secretary of State for Health. This issue may be freely reproduced for the purposes of private research and study and extracts (or indeed, the full report) may be included in professional journals provided that suitable acknowledgement is made and the reproduction is not associated with any form of advertising. Applications for commercial reproduction should be addressed to: NIHR Journals Library, National Institute for Health Research, Evaluation, Trials and Studies Coordinating Centre, Alpha House, University of Southampton Science Park, Southampton SO16 7NS, UK.

For peer review only - http://bmjopen.bmj.com/site/about/guidelines.xhtml

TABLE 21 Characteristics of children referred

Characteristic/summary	Nottingham	Cambridge
Number of children referred	1702	1108
Number of confirmed cases (yield)	195	98
Base population	42,553	17624
Person-years at risk for base population	77,825	32,232
Children referred per 1000 person-years at risk	21.9	34.4
Yield per 1000 person-years at risk	2.51	3.04
Age at referral in years, mean (SD)	4.7 (1.0)	4.7 (1.1)
Age at referral at last birthday		
3 years, *n* (%)	508 (29.8)	381 (34.4)
4 years, *n* (%)	496 (29.1)	316 (28.5)
5 years, *n* (%)	492 (28.9)	259 (23.4)
6 years, *n* (%)	206 (12.1)	152 (13.7)
Level of hearing impairment in dB HL[a]		
Left ear (0.5 kHz), median (IQR)	15 (15–25)	15 (10–20)
Left ear (1 kHz), median (IQR)	15 (10–20)	15 (10–20)
Left ear (2 kHz), median (IQR)	15 (10–20)	10 (10–20)
Left ear (4 kHz), median (IQR)	15 (10–20)	10 (10–20)
Left ear (average), median (IQR)	15 (11.3–20)	13.1 (10–20)
Right ear (0.5 kHz), median (IQR)	15 (15–25)	20 (10–20)
Right ear (1 kHz), median (IQR)	15 (10–20)	15 (10–20)
Right ear (2 kHz), median (IQR)	10 (10–20)	10 (10–20)
Right ear (4 kHz), median (IQR)	15 (10–20)	10 (10–20)
Right ear (average), median (IQR)	15 (10–20)	12.5 (10–20)
Source of referral		
GP, *n* (%)	186 (10.9)	458 (41.3)
Health visitor, *n* (%)	387 (22.7)	269 (24.3)
School screen, *n* (%)	366 (21.5)	N/A
Speech therapist, *n* (%)	147 (8.6)	278 (25.1)
Paediatrician, *n* (%)	137 (8.0)	35 (3.2)
Parent, *n* (%)	211 (12.4)	46 (4.2)
Education, *n* (%)	0 (0)	21 (1.9)
School nurse, *n* (%)	53 (3.1)	0 (0)
Community nursery nurse, *n* (%)	14 (0.8)	0 (0)
ENT consultant, *n* (%)	139 (8.2)	0 (0)
Other consultant, *n* (%)	28 (1.6)	0 (0)
NHSP, *n* (%)	0 (0)	1 (0.1)
Referrals from diagnostic accuracy study, *n* (%)	34 (2.0)	0 (0)

continued

For peer review only

For peer review only - http://bmjopen.bmj.com/site/about/guidelines.xhtml

DOI: 10.3310/hta20360 HEALTH TECHNOLOGY ASSESSMENT 2016 VOL. 20 NO. 36

TABLE 21 Characteristics of children referred (*continued*)

Characteristic/summary	Nottingham	Cambridge
Outcome of referral		
Discharge, *n* (%)	950 (83.0)	825 (89.4)
Further referral, *n* (%)	149 (13.0)	97 (10.5)
Hearing aid, *n* (%)	46 (4.0)	1 (0.1)
Missing, *n* (not reached end of care)	557[b]	185

N/A, not applicable.
a Sample size for these variables range from 915 to 941 in Cambridge and from 893 to 1050 in Nottingham.
b May be because of non-attendance at 'opt-in' review appointments.
Percentages may not add to 100% due to rounding.

TABLE 22 Characteristics of confirmed cases of hearing loss

Characteristic/summary	Nottingham	Cambridge
Confirmed cases		
Age at referral in years, mean (SD)	5.0 (1.0)	4.5 (0.9)
Age at referral at last birthday		
3 years, *n* (%)	35 (17.9)	32 (32.7)
4 years, *n* (%)	59 (30.3)	38 (38.8)
5 years, *n* (%)	70 (35.9)	22 (22.4)
6 years, *n* (%)	31 (15.9)	6 (6.1)
Level of hearing impairment in dB HL[a]		
Left ear (0.5 kHz), median (IQR)	40 (30–45)	30 (25–45)
Left ear (1 kHz), median (IQR)	35 (25–40)	30 (25–40)
Left ear (2 kHz), median (IQR)	30 (20–35)	25 (15–35)
Left ear (4 kHz), median (IQR)	35 (25–45)	35 (22.5–40)
Left ear (average), median (IQR)	35 (26.3–41.3)	31.3 (22.5–38.8)
Right ear (0.5 kHz), median (IQR)	35 (25–45)	35 (25–40)
Right ear (1 kHz), median (IQR)	35 (25–40)	35 (25–40)
Right ear (2 kHz), median (IQR)	30 (20–35)	25 (15–40)
Right ear (4 kHz), median (IQR)	35 (25–45)	32.5 (20–40)
Right ear (average), median (IQR)	32.5 (22.5–40)	31.3 (23.8–37.5)

continued

© Queen's Printer and Controller of HMSO 2016. This work was produced by Fortnum *et al.* under the terms of a commissioning contract issued by the Secretary of State for Health. This issue may be freely reproduced for the purposes of private research and study and extracts (or indeed, the full report) may be included in professional journals provided that suitable acknowledgement is made and the reproduction is not associated with any form of advertising. Applications for commercial reproduction should be addressed to: NIHR Journals Library, National Institute for Health Research, Evaluation, Trials and Studies Coordinating Centre, Alpha House, University of Southampton Science Park, Southampton SO16 7NS, UK.

For peer review only - http://bmjopen.bmj.com/site/about/guidelines.xhtml

TABLE 22 Characteristics of confirmed cases of hearing loss (*continued*)

Characteristic/summary	Nottingham	Cambridge
Source of referral		
GP, *n* (%)	21 (10.8)	63 (64.3)
Health visitor, *n* (%)	21 (10.8)	21 (21.4)
School screen, *n* (%)	60 (30.8)	N/A
Speech therapist, *n* (%)	7 (3.6)	12 (12.2)
Paediatrician	4 (2.1)	0 (0)
Parent, *n* (%)	23 (11.8)	2 (2.0)
Education, *n* (%)	0 (0)	0 (0.0)
School nurse, *n* (%)	9 (4.6)	0 (0)
Community nursery nurse, *n* (%)	0 (0)	0 (0)
ENT consultant, *n* (%)	46 (23.6)	0 (0)
Other consultant, *n* (%)	2 (1.0)	0 (0)
Referrals from diagnostic accuracy study, *n* (%)	2 (1.0)	0 (0)
Type of hearing impairment		
'Normal' binaural, *n* (%)[b]	7 (3.6)	2 (2.0)
Conductive impairment (bilateral), *n* (%)	138 (70.8)	70 (71.4)
Conductive impairment (unilateral), *n* (%)	40 (20.5)	20 (20.4)
Sensorineural impairment (bilateral), *n* (%)	1 (0.5)	2 (2.0)
Sensorineural impairment (unilateral), *n* (%)	5 (2.6)	1 (1.0)
Mixed impairment (unilateral), *n* (%)	0 (0)	2 (2.0)
Incomplete, *n* (%)	4 (2.1)	1 (1.0)

N/A, not applicable.
a Sample size for these variables range from 79 to 93 in Cambridge and from 146 to 179 in Nottingham.
b Absolute values of HL may be > 30 dB but soundfield testing would indicate that to be 'normal'.
Percentages may not add to 100% due to rounding.

Discussion

It might be expected that adding a screen to a system would result in a greater number of referrals. However, the observational comparison of two sites, one with SES (Nottingham) and one without SES (Cambridge), showed evidence that the rate of referral for hearing problems is lower when SES is present. The referral rate was 36% lower in Nottingham relative to Cambridge (rate ratio 0.64; $p < 0.001$) and the CI for the rate ratio indicates the true rate is at least 31% lower when there is SES.

In the SES site, one-third of children subsequently confirmed as cases were initially referred via SES. There was little evidence ($p = 0.12$) that the yield of confirmed cases is altered by SES; the estimated rate of confirmed cases was 18% lower in Nottingham relative to Cambridge but it is plausible within the bounds of the 95% CI that the true yield rate is the same in areas with and without SES. The CI, however, does indicate that it is unlikely that SES areas truly have a markedly higher yield rate. A higher proportion of referred children were subsequently confirmed to be HI in the area with SES (17.0% vs. 10.6%).

For peer review only - http://bmjopen.bmj.com/site/about/guidelines.xhtml

DOI: 10.3310/hta20360 HEALTH TECHNOLOGY ASSESSMENT 2016 VOL. 20 NO. 36

The mean age of referral was nearly identical (4.7 years) between the two sites when looking at all referrals. When focusing solely on children who were subsequently confirmed to have a hearing impairment, however, there was strong evidence that the children in the site with a screen were older at referral (5.0 years vs. 4.5 years; $p < 0.001$). One possibility is that, for children in this age range, parents/professionals in Cambridge seek referral when concerns are raised whereas in the area with SES, if concerns are raised around age 4 years, parents and professionals are aware that SES is coming up later that year and wait.

The CIs for the mean age difference indicates that the true mean age at referral in areas with a screen could plausibly be anywhere between 3 and 8 months greater than areas without one. Delay in identification of a hearing impairment has the potential to adversely affect development[1] but further research would be needed to evaluate the extent of the impact for children at school age.

There are also differences in the audiology services operated in the two areas. In Nottingham there is no second-tier audiology service and all referred children are seen within the same service. It has an open referral policy, which includes referrals from parents. It works closely with the ENT department and children move between the two services dependent on their needs. A health professional concerned that a child might have OME and hearing loss has the option to refer for a DEA or to ENT; ENT might then refer for a DEA if appropriate.

The second-tier service in Cambridge accepts referrals from health and education professionals. It provides assessment for children and onward referral for those children who may require hearing aids or surgical management. A health professional with a child with possible OME and hearing loss knows that there is a well-staffed intermediate (DEA) service which can effectively sieve referrals and send those needing ENT examination to ENT departments and those requiring hearing aids to third-tier audiology. Thus GPs will be likely to refer to second-tier audiology and referrals from ENT departments to second-tier audiology will be highly unlikely.

The very different numbers of children referred from different routes are likely to be a function of these different systems in the two areas. Parents can directly refer to the CHAC in Nottingham but in Cambridge they would have to go via a GP (or other professional). GPs in Cambridge would know that they have a second-tier community service which acts as a filter, so would have a tendency to refer there rather than to ENT or third-tier audiology. Hence referrals from ENT to second-tier audiology would be rare in Cambridge, but from ENT to the CHAC would be expected to be higher, as they are. Provision of hearing aids as an outcome is clear in the CHAC, but second-tier audiology services in Cambridge do not provide hearing aids.

Strengths and limitations

Our study had a number of strengths. Data collection in both sites was comprehensive and actively monitored by a senior member of the clinical staff with responsibility for audiological services in each of the two areas. Both were members of the research team. An electronic database used in both sites was developed by staff of PenCTU to standardise data collection.

However, our study design of an observational comparison of two areas [one that operates a SES programme (Nottingham) and the other that does not operate a SES programme (Cambridge)] is subject to major methodological limitations, in spite of our best attempts to choose two sites that were similar to each other. We acknowledge that there may be epidemiological and social differences between the two geographical areas that are likely to confound our findings. Reassuringly, population estimates indicate the proportion of children aged < 16 years to be similar in Nottinghamshire (18.7%) and in Cambridgeshire (18.5%).[54] However, the index of socioeconomic deprivation indicates the city of Nottingham (rank 17) to be more deprived on a range of measures than the Cambridge district (rank 188).[55] Given the lack of availability of child-level data, we were unable to adjust our analyses to take account of these and other potential confounders. Furthermore, given that we consider only two geographical areas, our results may not be considered as generalisable.

© Queen's Printer and Controller of HMSO 2016. This work was produced by Fortnum *et al.* under the terms of a commissioning contract issued by the Secretary of State for Health. This issue may be freely reproduced for the purposes of private research and study and extracts (or indeed, the full report) may be included in professional journals provided that suitable acknowledgement is made and the reproduction is not associated with any form of advertising. Applications for commercial reproduction should be addressed to: NIHR Journals Library, National Institute for Health Research, Evaluation, Trials and Studies Coordinating Centre, Alpha House, University of Southampton Science Park, Southampton SO16 7NS, UK.

For peer review only - http://bmjopen.bmj.com/site/about/guidelines.xhtml

Both sites mainly receive referrals from a defined geographical region but also accept referrals outwith that area. Equally, some children within the defined area may be referred elsewhere; the numbers are estimated to be few by the responsible audiologists. The referral catchment areas also do not exactly match the areas defined by the Office for National Statistics for the population estimates used and there may, therefore, be some minor imprecision in the population denominators used in the analyses.

For peer review only

For peer review only - http://bmjopen.bmj.com/site/about/guidelines.xhtml

DOI: 10.3310/hta20360 HEALTH TECHNOLOGY ASSESSMENT 2016 VOL. 20 NO. 36

Chapter 6 Exploring the impact on the child and family of a child being referred by the school hearing screen: findings from a questionnaire survey

Introduction

Any analysis of the cost-effectiveness of the screening process needs to consider the costs not only to the health and education services, but also to the families of children referred by the screen. Some of these children will be true positives (i.e. they have a confirmed hearing impairment) and many more will be false positives (identified by the screen but found to have no hearing impairment). Information to contribute to the cost-effectiveness model might include details of the amount of time and travel spent by families in attending the screen follow-up process but also an estimate of any anxiety caused. Our literature searches identified no prior information on the impact and costs of SES referral.

Objectives

- To determine the impact, both psychological and economic, for the child and the family of the child being referred for further assessment following the SES (both true and false positives).
- To collect cost data to inform the economic model.

Methods

Study population

Questionnaires (see *Appendix 5*) were distributed to parents of children referred to Nottingham CHAC from the school entry hearing screen between 1 September 2012 and 30 June 2014 (age range 4–6 years).

Questionnaire

The questionnaire captured data on aspects of the family's experience of the child being referred for a hearing assessment. Questions included how they found out about the screen and further testing, parent opinion about having a school hearing screen, the time taken to attend appointments, the cost of travel to appointments, and impact on work, school and social activities of attending clinic appointments. Data on gender, ethnicity and school attended were also requested. The level of anxiety experienced by the parent on finding out that their child needed further testing and again when they attended the appointment was rated by the parent on a scale from 0 (not at all anxious) to 10 (extremely anxious). There was also the possibility of an option to follow-up responses by telephone, with those who gave their contact details. No follow-up was carried out as it was not considered that it would add anything to the data already collected on the questionnaire, especially taking into account the bias of recall over a long time period and the small data set.

Data collection

Questionnaires plus pre-paid return envelopes were sent by the audiologist to the parents of all children referred to the CHAC by the school screen, once the end of care (discharge, hearing aid provision or referral to ENT) had been reached, whether or not they had a hearing impairment. The audiologist kept a list of questionnaires sent and which participant ID these related to. Parents who chose to take part returned the completed questionnaire to the researchers at NHBRU. These returns could be anonymous but questionnaire responders could also choose to be entered into a prize draw to win vouchers of their choice to a value of £50, or to agree to a possible follow-up interview and hence supply contact details.

© Queen's Printer and Controller of HMSO 2016. This work was produced by Fortnum *et al.* under the terms of a commissioning contract issued by the Secretary of State for Health. This issue may be freely reproduced for the purposes of private research and study and extracts (or indeed, the full report) may be included in professional journals provided that suitable acknowledgement is made and the reproduction is not associated with any form of advertising. Applications for commercial reproduction should be addressed to: NIHR Journals Library, National Institute for Health Research, Evaluation, Trials and Studies Coordinating Centre, Alpha House, University of Southampton Science Park, Southampton SO16 7NS, UK.

For peer review only - http://bmjopen.bmj.com/site/about/guidelines.xhtml

Nottingham researchers entered the data into the study database and copies of questionnaires (excluding the contact details page) were sent to PenCTU for second data entry and validation. A tracking log of the ID numbers of questionnaires returned was kept locally by researchers at NHBRU. A reminder and repeat questionnaire was sent after 3 months to non-responders identified from the list held by the audiologist.

Consent for the data collected in this study was implied by return of a completed questionnaire. It was explained to the parents completing the questionnaire that entry into the project was entirely voluntary and that the management and care of their child would not be affected by their decision on whether or not to take part.

Data analysis

The quantitative data were summarised using means with SDs or medians with IQRs.

To give structure to the qualitative data obtained from the questionnaires, a thematic analysis was carried out. Open comments from parents were assessed using a template analysis in which identified themes from each of the questionnaires were selected and ordered. Responses to the following questions were analysed using the template: (1) What are the good things about your child having their hearing checked at school?; (2) What are the not so good things about your child having their hearing checked at school?; and (3) Any further comments?

A five-step framework for analysing the questionnaire responses was used: familiarisation; identification of themes; indexing; charting; and interpretation.[56]

1. Familiarisation: each questionnaire was read through six times to get a feel for the data.
2. Identification of themes: the broad research question was to explore what parents thought about the SES tests. Using an inductive approach, parents' opinions were categorised into main themes that emerged across all the questionnaires. The researchers made notes on relevant points raised under each category. This process was repeated for all questionnaires and common themes were included in the final template. Ambiguous statements were not included as they were open to interpretation.
3. Indexing: subthemes based on similarities between all responses within the main identified categories were explored. Indexing involved generating more precise descriptions of the themes to make the analysis reader-friendly.
4. Charting: a template was drawn up for each category, theme and descriptor.
5. Interpretation: illustrative quotes were identified to provide the reader with examples of the parents' experiences.

Results

Questionnaires were sent to all 246 parents whose child was referred to the CHAC from the school entry hearing screen in the relevant period, and who reached end of care. Completed questionnaires were received from 60 parents by the end of the data collection period. No data were collected on the reasons for non-return.

Quantitative data

Of the 60 children for whom questionnaires were returned, 32 (53%) were female, 44 (73%) were white, 3 (5%) were mixed ethnicity, 10 (17%) were Asian, 1 (2%) was black/African/Caribbean and 2 (3%) were of other ethnic group.

Forty-five (75%) parents knew that their child was having their hearing checked at school. Parents found out that their child needed further testing by means of a letter either taken home ($n = 25$) or sent in the post ($n = 9$); by telephone ($n = 13$) or by other means ($n = 13$) [already noticed at home ($n = 5$), told by school ($n = 4$), told by school nurse ($n = 2$), told by doctor ($n = 1$), do not know ($n = 1$)].

For peer review only - http://bmjopen.bmj.com/site/about/guidelines.xhtml

DOI: 10.3310/hta20360 HEALTH TECHNOLOGY ASSESSMENT 2016 VOL. 20 NO. 36

Parental anxiety was scored on a 0–10 scale where 0 indicated 'not anxious at all' and 10 indicated 'extremely anxious'. The mean level of anxiety reported by the parent(s) on finding out that their child needed further testing was 5.3 (SD 2.1), and the median was 5 (IQR 4–7). At the clinic appointment the mean parental anxiety had reduced to 4.7 (SD 2.4); the median was 5 (IQR 3–6). Frequencies of anxiety are given in *Table 23*.

The same anxiety level on both occasions was reported by 36 out of 60 respondents (60%); 18 out of 60 (30%) reported feeling less anxious at the appointment and 6 out of 60 (10%) reported more anxiety at the appointment.

Most parents [51 out of 60 (85%)], strongly agreed that 'children should have their hearing checked at school', six (10%) agreed, one (2%) had no opinion, no one (0%) disagreed and one (2%) strongly disagreed (one missing).

Of the 60 questionnaires returned, parents reported that 25 children had only one appointment, 16 children had two appointments in total, nine had three appointments, five had four appointments, and five had five appointments. Altogether the 60 children had 129 appointments.

Time taken to get to an appointment was < 15 minutes for 24.8% (32/129) of appointments, about half an hour for 59.7% ($n = 77$) of appointments, about 1 hour for 14.0% ($n = 18$) of appointments and 1–2 hours for 1.6% ($n = 2$) of appointments. The length of appointment was < 30 minutes for 33.3% (43/129) of appointments, 30–60 minutes for 44.2% ($n = 57$) of appointments, about an hour for 13.2% ($n = 17$) of appointments, and 1–2 hours for 7.0% ($n = 9$) of appointments. Data on length of appointment were missing for three appointments.

For the journey to an appointment, 20.9% (27/129) included a bus or tram ride, 72.9% ($n = 94$) of journeys used a car, 5.4% ($n = 7$) included a taxi ride and one walked (0.8%). The median (IQR) total cost per appointment was £4.88 (£3.40–£8.80).

Parents were required to take either all or part of a day off work for 45.0% (58/129) of appointments; 29.5% ($n = 38$) of appointments were with parents who did not work and 25.6% ($n = 33$) of appointments did not require the parent to take time off work. The child was required to miss school for all or part of the day for 72.9% ($n = 94$) of appointments.

TABLE 23 Parental anxiety about school hearing screen referral

Anxiety level	Anxiety on finding out, *n* (%)	Anxiety at clinic appointment, *n* (%)
0 (not at all anxious)	1 (2%)	2 (3%)
1	3 (5%)	5 (8%)
2	2 (3%)	5 (8%)
3	5 (8%)	8 (13%)
4	5 (8%)	6 (10%)
5	16 (27%)	14 (23%)
6	10 (17%)	7 (12%)
7	10 (17%)	5 (8%)
8	6 (10%)	5 (8%)
9	0 (0%)	1 (2%)
10 (extremely anxious)	2 (3%)	2 (3%)

Percentages may not add to 100% due to rounding.

© Queen's Printer and Controller of HMSO 2016. This work was produced by Fortnum *et al.* under the terms of a commissioning contract issued by the Secretary of State for Health. This issue may be freely reproduced for the purposes of private research and study and extracts (or indeed, the full report) may be included in professional journals provided that suitable acknowledgement is made and the reproduction is not associated with any form of advertising. Applications for commercial reproduction should be addressed to: NIHR Journals Library, National Institute for Health Research, Evaluation, Trials and Studies Coordinating Centre, Alpha House, University of Southampton Science Park, Southampton SO16 7NS, UK.

For peer review only - http://bmjopen.bmj.com/site/about/guidelines.xhtml

Twelve out of the 60 (20%) children missed other activities, including ballet and music lessons but eight of these referred to missing school. Thirteen (21.7%) of the 60 parents missed other activities, although eight referred to work – probably a misunderstanding of the questions. Eight of 60 (13%) families said appointments caused problems for other family members by requiring additional childcare for other children.

Qualitative data

Table 24 lists the themes that emerged from the data and provides illustrative quotes.

TABLE 24 What parents think about the school entry hearing screen: thematic analysis

Main themes	Subthemes	Description	Example quotes	Respondent ID number
What are the good things about your child having their hearing checked at school?				
A1. Ease of testing	i. Convenience	Refers to the benefits to parents of the hearing test being carried out at school during school hours	*. . . in normal school hours*	1
			. . . without parents having to be proactive	1
			Don't need to take time from school to attend appointment	16
			Easier than trying to attend appointments	19
			It is local . . .	37
			. . . don't need to worry about . . . taking your child out of school to have their hearing tested	39
	ii. Familiarisation	Refers to how comfortable the child is having the hearing test in a location and with classmates that are familiar to them	*. . . feels normal to child as everyone has it done*	1
			Child not . . . upset as they are in a familiar environment	9
			Secure, familiar environment	14
			No anxiety for the child	34
			Child sees their peers having the same test	38
			All children tested together takes away. . . fear/anxiety	42

For peer review only

For peer review only - http://bmjopen.bmj.com/site/about/guidelines.xhtml

DOI: 10.3310/hta20360 HEALTH TECHNOLOGY ASSESSMENT 2016 VOL. 20 NO. 36

TABLE 24 What parents think about the school entry hearing screen: thematic analysis (*continued*)

Main themes	Subthemes	Description	Example quotes	Respondent ID number
A2. Identification of hearing impairment	i. Importance	Refers to how important it is to parents to have any hearing impairments in their children identified	*. . . they need to hear well in order to follow lessons*	4
			. . . the child may become disengaged with school/learning if their hearing is impaired	18
			. . . notifies the school so they become aware of [the] hearing issues . . . sooner	24
			It can rule out many other things	33
			[A] lack of hearing can have huge effects on child's ability and confidence . . .	40
	ii. Early detection	Refers to how the hearing test can enable potential hearing impairments to be picked up and treated quickly	*Any problems will be dealt with much quicker*	2
			. . . any problems . . . can be investigated at an early stage	3
			. . . parents can be alerted immediately	37
			. . . know early on that there is a problem and it can be sorted out	51
			They can be referred straight away for further tests	53
	iii. Acknowledgement	Refers to how a referral from the hearing screen at school supported suspicions that parent may already have had	*[My] concern was acknowledged*	10
			Confirmation from another source that there was a concern	11
			For us, having our school nurse check our daughter's hearing got her an appointment to get a proper check	23
			. . . backed up suspicions I already had regarding my daughter's hearing . . . and made me feel it was worthwhile going back to the GP . . .	50

continued

© Queen's Printer and Controller of HMSO 2016. This work was produced by Fortnum *et al.* under the terms of a commissioning contract issued by the Secretary of State for Health. This issue may be freely reproduced for the purposes of private research and study and extracts (or indeed, the full report) may be included in professional journals provided that suitable acknowledgement is made and the reproduction is not associated with any form of advertising. Applications for commercial reproduction should be addressed to: NIHR Journals Library, National Institute for Health Research, Evaluation, Trials and Studies Coordinating Centre, Alpha House, University of Southampton Science Park, Southampton SO16 7NS, UK.

For peer review only - http://bmjopen.bmj.com/site/about/guidelines.xhtml

TABLE 24 What parents think about the school entry hearing screen: thematic analysis (*continued*)

Main themes	Subthemes	Description	Example quotes	Respondent ID number
	iv. Otherwise missed	Refers to recognition from parents that any potential hearing problems may have been missed without the school hearing test	*. . . children of a young age are easily distracted and hearing problems won't be picked up readily as this [not hearing] would be considered the norm (as in my son's case)*	6
			. . . not noticed by home	8
			Could identify problems which are otherwise missed	15
			Often parents . . . dismiss things and . . . wouldn't follow up on possible concerns	41
What are the not so good things about your child having their hearing checked at school?				
B1. Communication with parents	i. Lack of information	Refers to parents being unaware of the screening test	*Parents not informed about how the test is done or how accurate the test is*	1
			I didn't know he was having this done	54
			I didn't know my child was having a test on that day until she came home and told me she had one	47
	ii. Poor correspondence	Refers to the dissatisfaction parents had with the correspondence after their child had been referred by the school hearing test	*The letter was very vague – it said she required further testing and it wasn't until I spoke to the school nurse that I established what the test had clinically shown. It was probably designed not to panic parents but I would have preferred to have been told*	50
			. . . unreliable test results, parents get informed there may be a problem when there isn't	15
			Got letter from school to say he would be retested but no date. The retest happened a long time after first test . . .	15

For peer review only

For peer review only - http://bmjopen.bmj.com/site/about/guidelines.xhtml

DOI: 10.3310/hta20360 HEALTH TECHNOLOGY ASSESSMENT 2016 VOL. 20 NO. 36

TABLE 24 What parents think about the school entry hearing screen: thematic analysis (*continued*)

Main themes	Subthemes	Description	Example quotes	Respondent ID number
B2. Parental absence	–	Refers to parents opinions about not being present while the school hearing test was undertaken	*Can't talk to the person carrying out the test about the results*	38
			Not being present to ask questions if test failed	12
			A parent is not around for when something (problem) arises	36
			I would have preferred to be present for checks	16
			Parents don't have the opportunity to be there	30
			Could provoke anxiety in child (no parent accompanying)	28
			Possibly the child may feel more comfortable with their parent being present	35
B3. Problems with the test	B3i. Distractions	Refers to testing children whilst they are at school may result in them being distracted from the test	*. . . may be distracted*	3
			Could be distracted if friends nearby or . . . at end of day and child not concentrating	9
	B3ii. Noise	Refers to testing children within noisy school environments rather than in sound proofed conditions	*The school is not the place to have a proper hearing test as they don't have a quiet room or the equipment to do so*	23
			Very noisy environment	26
			Not in a sound proof[ed] room	44

continued

For peer review only

© Queen's Printer and Controller of HMSO 2016. This work was produced by Fortnum *et al.* under the terms of a commissioning contract issued by the Secretary of State for Health. This issue may be freely reproduced for the purposes of private research and study and extracts (or indeed, the full report) may be included in professional journals provided that suitable acknowledgement is made and the reproduction is not associated with any form of advertising. Applications for commercial reproduction should be addressed to: NIHR Journals Library, National Institute for Health Research, Evaluation, Trials and Studies Coordinating Centre, Alpha House, University of Southampton Science Park, Southampton SO16 7NS, UK.

For peer review only - http://bmjopen.bmj.com/site/about/guidelines.xhtml

TABLE 24 What parents think about the school entry hearing screen: thematic analysis (*continued*)

Main themes	Subthemes	Description	Example quotes	Respondent ID number
Any further comments?				
C1. Age of the child	–	Refers to hearing tests being conducted at pre-school age (earlier than the current SES programme)	*My child had her pre-school check missed. This is why it was done at school*	13
			I agree children need a hearing test early and school is the only option currently. Would be better to be done before entering school at a proper hearing unit	22
			As a parent I think a child's hearing should also be checked as part of a pre-school check at the doctor's	23
C2. Satisfaction	i. With the follow-up service	Refers to positive comments on the service received after the school hearing test	*The staff of . . . was really good and made my daughter feel comfortable and less worried about the tests*	56
			I found the . . . centre brilliant and they put my mind at ease straight away	54
			Overall I'm very happy with the service we received. Everyone was really nice to my daughter and she wasn't worried about the appointments	47
			We have been happy with the whole process. The initial communication from the school test was informative and prompt. The tests were thoroughly explained to our child & to us, as were the results both immediately after the tests & via post a week later . . .	48
	ii. Overall	Refers to summaries about the school hearing screening test	*I think the pros outweigh the cons*	26
			School test cannot replace a proper hearing test as they cannot recreate that acoustic free environment but it can act as a good indicator of any problem area	55

Note
For simplification, when quoting: '. . .' is used when the authors excluded text from the original response and [ ] is used where text has been added in for clarification.

For peer review only

For peer review only - http://bmjopen.bmj.com/site/about/guidelines.xhtml

DOI: 10.3310/hta20360 HEALTH TECHNOLOGY ASSESSMENT 2016 VOL. 20 NO. 36

Discussion

Main findings

The consequences for costs and the views of parents concerning referral for diagnostic evaluation were assessed by means of a questionnaire sent to parents of children referred to the Nottingham audiological service by the SES programme. The response rate was 24.4% (60/246). It is possible that those who did not respond are neutral, that is, they had no strong opinion with respect to the subjects in the questionnaire, but this cannot be assumed. If an adverse experience engendered non-response, then the low response rate could conceal an unsuspected level of 'harm' associated with SES.

Of the respondents, the majority had more than one appointment. Some of those with multiple appointments may be individuals in whom hearing impairment was confirmed (true positives), but others will be those in whom no hearing impairment was identified (false positives).

The mean parental anxiety score on finding out about the referral appointment was 5.3 on a 0–10 scale, with 0 indicating 'not at all anxious' and 10 'extremely anxious'. The mean score was 4.7 at the clinic appointment. Severe anxiety, indicated by scores of 10, was registered by only two (3%) parents. Thus, although there was some anxiety associated with referral in those who responded, it appeared moderate, albeit sustained during the waiting period.

Costs were associated with attending the referral appointment in the respondents in terms of time, travel and activities foregone. The median travel cost per appointment was £4.88, travel times to get to the appointment was about half an hour in 60% of appointments, and appointment times were 30–60 minutes in 44% of appointments. Activities foregone were generally work in the case of parents (45% of appointments involved missing work) and school in the case of children (73% of appointments involved missing school).

Despite the inconvenience, virtually all respondents were supportive of SES; 85% of parents strongly agreeing that 'children should have their hearing checked at school'. Supportive statements and overall satisfaction were also evident in the open comments. These identified areas where care needs to be maintained in delivering SES, particularly attention to communication, thereby minimising concerns about the absence of parents at the initial test but also the potential for noise and other distractions in schools to reduce the value of hearing tests.

Strengths and weaknesses

The study was prospectively designed and carried out in accordance with a protocol without deviation. The most important challenge to the validity of the findings was the low response rate, with only 24% of parents to whom questionnaires were sent out providing responses. This was despite a small incentive for taking part and a reminder being offered. This may have been in part because of the length of time between the child being referred and receiving the questionnaire which was sent out after the child reached the end of care. However, for the specific purpose that this component of the clinical studies was designed, we do not believe this is a major shortcoming, but the conclusions drawn should be viewed in the light of a low and potentially biased response rate.

Children referred from SES may truly have a hearing impairment (true positives), may not have a hearing impairment, or have a hearing impairment at the time of screening which later resolves (false positives). We failed to identify information in the literature on what the consequences of false positives might be in a SES programme (see *Chapter 4*). As far as the original 2007 HTA report[12] was concerned, the potential for disutility associated with false positives was not considered, almost certainly because there was no evidence to inform any assumptions. In this respect it would be useful to gauge whether there were likely to be substantial consequences associated with false positives, indicating that they might have a major impact on overall effectiveness and cost-effectiveness. However, as parents were given the option to return the questionnaires anonymously we were not able to collect data on outcomes for all those who

© Queen's Printer and Controller of HMSO 2016. This work was produced by Fortnum *et al.* under the terms of a commissioning contract issued by the Secretary of State for Health. This issue may be freely reproduced for the purposes of private research and study and extracts (or indeed, the full report) may be included in professional journals provided that suitable acknowledgement is made and the reproduction is not associated with any form of advertising. Applications for commercial reproduction should be addressed to: NIHR Journals Library, National Institute for Health Research, Evaluation, Trials and Studies Coordinating Centre, Alpha House, University of Southampton Science Park, Southampton SO16 7NS, UK.

For peer review only - http://bmjopen.bmj.com/site/about/guidelines.xhtml

responded. In *Chapter 5* we report that there were 366 referrals from the SES to the Nottingham service, 60 (16.4%) of whom were confirmed cases. Nearly 90% of confirmed cases had a conductive impairment. We can assume, therefore, that the majority of the questionnaires were returned by the parents of children who did not have a permanent impairment (false positives).

There are clearly some adverse consequences of referral, but these appear to be small, judged externally or by the effect on parental satisfaction. Furthermore, it is reasonable to assume that the effects in non-respondents are unlikely to be greater than those observed in the respondents, so we evaluate that the adverse consequences of referral, particularly for those found not to have hearing impairment, are likely to be no greater on average than those observed in the respondents.

Results in the context of other studies

We emphasise that this is the first attempt to quantify the effect of the referral process in SES on parents and children. Inevitably, there is still much uncertainty, but we would argue that the uncertainty has been reduced. How the results of this study were considered in the economic model of this report is discussed in more detail in *Chapter 8*.

We explored the option to formally quantify the impact on health-related quality of life, particularly for children, through the Health Utilities Index (HUI) instrument but this was not pursued, both because the instrument is not responsive to specific issues related to hearing and on the grounds of cost of using the instrument under licence, a factor amplified by the need to repeat the assessment. Given that the response rate emerged as being problematic in the actual study, it is likely that an attempt to use a more formal instrument like the HUI may have further compromised the response rate.

For peer review only

For peer review only - http://bmjopen.bmj.com/site/about/guidelines.xhtml

DOI: 10.3310/hta20360 HEALTH TECHNOLOGY ASSESSMENT 2016 VOL. 20 NO. 36

Chapter 7 Observations of the practical implementation of screening tests for hearing in schools

Introduction

Aside from the minimal and very weak evidence for the effectiveness of different implementations of SES reported in the 2007 HTA report,[12] we were unable to identify any literature on the practical implications of different implementations or different screen technologies. One paper[17] evaluated the accuracy of the HC navigator device in children with a mean age of 6.8 years in schools in the Philippines and reported low sensitivity when used in the school setting where there is significant ambient noise. The use of the PTS has not been formally evaluated in terms of practicality.

Objectives

- To determine the time resource in implementing either of the two alternative screening methods (PTS and HC screener) in primary schools.
- To elicit the views of the school nurses implementing the screening tests.

Methods

This was a prospective observational cohort study. A researcher from the project team observed school nurses while they conducted hearing screening using two methods: the standard PTS and the HC screener.

The primary end point was the mean cost per child of implementing each of the two test technologies based on time taken to do each test. Secondary end points included a pass or refer for each test, total time of session, school demographics, nurse opinion on ease of use, how much the nurses would want to use that screener in the future, plus other comments.

The school nurse team was approached by a member of the research team and asked for support with the study. School nurses who delivered hearing screening and were happy to be involved drew up a list of schools in which they routinely screened children for hearing impairment. The research team made contact directly with the head teachers of these identified schools, initially by letter, to gain approval for the researcher to access the school to observe the school nurse. Visits were conducted in all three terms of the school year, in order to cover a range of school conditions and the effects of seasonal infections.

Information sheets were distributed to the school via the school nurses (see *Appendix 6*). They were given to parents of children together with information about the school health screen, following the usual process of securing informed consent for the hearing screen. It was explained that, in addition to the routinely used PTS, an extra test (HC) would be carried out by the school nurse and why. Anyone not consenting to either the standard school screen or our extra screen replied to the school nurse, withdrawing their consent via an 'opt-out' system. Only those giving consent to both tests were included in the study, implied by no reply to the contrary.

Tests during SES were observed in primary schools in the Nottingham area. The number of schools was chosen to ensure resources were measured for at least 180 child screens, representing a range of

© Queen's Printer and Controller of HMSO 2016. This work was produced by Fortnum *et al.* under the terms of a commissioning contract issued by the Secretary of State for Health. This issue may be freely reproduced for the purposes of private research and study and extracts (or indeed, the full report) may be included in professional journals provided that suitable acknowledgement is made and the reproduction is not associated with any form of advertising. Applications for commercial reproduction should be addressed to: NIHR Journals Library, National Institute for Health Research, Evaluation, Trials and Studies Coordinating Centre, Alpha House, University of Southampton Science Park, Southampton SO16 7NS, UK.

For peer review only - http://bmjopen.bmj.com/site/about/guidelines.xhtml

catchment populations. Children in the Foundation year or Year 1 (age 4–6 years) were included if they had the usual parental consent for the school screen (following protocols and guidelines for parental consent normally administered by the service) and had not opted out of the SES study.

Data collection took place in the school year from October 2013 to June 2014.

Before the first school session, the researcher met with the nurse to explain and demonstrate how to use the HC screener.

In each school all children > 5 years old in Foundation and Year 1 classes were screened by the school nurse. This meant it was necessary for the school nurse and the observing researcher to attend each school more than once. All children in the appropriate classes who did not opt out were screened using both technologies (routine PTS and HC screener), unless the school nurse chose not to perform both tests (for instance because of a lack of attention or nervousness of the child). The order of the tests was randomised according to computer-generated lists provided by members of the research team in Exeter, but if the school nurse felt that the child might have found it difficult to complete two tests, the PTS was performed first to increase the likelihood of completing the routine screening data.

The HC screening method was used as explained in *Chapter 3*. The nurse indicated 'pass' or 'refer' to the researcher. The researcher recorded this, and the time taken for the screen, on the CRF (see *Appendix 6*) (note: on the CRF a refer outcome is entered as a fail). Nurses did not use HC data to inform their decision on whether a child should be referred for further assessment by audiology services.

The nurse performed the PTS method according to usual practice. The nurse indicated to the researcher whether or not the child had passed the screen. The researcher recorded the time taken for the screen (not including explanation). Data were recorded on the CRF. If the result of the PTS was to refer the child or it was unclear, the child was rescreened on another day and, if necessary, referred onward as appropriate according to usual practice.

The start and end time of the session were recorded on the CRF, along with date, school name and postcode, number of pupils on roll, number of pupils eligible for free school meals (a marker of deprivation), and the name of the nurse and the researcher. Comments were also made about the conditions for the screen (e.g. room, noise levels, disturbances, number of children seen and any difficulties during testing). The researcher informed the nurse which screen was to be conducted first for each child according to the randomisation scheme. The total time of the session included time to take children to and from classrooms, break time, screen explanation time, other screens (e.g. vision tests) and interruptions. The CRF included details of what 'other' times were included in the session.

If, during an assessment session, a particular child became upset, uncomfortable, or uncooperative it was up to the school nurse to decide whether or not they continued the session.

Finally, the school nurses were asked on a scale of 0–10 (0 being low) how they would rate each screening test on ease of use, accuracy and how much they would want to use it in the future.

Each participant was allocated a participant number, but to maintain anonymity and to work with the school nurse's system, no record was kept of which child the number applied to. The original CRFs were kept securely at NHBRU. Photocopies of all CRFs were sent to PenCTU for second data entry and checks.

The schools involved received a short summary of the findings at the end of the study.

Sample size

It was anticipated that a sample size of four schools would provide a convenience sample of about 180 children. The study size was not formally calculated.

For peer review only - http://bmjopen.bmj.com/site/about/guidelines.xhtml

DOI: 10.3310/hta20360 HEALTH TECHNOLOGY ASSESSMENT 2016 VOL. 20 NO. 36

Analysis

The mean and median time taken to complete the screening tests are presented for: (1) all children, (2) children for whom the PTS was administered first and (3) children for whom the HC screen was administered first. The mean time was compared between the PTS and the HC screener using linear regression models. As the distribution of time to complete the test was skewed, bias-corrected accelerated bootstrap CIs were constructed for the mean difference between the PTS and HC tests.

Results

The three school nurses covering Nottingham East (Carlton, Hucknall, Arnold and Calverton) agreed to do the extra screening. In the catchment areas of the three nurses, seven of the 34 schools covered were willing to take part.

Twenty-two observational sessions were conducted in the seven schools in the Nottingham East area through the school year 2013–14. The parents of four children at the sessions attended did not give consent for the study. Of the children for whom consent was given, 191 were observed, 184 of whom completed both tests; three did not complete either test, the other four did not complete the HC screen. Data were analysed only where the test produced a pass or refer classification. For the remaining participants either the test was not done at all or was incomplete.

Children were seen in groups of between two and five, depending on distance to the classroom and occasionally how disruptive the children were. Sometimes, if the classroom was close by, children were allowed to return to class once they had finished. The total session time was recorded, and included time spent collecting children, administration of other tests (vision) and activities (brushing teeth) and occasionally measurement of height and weight, or retests of previous screens.

Of the 188 PTS tests, 40 (21.3%) were referred. Of the 184 HC tests 71 (38.6%) were referred.

Time taken

The mean/median times taken for each screening test were similar for the two tests, at around 1.4 minutes per test, but the range of test times was wider for the PTS (to be expected as the test was not automated). The test time did not appear to vary with the order of the tests. The CI for the mean difference across all screens indicates that the PTS is unlikely to be more than 5 seconds quicker and unlikely to be more than 9 seconds longer on average to administer than the HC screen. That the CI includes zero indicates that it is plausible that there is no difference between the PTS and HC screener in mean time taken (*Table 25*).

TABLE 25 Time taken (minutes) to do screening tests and comparison between the PTS and HC screener

Test	*n*	Mean (SD)	Median (IQR)	Range	Mean difference (PTS – HC)	
Across all occasions						
PTS	188	1.39 (0.67)	1.24 (1.05–1.55)	0.63–7.5	0.002	95% CI –0.08 to 0.14[a]
HC	184	1.39 (0.24)	1.33 (1.27–1.58)	1.03–3.47		
When the PTS is administered first						
PTS	105	1.37 (0.42)	1.28 (1.08–1.57)	0.73–3.22	–0.007	95% CI –0.08 to 0.09[a]
HC	102	1.38 (0.18)	1.33 (1.28–1.45)	1.12–2.45		
When the HC is administered first						
PTS	83	1.42 (0.89)	1.2 (1.03–1.50)	0.63–7.5	0.013	95% CI –0.13 to 0.33[a]
HC	82	1.41 (0.30)	1.33 (1.25–1.45)	1.03–3.47		

a Calculated using the bias-corrected accelerated bootstrap method.

© Queen's Printer and Controller of HMSO 2016. This work was produced by Fortnum *et al.* under the terms of a commissioning contract issued by the Secretary of State for Health. This issue may be freely reproduced for the purposes of private research and study and extracts (or indeed, the full report) may be included in professional journals provided that suitable acknowledgement is made and the reproduction is not associated with any form of advertising. Applications for commercial reproduction should be addressed to: NIHR Journals Library, National Institute for Health Research, Evaluation, Trials and Studies Coordinating Centre, Alpha House, University of Southampton Science Park, Southampton SO16 7NS, UK.

For peer review only - http://bmjopen.bmj.com/site/about/guidelines.xhtml

Observations of researchers and nurses

The researchers observed that schools were often unsuitable for testing hearing because they were too noisy and a suitable alternative room was often not available. On some occasions the nurse had to give up with the hearing tests and return on a different day.

The nurses suggested advantages and disadvantages of each test (*Table 26*)

The three nurses scored each of the tests on a total of 20 occasions for 185 children (no tests at one session because no suitable room available; hearing testing abandoned at another session, after three children tested, owing to noise). All nurses scored all tests as 5 or above for ease of use, accuracy and future use. The mean, median and range of scores are shown in *Table 27*. The PTS test scores higher than the HC test on all measures but in terms of ease of use that might have been because the nurses were

TABLE 26 Advantages and disadvantages of the PTS and the HC screener as observed when used by school nurses

PTS	HC
Advantages	
Provides a full audiogram if needed	Lightweight
Can turn it up in noisy situations	Portable
Can turn it up to check a child's understanding	Hygienic
The headphones help block background noise	No need for mains electrical socket
	No need for headphones
Disadvantages	
Headphones can be tight	Cannot pause to check understanding
Headphones disliked by some children	Cannot pause to wait for background noise to stop
Needs to be plugged into a socket	Cannot vary timing of presentation so some children anticipate when to put up their hand (particularly if they see their peers doing the test before them)
	Cannot repeat a particular tone
	Only plays six tones – and only two frequencies are tested
	The tone at 20 dB is very quiet in a school situation and a lot of children miss it
	Younger children found this more difficult to understand
	No time between tones to give praise or encouragement
	The cups can fall off
	The equipment can get in the way of the child's hand going up

TABLE 27 Ratings of three nurses on the practical implementation of the PTS and HC screener in schools on 20 occasions – scoring from 0 (low) to 10 (maximum)

	PTS			HC		
Attribute	Mean	Median	Range	Mean	Median	Range
Ease of use	8.75	9	7–10	8.40	8	6–10
Accuracy	8.70	9	7–10	6.45	6	6–9
Future use	9.10	10	7–10	6.30	8	5–8

For peer review only - http://bmjopen.bmj.com/site/about/guidelines.xhtml

DOI: 10.3310/hta20360 HEALTH TECHNOLOGY ASSESSMENT 2016 VOL. 20 NO. 36

more familiar with using the PTS. They also rated accuracy more highly for the PTS than the HC screener. When asked how much they would want to use a test in the future they again rated the PTS higher, but commented that they could see the HC screener as being useful as a back-up in some situations.

For two of the nurses the scores all either decreased or stayed the same over time. For the third nurse the HC screener scores stayed the same and the PTS scores increased for ease of use and future use and decreased for accuracy over time.

Discussion

In order to assess the practical issues associated with using the PTS or HC screener as the screening tests in a SES programme, careful observation of screening 184 children in seven schools in the Nottingham SES programme was undertaken. Three nurses covered 22 sessions. Each child had both tests applied, with the test given first being randomly determined.

The average time taken to implement each screening test was nearly identical at about 1.4 minutes, noting that the variability in time was greater for the PTS than for the HC screener. The difference in average time taken to conduct the test was not statistically significant and this was not affected by which test was the first to be used.

Nurses slightly preferred the PTS, but acknowledged that the HC screener could still prove a useful backup for children who refused to wear headphones or when there was no electrical socket available to run the PTS test. The observations about potentially noisy school environments raise the possibility that the accuracy of screening tests may be overestimated in the quieter research environment experienced in diagnostic accuracy studies, including our own.

Strengths and weaknesses

The study was prospective and undertaken in accordance with a protocol without deviation.

The target number of participants was achieved. The observations were carried out in familiar environments for the children allowing assessment of the operation of the screening tests without being compromised by the children feeling anxious – an issue that may have hindered the testing of control children at NHBRU in the diagnostic accuracy study.

Testing took place in a number of different schools throughout the school year, enabling the capture of seasonal changes that might affect hearing through colds, illness or hay fever.

The observations captured data on a range of children from different backgrounds. Several city schools were observed, enabling examination of how the hearing screens by school nurses might be affected by school size, behavioural challenges, support from teaching staff and school facilities, such as test room conditions.

We note the limitation that feedback on the two screening tests and the observations of testing involved only three nurses. However, all nurses undergo the same training and follow a set protocol while screening and therefore procedural differences should not affect test performance. With regard to verbal feedback provided by the nurses on the two tests, all three demonstrated inter-rater reliability, finding similar strengths and weaknesses for both the PTS and HC tests. Gaining opinion from a wider nursing community is unlikely to affect the conclusions drawn.

Results in the context of other studies

As far as we are aware this is the first study to systematically examine the practical issues associated with applying tests that might be used in SES with children in real-life circumstances.

© Queen's Printer and Controller of HMSO 2016. This work was produced by Fortnum *et al.* under the terms of a commissioning contract issued by the Secretary of State for Health. This issue may be freely reproduced for the purposes of private research and study and extracts (or indeed, the full report) may be included in professional journals provided that suitable acknowledgement is made and the reproduction is not associated with any form of advertising. Applications for commercial reproduction should be addressed to: NIHR Journals Library, National Institute for Health Research, Evaluation, Trials and Studies Coordinating Centre, Alpha House, University of Southampton Science Park, Southampton SO16 7NS, UK.

For peer review only - http://bmjopen.bmj.com/site/about/guidelines.xhtml

For peer review only

DOI: 10.3310/hta20360 HEALTH TECHNOLOGY ASSESSMENT 2016 VOL. 20 NO. 36

Chapter 8 Modelling cost-effectiveness of school entry hearing screening

Introduction

Ultimately, the value of a new approach to health care must be judged on the degree to which additional benefits that might arise match the amount of additional resource that would be required to bring about the new approach. This is equally required of SES and in the forerunner to this HTA report,[12] health economic modelling was employed to this end. The health economists on that research team conducted a decision-analytic model after finding that there was little relevant health economic literature and reported it in 2007. The report concluded that SES was potentially cost-effective but subject to considerable uncertainty. The main source of this uncertainty was test accuracy estimates (Professor Linda Davies, University of Manchester, 2011, personal communication). One purpose of this project was thus to improve the estimates of cost-effectiveness by incorporating more precise parameter estimates (particularly test accuracy) into the existing health economic model, accepting that re-examination and redevelopment might be required too, which turned out to be the case as indicated in the methods that follow. The inter-relationship between the studies reported in *Chapters 3–7* and the economic modelling as originally envisaged is shown diagrammatically in *Figure 17*. The other major change from the original health economic model was to be more specific about the methods of screening in the current report. The PTS and HC tests were the two methods evaluated.

Objectives

The overall aim of this chapter is to compare the cost-effectiveness of the PTS and HC tests as methods of SES for hearing impairment and to compare the cost-effectiveness of SES for hearing impairment relative to no screening.

Specific objectives were to:

- update the existing hearing screening model from the 2007 HTA report[12]
- incorporate data from the studies on referrals, costs and diagnostic accuracy (see *Chapters 3–7*)
- estimate the health-related quality of life, costs and utilities of SES compared with no screening and of the PTS versus HC screen, with comparisons based on cost per quality-adjusted life-year (QALY) gained.

Methods

Overall approach

Our approach was designed to estimate the cost-effectiveness of SES primarily from a health-care perspective but to consider other costs where data were available. In this report this was limited to consideration of transport costs associated with families attending diagnostic evaluation; thus the perspective adopted in this report was that of the NHS and the family. The study was designed to capture the costs and benefits of the two different methods of SES in order to inform the policy decision regarding the appropriate use of SES. Since SES is likely to have an impact on the costs and outcomes of all types of hearing impairment, the analysis is concerned with the identification of children with sensorineural or permanent conductive (SNPC) hearing impairment or transitory hearing impairment not diagnosed during the newborn hearing screen or the first 3 years of life (the final pre-school year which is the starting point for the economic model).

© Queen's Printer and Controller of HMSO 2016. This work was produced by Fortnum *et al.* under the terms of a commissioning contract issued by the Secretary of State for Health. This issue may be freely reproduced for the purposes of private research and study and extracts (or indeed, the full report) may be included in professional journals provided that suitable acknowledgement is made and the reproduction is not associated with any form of advertising. Applications for commercial reproduction should be addressed to: NIHR Journals Library, National Institute for Health Research, Evaluation, Trials and Studies Coordinating Centre, Alpha House, University of Southampton Science Park, Southampton SO16 7NS, UK.

For peer review only - http://bmjopen.bmj.com/site/about/guidelines.xhtml

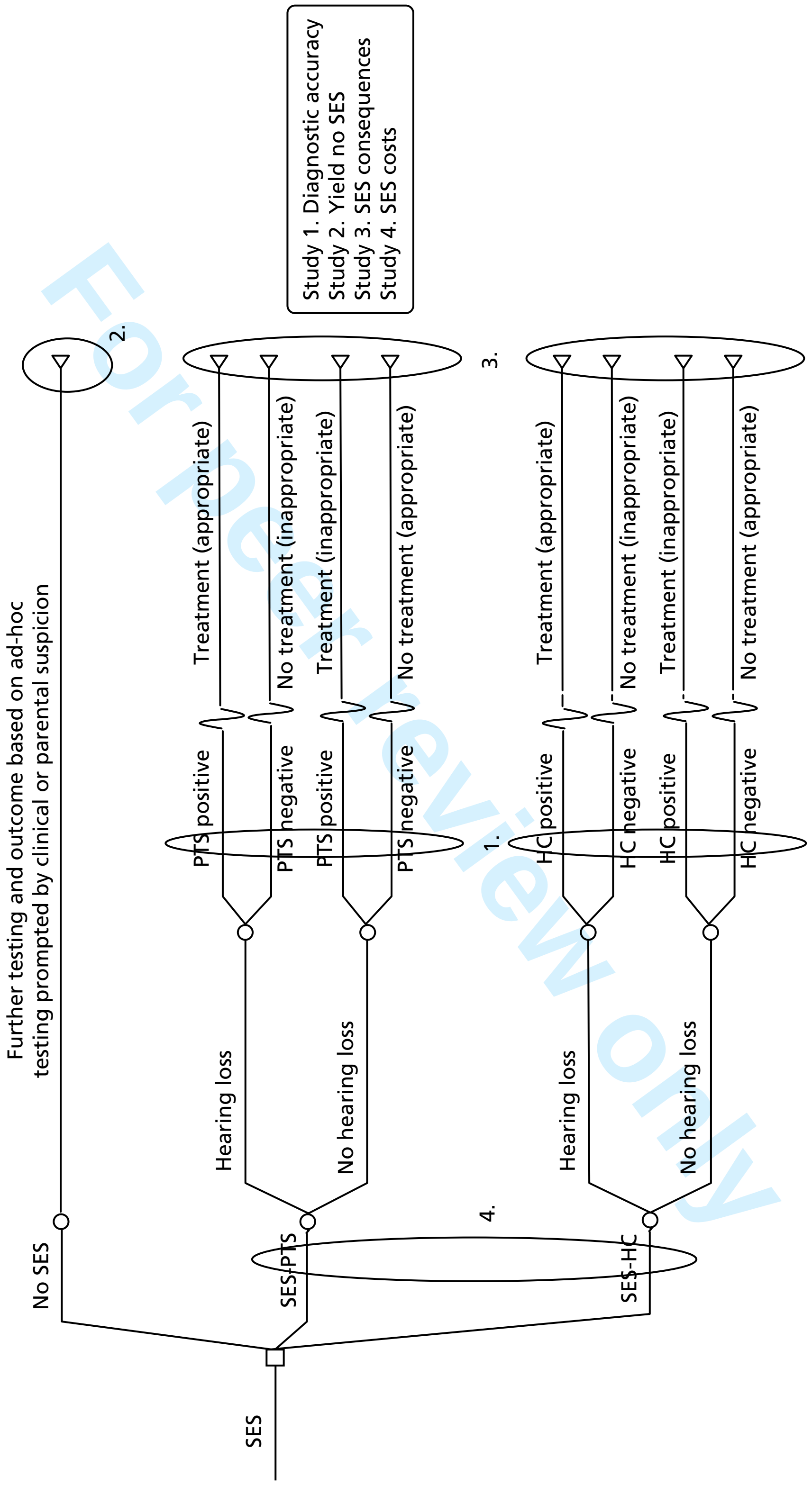

FIGURE 17 Decision tree representation of SES from original bid.

For peer review only

For peer review only - http://bmjopen.bmj.com/site/about/guidelines.xhtml

DOI: 10.3310/hta20360 HEALTH TECHNOLOGY ASSESSMENT 2016 VOL. 20 NO. 36

There are three key considerations that inform whether or not screening is cost-effective relative to no screening. First, there is the issue of whether or not screening at school entry improves the timeliness with which children are referred to diagnostic evaluation and, if indicated, to management, thus generating a more prolonged improvement in quality of life than would otherwise occur. Second, cost-effectiveness will depend on whether or not the diagnostic accuracy of the test makes a difference in the time at which children are identified, referred and managed. If a test has a high level of false negatives, children who should have been picked up by the screening test will experience a delay in their diagnosis and management. Third, to the extent that there are false positives (either from screening or other modes of identification), there is a potential associated negative impact on the child and family of stress and lost time in school or work, as well as additional unnecessary costs to the health system.

The following sections outline the key components of our modelling approach. This includes the rationale and important assumptions required to estimate the cost-effectiveness of the PTS and HC tests and provides some background to the evolution of the modelling approach since the 2007 HTA report.[12]

Decision-analytic structure

The screening question was addressed using a decision-analytical approach to evaluate the cost-effectiveness of school entry hearing screening programmes. The model estimates the costs and consequences of a hypothetical cohort of 10,000 children with a given prevalence of hearing impairment receiving the PTS, HC testing or no screening. The basic structure is presented in *Figure 18*. *Figure 19* shows the route followed from screening to diagnosis of hearing impairment while *Figures 20* and *21* illustrate the management follow-up for transitory and SNPC hearing impairment, respectively. As the decision tree representation does not explicitly capture the time element in the model, this aspect is detailed in the following sections.

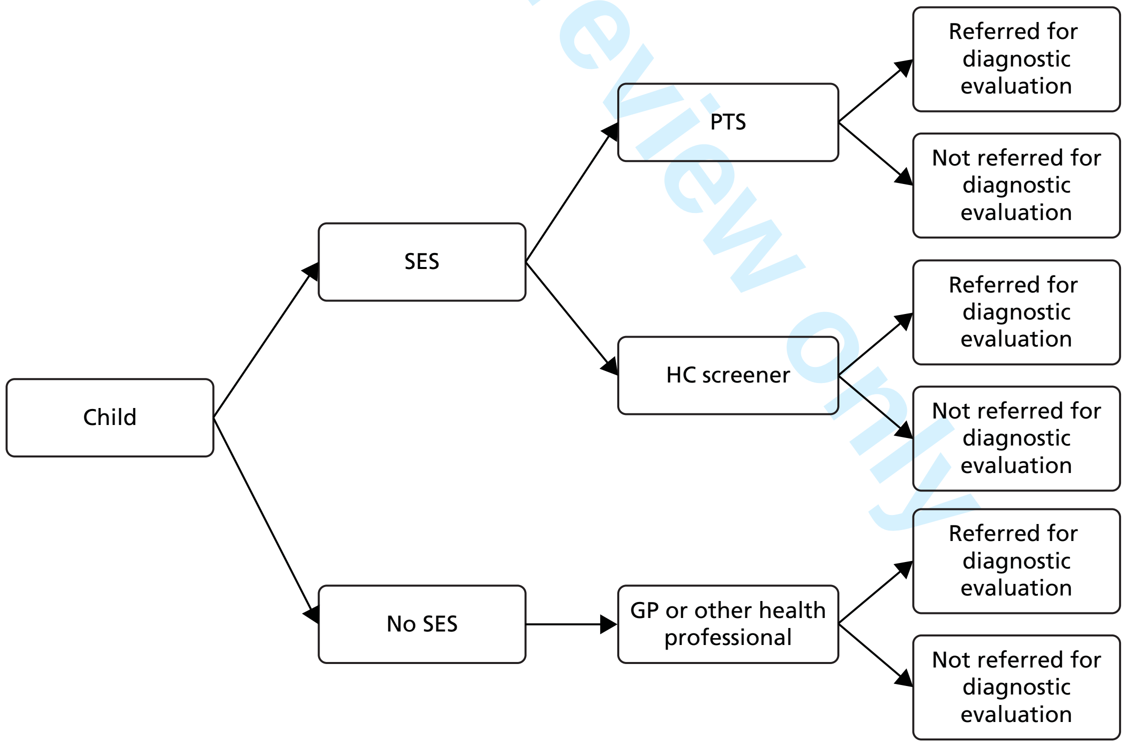

FIGURE 18 Basic decision tree structure.

For peer review only

© Queen's Printer and Controller of HMSO 2016. This work was produced by Fortnum *et al.* under the terms of a commissioning contract issued by the Secretary of State for Health. This issue may be freely reproduced for the purposes of private research and study and extracts (or indeed, the full report) may be included in professional journals provided that suitable acknowledgement is made and the reproduction is not associated with any form of advertising. Applications for commercial reproduction should be addressed to: NIHR Journals Library, National Institute for Health Research, Evaluation, Trials and Studies Coordinating Centre, Alpha House, University of Southampton Science Park, Southampton SO16 7NS, UK.

For peer review only - http://bmjopen.bmj.com/site/about/guidelines.xhtml

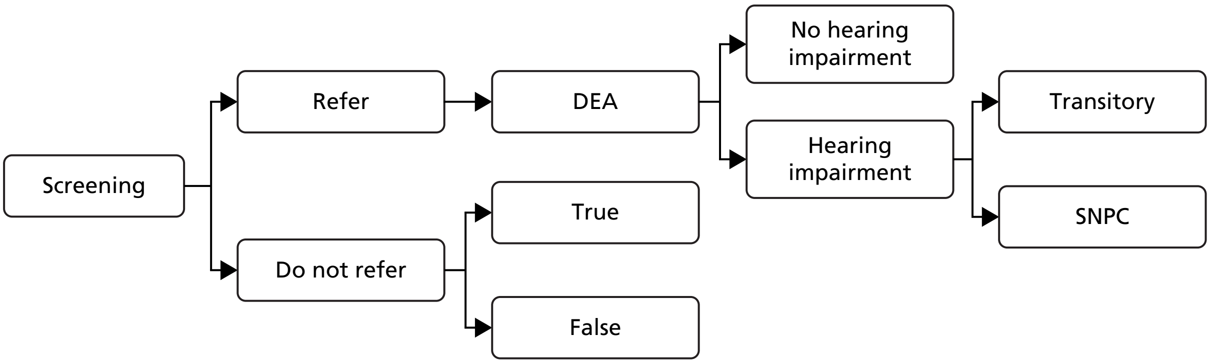

FIGURE 19 Decision tree: screening arm.

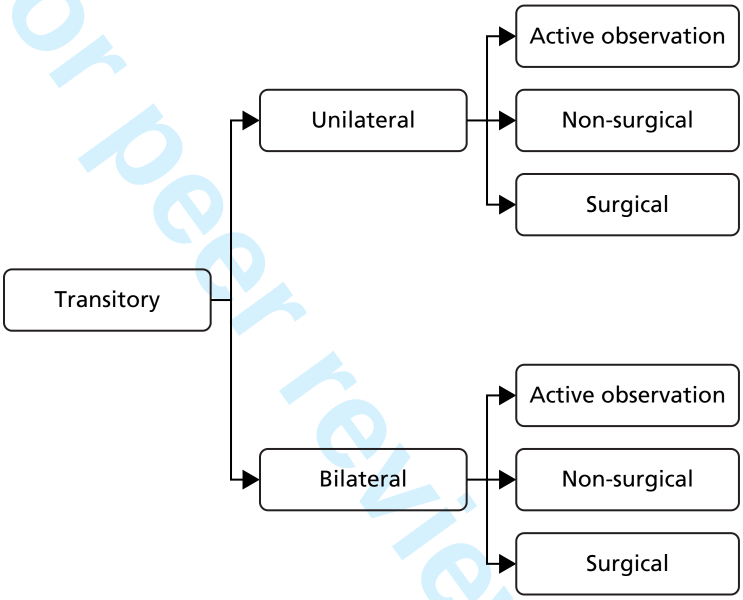

FIGURE 20 Transitory hearing impairment.

For peer review only

For peer review only - http://bmjopen.bmj.com/site/about/guidelines.xhtml

DOI: 10.3310/hta20360 HEALTH TECHNOLOGY ASSESSMENT 2016 VOL. 20 NO. 36

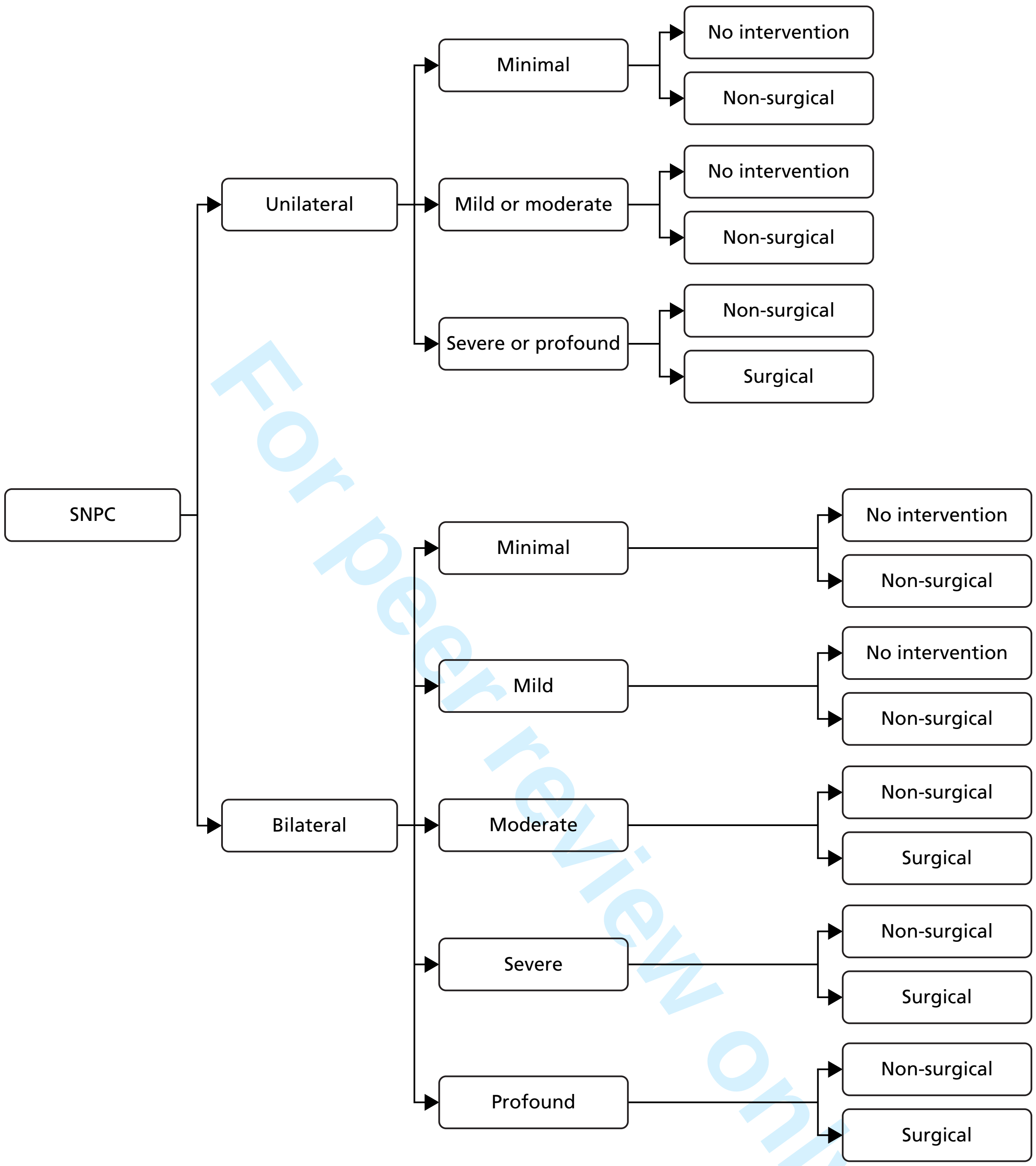

FIGURE 21 Sensorineural or permanent conductive hearing impairment.

For peer review only

Initial model development

Although a version of the original model from the 2007 HTA report[12] [built in TreeAge Pro 2005 (TreeAge Software Inc., Williamstown, MA, USA)] was provided for the current research, the updated model was developed in Microsoft Excel to provide flexibility in the modelling approach. Part of the initial work on this project was to simplify the decision tree model developed for the 2007 HTA report.[12] Based on consultations with the Project Steering Group, a flexible modelling approach was adopted which would retain the logic of the original model and could be represented by a decision tree format, but also capture the way in which hearing impairment is identified over a period of time. This is relevant not only when SES is unavailable and hearing impairment is identified exclusively by other means, but also in the presence of screening, when these other routes of identification may also be important. This chapter describes the

© Queen's Printer and Controller of HMSO 2016. This work was produced by Fortnum *et al.* under the terms of a commissioning contract issued by the Secretary of State for Health. This issue may be freely reproduced for the purposes of private research and study and extracts (or indeed, the full report) may be included in professional journals provided that suitable acknowledgement is made and the reproduction is not associated with any form of advertising. Applications for commercial reproduction should be addressed to: NIHR Journals Library, National Institute for Health Research, Evaluation, Trials and Studies Coordinating Centre, Alpha House, University of Southampton Science Park, Southampton SO16 7NS, UK.

For peer review only - http://bmjopen.bmj.com/site/about/guidelines.xhtml

initial development of the model in anticipation of the results of the clinical studies described elsewhere in this report (see *Chapters 3*, *5–7*) and subsequent to the data becoming available, at which point we were able to generate final estimates of cost-effectiveness for the new technology (HC screener) and the existing technology (PTS) compared with no screening. For the two screening tests, the structure of the evaluation is identical.

Based on information in the 2007 HTA report[12] and correspondence with the lead modeller (Professor Linda Davies), we reconstructed the model used to generate the cost-effectiveness results presented in that report. Before updating the model, as specified in the original protocol, the modelling approach was reviewed to ensure that it was still fit for purpose. The alternative of a more detailed simulation model was considered but it was felt that it would add minimal benefit in terms of answering the research question. It was concluded that a Markov model or discrete-event simulation would not greatly improve the representation of screening while adding to the complexity of the modelling by increasing its data requirements (e.g. the use of transition probabilities). At the same time, this would have presented a considerable challenge in terms of data availability and would have introduced additional parameter uncertainty. As part of the validation of the initial model we developed, we were able to reproduce, within reasonable limits, the original base-case results from the 2007 HTA report.

This initial model was populated with the parameter values used in the original modelling exercise conducted for the 2007 HTA report[12] and was driven by the diagnostic accuracy of the screening tests. For the screening arm of the model, this generated a number of referrals for suspected hearing impairment in the first year (true positives and false positives). As *Figure 19* indicates, children who are referred by the screening test (positive result) are sent for a DEA for a definitive assessment (DEA being assumed to be 100% accurate). It was then assumed that all remaining cases of hearing impairment (false-negative results of the screen) would be identified over the following 2 years (i.e. up to age 7) by referral for a DEA as a result of concern by parents, teachers or other professionals. An assumption was required about the timing of these additional cases. In the absence of other evidence, they were evenly spread over the subsequent 2 years of the model. Similarly, an assumption was required about the rate of identification in the no screening arm. Under no screening, it is assumed that those with a suspected hearing impairment (on the basis of concern by parents, teachers or other professionals) are referred for a DEA. The background rate of identification was set so as to generate an even flow of diagnosed cases over a 3-year period.

Those referred by the screen or referred for a DEA on the basis of the concerns of parents, health professionals, teachers and others (in the presence or absence of screening) will either be found not to have a hearing impairment (false positives) or will be confirmed as having transitory or SNPC hearing impairment in either one or both ears (true positives). Management strategies received by children with hearing impairment include active observation, non-surgical and surgical interventions, with the type of intervention offered varying depending on the severity of hearing impairment.

Model development incorporating new clinical data

Although the basic model structure illustrated in *Figure 18* has remained essentially unchanged in the current version of the model, there have been substantial revisions compared with the model described above to take account of the clinical observations reported in previous chapters. The present model has been informed by the two-gate ('case–control') study investigating the diagnostic accuracy of the two screening methods (see *Chapter 3*), a comparison of a site with a SES programme (Nottingham) and a site without a SES programme (Cambridge) (see *Chapter 5*), a study exploring the impact of referral for diagnostic evaluation on parents and children (see *Chapter 6*) and data on practical implementation in schools (see *Chapter 7*).

For both transitory and SNPC hearing impairment, the model takes account of the benefits of management for those found to have mild, moderate or severe hearing impairment. The analysis runs for a 4-year time period, starting in the year before school entry. By the end of the 4-year time period, evidence on referrals from the studies undertaken in Nottingham and Cambridge indicates that all cases of hearing impairment

For peer review only - http://bmjopen.bmj.com/site/about/guidelines.xhtml

DOI: 10.3310/hta20360 HEALTH TECHNOLOGY ASSESSMENT 2016 VOL. 20 NO. 36

are likely to have been diagnosed regardless of whether children at age 4 years are screened or not screened. In the absence of screening, identification of hearing problems occurs as a result of the concerns of parents, teachers or other professionals, sources which can also generate referrals when a screening programme is in place. As discussed in the following sections, the results of the model are primarily driven by the total numbers of referrals with and without screening, and the numbers referred for a DEA in each year.

Given the key influence of referrals on the results of the analysis, the analysis has explored the effect of varying the number and pattern of referrals over time. This analysis has been reported as a threshold analysis following the presentation of the base-case results. A probabilistic sensitivity analysis (PSA) was not carried out, as it was not considered that probability distributions could usefully be attributed to the numbers of referrals or their timing based on referrals data drawn from two areas. It is argued here that PSA may not always be the most informative technique for exploring parameter uncertainty, as it can result in attention being focused on those variables for which probability distributions are most easily attributed rather than those that have the greatest influence on the results. In this case, while it is recognised that there will be variability in the numbers and rate of referrals with and without hearing screening, this uncertainty is particularly difficult to quantify. Threshold analysis was therefore performed on these variables.

Total referrals and cases of hearing impairment

Table 28 presents the key items of data obtained from the clinical studies that were used in obtaining estimates of the prevalence of hearing impairment, numbers of referrals and results of the screening tests.

The total number of cases of hearing impairment to be identified (with or without screening) is based on the prevalence of hearing impairment using the number of confirmed cases of hearing impairment in Nottingham ($n = 195$) as a proportion of the base population ($N = 42,553$). This gives a prevalence of approximately 46 cases in a population of 10,000. These cases were divided into SNPC and transitory cases of hearing impairment in accordance with the assumption used in the 2007 HTA report.[12] In the base case, the number of referrals has been assumed to be the same in both screening and non-screening arms of the model and is given by the number of children referred in Cambridge ($n = 1108$) as a proportion of the base population ($N = 17,624$), or around 6.3%. This is equivalent to 629 referrals in a population of 10,000. Using the rate of referrals observed in Nottingham does not have a material impact on the cost-effectiveness results. The estimates of test sensitivity are the child-level estimates reported in the diagnostic accuracy study. The implications of these data in terms of screening results (true positive, false positives, true negatives and false negatives) are presented in *Table 29*.

The three groups of most interest are the true positives, false positives and false negatives since no further intervention is required in the true-negative group. True positives and false negatives are generated by the observed diagnostic sensitivity of the two tests, given the prevalence of hearing impairment. Total referrals are made up of true positives, false positives and false negatives, the last of these groups being referred by means other than the screening test so that all cases of hearing impairment are ultimately identified.

TABLE 28 Key data points derived from clinical studies

Parameter	Parameter value	Source
Prevalence of hearing impairment	45.8 per 10,000 children	Confirmed cases in Nottingham as a proportion of base population (see *Chapter 5*)
Referrals for a DEA	628.7 per 10,000 children	Referrals in Cambridge as a proportion of base population (see *Chapter 5*)
Diagnostic sensitivity	PTS: 95.9%; HC: 88.7%	Diagnostic case–control study (see *Table 16*)

© Queen's Printer and Controller of HMSO 2016. This work was produced by Fortnum *et al.* under the terms of a commissioning contract issued by the Secretary of State for Health. This issue may be freely reproduced for the purposes of private research and study and extracts (or indeed, the full report) may be included in professional journals provided that suitable acknowledgement is made and the reproduction is not associated with any form of advertising. Applications for commercial reproduction should be addressed to: NIHR Journals Library, National Institute for Health Research, Evaluation, Trials and Studies Coordinating Centre, Alpha House, University of Southampton Science Park, Southampton SO16 7NS, UK.

For peer review only - http://bmjopen.bmj.com/site/about/guidelines.xhtml

TABLE 29 Screening results: persons per hypothetical cohort of 10,000 children screened

Category	PTS	HC
True positives	43.9	40.6
False positives	582.9	582.9
True negatives	9371.3	9371.3
False negatives	1.9	5.2

Owing to the constraints imposed by the numbers of referrals and prevalence of hearing impairment, the resulting number of false positives implies a substantially higher test specificity (the probability of a child without hearing impairment testing negative) than that found in the new diagnostic accuracy data from Nottingham (*Table 30*). Moreover, these constraints mean that the implied specificity is the same under either screening method in the base case and cannot be varied between the PTS and HC screener.

However, it was possible to explore the impact of varying the numbers of false-positive referrals between the screening and no screening options by varying the total number of referrals. This is a particularly relevant area of uncertainty to consider as, in the base case, total referrals are assumed to be the same with and without screening, whereas evidence from the service comparison study suggests that the rate of referrals may be higher in the absence of screening than when a screening programme is in place.

Although the significantly higher referral rate in Cambridge than Nottingham (34.4 vs. 21.9 per 1000 children per year) does not lead to a significantly higher yield of confirmed cases in Cambridge (3.04 vs. 2.51 per 1000 children per year), the possibility that a screening programme will reduce the number of referrals needs to be considered. Given the differences between the characteristics of the populations in the two areas, it is unclear what the difference in referral rates might be in any given area in the presence or absence of screening. Nevertheless, a higher rate of referrals in the no screening arm could result in a substantial increase in costs relative to the screening option (given the unit cost of diagnostic evaluation relative to screening) and thus have an important influence on the cost-effectiveness of screening. We therefore vary the referral rate between screening and no screening arms of the model in sensitivity analysis.

Distribution of referrals over time

In the current model, the previous assumptions about the identification of hearing impairment over time have been superseded by data on referrals obtained from the screening area (Nottingham) and the no screening area (Cambridge). *Table 31* gives the distribution of referrals by age at last birthday in Cambridge and Nottingham. Referrals at age 3 years are taken to apply to the pre-school entry year (year 1 of the model) while referrals at ages 4–6 years are taken to apply to the first 3 years of school (years 2–4 of the model). In addition to the distribution over time, the model also takes account of the different sources of referral in the presence and absence of screening, based on the available data (*Table 32*). It is worth noting the importance in the screening area of sources of referral other than screening.

TABLE 30 Diagnostic accuracy

Variable	Sensitivity – observed and modelled[a]	Specificity – observed[b]	Specificity – modelled[c]
PTS	95.9%	79.8%	94.1%
HC	88.7%	83.8%	94.1%

a As measured in accuracy study and value used in model.
b As measured in accuracy study.
c Value used in model, implied by the referral rates for a DEA in Cambridge.

For peer review only - http://bmjopen.bmj.com/site/about/guidelines.xhtml

DOI: 10.3310/hta20360 HEALTH TECHNOLOGY ASSESSMENT 2016 VOL. 20 NO. 36

TABLE 31 Percentage of children being referred to diagnostic evaluation by year: base case

Age at last birthday (years)	Model year	Total referrals (non-screening area) (%)	Cumulative	Total referrals (screening area) (%)	Cumulative
3	1	34.4%	34.4%	29.9%	29.9%
4 (school entry year)	2	28.5%	62.9%	29.1%	59.0%
5	3	23.4%	86.3%	28.9%	87.9%
6	4	13.7%	100%	12.1%	100%

TABLE 32 Distribution of referrals by type of referral

Type of referral	No screening (%)	Screening (%)
GP	41.3	10.9
Health visitor	24.3	22.7
School screen	0	21.5
Speech therapist	25.1	8.6
Paediatrician	3.2	8.1
Parent	4.2	12.4
Education	1.9	0.0
School nurse	0	3.1
Community nursery nurse	0	0.8
ENT consultant	0	8.2
Other consultant	0	1.7
Referrals from diagnostic accuracy study	0	2.0

Management of hearing impairment

Once cases of hearing impairment are confirmed by a DEA, their management will depend on the type of hearing impairment and its severity. The available management options are essentially unchanged from the 2007 HTA report.[12] We simply note here that management of hearing impairment results in an improvement in quality of life, which translates into QALYs. While the total QALY difference between screening and no screening will include the QALYs experienced by those with no hearing impairment, we exclude these from our results, as we assume that the characteristics of the modelled populations are the same in respects other than their exposure to type of screening (or no screening). The reported QALYs in screening and no screening groups represent the QALYs accruing only to those with managed hearing impairment who thereby experience an improvement in quality of life. The assumptions underlying the QALY calculations and the other parameters in the model are reported in the following sections.

Sources of parameter inputs

Values of input parameters for the model are based on the original HTA model, the literature, the observational studies described in greater detail elsewhere in this report (see *Chapters 3*, *5–7*) and standard reference sources. A limited literature review was carried out to update health state utilities and costs. For probabilities of different severities of hearing impairment and utilisation of management options, the model used the data on which the original HTA report[12] was based as the steering group felt that these were still relevant.

An overview of sources for the main broad categories of data input is given in *Table 33*.

© Queen's Printer and Controller of HMSO 2016. This work was produced by Fortnum *et al.* under the terms of a commissioning contract issued by the Secretary of State for Health. This issue may be freely reproduced for the purposes of private research and study and extracts (or indeed, the full report) may be included in professional journals provided that suitable acknowledgement is made and the reproduction is not associated with any form of advertising. Applications for commercial reproduction should be addressed to: NIHR Journals Library, National Institute for Health Research, Evaluation, Trials and Studies Coordinating Centre, Alpha House, University of Southampton Science Park, Southampton SO16 7NS, UK.

For peer review only - http://bmjopen.bmj.com/site/about/guidelines.xhtml

TABLE 33 Data sources used for the current model in comparison with the 2007 HTA report[12]

Type of parameter	HTA 2007 parameter source	This HTA parameter source	Updated	Note
Discount rate (costs and QALYs)	UK Treasury green book	UK Treasury green book[57]	No	Deemed to be still valid and follows the NICE Reference Case[58]
Prevalence of deafness	Literature and calculation	New clinical studies	Yes	
Probabilities of types of hearing impairment	Study, literature and calculation	HTA 2007 report and calculation	Yes	
Probabilities of management types	Study, literature and calculation	HTA 2007 report and calculation	Yes	
Utility values	Literature	Literature, HTA 2007 report and new clinical studies	Yes	Updated where new data were available from systematic search
Costs of screening	NHS Reference Costs	Clinical studies and NHS reference costs[59,60]	Yes	
Costs of management	NHS Reference Costs	New clinical studies and NHS reference costs	Yes	
Diagnostic accuracy estimates	Literature	New clinical studies (sensitivity and specificity)	Yes	

NICE, National Institute for Health and Care Excellence.

Reviews of the literature

A short literature review was undertaken to search for any updates to the data required by the model that were not expected to be included in the results of the clinical studies. These were utility values of hearing impairment, the prevalence of hearing impairment (using data from the NHSP) and costs of management.

The scope for searches was publications since 2007, to capture new research published since the 2007 HTA report.[12] Search ranges were from January 2007 to July 2014 and were carried out on 10 July 2014.

The following databases were searched:

- MEDLINE
- EconLit
- SocINDEX
- PsycINFO.

Searches were also run through Google Scholar.

The following charity and support group websites were checked for useful publications (grey literature) that may not show up in searches of conventional databases:

- Action on Hearing Loss
- National Children's Bureau
- National Deaf Children's Society.

Referrals

The clinical studies have, to date, reported on samples of 1108 children referred to audiology services in the no screening area (Cambridge) and 1702 children referred in the screening area (Nottingham).

For peer review only - http://bmjopen.bmj.com/site/about/guidelines.xhtml

For peer review only

DOI: 10.3310/hta20360 HEALTH TECHNOLOGY ASSESSMENT 2016 VOL. 20 NO. 36

Number of children with hearing impairment

Determining the number of children who need to be identified is a critical assumption within the model and the majority of the analysis relies on this calculation. The original HTA report[12] on which this analysis draws assumed that the prevalence of hearing impairment was 78 of 1000 children. This was split into approximately 3.5 of 1000 children with SNPC hearing impairment and 74.5 of 1000 children with transitory hearing impairment through the probabilities that populate the model. The SNPC number is supported by research used, and subsequently published, for the 2007 HTA report,[12] which gives a figure of 3.65 of 1000 children with SNPC hearing impairment.[8,12]

However, although this figure is likely to be an accurate representation of the total prevalence of SNPC hearing impairment in the child population, it is also likely to be a sizeable overestimate of the numbers of children who have SNPC hearing impairment yet to be diagnosed at the age of 3 years (prior to the start of the model). This is because cases of hearing impairment in children are generally identified either through the NHSP or by parents before school age. Moreover, while our main interest is in children with SNPC hearing impairment, the vast majority of the children who are detected as a result of screening will have transitory hearing impairment.

The prevalence of both transitory and SNPC hearing impairment in the modelled population has been estimated on the basis of the numbers with a confirmed diagnosis of hearing impairment as a proportion of the base population in the two areas studied. Based on last appointment, we obtain estimates of 46 per 10,000 children in Nottingham (195/42,553) or 56 per 10,000 children in Cambridge (98/17,624). The former has been used for the purposes of the model and divided between transitory and SNPC hearing impairment in the ratio 96% : 4% as applied in the 2007 HTA report.[12] This gives approximately 2 children per 10,000 with undiagnosed SNPC hearing impairment and 44 with undiagnosed transitory hearing impairment.

Diagnostic accuracy

The sensitivity of the two screening tests used in the model was based on the child-level analysis of the case–control study using all children whether nominally recruited as cases (HI) or controls (NHI), where hearing impairment was defined as a PTA score of ≥ 30 dB on at least one of the four frequencies. Estimates of diagnostic accuracy have been reported (see *Table 30*).

Both screening methods generate a small number of false-negative cases, and, in these instances, the children should be picked up via other referral methods such as through GPs or speech therapists. Differences in sensitivity between the two screening methods will give rise to different numbers of false negatives which have been distributed over time in the model in the manner illustrated in *Table 34*. The distribution draws on the distribution of total referrals but adjusts for false negatives of the screening tests occurring only in year 2 onwards (rather than all 4 years of the model).

TABLE 34 False negatives under the PTS and HC tests

Parameter	PTS value	HC value	Total false negatives (%)
Incorrectly have negative test, will be identified in subsequent years	1.89	5.20	–
Incorrectly pass screening test but are identified in first year	N/A, as screening test run in second year	N/A, as screening test run in second year	–
Incorrectly pass and are identified in second year	0.78	2.16	42%
Incorrectly pass and are identified in third year	0.78	2.14	41%
Incorrectly pass and are identified in fourth year	0.33	0.90	17%

N/A, not applicable.

© Queen's Printer and Controller of HMSO 2016. This work was produced by Fortnum *et al.* under the terms of a commissioning contract issued by the Secretary of State for Health. This issue may be freely reproduced for the purposes of private research and study and extracts (or indeed, the full report) may be included in professional journals provided that suitable acknowledgement is made and the reproduction is not associated with any form of advertising. Applications for commercial reproduction should be addressed to: NIHR Journals Library, National Institute for Health Research, Evaluation, Trials and Studies Coordinating Centre, Alpha House, University of Southampton Science Park, Southampton SO16 7NS, UK.

For peer review only - http://bmjopen.bmj.com/site/about/guidelines.xhtml

Probabilities of hearing impairment by severity

Table 35 reports the proportions of cases of transitory and SNPC hearing impairment that are unilateral or bilateral and the distributions of unilateral and bilateral SNPC hearing impairment by severity. The source of these probabilities is the 2007 HTA report.[12]

Management probabilities

Probabilities of each management type for unilateral and bilateral transitory hearing impairment and the different severities of unilateral and bilateral SNPC hearing impairment were sourced from the 2007 HTA report[12] and are listed in *Table 36*.

Resource use and costs

The following sections report the sources underlying the calculation of costs over the 4-year modelling period. The cost base year is 2012–13. Costs in the first year are undiscounted and, in subsequent years, are discounted at an annual rate of 3.5%. This is the rate recommended by the UK Treasury Green Book,[57] based on the rate at which individuals discount future consumption over present consumption.

Screening costs

All children in the screening arm incur the costs of screening. Children who are referred by the test also incur the cost of a DEA and the subsequent cost of management is incurred by children who are referred by the screening test and are diagnosed with hearing impairment by a DEA.

TABLE 35 Distribution of hearing impairment states

Type of hearing impairment	Probability
Transitory hearing impairment	
Unilateral	0.56
Bilateral	0.44
SNPC hearing impairment is unilateral	
Unilateral	0.60
Bilateral	0.40
Unilateral SNPC	
Minimal severity	0.58
Mild or moderate	0.20
Severe or profound	0.22
Bilateral SNPC	
Minimal severity	0.20
Mild	0.36
Moderate	0.23
Severe	0.10
Profound	0.11

For peer review only - http://bmjopen.bmj.com/site/about/guidelines.xhtml

DOI: 10.3310/hta20360 HEALTH TECHNOLOGY ASSESSMENT 2016 VOL. 20 NO. 36

TABLE 36 Distributions of management types

Type of hearing impairment	Type of management	Probability
Unilateral transitory	Active observation	0.935
	Non-surgical	0.001
	Surgical	0.064
Bilateral transitory	Active observation	0.74
	Non-surgical	0.05
	Surgical	0.21
Unilateral SNPC minimal	No intervention	0.99
	Non-surgical	0.01
Unilateral SNPC mild or moderate	No intervention	0.49
	Non-surgical	0.51
Unilateral SNPC severe or profound	Non-surgical	0.05
	Surgical	0.95
Bilateral SNPC minimal	No intervention	0.96
	Non-surgical	0.04
Bilateral SNPC mild	No intervention	0.7
	Non-surgical	0.3
Bilateral SNPC moderate	Non-surgical	0.7
	Surgical	0.3
Bilateral SNPC severe	Non-surgical	0.9
	Surgical	0.1
Bilateral SNPC profound	Non-surgical	0.5
	Surgical	0.5

The length of time to perform the test was collected (see *Chapter 7*). A mean duration of 1.4 minutes (measured to be the same for both methods) and an hourly rate of £73 per hour from the relevant 2012–13 Reference Costs[60] (N05OGS – School-Based Children's Health Other Services – Group Single Professional) give a staff cost associated with the screening tests of £1.69. This is reported in *Table 37* while unit costs for other sources by which hearing impairment can be identified are presented in *Table 38*. The cost per child of screening was calculated by dividing the total costs (total costs of screening tests and total capital costs) by the total cohort of children screened over 5 years (10,000). The cost of each diagnostic evaluation was £150 (NHS Reference Costs 2012–13[60]).

Cost of travelling to appointments

Sixty respondents to a questionnaire asking about travel costs to attend screening appointments in Nottingham reported a total of 129 appointments and expenditure of £788.64, or £13.14 per child.

Unit costs of the pure-tone screen and HearCheck screener

Unit costs of each method of screening are presented in *Table 37*. Where costs extend beyond 1 year, these have been discounted accordingly in the model. Equipment suppliers have provided the costs of devices and consumables. Although the device costs for the PTS are higher than those for the HC screener (£898.80 vs. £139.20), the total costs for the HC screener are higher, primarily owing to the costs of ear cups. *Table 38* reports the unit costs associated with other means by which hearing impairment is identified.

© Queen's Printer and Controller of HMSO 2016. This work was produced by Fortnum *et al.* under the terms of a commissioning contract issued by the Secretary of State for Health. This issue may be freely reproduced for the purposes of private research and study and extracts (or indeed, the full report) may be included in professional journals provided that suitable acknowledgement is made and the reproduction is not associated with any form of advertising. Applications for commercial reproduction should be addressed to: NIHR Journals Library, National Institute for Health Research, Evaluation, Trials and Studies Coordinating Centre, Alpha House, University of Southampton Science Park, Southampton SO16 7NS, UK.

For peer review only - http://bmjopen.bmj.com/site/about/guidelines.xhtml

TABLE 37 Unit costs of the PTS and HC tests

Parameter	Value	Source	Comment
PTS			
School-Based Children's Health Other Services – Group Single Professional (1 hour)	£73.00	NHS Reference Costs 2012–13[60]	
Average duration of screening test	1.39 minutes	Clinical study	
Screening cost per child	£1.69	Calculation	
Cost of device	£898.80	Clinical study	Equipment suppliers
Printer	£228.00	Clinical study	Equipment suppliers
Battery	£102.00	Clinical study	Equipment suppliers
Calibration (per year)	£170.88	Clinical study	Calibration needed every 12 months
Medicated alcohol-free wipes	£0.032	www.firstaid4less.co.uk/12888_-Infection-Control/Wipes/Multi-Purpose-Wipes/Bioguard-Wipes.html	200 wipes per pack
HC			
School-Based Children's Health Other Services – Group Single Professional (1 hour)	£73.00	NHS Reference Costs 2012–13[60]	
Cost of device	£139.20	Clinical study	Cost calculated with 20% VAT
Cost of ear cup	£0.72	Calculation based on information from clinical study	One to two ear cups used per child. Average of 1.5 used to calculate cost
Calibration cost	£70.01	Calculation based on information from clinical study	Between £50–100 (mid-point used)<br>Calibration needed after 3 years (cost discounted)
Cost of AAA battery	£0.81	www.amazon.co.uk	RRP for pack of eight is £6.49
Outcome pad	£5.60	Clinical study	

RRP, recommended retail price; VAT, value added tax.

For peer review only

For peer review only - http://bmjopen.bmj.com/site/about/guidelines.xhtml

DOI: 10.3310/hta20360 HEALTH TECHNOLOGY ASSESSMENT 2016 VOL. 20 NO. 36

TABLE 38 Unit costs of all non-screening forms of identification

Method of identification	Unit cost	Source
GP	£45.00	Curtis 2014[59]
Health visitor	£20.33	Curtis 2014[59]
Speech therapist	£90.00	Curtis 2014[59]
Paediatrician	£289.00	Curtis 2014[59]
Parent	£13.14	Survey data: 60 children incurred costs of £788.64
Education	£14.10	Calculation (based on average teacher salary of £22,000, working 40 hours a week 39 weeks per year)
School nurse	£27.00	Curtis 2014[59]
Community nursery nurse	£95.00	Curtis 2014[59]
ENT consultant	£289.00	Curtis 2014[59]
Other consultant	£289.00	Curtis 2014[59]

Management costs

Management costs were compiled from NHS Reference Costs for 2012–13,[60] representing national unit costs, and are reported in *Table 39*. Where cost estimates were available for unilateral impairment only, these were multiplied by two to give the corresponding costs associated with bilateral impairment. This is a conservative assumption to account for the costs of providing and maintaining hearing aids for two ears. In the case of surgery, expert opinion suggests that this approach may overestimate costs. No follow-up costs or postoperation observation (active observation) were taken into account with surgical interventions. As with NHS reference costs generally, figures relating to paediatric services or the under 18 years age group were used where available. However, for hearing aids, the reference costs are not broken down for children and adults separately.

Utilities

A search to update the utilities from the 2007 HTA model[12] was undertaken and the results were used to inform the updated parameter sheet (*Table 40*). Where possible, a distinction was made between unilateral and bilateral hearing impairment; otherwise, the utility associated with hearing impairment was determined primarily by its severity. In the first year (the pre-screening year), we assume that children entering the model do so evenly over the course of the year. Total utilities in the first year are therefore reduced by 50% (in undiscounted terms) compared with subsequent years. QALYs have been calculated only for children with a managed hearing impairment who consequently achieve a quality-of-life improvement. As QALYs accruing to children without hearing impairment do not affect the incremental cost-effectiveness ratio (ICER), they have been excluded from the calculations. Limited updating of the parameter values used in the 2007 HTA report was possible. For example, utilities for cochlear implants came from Summerfield *et al.*,[62] and utilities for grommet surgery came from Bisonni *et al.*[63] These studies provided intervention-specific utility data to supplement the evidence on utility by severity of hearing impairment.

Modelling the potential costs and consequences of false-positive results

Chapter 6 presents the outcomes of a questionnaire given to parents whose children were referred from SES for a DEA appointment. Survey responses were obtained in respect of 60 children over 129 appointments.

The questionnaire asked a range of questions concerning parents' views of the screening programme and the impact on themselves and their children. The travel costs associated with screening and diagnostic visits were based on this survey. One impact that is commonly discussed in the context of screening programmes, although not straightforward to value in monetary terms, is the anxiety associated with false-positive results. This was an issue addressed by the questionnaire.

© Queen's Printer and Controller of HMSO 2016. This work was produced by Fortnum *et al.* under the terms of a commissioning contract issued by the Secretary of State for Health. This issue may be freely reproduced for the purposes of private research and study and extracts (or indeed, the full report) may be included in professional journals provided that suitable acknowledgement is made and the reproduction is not associated with any form of advertising. Applications for commercial reproduction should be addressed to: NIHR Journals Library, National Institute for Health Research, Evaluation, Trials and Studies Coordinating Centre, Alpha House, University of Southampton Science Park, Southampton SO16 7NS, UK.

For peer review only - http://bmjopen.bmj.com/site/about/guidelines.xhtml

TABLE 39 NHS management-related reference costs used in the model

Item	Unit cost	Source
Costs of conductive hearing impairment		
Cost of ENT consultant appointment	£95	NHS *Reference Costs 2012–13*[60]
Cost of grommet operation (surgical intervention)	£2325.61	NHS *Reference Costs 2012–13*[60]
Cost of SNPC hearing impairment		
Cost of ENT consultant appointment	£95	NHS *Reference Costs 2012–13*[60]
Cost of hearing aid assessment	£65	NHS *Reference Costs 2012–13*[60]
Cost of hearing aid fit: first visit	£65	NHS *Reference Costs 2012–13*[60]
Cost of hearing aid fit: follow-up visit	£54	NHS *Reference Costs 2012–13*[60]
Cost of hearing aid repair	£26	NHS *Reference Costs 2012–13*[60]
Cost of cochlear implant: unilateral	£20,148.16	NHS *Reference Costs 2012–13*[60]
Cost of cochlear implant: bilateral	£35,653.73	NHS *Reference Costs 2012–13*[60]
Cost of standard hearing aid	£77	NHS *Reference Costs 2012–13*[60]
Cost of digital hearing aid	£85	NHS *Reference Costs 2012–13*[60]
Cost of bone anchored hearing aid: fixture	£3077.35	NHS *Reference Costs 2012–13*[60]
Cost of bone anchored hearing aid: fit	£7997.51	NHS *Reference Costs 2012–13*[60]

Note
NHS Reference Costs do not identify hearing aid-related costs for children separately.

TABLE 40 Utilities used in the model

Health state	Utility	Source
Utility of 1 year with minimal hearing impairment	1.000	Modelling assumption
Utility of 1 year with mild hearing impairment	1.000	Modelling assumption
Utility of 1 year with conductive hearing impairment (unilateral and bilateral)	0.677	Modelling assumption
Utility of 1 year with moderate hearing impairment	0.677	Barton *et al.*, 2004[61]
Utility of 1 year with severe hearing impairment	0.616	Barton *et al.*, 2004[61]
Utility of 1 year with profound hearing impairment	0.353	Barton *et al.*, 2004[61]
Utility of 1 year with mild or moderate hearing impairment	0.8385	Average (mild and moderate)
Utility of 1 year with severe or profound hearing impairment	0.485	Average (severe and profound)
Utility of 1 year with active observation	1.000	Modelling assumption
Utility of 1 year with hearing aid (non-surgical)	1.000	Modelling assumption
Utility of bilateral cochlear implant	0.965	Summerfield *et al.*, 2002[62]
Utility of unilateral cochlear implant	0.934	
Utility of 1 year with grommet surgery	0.995	Bisonni *et al.*, 1991[63]

For peer review only - http://bmjopen.bmj.com/site/about/guidelines.xhtml

DOI: 10.3310/hta20360 HEALTH TECHNOLOGY ASSESSMENT 2016 VOL. 20 NO. 36

Anxiety

Parents were asked to rate their anxiety level on a scale from 0 to 10 as a result of finding out that their child needed further testing. Approximately 60% of respondents listed their anxiety as ≤ 5 out of 10, with only one parent rating their anxiety > 8 out of 10. Between finding out that their child needed further testing and attending the clinic visit, mean parental anxiety score fell slightly, from a score of 5.3 to a score of 4.7. The survey was felt to provide insufficiently compelling evidence to make an adjustment to the model, either by incorporating anxiety into the QALY or as a monetary disbenefit. On the basis of current evidence, it is unknown whether or not there is a health impact on parents of further hearing tests that would outweigh the benefits gained by children receiving a correct diagnosis; this is an issue on which further research may shed some light.

Results

Number of referrals to diagnostic evaluation and numbers diagnosed with hearing impairment

Tables 41 and *42* show the number of children referred for a DEA and the numbers diagnosed with hearing impairment in each model arm over the 4 years of the model. Under the base case, a hypothetical population of 10,000 children has been used, of which the number referred for diagnostic evaluation in the counterfactual and the two intervention arms is 629, and the number with hearing impairment is approximately 46. The numbers referred in each year are determined by the data on referrals at different ages in the screening and no screening sites and have an important bearing on the cost-effectiveness of screening compared with no screening.

TABLE 41 Rate at which children are referred to diagnostic evaluation with audiologist in each model arm: base case

	No screening		PTS		HC	
Year	Referred each year	Cumulative	Referred each year	Cumulative	Referred each year	Cumulative
1	216.3	216.3	188.0	188.0	188.0	188.0
2	179.2	395.5	181.8	369.8	179.9	367.9
3	147.1	542.6	182.5	552.3	183.8	551.7
4	86.1	628.7	76.4	628.7	77.0	628.7

TABLE 42 Rate at which children are diagnosed in each model arm (based on a hypothetical 10,000 population)

	No screening		PTS		HC	
Year	Diagnosed each year	Cumulative	Diagnosed each year	Cumulative	Diagnosed each year	Cumulative
1	15.3	15.3	14.3	14.3	14.3	14.3
2	13.2	28.5	12.5	26.8	10.6	24.9
3	10.6	39.1	12.8	39.6	14.1	39.0
4	6.8	45.9	6.2	45.8	6.8	45.8

© Queen's Printer and Controller of HMSO 2016. This work was produced by Fortnum *et al.* under the terms of a commissioning contract issued by the Secretary of State for Health. This issue may be freely reproduced for the purposes of private research and study and extracts (or indeed, the full report) may be included in professional journals provided that suitable acknowledgement is made and the reproduction is not associated with any form of advertising. Applications for commercial reproduction should be addressed to: NIHR Journals Library, National Institute for Health Research, Evaluation, Trials and Studies Coordinating Centre, Alpha House, University of Southampton Science Park, Southampton SO16 7NS, UK.

For peer review only - http://bmjopen.bmj.com/site/about/guidelines.xhtml

School entry hearing screening versus no screening: costs and quality-adjusted life-years

Tables 43 and *44* present the undiscounted and discounted costs broken down into the costs of identification (whether by screening or other means), diagnostic evaluation and management over 4 years for each arm of the model.

Diagnosis and management of hearing impairment has a positive impact on children's quality of life as measured using the QALY. The figure for QALYs gained outlined in *Table 45* is an estimate of the QALYs gained from children being diagnosed and managed (children moving from hearing impairment to no hearing impairment/managed hearing impairment) over the 4 years of the model. The QALYs accruing to children with no hearing impairment are not included here. The results show that, in the base case, not having a screening programme results in more QALYs than either the PTS or HC screen, which is associated with the lowest QALY gain of the three options.

In order to determine the cost-effectiveness of each method of screening compared with no screening, the ICER needs to be calculated. The ICER presents the ratio of the marginal gain of the intervention over the counterfactual in terms of both costs and benefits. It is calculated as:

$$\text{incremental costs/incremental QALYs.} \quad (1)$$

Table 46 presents incremental costs and QALYs for the two screening approaches relative to no screening and for the PTS compared with the HC. In the base case, it is not appropriate to report an ICER, as no screening dominates (is more effective and less costly than) either screening approach. In the context of our research question, the results indicate that SES is not cost-effective compared with no screening.

TABLE 43 Undiscounted costs

Category	No screening	PTS	HC
Screening and identification	£34,902	£63,786	£69,699
Diagnostic evaluation	£102,564	£102,564	£102,564
Management	£52,194	£52,194	£52,194
Total	£189,660	£218,544	£224,457

TABLE 44 Discounted costs

Category	No screening	PTS	HC
Screening and identification	£33,553	£61,224	£66,929
Diagnostic evaluation	£98,602	£98,360	£98,346
Management	£50,177	£50,054	£50,047
Total	£182,332	£209,638	£215,322

TABLE 45 Quality-adjusted life-years for children with hearing impairment

QALYs	No screening	PTS	HC
Undiscounted	40.93	39.91	39.86
Discounted	35.90	35.21	35.16

For peer review only - http://bmjopen.bmj.com/site/about/guidelines.xhtml

DOI: 10.3310/hta20360 HEALTH TECHNOLOGY ASSESSMENT 2016 VOL. 20 NO. 36

TABLE 46 Incremental costs and QALYs (discounted)

Cost-effectiveness values	PTS vs. no screening	HC vs. no screening	HC vs. PTS
Total costs	£23,171	£28,840	£5,669
QALYs	–0.68	–0.74	–0.06
Incremental cost per QALY ratio	No screening dominates	No screening dominates	PTS dominates

Sensitivity analysis

The key data from the clinical studies that influence the magnitude and direction of the cost-effectiveness results primarily relate to the number and timing of referrals with and without a SES programme. While there is uncertainty about the applicability of the findings from Nottingham and Cambridge to a more general assessment of the costs and benefits of a screening programme as opposed to no screening programme, it is difficult to put boundaries on this uncertainty. This is in part because of the sociodemographic differences between the populations in Cambridge (the counterfactual) and Nottingham (screening site), and to the differences in service configuration between the two sites. A factor of particular significance is the difference in the rates of referrals between the two areas (the question of whether a screening site has more or fewer referrals than a non-screening site was an explicit objective of the study). In the light of clinical data suggesting that the referral rate in Cambridge is higher than that in Nottingham, the impact of a higher referral rate in the absence of screening was felt worthy of exploration. Increasing the referral rate when no screening programme exists increases the costs of this option while leaving QALY benefits unchanged, as these depend on the timing rather than the number of referrals. Increasing referrals sufficiently will render no screening more costly than screening and enable the trade-off between costs and benefits to be investigated.

In order to determine whether an intervention is cost-effective, the National Institutes of Health and Care Excellence (NICE) recommends comparing the ICER with a benchmark value of between £20,000 and £30,000 per QALY gained.[58] Technologies with an ICER of < £20,000 are generally considered to be cost-effective while, for those with an ICER > £20,000, reference needs to be made to other factors when considering value for money (and the case needs to be made increasingly strongly in relation to these factors when the ICER is > £30,000). Using £30,000 per QALY as the cut-off point for cost-effectiveness, the referral rate in the absence of screening would need to increase by ≥ 36% for no screening to cease being cost-effective relative to the PTS. This gives an upper limit on the extent to which referrals can be increased in the absence of screening without this option becoming excessively costly relative to its QALY benefits.

We are also interested in the circumstances under which screening becomes more effective than no screening. Leaving the baseline level of referrals unchanged, we investigated the extent to which referrals would need to be brought forward with screening compared with no screening. As we lacked clear bounds to place around the proportions of children referred at different ages, we again conducted a threshold analysis. It was found that the benefits of the PTS test would be increased sufficiently for the ICER to fall below £30,000 per QALY gained compared with no screening if the proportion of children referred in the first year of school (the screening year) increased by 5.9 percentage points or more.

Compared with the distribution and total numbers of referrals, other variables had relatively little impact on the conclusions of the analysis. For example, raising the sensitivity of the screening tests to 100% increased the QALY benefits under the PTS and HC but not sufficiently to make screening more effective than no screening. Altering the prevalence of hearing impairment had no impact on the results.

© Queen's Printer and Controller of HMSO 2016. This work was produced by Fortnum *et al.* under the terms of a commissioning contract issued by the Secretary of State for Health. This issue may be freely reproduced for the purposes of private research and study and extracts (or indeed, the full report) may be included in professional journals provided that suitable acknowledgement is made and the reproduction is not associated with any form of advertising. Applications for commercial reproduction should be addressed to: NIHR Journals Library, National Institute for Health Research, Evaluation, Trials and Studies Coordinating Centre, Alpha House, University of Southampton Science Park, Southampton SO16 7NS, UK.

For peer review only - http://bmjopen.bmj.com/site/about/guidelines.xhtml

Discussion

Based on a hypothetical population of 10,000 children, it has been calculated that 629 children will be identified and referred for diagnostic evaluation, of which 46 children will be identified as having hearing impairment in the screening and no screening scenarios. The summary of results for this population is as follows:

- The cost of screening ranges between £1.93 (PTS) and £2.49 (HC screener) per child. The total discounted costs associated with the no screening arm are estimated to be £182,333, which consists of the costs of identification, referral, diagnostic evaluation (including travel costs) and management. The screening arm is more costly, with total discounted incremental costs ranging between £27,304 (PTS) and £32,990 (HC screener).
- The discounted QALY gain associated with children being treated for hearing impairment in the no screening arm is 35.9 over 4 years. In the base case, QALYs generated in the absence of screening are greater than those generated in the presence of the PTS (35.21) or HC screen (35.16).
- No screening dominates both screening methods in the base case.

When considering the relative cost-effectiveness of each screening method, the PTS test is less costly (£5686) and is more effective (incremental QALY gain of 0.06) than the HC screen, rendering it dominant over the HC screener.

These conclusions appear to be robust in various sensitivity analyses. The notable exception is where SES is associated with fewer referrals for a DEA, implying a reduction in false-positive cases relative to no screening, which suggest SES could be the cost-effective (if less effective) option. To be more effective than no screening, referrals need to be expedited relative to the base case.

Discussion of key assumptions

Several key modelling assumptions require further discussion. Good use of assumptions is crucial to economic modelling. Done correctly, assumptions simplify the modelling approach, allowing for targeted models to be built using the best quality of data. A model with large parameter demands often has to make compromises and assumptions that can end up weakening the model.

One of the main assumptions in the model concerns the rate of referral to diagnostic evaluation. For the base-case scenario, it has been assumed that the number of referrals in the screening and non-screening areas is the same. From the comparative data presented in *Chapter 5*, the rate of referral in Cambridge (area without SES) was higher than that of Nottingham (area with SES). However, after careful consideration of the issue, these comparative data were not used for the base case, as Cambridge may not be reflective of all non-screening areas. Assuming that the rates of referral would be higher when no screening programme is in place makes an implicit assumption that other methods of referral, such as referral via GP, health visitors and speech therapists have low specificity. That is, it assumes that these other methods require a higher number of referrals than are required with a screening programme to identify the same number of cases of hearing impairment when, in practice, it is not known what the true increase in rate of referrals for non-SES areas relative to SES areas would be.

In the base case, screening is more costly than no screening. However, if referrals are increased in the no screening option, there is a point when no screening becomes more costly than screening. If referrals are increased further, the no screening option will eventually become too costly to justify its additional QALY benefits over screening relative to conventional cost-effectiveness benchmarks such as the £30,000 figure used by NICE. Sensitivity analysis considered the ‘tipping point’ in terms of referral numbers under the no screening option at which no screening would no longer be cost-effective. It was found that the cost per QALY ratio of no screening relative to the PTS test increased to £30,000 if the rate of referrals was 36% higher in the absence of screening compared with the PTS. In this case, screening is the cost-effective (if less effective) option.

For peer review only - http://bmjopen.bmj.com/site/about/guidelines.xhtml

DOI: 10.3310/hta20360 HEALTH TECHNOLOGY ASSESSMENT 2016 VOL. 20 NO. 36

An alternative way in which screening could become cost-effective is if, despite being more costly than no screening (as in the base case), it was also more effective. This could come about if screening was associated with more rapid detection of cases of hearing impairment than no screening. A threshold analysis on the pattern of referrals suggested that, under screening, referrals would need to increase by around 5.9 percentage points in the screening year (increasing the proportion of referrals taking place either in the pre-screening year or the screening year from 59% to 64.9%) in order to reduce the cost per QALY of the PTS relative to no screening to £30,000. While there are grounds for believing that the number of referrals is likely to be higher without screening, the potential for screening to achieve timelier referral and management of HI children is less clear.

Strengths and weaknesses

The model developed has a number of features that support the validity of its results.

It builds on an existing model (2007 HTA report[12]), which allowed a systematic consideration of areas where the original model could be improved and incorporates these into the updated model. This was greatly assisted through the involvement of the architect of the original model who contributed to the project as a consultant (Professor Linda Davies).

The model was conducted by an experienced multidisciplinary team of researchers who had been involved in the development of economic models, and models concerning hearing impairment in particular, prior to this project.

The model also drew on the experience of the project steering group, both in terms of content knowledge to advise on the design of the model and the input of a health economics specialist who fed back on the model design and early results.

The model was underpinned by a protocol outlining the key features and limiting the opportunity for results to be data driven. In the event, changes were made to the structure of the model so that it could capture aspects of the impact of SES that were not originally anticipated. These changes have been fully documented relative to the original model plan, so reducing the opportunity for bias.

The model was conducted in parallel with a series of clinical studies that were designed to improve information on key parameters where high levels of uncertainty had been identified in the 2007 HTA report model (see *Chapters 3*, *5–7*).[12]

There were also some limitations. The greatest limitation was that, despite attempts to reduce uncertainty, the data collection in the accompanying clinical studies was unable to overcome this uncertainty completely. For instance, the study designs for accuracy were chosen on the basis of feasibility and used a diagnostic case–control study, which is known to exaggerate accuracy, particularly where the controls are healthy subjects. Similarly, a randomised comparison between SES and non-SES areas would have been desirable to assess the impact of SES on referrals and yield. Instead we had to employ an observational two-centre comparative study design subject to major potential limitations, including confounding and lack of generalisability. In retrospect we could have invested research in quantifying the accuracy of processes used to refer children for a DEA where SES was not in place, but the importance of this emerged only when the results of the clinical studies were reported.

The inevitable lack of availability of the results of the clinical studies until late in the research programme limited the time available to perform sensitivity analyses in the economic model. These were thus prioritised in consultation with the research and steering group and we remain confident that all key aspects have been covered and that the cost-effectiveness findings remain robust.

© Queen's Printer and Controller of HMSO 2016. This work was produced by Fortnum *et al.* under the terms of a commissioning contract issued by the Secretary of State for Health. This issue may be freely reproduced for the purposes of private research and study and extracts (or indeed, the full report) may be included in professional journals provided that suitable acknowledgement is made and the reproduction is not associated with any form of advertising. Applications for commercial reproduction should be addressed to: NIHR Journals Library, National Institute for Health Research, Evaluation, Trials and Studies Coordinating Centre, Alpha House, University of Southampton Science Park, Southampton SO16 7NS, UK.

For peer review only - http://bmjopen.bmj.com/site/about/guidelines.xhtml

Findings in comparison with other health economic evaluations

The 2007 HTA report[12] identified virtually no health economics literature on SES, and the update search for this report identified no new health economics literature since the 2007 report. Thus the main point of comparison for the new economic model is the economic model from the 2007 HTA report.[12]

The most important difference between the two reports is a change in view about the likely cost-effectiveness of SES from possibly cost-effective (albeit with considerable uncertainty in 2007) to probably not cost-effective in this report. This change is primarily because of the use of observations on the timing of referrals as the basis for the updated model. The 1-year results from the 2007 report, showing a favourable cost-effectiveness ratio for screening, are consistent with a substantial advantage for screening in terms of the timeliness of referrals compared with no screening. This is also implied by the assumptions used in initial attempts to replicate the 2007 results. In comparison, the observational data incorporated into the model on which the findings presented here are based suggest that screening does not result in a more rapid rate of referral and that other methods used in the absence of screening may be more effective in this regard.

For peer review only

For peer review only - http://bmjopen.bmj.com/site/about/guidelines.xhtml

DOI: 10.3310/hta20360 HEALTH TECHNOLOGY ASSESSMENT 2016 VOL. 20 NO. 36

Chapter 9 Conclusions and recommendations

The overarching aims of this project were to evaluate the diagnostic accuracy of hearing screening tests and the cost-effectiveness of screening for hearing impairment at school entry in the UK.

Summary of findings

Systematic review of diagnostic accuracy (see Chapter 2)

The updated review of diagnostic accuracy studies confirms the conclusion from the 2007 HTA report[12] that research to date demonstrates significant variability in the design, methodological quality, and results. Robust conclusions about the performance of individual test types for use in SES cannot be drawn. Summarising the review reported in the 2007 HTA report and this update we conclude that:

- Parental questionnaires had the poorest diagnostic accuracy compared with all other tests.
- The findings from the new audiometry-based studies evaluating computer-based devices and the HC screener reported higher and more consistent specificity but lower and widely varying sensitivity estimates compared with the sweep PTA studies included in the original report.
- Studies evaluating TEOAE reported variable sensitivity with wide CIs, while specificity estimates were relatively high and more consistent.
- The study evaluating AABR reported high sensitivity and specificity.

The review included studies from countries with and without an established UNHS system and with very different systems of health-care delivery. The generalisability of the findings to other situations, including the UK NHS system, is likely to be limited.

Diagnostic accuracy study (see Chapter 3)

The findings of our diagnostic accuracy study indicate that the PTS and HC devices have a high level of sensitivity (PTS $\geq$ 89%, HC $\geq$ 83%) and an acceptably high level of specificity (PTS $\geq$ 78%, HC $\geq$ 83%) for identifying hearing impairment at the level of the ear. The PTS test has greater sensitivity than the HC screener and the HC screener has greater specificity than the PTS.

These conclusions appear robust, with the child-level analyses indicating similar levels of sensitivity and specificity for the screening tests to those seen for the ear-level analyses and for all different definitions of impairment.

Assessment of false negatives (see Chapter 4)

Assessment of false-negative rates is challenging for screening evaluations of a condition that may fluctuate, progress or be of later onset. From our review of the existing literature and data from the diagnostic accuracy study, we are unable to quantify the effect of false-negative results from the PTS or HC screening, but were able to confirm that the rate was extremely low. Of the 16 ears of children in our diagnostic study (total $N = 630$) which passed one or both of the screening tests but were referred by the PTA measure, only four were confirmed to have a hearing impairment at diagnostic evaluation and all were mild (4/630, 0.63%).

Comparison of school entry hearing screening and non-school entry hearing screening services (see Chapter 5)

There was strong evidence that the rate of referral for hearing problems is lower when a SES programme is present. The referral rate was 36% lower in Nottingham (SES) relative to Cambridge (no SES) (rate ratio 0.64, 95% CI 0.59 to 0.69; $p < 0.001$).

© Queen's Printer and Controller of HMSO 2016. This work was produced by Fortnum *et al.* under the terms of a commissioning contract issued by the Secretary of State for Health. This issue may be freely reproduced for the purposes of private research and study and extracts (or indeed, the full report) may be included in professional journals provided that suitable acknowledgement is made and the reproduction is not associated with any form of advertising. Applications for commercial reproduction should be addressed to: NIHR Journals Library, National Institute for Health Research, Evaluation, Trials and Studies Coordinating Centre, Alpha House, University of Southampton Science Park, Southampton SO16 7NS, UK.

For peer review only - http://bmjopen.bmj.com/site/about/guidelines.xhtml

There was little evidence that the yield of confirmed cases differs between areas with and without a SES programme but a higher proportion of referred children were subsequently confirmed to be HI in the area with a SES programme (17.0% in Nottingham vs. 10.6% in Cambridge).

The mean age of referral was nearly identical between the two sites when looking at all referrals but for children who were subsequently confirmed as having a hearing impairment there was strong evidence that children in sites with a screen are older at referral (mean age difference 0.47 years, 95% CI 0.24 to 0.70 years; $p < 0.001$).

It appears that the site with a SES programme is referring fewer children for suspected hearing problems than the site with no SES programme, but there was little evidence that the yield of children with confirmed hearing impairment was different. Children with confirmed hearing impairment are older at referral in the SES site.

Survey of parents (see Chapter 6)

We found from our survey of parents of children referred by the SES programme in Nottingham that the consequences of the referral process for parents and children, including false positives, are minor. They certainly do not appear to be sufficient to undermine parental views about the value of SES. However, it should be noted that the referral process, including the possibility of false positives, occurs both in a system with SES and one relying on ad-hoc referral based on concern alone and hence the data on anxiety and costs could apply to both service implementations. The difference for parents whose child is referred by the SES programme is that they may have had no concerns prior to the screening test.

Practical implementation (see Chapter 7)

We demonstrated minimal differences between the PTS and HC tests in terms of time taken to conduct each examination and practical issues. Testing covered a range of schools throughout the school year and thus we suggest the findings might be generalisable beyond the Nottingham schools.

Cost-effectiveness of school entry hearing screening (see Chapter 8)

Our economic modelling showed that SES is unlikely to be cost-effective and, using base-case assumptions, including the assumption that the number of referrals is the same in the presence or absence of screening, is dominated by a no screening strategy. This is consistent with the observed results of the clinical studies (see *Chapter 5*), which suggest that cases of hearing impairment are identified in similar numbers but at a younger average age in the absence of SES.

Two situations where SES might be cost-effective were identified. In the first situation, SES may be considered cost-effective if there are fewer referrals associated with SES or, conversely, if there are more referrals without screening. This assumption might be supported by the observation from our clinical study (see *Chapter 5*) that referral rates (and by assumption, potential false positives) were less in the site where SES had been in place for many years. However, in order for this to be the case the reduction in referrals would need to be attributable to SES and there is considerable uncertainty about this. The model is also sensitive to a second set of assumptions in which referrals occur more quickly with screening than is observed from our study comparing SES and non-SES sites.

Discussion

The underlying rationale of this research project was to revisit an earlier attempt to assess the clinical effectiveness and cost-effectiveness of SES in the context of the UK NHS, and to seek to improve the quality of evidence on key pieces of information about which there was great uncertainty in the 2007 HTA report.[12] These key uncertainties included (1) the accuracy of the screening tests which might be used; (2) the feasibility of using such tests, particularly in terms of the time required to do them; (3) the consequences to children and parents of being referred for a DEA; and (4) most importantly, the difference

For peer review only - http://bmjopen.bmj.com/site/about/guidelines.xhtml

DOI: 10.3310/hta20360 HEALTH TECHNOLOGY ASSESSMENT 2016 VOL. 20 NO. 36

in number of referrals and yield which might arise where SES is implemented. We have integrated the findings from our clinical studies designed to address these key uncertainties into an updated version of the economic model used in the 2007 HTA report.[12] The overall conclusions for this report should be drawn from our updated overarching cost-effectiveness analysis.

A good example of the need to adopt this more integrated approach to the interpretation of the results is the test accuracy study for the PTS and HC screener (see *Chapter 3*). Although it is clear that the accuracy of both tests, particularly their sensitivity, may look acceptable for the purposes of screening in isolation, it is only when these results are put in the context of our economic model and the screening test considered in the context of the wider health-care system as part of a programme of care, that the question of whether or not the tests are accurate enough can be truly answered. Integrating the various clinical studies of this project, our economic modelling shows that SES is unlikely to be cost-effective and, using base-case assumptions, is dominated by a no screening strategy.

There are, however, some aspects of the individual clinical studies that deserve highlighting in addition. We examined the diagnostic accuracy of two devices, but it might be argued that these were not the optimal devices to assess, and that other devices would perform better, leading to improved effectiveness and cost-effectiveness of SES. The relatively good accuracy achieved by the chosen devices suggests otherwise. Even though there may be devices that achieve greater accuracy, sensitivity analysis on the cost-effectiveness results suggests that even an assumption of 100% sensitivity would not give a cost-effective result. The differences between the tests could be of value in terms of guiding technology for developing improved hearing screening devices. Considering some combination of the two tests might be appropriate, but this was not evaluated here. The updated systematic review does, however, emphasise that technology is constantly evolving with new devices continually emerging and improved evaluations of existing devices being produced.

Two further issues arise when considering diagnostic accuracy. The first relates to considerations of sensitivity and specificity. Sensitivity measures the extent to which the screening test correctly identifies children with a hearing impairment (true positives). Specificity measures the extent to which the screening test correctly identifies children who do not have a hearing impairment (true negatives). Evaluation of the screening tests is influenced by the balance between the two. A high false-negative rate (low sensitivity, i.e. children with a hearing impairment who pass the screening test) is probably a more important problem than a high false-positive rate (low specificity, i.e. children with no hearing impairment referred for a DEA by the screening test). Thus, the desirable balance is for high sensitivity over high specificity.

The second issue concerns the definition of hearing impairment that the screening tests aim to identify and that definition concerns both degree and type. In terms of degree, the 2007 HTA report[12] found that there was considerable variation around the UK in the frequencies used by different services (not all used 0.5, 1, 2, and 4 kHz) and the referral level (20, 30 or 35 dB). The diagnostic accuracy study reported here used the 20 dB level as a referral criterion for the PTS test (as used in several SES programmes) and ≥ 30 dB (PTA) to indicate a hearing impairment.

A more important issue of definition concerns the type of hearing impairment the screening test is designed to identify. Sensorineural hearing impairments are permanent and have implications for the child at whatever level they occur, although the greater the level of impairment, the greater the consequences tend to be. Conductive hearing impairments may be permanent, often associated with anatomical causes, but are usually transient. Such impairments are often caused by OME (glue ear), a very prevalent condition in children. When considering long-term effects for the child, management of permanent impairments is critical but transient impairments can be important in the shorter term, particularly if the glue ear is persistent, and so do warrant management.

© Queen's Printer and Controller of HMSO 2016. This work was produced by Fortnum *et al.* under the terms of a commissioning contract issued by the Secretary of State for Health. This issue may be freely reproduced for the purposes of private research and study and extracts (or indeed, the full report) may be included in professional journals provided that suitable acknowledgement is made and the reproduction is not associated with any form of advertising. Applications for commercial reproduction should be addressed to: NIHR Journals Library, National Institute for Health Research, Evaluation, Trials and Studies Coordinating Centre, Alpha House, University of Southampton Science Park, Southampton SO16 7NS, UK.

For peer review only - http://bmjopen.bmj.com/site/about/guidelines.xhtml

One crucial question is 'What should a screening test of hearing be aiming to identify and hence how should the sensitivity and specificity be assessed?'. Hearing screening tests as they are currently used cannot distinguish between sensorineural and conductive impairments so all children with a hearing impairment will be referred by the screening test. There is an argument that any assessment of cost-effectiveness of a hearing screening programme should consider only permanent impairments and consider the identification of transient losses as an unavoidable by-product. A key point here is that if the programme is not cost-effective when transient impairments are included (and hence more children are identified), a situation based on only identification of permanent impairments is unlikely ever to be cost-effective because identifying and managing transient impairment is no longer counted as a benefit. For the diagnostic accuracy study we have considered a positive result as any impairment, since that is what the screening tests are designed to identify. We have analysed this by ear (to assess the screening tests) and by child (to assess the screening programme).

Ascertaining and capturing the impact of false negatives is a major challenge in any assessment of any screening programme. False negatives arising from hearing impairment, which would be invisible even if the best diagnostic test (PTA) was applied to all children, need to be distinguished from hearing impairment that is overlooked because a screening test is used. Concerning the former, the evidence is that this is likely to be small in amount and minor in nature. In practice false-negative cases are most likely to arise where children are not exposed to the general surveillance of the health care and educational system that might prompt an ad-hoc referral. In contrast, the false negatives arising because a screening test is used instead of a definitive test are measurable and reflected in the screening test's sensitivity. It is again noted that the sensitivity of the PTS and HC screener is good with few false negatives and that where hearing impairment was missed, the nature of the hearing impairment was minor.

The comparison between a site with a SES programme and a site that has not provided a SES programme for a number of years is novel and timely given the number of sites that are actively reviewing all their audiological services. However, any service without a SES programme needs to be very responsive and backed up with information strategies for parents and professionals.

One issue not raised thus far is the availability of the population to be screened. Children who undergo hearing screening at school age could be considered to be a captive audience. Their parents usually have the option to opt out of the screen but the default situation is that children will be screened. In an area with no SES programme, children are referred only if concern is expressed. For this reason in the SES system it could be argued that fewer children with true hearing impairment might be missed (fewer false negatives). For both systems the rate of parents not attending for a DEA with their children is about 15%.

The survey of parents provided measures of anxiety and qualitative data on views of the service as a whole. We did not quantify the costs of that anxiety which might have informed any disutility associated with false positives. False positives would be particularly important if it was thought that screening does lead to a reduction in referrals because, if there was serious disutility, this would amplify the effect of reducing false positives.

This project is unique in that it observed the screening devices being used by regular personnel under usual situations. The data collected on time taken for each test have contributed to the economic model. Perhaps more notable were the anecdotal observations of the researchers and nurses although views from only three nurses using two devices were sought. Confirming the situation reported in the 2007 report,[12] the rooms and facilities offered for screening in many schools are not ideal. Account was not always taken of the requirement for a relatively quiet environment. The presence of children waiting to be screened while another child is screened allows them to become bored and impatient as well as enabling them to see how the system works and what they have to do. This raises the issue of how well accuracy studies that are carried out in rather more optimal conditions with individual children (including our own diagnostic accuracy study), can truly reflect performance in the real-world situation. In assessing the practical implementation of the tests the views of those administering the test are important.

For peer review only - http://bmjopen.bmj.com/site/about/guidelines.xhtml

DOI: 10.3310/hta20360 HEALTH TECHNOLOGY ASSESSMENT 2016 VOL. 20 NO. 36

There are strengths and weaknesses of this programme of research that need to be taken into account in drawing an overall conclusion. The strengths and limitations of the various individual studies of this project have been identified in the context of the contributory chapters of this report. The key overarching strength in this project is that it was planned and conducted in a prospective manner specifically to address the key uncertainties of the 2007 HTA report[12] and thereby provide an improved understanding of the clinical effectiveness and cost-effectiveness of SES, particularly in the context of the UK NHS. The main limitation is that, although uncertainty has been reduced, it has not been eliminated and these continuing areas of uncertainty are highlighted below to inform the suggestions for further research. The most important uncertainty remains the estimates of the number of referrals and yield attributable to SES. The evidence collected here to inform this comes from the observational study comparing an established SES site (Nottingham) with a site where SES has not been employed for many years (Cambridge). While its prospective design limits some of the biases associated with this comparison, major threats to its validity remain, in particular selection bias and confounding. In other words, differences between the areas of Nottingham and Cambridge (other than the provision of SES), such as population characteristics and the nature of the other parts of the health-care system contributing to identification and management of hearing impairment in children, may explain the observed differences in referral rate and yield. While a more experimental design, such as a cluster randomised controlled trial, may overcome these biases and therefore provide a higher level of evidence, this needs to be weighed against the ethical and logistical challenges and costs of such a study.

Overall conclusions and recommendations

In the context of the UK NHS, and similar health-care systems, SES using tests such as the PTS test is unlikely to be effective in increasing the number of cases of hearing impairment identified or lowering the average age at which these cases are identified. SES is also unlikely to be cost-effective when judged against the benchmarks normally used by NICE, relative to a system entirely reliant on ad-hoc referral when a suspicion of hearing impairment is raised.

Implications for practice

Although our finding that the lack of cost-effectiveness of SES may be considered as a reason to withdraw SES where it is currently being practised, we would highlight aspects of the results that suggest caution. First, we have not completely excluded the possibility that SES is cost-effective and we have shown that there are at least two scenarios in which it may be cost-effective. Second, our conclusions are highly dependent on findings in the two specific areas (Nottingham and Cambridge) that were used here, and may not be generalisable to other areas. Third, the cost-effectiveness of SES depends on how effective (or ineffective) the 'no SES system' is. This, in turn, is highly dependent on the effectiveness of ad-hoc identification and referral for a DEA, which is not only largely unknown, but likely to be variable. It seems plausible that SES might have greater potential to be cost-effective where ad-hoc identification and referral is less well developed than in a system where it is well established. If withdrawal of the SES service is to be considered it needs to be carefully managed to ensure that the ad-hoc referral system is working effectively. Health professionals, school and nursery staff, and parents who would then be responsible for referral of children about whom there were concerns in the school entry year might need to be reminded to be more vigilant for signs of hearing impairment.

Implications for research

There are opportunities for further research.

We have highlighted the continuing evolution of evidence on the accuracy of tests for screening for hearing impairment in children of school entry age, and think that it would be useful to do this in an ongoing manner, particularly for the general value of the information to health-care workers testing hearing in childhood. Cochrane now includes reviews of test accuracy, and so would be an ideal vehicle for such an ongoing systematic review. We thus suggest that even though it might not directly impinge on

© Queen's Printer and Controller of HMSO 2016. This work was produced by Fortnum *et al.* under the terms of a commissioning contract issued by the Secretary of State for Health. This issue may be freely reproduced for the purposes of private research and study and extracts (or indeed, the full report) may be included in professional journals provided that suitable acknowledgement is made and the reproduction is not associated with any form of advertising. Applications for commercial reproduction should be addressed to: NIHR Journals Library, National Institute for Health Research, Evaluation, Trials and Studies Coordinating Centre, Alpha House, University of Southampton Science Park, Southampton SO16 7NS, UK.

For peer review only - http://bmjopen.bmj.com/site/about/guidelines.xhtml

a decision about SES, systematic reviews of the accuracy of devices that might be used to measure hearing in children at around school entry age should continue to be pursued, as this performance will undoubtedly influence the performance of any ad-hoc referral system prompted by suspicion of hearing loss.

Our economic model has highlighted the need for careful consideration of the alternatives to SES. It is not appropriate to consider a comparison with SES where there is absolutely no screening/identification activity. Although less well described and potentially subject to variation, the alternative to SES in the UK is a system in which parents, or those involved in the care of children, raise concerns. Where the concerns are substantiated this is followed by referral from a health-care professional, such as a GP, for a DEA. This system will also be taking place where SES is provided, not just in the years before or after school entry, but also in the year of SES. This is an important general finding from this work. Success in identifying hearing impairment in children in the UK is heavily dependent on the effectiveness and cost-effectiveness of the ad-hoc system of referral by a wide range of agents (parents, education, social services and health care) for formal evaluation on suspicion of hearing impairment. This appears to have been very little explored. Thus, in the future, characterising and then measuring the cost-effectiveness of different approaches to the ad-hoc system, with a view to optimising it should receive as much, if not greater priority, than further evaluation of SES.

A related issue is the process by which concern, or referral from SES, is converted into DEAs. It is clear from this project that there is considerable additional triage as the actual number of referrals for a DEA in Nottingham (a service with a SES programme) is much less than the number of referrals that would be expected from the false-positive rates seen in the accuracy estimates (see *Chapter 3*). This may, in part, be because of the fact that, in the real-world implementation of the SES, a child who is referred by a screening test will be re-tested some weeks later before an onward referral for a DEA is made. In this respect, more research on what determines programme specificity (as opposed to test specificity) would be useful.

Understanding why the referral rate in Nottingham was less than in Cambridge (a service without a SES programme) and whether or not this is related to the presence of SES would be of specific interest. Whether or not the difference and cause of any differences is generalisable to other areas would be the wider objective of further research. Further observational studies similar to our comparison between Nottingham and Cambridge could be undertaken, albeit recognising the difficulty of matching the geographical areas.

Further research to better quantify the impact of referral, particularly with respect to anxiety, and whether or not all referrals are affected to the same degree as respondents in our study may be required, particularly if it appears that overall effectiveness and cost-effectiveness could be critically dependent on the costs and disutilities experienced by those testing false positive. We note also that our research has not explored children's perspectives on testing for hearing impairment and so this could be an important target for further research.

Expanding the research on implementation to more sites could contribute further information on differences in provision and further opinion from nurses applying the tests within those different systems.

If withdrawal of SES is contemplated in particular settings, this could be used as an opportunity for further data collection. Particularly where the pattern of referrals and cases was known over many years in the run up to withdrawal, any change in pattern of referrals/cases could be very useful evidence confirming the lack of effectiveness and cost-effectiveness of SES, or challenging it. More formally if SES cessation is being contemplated in many areas, a randomised trial of withdrawal of SES programmes could be designed using referrals and hearing impairment cases identified as outcomes.

For peer review only - http://bmjopen.bmj.com/site/about/guidelines.xhtml

DOI: 10.3310/hta20360 HEALTH TECHNOLOGY ASSESSMENT 2016 VOL. 20 NO. 36

Acknowledgements

We would like to thank the following, without whom this project could not have taken place.

The participating families and schools.

The steering committee for their contributions to the project and in particular the chairperson, Professor John Bamford, for his advice on the final report.

Linda Davies (University of Manchester) and Jon Deeks (University of Birmingham) who provided comments on the cost-effectiveness and test accuracy components of the study respectively and received a small consultancy payment for this feedback.

The school nurses who carried out the extra screening for us: Natalie Campbell, Rachel Hodgkinson and Shelley Whiteley, and Mary Barks and Ann Allardice for helping to co-ordinate the practical implementation study.

Elliot Carter, Brendan Lane and Paul Williams from PenCTU who developed the data entry websites and databases. Brian Wainman and Mark Warner from PenCTU for entering data and assisting with the data management tasks.

Former project team member Vasilis Nikolaou.

Ketevan Rtveladze, and Tracey Jhita (Optimity Matrix) for developing the model.

Nicola Huxley and Tristan Snowshill (PenTAG, University of Exeter Medical School) for checking the model.

Venessa Vas for helping to complete the screening in the diagnostic accuracy study when Sam Errington left the team.

The audiologists who extracted and entered data, and sent out questionnaires for the survey: Karenbir Basi, Jeff Davis, Gemma Hopkins and Emily Balmer.

The audiologists at each centre for sending out invitations for the diagnostic accuracy study.

Simon Briscoe (Exeter) for reviewing the search strategy.

Heather Fortnum is funded by the University of Nottingham and supported by the NIHR NHBRU.

Mara Ozolins and Sam Errington are supported by a grant from the NIHR Health HTA programme (10/63/03).

Obioha C Ukoumunne, Rod S Taylor and Zhivko Zhelev are supported by the NIHR Collaboration for Leadership in Applied Health Research and Care South West Peninsula at the Royal Devon and Exeter NHS Foundation Trust.

Chris Hyde is supported by the University of Exeter.

Claire Benton is funded by Nottingham University Hospitals NHS Trust.

Joanne Moody is funded by CCS NHS Trust and supported by Addenbrookes Hospital, Cambridge University Hospitals Foundation Trust.

© Queen's Printer and Controller of HMSO 2016. This work was produced by Fortnum *et al.* under the terms of a commissioning contract issued by the Secretary of State for Health. This issue may be freely reproduced for the purposes of private research and study and extracts (or indeed, the full report) may be included in professional journals provided that suitable acknowledgement is made and the reproduction is not associated with any form of advertising. Applications for commercial reproduction should be addressed to: NIHR Journals Library, National Institute for Health Research, Evaluation, Trials and Studies Coordinating Centre, Alpha House, University of Southampton Science Park, Southampton SO16 7NS, UK.

For peer review only - http://bmjopen.bmj.com/site/about/guidelines.xhtml

Membership of the steering committee

John Bamford (Chairperson and Honorary Professor of Audiology) University of Manchester.

John Fitzgerald (Consultant Clinical Scientist and Head of Audiological Services) Norfolk and Norwich University Hospital.

Vicki Kirwin (Audiology Specialist) The National Deaf Children's Society.

Kevin Munro (Professor of Audiology) University of Manchester and Honorary Consultant Clinical Scientist, Central Manchester University Hospitals NHS Foundation Trust.

Kate Northstone (Senior Research Fellow) School of Social and Community Medicine, University of Bristol (to March 2013).

Karen Smith (Principal Statistician) Department of Health Sciences, University of Leicester (from April 2013).

Eldon Spackman (Research Fellow) Centre for Health Economics, University of York.

Observers

Heather Fortnum (Chief Investigator), Associate Professor and Reader, NIHR NHBRU, University of Nottingham.

Maria Koufali (deputised by Stacey Arland), Deputy Director, Research and Innovation, Nottingham University Hospitals.

Angela Shone (Sponsor), Head of Research Governance, Research and Graduate Services, University of Nottingham.

Obi Ukoumunne (Project Statistician), Associate Professor in Medical Statistics, University of Exeter.

Contributions of authors

Dr Heather Fortnum (Associate Professor and Reader, hearing research) conceived the study, was principal investigator for the study, wrote the original protocol, contributed to revisions of design and conduct of the study, oversaw data collection and analyses, was responsible for the first draft of *Chapters 1*, *4*, *6*, *7* and *9* and contributed to the whole manuscript.

Dr Obioha C Ukoumunne (Associate Professor, statistics) was a co-applicant, contributed to revisions of design and conduct of the study, is the project statistician, analysed all data for the project, was responsible for *Chapters 3* and *5* and contributed to the whole manuscript.

Professor Chris Hyde (Professor, public health and epidemiology) was a co-applicant, contributed to revisions of design and conduct of the study, developed and supervised the analyses of the economic model, was responsible for *Chapter 8* and contributed to the whole manuscript. He took over the role of corresponding author on the retirement of the principal investigator, Heather Fortnum, in 2015.

Professor Rod S Taylor (Professor, health services research) was a co-applicant, contributed to revisions of design and conduct of the study, and contributed to this manuscript.

For peer review only - http://bmjopen.bmj.com/site/about/guidelines.xhtml

For peer review only

DOI: 10.3310/hta20360 HEALTH TECHNOLOGY ASSESSMENT 2016 VOL. 20 NO. 36

Ms Mara Ozolins (Research Fellow, hearing research) contributed to revisions of design and conduct of the study, wrote study documents and meeting minutes, collected data for the diagnostic accuracy and practical implementation studies, monitored the budget, produced progress reports for the HTA and contributed to the whole manuscript.

Ms Sam Errington (Research Associate, hearing research) contributed to revisions of design and conduct of the study, wrote study documents and meeting minutes, collected data for the diagnostic accuracy and practical implementation studies, obtained ethical approvals, ran search strategies for systematic reviews and contributed to this manuscript.

Dr Zhivko Zhelev (Research Fellow, systematic reviews) conducted and wrote up the systematic review (see *Chapter 2*).

Mr Clive Pritchard (Principal Economist) managed the changes to the economic model and contributed to *Chapter 8*.

Ms Claire Benton [Clinical Scientist (audiology), paediatric audiology] was a co-applicant, contributed to revisions of design and conduct of the study, invited case children for the diagnostic accuracy study, collected data for the comparison study and contributed to this manuscript.

Ms Joanne Moody [Clinical Scientist (audiology), paediatric audiology] was a co-applicant, contributed to revisions of design and conduct of the study, collected data for the comparison study and contributed to this manuscript.

Mrs Laura Cocking (PenCTU Senior Data Manager) advised on and co-ordinated database design and development, data validation and data export, and contributed to this manuscript.

Mr Julian Watson (Parent Representative) contributed to design and conduct of the study, and contributed to this manuscript.

Ms Sarah Roberts (Economist) made the changes to the economic model and contributed to *Chapter 8*.

Data sharing statement

Data are available via the corresponding author.

For peer review only

© Queen's Printer and Controller of HMSO 2016. This work was produced by Fortnum *et al.* under the terms of a commissioning contract issued by the Secretary of State for Health. This issue may be freely reproduced for the purposes of private research and study and extracts (or indeed, the full report) may be included in professional journals provided that suitable acknowledgement is made and the reproduction is not associated with any form of advertising. Applications for commercial reproduction should be addressed to: NIHR Journals Library, National Institute for Health Research, Evaluation, Trials and Studies Coordinating Centre, Alpha House, University of Southampton Science Park, Southampton SO16 7NS, UK.

For peer review only - http://bmjopen.bmj.com/site/about/guidelines.xhtml

For peer review only

DOI: 10.3310/hta20360 HEALTH TECHNOLOGY ASSESSMENT 2016 VOL. 20 NO. 36

References

1. Yoshinaga-Itano C, Coulter D, Thomson V. The Colorado Newborn Hearing Screening Project: effects on speech and language development for children with hearing loss. *J Perinatol* 2000;**20**:S132–7. http://dx.doi.org/10.1038/sj.jp.7200438
2. Davis A, Bamford J, Wilson I, Ramkalawan T, Forshaw M, Wright S. A critical review of the role of neonatal hearing screening in the detection of congenital hearing impairment. *Health Technol Assess* 1997;**1**:(10).
3. Kennedy C, McCann D, Campbell MJ, Kimm L, Thornton R. Universal newborn screening for permanent childhood hearing impairment: an 8-year follow-up of a controlled trial. *Lancet* 2005;**366**:660–2. http://dx.doi.org/10.1016/S0140-6736(05)67138-3
4. van Dommelen P, van Straaten HL, Verkerk PH. Ten-year quality assurance of the nationwide hearing screening programme in Dutch neonatal intensive care units. *Acta Paediatr* 2011;**100**:1097–103. http://dx.doi.org/10.1111/j.1651-2227.2011.02230.x
5. Fortnum HM, Summerfield AQ, Marshall DH, Davis AC, Bamford JM. Prevalence of permanent childhood hearing impairment in the United Kingdom and implications for universal neonatal hearing screening: questionnaire based ascertainment study. *BMJ* 2001;**323**:536–40. http://dx.doi.org/10.1136/bmj.323.7312.536
6. Bamford J, Uus K, Davis A. Screening for hearing loss in childhood: issues, evidence and current approaches in the UK. *J Med Screen* 2005;**12**:119–24. http://dx.doi.org/10.1258/0969141054855256
7. Russ SA, Poulakis Z, Wake M, Barker M, Rickards F, Jarman FC, *et al.* The distraction test: the last word? *J Paediatr Child Health* 2005;**41**:197–200. http://dx.doi.org/10.1111/j.1440-1754.2005.00587.x
8. Watkin PM, Baldwin M. Identifying deafness in early childhood: requirements after the newborn hearing screen. *Arch Dis Child* 2011;**96**:62–6. http://dx.doi.org/10.1136/adc.2010.185819
9. Stewart-Brown S, Haslum MN. Screening for hearing loss in childhood: a study of national practice. *Br Med J (Clin Res Ed)* 1987;**294**:1386–8. http://dx.doi.org/10.1136/bmj.294.6584.1386
10. Haggard M. *Research and Development of Effective Services for Hearing-Impaired People*. London: Nuffield Provincial Hospitals Trust; 1993.
11. Fonseca S, Forsyth H, Neary W. School hearing screening programme in the UK: practice and performance. *Arch Dis Child* 2005;**90**:154–6. http://dx.doi.org/10.1136/adc.2003.046979
12. Bamford J, Fortnum H, Bristow K, Smith J, Vamvakas G, Davies L, *et al.* Current practice, accuracy, effectiveness and cost-effectiveness of the school entry hearing screen. *Health Technol Assess* 2007;**11**(32). http://dx.doi.org/10.3310/hta11320
13. Williamson I. Otitis media with effusion in children. *BMJ Clin Evid* 2011;**2011**:502.
14. Midgley EJ, Dewey C, Pryce K, Maw AR. The frequency of otitis media with effusion in British pre-school children: a guide for treatment. ALSPAC Study Team. *Clin Otolaryngol Allied Sci* 2000;**25**:485–91. http://dx.doi.org/10.1046/j.1365-2273.2000.00360.x
15. National Collaborating Centre for Women's and Children's Health (UK). *Surgical Management of Otitis Media with Effusion in Children*. London: RCOG Press; 2008.
16. Feldman W, Milner RA, Sackett B, Gilbert S. Effects of preschool screening for vision and hearing on prevalence of vision and hearing problems 6–12 months later. *Lancet* 1980;**2**:1014–16. http://dx.doi.org/10.1016/S0140-6736(80)92167-4

© Queen's Printer and Controller of HMSO 2016. This work was produced by Fortnum *et al.* under the terms of a commissioning contract issued by the Secretary of State for Health. This issue may be freely reproduced for the purposes of private research and study and extracts (or indeed, the full report) may be included in professional journals provided that suitable acknowledgement is made and the reproduction is not associated with any form of advertising. Applications for commercial reproduction should be addressed to: NIHR Journals Library, National Institute for Health Research, Evaluation, Trials and Studies Coordinating Centre, Alpha House, University of Southampton Science Park, Southampton SO16 7NS, UK.

For peer review only - http://bmjopen.bmj.com/site/about/guidelines.xhtml

REFERENCES

17. Gloria-Cruz TLI, Carrillo JRC, Chan AL, Chiong CM, Llanes EGDV, Plete JR, *et al.* The accuracy of the hearing screener device as a hearing screening tool in the school setting for first graders aged 5–10 years. *Otolaryngology* 2013;**134**. http://dx.doi.org/10.4172/2161-119x.1000134

18. Whiting P, Rutjes A, Reitsma J, Bossuyt P, Kleijnen J. The development of QUADAS: a tool for the quality assessment of studies of diagnostic accuracy included in systematic reviews. *BMC Med Res Methodol* 2003;**3**:25. http://dx.doi.org/10.1186/1471-2288-3-25

19. Whiting PF, Rutjes AW, Westwood ME, Mallett S, Deeks JJ, Reitsma JB, *et al.* QUADAS-2: a revised tool for the quality assessment of diagnostic accuracy studies. *Ann Intern Med* 2011;**155**:529–36. http://dx.doi.org/10.7326/0003-4819-155-8-201110180-00009

20. Deeks JJ, Macaskill P, Irwig L. The performance of tests of publication bias and other sample size effects in systematic reviews of diagnostic test accuracy was assessed. *J Clin Epidemiol* 2005;**58**:882–93. http://dx.doi.org/10.1016/j.jclinepi.2005.01.016

21. El-Naggar M, Hashlamoun M. Paediatric hearing assessment and screening clinic at Fujairah: analysis of the results of the first 6 months of clinic practice. *Emirates Med J* 2005;**23**:15–20.

22. Bu X, Li X, Driscoll C. The Chinese hearing questionnaire for school children. *J Am Acad Audiol* 2005;**16**:687–97. http://dx.doi.org/10.3766/jaaa.16.9.6

23. Li X, Driscoll C, Culbert N. Investigating the performance of a questionnaire for hearing screening of schoolchildren in China. *Aust NZ J Audiol* 2009;**31**:45–52. http://dx.doi.org/10.1375/audi.31.1.45

24. Newton VE, Macharia I, Mugwe P, Ototo B, Kan SW. Evaluation of the use of a questionnaire to detect hearing loss in Kenyan pre-school children. *Int J Pediatr Otorhinolaryngol* 2001;**57**:229–34. http://dx.doi.org/10.1016/S0165-5876(00)00453-5

25. Samelli AG, Rabelo CM, Vespasiano AP. Development and analysis of a low-cost screening tool to identify and classify hearing loss in children: a proposal for developing countries. *Clinics (Sao Paulo)* 2011;**66**:1943–8.

26. Soares M, Nakazawa M, Ishikawa K, Sato T, Honda K. Hearing screening for Japanese children and young adults using the automated auditory brainstem response. *Auris Nasus Larynx* 2014;**41**:17–21. http://dx.doi.org/10.1016/j.anl.2013.08.001

27. Wu W, Lu J, Li Y, Kam AC, Fai Tong MC, Huang Z, *et al.* A new hearing screening system for preschool children. *Int J Pediatr Otorhinolaryngol* 2014;**78**:290–5. http://dx.doi.org/10.1016/j.ijporl.2013.11.026

28. Yin L, Bottrell C, Clarke N, Shacks J, Poulsen MK. Otoacoustic emissions: a valid, efficient first-line hearing screen for preschool children. *J Sch Health* 2009;**79**:147–52. http://dx.doi.org/10.1111/j.1746-1561.2009.00383.x

29. Georgalas C, Xenellis J, Davilis D, Tzangaroulakis A, Ferekidis E. Screening for hearing loss and middle-ear effusion in school-age children, using transient evoked otoacoustic emissions: a feasibility study. *J Laryngol Otol* 2008;**122**:1299–304. http://dx.doi.org/10.1017/S0022215108002156

30. McPherson B, Law MMS, Wong MSM. Hearing screening for school children: comparison of low-cost, computer-based and conventional audiometry. *Child Care Health Dev* 2010;**36**:323–31. http://dx.doi.org/10.1111/j.1365-2214.2010.01079.x

31. Leeflang MM, Moons KG, Reitsma JB, Zwinderman AH. Bias in sensitivity and specificity caused by data-driven selection of optimal cutoff values: mechanisms, magnitude, and solutions. *Clin Chem* 2008;**54**:729–37. http://dx.doi.org/10.1373/clinchem.2007.096032

For peer review only

For peer review only - http://bmjopen.bmj.com/site/about/guidelines.xhtml

32. Rutjes AW, Reitsma JB, Vandenbroucke JP, Glas AS, Bossuyt PM. Case–control and two-gate designs in diagnostic accuracy studies. *Clin Chem* 2005;**51**:1335–41. http://dx.doi.org/10.1373/clinchem.2005.048595

33. Gomes M, Lichtig I. Evaluation of the use of a questionnaire by non-specialists to detect hearing loss in preschool Brazilian children. *Int J Rehabil Res* 2005;**28**:171–4. http://dx.doi.org/10.1097/00004356-200506000-00012

34. Olusanya B. Early detection of hearing impairment in a developing country: what options? *Audiology* 2001;**40**:141–7. http://dx.doi.org/10.3109/00206090109073109

35. Hammond PD, Gold MS, Wigg NR, Volkmer RE. Preschool hearing screening: evaluation of a parental questionnaire. *J Paediatr Child Health* 1997;**33**:528–30. http://dx.doi.org/10.1111/j.1440-1754.1997.tb01664.x

36. Sabo MP, Winston R, Macias JD. Comparison of pure tone and transient otoacoustic emissions screening in a grade school population. *Am J Otol* 2000;**21**:88–91. http://dx.doi.org/10.1016/S0196-0709(00)80080-0

37. Orlando MS, Frank T. Audiometer and AudioScope hearing screening compared with threshold test in young children. *J Pediatr* 1987;**110**:261–3. http://dx.doi.org/10.1016/S0022-3476(87)80168-3

38. FitzZaland RE, Zink GD. A comparative study of hearing screening procedures. *Ear Hear* 1984;**5**:205–10. http://dx.doi.org/10.1097/00003446-198407000-00005

39. Holtby I, Forster DP, Kumar U. Pure tone audiometry and impedance screening of school entrant children by nurses: evaluation in a practical setting. *J Epidemiol Community Health* 1997;**51**:711–15. http://dx.doi.org/10.1136/jech.51.6.711

40. Nozza RJ, Sabo DL, Mandel EM. A role for otoacoustic emissions in screening for hearing impairment and middle ear disorders in school-age children. *Ear Hear* 1997;**18**:227–39. http://dx.doi.org/10.1097/00003446-199706000-00006

41. Siemens. *HearCheck Screener Userguide*. 2012. URL: www.connevans.info/image/connevans/38shearcheck.pdf (accessed 24 March 2015).

42. British Sociery of Audiology. *Pure-Tone Air-Conduction And Bone-Conduction Threshold Audiometry With And Without Masking*. 2012. URL: www.thebsa.org.uk/wp-content/uploads/2014/04/BSA_RP_PTA_FINAL_24Sept11_MinorAmend06Feb12.pdf (accessed 24 March 2015).

43. Taylor EJ, Emanuel DC. Assessment of the efficacy of a hearing screening program for college students. *J Am Acad Audiol* 2013;**24**:607–15. http://dx.doi.org/10.3766/jaaa.24.7.9

44. Sideris I, Glattke TJ. A comparison of two methods of hearing screening in the preschool population. *J Comm Disord* 2006;**39**:391–401. http://dx.doi.org/10.1016/j.jcomdis.2005.11.006

45. Bennett M, Mowat L. Validity of impedance measurements and referral criteria in school hearing screening programmes. *Br J Audiol* 1981;**15**:147–50. http://dx.doi.org/10.3109/03005368109081431

46. Grill E, Hessel F, Siebert U, Schnell-Inderst P, Kunze S, Nickisch A, *et al.* Comparing the clinical effectiveness of different new-born hearing screening strategies. A decision analysis. *BMC Public Health* 2005;**5**:12. http://dx.doi.org/10.1186/1471-2458-5-12

47. Olusanya BO, Bamigboye BA. Is discordance in TEOAE and AABR outcomes predictable in newborns? *Int J Pediatr Otorhinolaryngol* 2010;**74**:1303–9. http://dx.doi.org/10.1016/j.ijporl.2010.08.010

48. Mathew R, Asimacopoulos E, Valentine P. Toward safer practice in otology: a report on 15 years of clinical negligence claims. *Laryngoscope* 2011;**121**:2214–9. http://dx.doi.org/10.1002/lary.22136

© Queen's Printer and Controller of HMSO 2016. This work was produced by Fortnum *et al.* under the terms of a commissioning contract issued by the Secretary of State for Health. This issue may be freely reproduced for the purposes of private research and study and extracts (or indeed, the full report) may be included in professional journals provided that suitable acknowledgement is made and the reproduction is not associated with any form of advertising. Applications for commercial reproduction should be addressed to: NIHR Journals Library, National Institute for Health Research, Evaluation, Trials and Studies Coordinating Centre, Alpha House, University of Southampton Science Park, Southampton SO16 7NS, UK.

For peer review only - http://bmjopen.bmj.com/site/about/guidelines.xhtml

REFERENCES

49. Reilly BK, Horn GM, Sewell RK. Hearing loss resulting in malpractice litigation: what physicians need to know. *Laryngoscope* 2013;**123**:112–17. http://dx.doi.org/10.1002/lary.23608

50. Mason S, Davis A, Wood S, Farnsworth A. Field sensitivity of targeted neonatal hearing screening using the Nottingham ABR Screener. *Ear Hear* 1998;**19**:91–102. http://dx.doi.org/10.1097/00003446-199804000-00001

51. Lutman ME, Davis AC, Fortnum HM, Wood S. Field sensitivity of targeted neonatal hearing screening by transient-evoked otoacoustic emissions. *Ear Hear* 1997;**18**:265–76. http://dx.doi.org/10.1097/00003446-199708000-00001

52. Geal-Dor M, Adelman C, Levi H, Zentner G, Stein-Zamir C. Comparison of two hearing screening programs in the same population: oto-acoustic emissions (OAE) screening in newborns and behavioral screening when infants. *Int J Pediatr Otorhinolaryngol* 2010;**74**:1351–5. http://dx.doi.org/10.1016/j.ijporl.2010.08.023

53. Lo PS, Tong MC, Wong EM, van Hasselt CA. Parental suspicion of hearing loss in children with otitis media with effusion. *Eur J Pediatr* 2006;**165**:851–7. http://dx.doi.org/10.1007/s00431-006-0181-5

54. Office of National Statistics (ONS). *Mid-2013 Estimates of the Population*. URL: www.ons.gov.uk/ons/rel/pop-estimate/population-estimates-for-uk–england-and-wales–scotland-and-northern-ireland/2013/index.html (accessed 24 March 2015).

55. Government Department for communities and local government. *English Indices of Deprivation 2010*. 2011. URL: www.gov.uk/government/statistics/english-indices-of-deprivation-2010 (accessed 24 March 2015).

56. Ritchie J, Spencer L. Qualitative Data Analysis for Applied Policy Research. In Burgess BA, editor. *Analysing Qualitative Data*. London: Routledge; 1994. pp. 173–94. http://dx.doi.org/10.4324/9780203413081_chapter_9

57. HM Treasury. *The Green Book. Appraisal and Evaluation in Central Government*. 2003. URL: www.gov.uk/government/uploads/system/uploads/attachment_data/file/220541/green_book_complete.pdf (accessed 24 March 2015).

58. National Institute for Health and Care Excellence. *Developing NICE Guidelines: The Manual*. 2014. URL: www.nice.org.uk/article/pmg20/resources/non-guidance-developing-nice-guidelines-the-manual-pdf (accessed 24 March 2015).

59. Curtis L. *Unit Costs of Health and Social Care 2014*. Canterbury: PSSRU; 2014.

60. Department of Health. *Reference Costs 2012–2013*. London: Department of Health; 2013.

61. Barton GR, Bankart J, Davis AC, Summerfield QA. Comparing utility scores before and after hearing-aid provision: results according to the EQ-5D, HUI3 and SF-6D. *Appl Health Econ Health Policy* 2004;**3**:103–5. http://dx.doi.org/10.2165/00148365-200403020-00006

62. Summerfield AQ, Marshall DH, Barton GR, Bloor KE. A cost-utility scenario analysis of bilateral cochlear implantation. *Arch Otolaryngol Head Neck Surg* 2002;**128**:1255–62. http://dx.doi.org/10.1001/archotol.128.11.1255

63. Bisonni RS, Lawler FH, Pierce L. Recurrent otitis media: a cost-utility analysis of simulated treatment using tympanostomy tubes vs antibiotic prophylaxis. *Fam Pract Res J* 1991;**11**:371–8.

For peer review only

For peer review only - http://bmjopen.bmj.com/site/about/guidelines.xhtml

DOI: 10.3310/hta20360 HEALTH TECHNOLOGY ASSESSMENT 2016 VOL. 20 NO. 36

Appendix 1 Executive summary from 2007 report[12]

This text is reproduced from Bamford J, Fortnum H, Bristow K, Smith J, Vamvakas G, Davies L, *et al.* Current practice, accuracy, effectiveness and cost-effectiveness of the school entry hearing screen. *Health Technol Assess* 2007;**11**(32) under the Non-Commercial Government Licence for public sector information.

Background

The ability to hear is important, particularly during children's formal education. Hearing impairment is amenable to intervention and hence a screening programme when children begin their school careers has potential value. School entry hearing screening (SES) has been implemented throughout the UK since the 1950s. There is evidence of mixed practice and uncertainty about the value of the screen. In addition, recent changes in childhood hearing screening policy (abandonment of a screen at 8 months and introduction of universal newborn screening) have implications for identification of children with hearing impairment at school entry.

Objectives

This report aimed to determine answers to the following three questions:

- What is current practice for the SES in the UK?
- What is known about the accuracy of alternative screening tests and the effectiveness of interventions?
- What is known about costs, and what is the likely cost-effectiveness of the SES?

Methods

A national postal questionnaire survey was addressed to all leads for the SES in the UK, considering current practice in terms of implementation, protocols, target population and performance data. Primary data from cohort studies in one area of London were examined. A systematic review of alternative SES tests, test performance and impact on outcomes was carried out. Finally, a review of published studies on costs, plus economic modelling of current and alternative programmes was prepared.

Results

The evidence from the national survey of current practice is that:

- the SES is in place in most areas of England, Wales and Scotland; just over 10% of respondents have abandoned the screen; others are awaiting guidance in the light of the national implementation of newborn hearing screening
- coverage of the SES is variable, but is often over 90% for children in state schools
- referral rates are variable, with a median of about 8%
- the test used for the screen is the pure-tone sweep test but with wide variation in implementation, with differing frequencies, pass criteria and retest protocols; written examples of protocols were often poor and ambiguous
- there is no national approach to data collection, audit and quality assurance, and there are variable approaches at local level
- the screen is performed in less than ideal test conditions
- resources are often limited and this has an impact on the quality of the screen.

© Queen's Printer and Controller of HMSO 2016. This work was produced by Fortnum *et al.* under the terms of a commissioning contract issued by the Secretary of State for Health. This issue may be freely reproduced for the purposes of private research and study and extracts (or indeed, the full report) may be included in professional journals provided that suitable acknowledgement is made and the reproduction is not associated with any form of advertising. Applications for commercial reproduction should be addressed to: NIHR Journals Library, National Institute for Health Research, Evaluation, Trials and Studies Coordinating Centre, Alpha House, University of Southampton Science Park, Southampton SO16 7NS, UK.

For peer review only - http://bmjopen.bmj.com/site/about/guidelines.xhtml

The evidence from the primary cohort studies is that:

- the prevalence of permanent childhood hearing impairment continues to increase through infancy
- of the 3.47 in 1000 children with a permanent hearing impairment at school screen age, 1.89 in 1000 required identification after the newborn screen
- the introduction of newborn hearing screening is likely to reduce significantly the yield of SES for permanent bilateral and unilateral hearing impairments; yield had fallen from about 1.11 in 1000 before newborn screening to about 0.34 in 1000 for cohorts that had had newborn screening, of which only 0.07 in 1000 were unilateral impairments
- just under 20% of permanent moderate or greater bilateral, mild bilateral and unilateral impairments, known to services as 6-year-olds or older, remained to be identified around the time of school entry.

The evidence from the systematic review of the alternative tests and of the effectiveness of interventions is that:

- no good-quality published comparative trials of alternative screens or tests for school entry hearing screening were identified
- studies concerned with the relative accuracy of alternative tests are difficult to compare and often flawed by differing referral criteria and case definitions; with full pure-tone audiometry as the reference test, the pure-tone sweep test appears to have high sensitivity and high specificity for minimal, mild and greater hearing impairments, better than alternative tests for which evidence was identified
- there is insufficient evidence to draw any conclusions about possible harm of the screen
- there were no published studies identified that examined the possible effects of SES on longer-term outcomes.

The evidence from the cost-effectiveness study is that:

- no good-quality published economic evaluations of SES were identified
- a universal SES based on pure-tone sweep tests was associated with higher costs and slightly higher quality-adjusted life-years (QALYs) compared with no screen and other screen alternatives; the ICER for such a screen is around £2500 per QALY gained; the range of expected costs, QALYs and net benefits was broad, indicating a considerable degree of uncertainty
- targeted screening could be more cost-effective than universal SES
- lack of primary data and the wide limits for variables in the modelling mean that any conclusions must be considered indicative and exploratory only.

A national screening programme for permanent hearing impairment at school entry meets all but three of the criteria for a screening programme, but at least six criteria are not met for screening for transient hearing impairment.

Conclusions

The lack of good-quality evidence in this area remains a serious problem. Services should improve quality and audit screen performance for identification of previously unknown permanent hearing impairment, pending evidence-based policy decisions based on the research recommendations.

For peer review only

For peer review only - http://bmjopen.bmj.com/site/about/guidelines.xhtml

DOI: 10.3310/hta20360 HEALTH TECHNOLOGY ASSESSMENT 2016 VOL. 20 NO. 36

Recommendations for research

Further research is highlighted in the following areas:

- evaluation of an agreed national protocol for services delivering the SES to make future studies and audits of screen performance more directly comparable
- development and evaluation of systems for data monitoring so that robust data on screen performance are available
- determination with greater certainty of the prevalence of congenital unilateral hearing impairment, and permanent mild and minimal hearing impairment at school entry, that could be identified by a suitable quality-assured screen protocol
- a comparison of the effectiveness, efficacy and efficiency of alternative approaches (reactive services, formal surveillance, targeted screening and universal screening at school entry age) to the identification of permanent hearing impairment post newborn screen
- controlled studies of the effectiveness of hearing screening and subsequent interventions for later outcomes in children with permanent mild, minimal and unilateral hearing impairment identified at school entry
- determination of the distribution of detection thresholds for pure tones in the population at school entry.

For peer review only

© Queen's Printer and Controller of HMSO 2016. This work was produced by Fortnum *et al.* under the terms of a commissioning contract issued by the Secretary of State for Health. This issue may be freely reproduced for the purposes of private research and study and extracts (or indeed, the full report) may be included in professional journals provided that suitable acknowledgement is made and the reproduction is not associated with any form of advertising. Applications for commercial reproduction should be addressed to: NIHR Journals Library, National Institute for Health Research, Evaluation, Trials and Studies Coordinating Centre, Alpha House, University of Southampton Science Park, Southampton SO16 7NS, UK.

For peer review only - http://bmjopen.bmj.com/site/about/guidelines.xhtml

For peer review only

DOI: 10.3310/hta20360 HEALTH TECHNOLOGY ASSESSMENT 2016 VOL. 20 NO. 36

Appendix 2 Systematic review

Search strategies: accuracy of diagnostic tests

Ovid MEDLINE(R)

Date range of search: January 2005 to July 2014.

Date of search: 10 July 2014.

Search strategy

1 audiometry.mp. or audiometry/ or exp audiometry, pure-tone/

2 exp otoacoustic emissions, spontaneous/ or otoacoustic emission$.mp.

3 exp acoustic impedance tests/ or acoustic impedance.mp.

4 exp hearing tests/is, mt [instrumentation, methods]

5 hearing test$.mp.

6 sweep audio.mp.

7 sweep test$.mp.

8 (hearing adj2 questionnaire$).mp.

9 cmedhq.mp.

10 conventional audiometry.mp.

11 conditioned play audiometry.mp.

12 cpa.mp.

13 exp audiometry, evoked response/

14 audiologic$ assessment$.mp.

15 acoustic intermittance.tw.

16 tympanometry.mp.

For peer review only

© Queen's Printer and Controller of HMSO 2016. This work was produced by Fortnum *et al.* under the terms of a commissioning contract issued by the Secretary of State for Health. This issue may be freely reproduced for the purposes of private research and study and extracts (or indeed, the full report) may be included in professional journals provided that suitable acknowledgement is made and the reproduction is not associated with any form of advertising. Applications for commercial reproduction should be addressed to: NIHR Journals Library, National Institute for Health Research, Evaluation, Trials and Studies Coordinating Centre, Alpha House, University of Southampton Science Park, Southampton SO16 7NS, UK.

For peer review only - http://bmjopen.bmj.com/site/about/guidelines.xhtml

APPENDIX 2

17 otoscopy.mp. or exp otoscopy/ or exp

diagnostic techniques, otological/

18 otological exam$.mp.

19 acoustic reflex test$.mp.

20 teoae.mp.

21 dpoae.mp.

22 (impedance adj screening).mp.

23 (impedance adj method$).mp.

24 fixed frequency audio.mp.

25 (speech adj2 noise).mp.

26 reflectometry.mp.

27 acoustic impedance.mp.

28 or/1-27

29 exp hearing loss, sensorineural/pc, di

30 exp hearing disorders/di, pc

31 exp otitis media/pc, di

32 exp hearing loss, high-frequency/pc, di

33 exp hearing loss/di, pc

34 hearing impairment$.mp.

35 exp hearing loss, conductive/di, pc

36 (hearing adj3 screen$).mp.

37 exp "sensitivity and specificity"/

38 exp predictive value of tests/

39 (diagnos$ adj2 accura$).mp.

40 or/37-39

41 exp child, preschool/ or school entry.mp.

For peer review only

For peer review only - http://bmjopen.bmj.com/site/about/guidelines.xhtml

DOI: 10.3310/hta20360 HEALTH TECHNOLOGY ASSESSMENT 2016 VOL. 20 NO. 36

42 exp child development/

43 early detect$.mp.

44 infant school$.mp.

45 exp schools, nursery/ or exp nurseries/ or exp child day care centers/ or kindergarten$.mp.

46 nursery school$.mp.

47 or/41-46

48 screen$.mp. or exp mass screening/

49 (school entry adj3 (screen$ or exam$)).mp.

50 (medical exam$ adj2 school$).mp.

51 or/48-50

52 28 and 40

53 47 or 51

54 or/29-36

55 52 and 53

56 40 and 54

57 53 and 56

58 55 or 57

EMBASE

Date range search from: January 2005 to July 2014.

Date of search: 10 July 2014.

Search strategy

1 exp pure tone audiometry/ or exp audiometry/ or audiometry.mp.

2 otoacoustic emission$.mp. or exp spontaneous otoacoustic emission/ or exp otoacoustic emission/

3 acoustic impedance.mp. or exp acoustic

© Queen's Printer and Controller of HMSO 2016. This work was produced by Fortnum *et al.* under the terms of a commissioning contract issued by the Secretary of State for Health. This issue may be freely reproduced for the purposes of private research and study and extracts (or indeed, the full report) may be included in professional journals provided that suitable acknowledgement is made and the reproduction is not associated with any form of advertising. Applications for commercial reproduction should be addressed to: NIHR Journals Library, National Institute for Health Research, Evaluation, Trials and Studies Coordinating Centre, Alpha House, University of Southampton Science Park, Southampton SO16 7NS, UK.

For peer review only - http://bmjopen.bmj.com/site/about/guidelines.xhtml

For peer review only

APPENDIX 2

impedance/

4 hearing test$.mp.

5 exp hearing test/

6 sweep audio.mp.

7 sweep test$.mp.

8 (hearing adj2 questionnaire$).mp.

9 cmedhq.mp.

10 ((conventional or conditioned play) adj audiometry).mp.

11 cpa.mp.

12 exp evoked response audiometry/

13 (audiologic$ adj assessment$).mp.

14 (acoustic adj intermittance).mp.

15 tympanometry.mp. or exp tympanometry/

16 otoscopy.mp. or exp otoscopy/

17 (otological adj2 technique$).mp.

18 (otological adj2 (exam$ or technique$)).mp.

19 teoae.mp.

20 dpoae.mp. or exp distortion product otoacoustic emission/

21 (impedance adj (screen$ or method$)).mp.

22 fixed frequency audio.mp.

23 (speech adj2 noise).mp.

24 reflectometry.mp. or exp reflectometry/

25 or/1-24

26 hearing loss/pc, di [prevention, diagnosis]

For peer review only - http://bmjopen.bmj.com/site/about/guidelines.xhtml

DOI: 10.3310/hta20360 HEALTH TECHNOLOGY ASSESSMENT 2016 VOL. 20 NO. 36

27 hearing disorder/pc, di [prevention, diagnosis]

28 otitis media/pc, di [prevention, diagnosis]

29 hearing impair$.mp.

30 hearing impairment/pc, di [prevention, diagnosis]

31 (hearing adj3 screen$).mp.

32 exp “sensitivity and specificity”/

33 (predictive adj2 test$).mp.

34 (diagnos$ adj2 accura$).mp.

35 or/32-34

36 or/26-31

37 25 and 35

38 school entry.mp.

39 pre-school.mp.

40 child/

41 exp child development/

42 early detect$.mp.

43 infant school$.mp.

44 nursery school$.mp. or exp nursery school/

45 exp child care/ or child day care.mp.

46 kindergarten$.mp. or exp kindergarten/

47 or/38-46

48 screen$.mp.

49 exp mass screening/

50 (school entry adj3 (screen$ or exam$)).mp.

51 (medical exam$ adj2 school$).mp.

© Queen's Printer and Controller of HMSO 2016. This work was produced by Fortnum *et al.* under the terms of a commissioning contract issued by the Secretary of State for Health. This issue may be freely reproduced for the purposes of private research and study and extracts (or indeed, the full report) may be included in professional journals provided that suitable acknowledgement is made and the reproduction is not associated with any form of advertising. Applications for commercial reproduction should be addressed to: NIHR Journals Library, National Institute for Health Research, Evaluation, Trials and Studies Coordinating Centre, Alpha House, University of Southampton Science Park, Southampton SO16 7NS, UK.

For peer review only - http://bmjopen.bmj.com/site/about/guidelines.xhtml

52 or/48-49

53 or/50-51

54 25 and 35 and 47

55 35 and 36 and 47

56 54 or 55

57 or/52-53

58 47 or 57

59 35 and 36 and 58

60 56 or 59

Cumulative Index to Nursing and Allied Health Literature

Date range searched from: January 2005 to July 2014.

Date of search: 10 July 2014.

Search strategy

1 audiometry.mp. or exp audiometry, evoked

response/ or exp audiometry/ or exp

audiometry, pure-tone/

2 otoacoustic emission$.mp. or exp otoacoustic

emissions, spontaneous/

3 exp acoustic impedance tests/ or acoustic

impedance.mp.

4 hearing test$.mp. or exp hearing tests/

5 sweep test$.mp.

6 sweep audio.mp.

7 (hearing adj2 questionnaire$).mp.

8 cmedhq.mp.

9 (conventional adj2 audiometry).mp.

10 (conditioned adj2 audiometry).mp.

11 cpa.mp.

For peer review only - http://bmjopen.bmj.com/site/about/guidelines.xhtml

For peer review only

DOI: 10.3310/hta20360 HEALTH TECHNOLOGY ASSESSMENT 2016 VOL. 20 NO. 36

12 evoked response.mp. or exp evoked

potentials/

13 (audiologic$ adj assessment$).mp.

14 (acoustic adj intermittance).mp.

15 tympanometry.mp.

16 otoscopy.mp.

17 (otological adj2 technique$).mp.

18 (otological adj2 exam$).mp.

19 teoae.mp.

20 dpoae.mp. or exp otoacoustic emissions,

evoked/

21 (impedance adj (screen$ or method$)).mp.

22 fixed frequency audio.mp.

23 (speech adj2 noise).mp.

24 reflectometry.mp.

25 or/1-24

26 hearing disorders/di, pc

27 hearing impair$.mp.

28 exp hearing screening/

29 (hear$ adj2 screen$).mp.

30 or/26-29

31 exp "sensitivity and specificity"/

32 exp "predictive value of tests"/

33 (predictive adj2 test$).mp.

34 (diagnos$ adj2 accura$).mp.

35 or/31-34

36 school entry.mp.

© Queen's Printer and Controller of HMSO 2016. This work was produced by Fortnum *et al.* under the terms of a commissioning contract issued by the Secretary of State for Health. This issue may be freely reproduced for the purposes of private research and study and extracts (or indeed, the full report) may be included in professional journals provided that suitable acknowledgement is made and the reproduction is not associated with any form of advertising. Applications for commercial reproduction should be addressed to: NIHR Journals Library, National Institute for Health Research, Evaluation, Trials and Studies Coordinating Centre, Alpha House, University of Southampton Science Park, Southampton SO16 7NS, UK.

For peer review only - http://bmjopen.bmj.com/site/about/guidelines.xhtml

For peer review only

APPENDIX 2

37 exp child, preschool/ or pre-school.mp.

38 exp child development/

39 early detect$.mp.

40 infant school$.mp. or exp infant development/

41 nursery school$.mp. or exp schools, nursery/

42 child day care.mp. or exp child day care/

43 kindergarten$.mp.

44 or/36-43

45 screen$.mp.

46 exp hearing screening/

47 exp school admissions/

48 (school entry adj2 (screen$ or exam$)).mp.

49 (medical exam$ adj2 school$).mp.

50 or/45-46

51 or/47-49

52 25 and 35 and 44

53 30 and 35 and 44

54 52 or 53

55 50 or 51 or 44

56 30 and 35 and 55

57 54 or 56

PsycINFO

Date range searched: January 2005 to July 2014.

Date of search: 10 July 2014.

Search strategy

1 exp bone conduction audiometry/ or exp

audiometry/ or audiometry.mp.

2 otoacoustic emission$.mp.

For peer review only - http://bmjopen.bmj.com/site/about/guidelines.xhtml

For peer review only

DOI: 10.3310/hta20360 HEALTH TECHNOLOGY ASSESSMENT 2016 VOL. 20 NO. 36

3 acoustic impedance.mp.

4 hearing test$.mp.

5 sweep audio.mp.

6 sweep test$.mp.

7 (hearing adj2 questionnaire$).mp.

8 cmedhq.tw.

9 cpa.mp.

10 evoked response audiometry.mp.

11 audiologic$ assessment$.mp.

12 acoustic intermittance.tw.

13 tympanometry.mp.

14 otoscopy.mp.

15 (otological adj2 diagnos$).mp.

16 otological exam$.mp.

17 acoustic reflex test$.mp.

18 teoae.mp.

19 dpoae.mp.

20 (impedance adj screening).mp.

21 (impedance adj method$).mp.

22 fixed frequency audio.mp.

23 (speech adj2 noise).mp.

24 reflectometry.mp.

25 acoustic impedance.mp.

26 or/1-25

27 (sensitivity adj2 specificity).mp.

For peer review only

© Queen's Printer and Controller of HMSO 2016. This work was produced by Fortnum *et al.* under the terms of a commissioning contract issued by the Secretary of State for Health. This issue may be freely reproduced for the purposes of private research and study and extracts (or indeed, the full report) may be included in professional journals provided that suitable acknowledgement is made and the reproduction is not associated with any form of advertising. Applications for commercial reproduction should be addressed to: NIHR Journals Library, National Institute for Health Research, Evaluation, Trials and Studies Coordinating Centre, Alpha House, University of Southampton Science Park, Southampton SO16 7NS, UK.

For peer review only - http://bmjopen.bmj.com/site/about/guidelines.xhtml

APPENDIX 2

28 (predictive value adj2 test$).mp.

29 (diagnos$ adj2 accurac$).mp.

30 or/27-29

31 ((hearing loss$ or hearing disorder$ or hearing impair$ or otitis media) adj3 (diagnos$ or screen$)).mp.

32 30 or 31

33 26 and 32

34 child$.mp. or exp child day care/

35 exp early childhood development/ or exp preschool education/ or exp preschool students/ or pre-school.mp. or exp nursery schools/

36 kindergarten$.mp. or exp kindergartens/

37 nursery school$.mp.

38 exp elementary school students/ or infant school$.mp.

39 exp early intervention/ or early detect$.mp.

40 or/34-39

41 33 and 40

Education Resources Information Center (Cambridge Scientific Abstracts)

Date range searched: January 2005 to July 2014.

Search date: 10 July 2014.

Search strategy

(hearing or otitis) and (diagnos* or screen* or test*) and (school*or nurser* or infant*) and (accur* or predictive or sensitiv*)

For peer review only - http://bmjopen.bmj.com/site/about/guidelines.xhtml

DOI: 10.3310/hta20360 HEALTH TECHNOLOGY ASSESSMENT 2016 VOL. 20 NO. 36

Science Citation Index (Web of Knowledge)

Date range searched: January 2005 to July 2014.

Search date: 10 July 2014.

Search strategy

(Hearing or otitis or deaf*) and (screen* or test*

or diagnos*) and (accura* or predictive or

sensitive) and (pre-school or infant* or nurser* or school*

Full-text studies excluded from the review and reasons for exclusion

Anon. The EarCheck Middle Ear Monitor for detection of middle ear effusion in children. *Med Lett Drugs Ther* 2008;**50**:55–6. (Not a diagnostic accuracy study, description of device.)

Ahn JH, Lee HS, Kim YJ, Yoon TH, Chung JW. Comparing pure-tone audiometry and auditory steady state response for the measurement of hearing loss. *Otolaryngol Head Neck Surg* 2007;**136**:966–71. (Mean age around 20 years.)

Arnold L, Boyle P, Canning D. Development of a paediatric audiovisual speech test in noise. *Cochlear Implants Int* 2010;**11**(Suppl. 1):244–8. (Not a screening study, cochlear implant users.)

Bagatto MP, Brown CL, Moodie ST, Scollie SD. External validation of the LittlEARS(R) Auditory Questionnaire with English-speaking families of Canadian children with normal hearing. *Int J Pediatr Otorhinolaryngol* 2011;**75**:815–7. (Not a diagnostic accuracy study, children younger than 2 years.)

Bamford J, Fortnum H, Bristow K, Smith J, Vamvakas G, Davies L, *et al.* Current practice, accuracy, effectiveness and cost-effectiveness of the school entry hearing screen. *Health Technol Assess* 2007;**11**(32). (Systematic review, 2007 HTA report.)

Bhatia P, Mintz S, Hecht BF, Deavenport A, Kuo AA. Early identification of young children with hearing loss in federally qualified health centers. *J Dev Behav Pediatr* 2013;**34**:15–21. (Not a diagnostic accuracy study, children 0–3 years old.)

Blomgren K, Haapkyla J, Pitkaranta A. Tympanometry by nurses–can allocation of tasks be optimised? *Int J Pediatr Otorhinolaryngol* 2007;**71**:7–10. (Evaluate performance of nurses, aim is not screening but testing effectiveness of training.)

Bristow K, Fortnum H, Fonseca S, Bamford J. United Kingdom school-entry hearing screening: current practice. *Arch Dis Child* 2008;**93**:232–5. (Not a diagnostic accuracy study, survey included in the original HTA report.)

Chen G, Fu S, Luo S, Zhang W, Yang G. Screening of delayed-onset hearing loss in preschool children in the mid-south of China. *Int J Audiol* 2013;**52**:568–71. (Only patients with positive result on the index test received the reference standard.)

Dale OT, McCann LJ, Thio D, Wells SC, Drysdale AJ. The use of transient evoked otoacoustic emissions as a hearing screen following grommet insertion. *J Laryngol Otol* 2011;**125**:692–5. (Screening after grommet insertion, children 3–16 years old.)

© Queen's Printer and Controller of HMSO 2016. This work was produced by Fortnum *et al.* under the terms of a commissioning contract issued by the Secretary of State for Health. This issue may be freely reproduced for the purposes of private research and study and extracts (or indeed, the full report) may be included in professional journals provided that suitable acknowledgement is made and the reproduction is not associated with any form of advertising. Applications for commercial reproduction should be addressed to: NIHR Journals Library, National Institute for Health Research, Evaluation, Trials and Studies Coordinating Centre, Alpha House, University of Southampton Science Park, Southampton SO16 7NS, UK.

For peer review only - http://bmjopen.bmj.com/site/about/guidelines.xhtml

APPENDIX 2

Demorest ME, Wark DJ, Erdman SA. Development of the screening test for hearing problems. *Am J Audiol* 2011;**20**:100–10. (Screening tool for communication problems and psychological adjustment to hearing impairment.)

Dille M, Glattke TJ, Earl BR. Comparison of transient evoked otoacoustic emissions and distortion product otoacoustic emissions when screening hearing in preschool children in a community setting. *Int J Pediatr Otorhinolaryngol* 2007;**71**:1789–95. (Children's age ranged from 4 months to 4 years, most of them younger than 3 years; focus on agreement between tests, no 2 x 2 data reported.)

Eiserman WD, Hartel DM, Shisler L, Buhrmann J, White KR, Foust T. Using otoacoustic emissions to screen for hearing loss in early childhood care settings. *Int J Pediatr Otorhinolaryngol* 2008;**72**:475–82. (Children younger than 3 years.)

Eiserman WD, Shisler L. Identifying hearing loss in young children: technology replaces the Bell. *Zero Three (J)* 2010;**30**:24–8. (Review paper.)

El-Naggar M, Hashlamoun M. Paediatric hearing assessment and screening clinic at Fujairah: analysis of the results of the first 6 months of clinic practice. *Emirates Med J* 2005;**23**:15–20. (Unable to obtain an abstract or full text.)

Fasunla AJ, Adeosun AA, Afolabi AO, Nwaorgu OG. Usefulness of behavioral test of hearing as a rapid public health screening tool for infants. *J Pediatr Neurol* 2011;**9**:29–33. (Infants aged ≤ 12 months.)

Gierek T, Gwozdz-Jezierska M, Slaska-Kaspera A. [The evaluation of efficaciency of the 'Slysze' screening test on the base of the results of hearing examinations in schoolchildren in Silesia in 2002 year]. *Otolaryngologia Polska* 2007;**61**:707–12. (Children aged ≥ 7 years or older.)

Glasziou P. Review: self report of hearing loss and the whispered voice test are useful for screening for hearing impairment. *Evid Based Med* 2006;**11**:116. (Systematic review, adolescents over 16 years old.)

Halloran DR, Hardin JM, Wall TC. Validity of pure-tone hearing screening at well-child visits. *Arch Pediatr Adolesc Med* 2009;**163**:158–63. (Participants' age ranged from 3 to 19 years, no separate results for different age groups are reported.)

Kemper AR. Primary care hearing screening is of limited utility. *J Pediatr* 2009;**155**:448–9. (Summary of Halloran 2009.)

Khoza-Shangase K, Kassner L. Automated screening audiometry in the digital age: exploring Uhear and its use in a resource-stricken developing country. *Int J Technol Assess Health Care* 2013;**29**:42–7. (Children aged between 8 and 10 years.)

Kiese-Himmel C, Kruse E. [Who first suspects a hearing loss in infancy and childhood?] *HNO* 2005;**53**:810–16. (Not a diagnostic accuracy study, a survey of who and when first suspected hearing impairment.)

Liao W-H, Lien C-F, Young S-T. The hearing scale test for hearing screening of school-age children. *Int J Pediatr Otorhinolaryngol* 2010;**74**:760–4. (Children aged 9–10 years.)

Liu C, Xing G, Xu X, Chen Z, Zhou H, Wang D, *et al.* [The application of improved CHQS for mass epidemiology study on hearing impairment]. *Lin Chuang Er Bi Yan Hou Tou Jing Wai Ke Za Zhi* 2010;**24**:19–20. (Participants aged ≥ 7 years, adults as well as children.)

For peer review only - http://bmjopen.bmj.com/site/about/guidelines.xhtml

DOI: 10.3310/hta20360 HEALTH TECHNOLOGY ASSESSMENT 2016 VOL. 20 NO. 36

Lo AH, McPherson B. Hearing screening for school children: utility of noise-cancelling headphones. *BMC Ear, Nose and Throat Disord* 2013;**13**:6. (Not a data study, compares performance of different earphones, children between 6 and 8 years old.)

Margolis RH, Glasberg BR, Creeke S, Moore BC. AMTAS: automated method for testing auditory sensitivity: validation studies. *Int J Audiol* 2010;**49**:185–94. (No 2 × 2 data provided for the screening test, only for the tool designed to identify poor quality audiograms.)

Munoz K, Caballero A, White K. Effectiveness of questionnaires for screening hearing of school-age children: a comprehensive literature review. *Int J Audiol* 2014;**53**:910–4. (Systematic review.)

Parving A, Sørup Sørensen M, Christensen B, Davis A. Evaluation of a hearing screener. *Audiolog Med* 2008;**6**:115–9. (Participants between 20 and 86 years old.)

Perez MC, Gaya JA, Savio G, Perera M, Ponce de Leon M, Sanchez M. A 25-year review of Cuba's screening program for early detection of hearing loss. *MEDICC review* 2009;**11**:21–8. (Review paper.)

Sideris I, Glattke TJ. A comparison of two methods of hearing screening in the preschool population. *J Commun Disord* 2006;**39**:391–401. (Compares if PTA and TEOAE have the same referral rates and reasons for referral but no 2 x 2 data reported, children's age ranged from 2 years and 1 month to 5 years and 10 months.)

Sliwa L, Hatzopoulos S, Kochanek K, Pilka A, Senderski A, Skarzynski PH. A comparison of audiometric and objective methods in hearing screening of school children. A preliminary study. *Int J Pediatr Otorhinolaryngol* 2011;**75**:483–8. (Children between 10.9 and 14.9 years old.)

Stearn N, Swanepoel de W. AJTCAM/African Networks on Ethnomedicines. *Afr J Tradit Complement Altern Med* 2006;**4**:205–10. (Participants between 15 and 19 years old.)

Swanepoel DW. Early detection of infant hearing loss in South Africa. *S Afr Med J* 2009;**99**:158–9. (Review paper.)

Szudek J, Ostevik A, Dziegielewski P, Robinson-Anagor J, Gomaa N, Hodgetts B, *et al.* Can Uhear me now? Validation of an iPod-based hearing loss screening test. *J Otolaryngol Head Neck Surg* 2012;**41**(Suppl. 1):S78–84. (Adult participants.)

Taylor EJ, Emanuel DC. Assessment of the efficacy of a hearing screening program for college students. *J Am Acad Audiol* 2013;**24**:607–15. (Adults between 18 and 49 years old.)

Walker AN, G. The role of the school entry hearing screen in identifying childhood hearing impairment in reception age children. [Only children with positive screen (unspecified) received the reference standard.]

For peer review only

© Queen's Printer and Controller of HMSO 2016. This work was produced by Fortnum *et al.* under the terms of a commissioning contract issued by the Secretary of State for Health. This issue may be freely reproduced for the purposes of private research and study and extracts (or indeed, the full report) may be included in professional journals provided that suitable acknowledgement is made and the reproduction is not associated with any form of advertising. Applications for commercial reproduction should be addressed to: NIHR Journals Library, National Institute for Health Research, Evaluation, Trials and Studies Coordinating Centre, Alpha House, University of Southampton Science Park, Southampton SO16 7NS, UK.

For peer review only - http://bmjopen.bmj.com/site/about/guidelines.xhtml

APPENDIX 2

List of criteria used in the methodological quality assessment

1. Was the spectrum of participants representative of the population who will receive the test in practice?

   The spectrum was considered representative if the study included children 4–6 (± 1) years old at or around school entry stage who had been tested and found to have no hearing impairment at birth.

2. Were the selection criteria clearly described?
3. Is the reference standard likely to correctly classify the target condition?

   The reference standard was considered able to correctly classify the target condition if it included a combination of PTA and tympanometry performed in a suitable setting (quite room with ambient noise monitored) by a qualified professional.

4. Is the time period between reference standard and index test short enough to be reasonable?

   The time period between the performance of the index test and the reference standard was considered reasonable if it was < 4 weeks.

5. Did the whole sample or a random selection of the sample receive verification using a reference standard?
6. Did patients receive the same reference standard regardless of the index test result?
7. Was the reference standard independent of the index test?

   (i.e. the index test did not form part of the reference standard)

8. Was the execution of the index test described in sufficient detail to permit replication of the test?
9. Was the execution of the reference standard described in sufficient detail to permit its replication?
10. Were the index test results interpreted without knowledge of the results of the reference standard?
11. Were the reference standard results interpreted without knowledge of the results of the index test?
12. Were the same clinical data available when test results were interpreted as would be available when the test is used in practice?

    Testers usually have no prior information about children's hearing, so any information that may affect the interpretation of the index test was considered inappropriate.

13. Were uninterpretable/intermediate test results reported?
14. Were withdrawals explained?

For peer review only

For peer review only - http://bmjopen.bmj.com/site/about/guidelines.xhtml

DOI: 10.3310/hta20360 HEALTH TECHNOLOGY ASSESSMENT 2016 VOL. 20 NO. 36

Appendix 3 Diagnostic accuracy study: information

Case record form – cases p. 140.

Case record form – controls p. 144.

Participant information sheet – cases p. 147.

Participant summary information sheet – cases p. 150.

Children's pictorial information sheet – cases p. 152.

Participant information sheet – controls p. 153.

Participant summary information sheet – controls p. 156.

Children's information sheet – controls p. 157.

For peer review only

© Queen's Printer and Controller of HMSO 2016. This work was produced by Fortnum *et al.* under the terms of a commissioning contract issued by the Secretary of State for Health. This issue may be freely reproduced for the purposes of private research and study and extracts (or indeed, the full report) may be included in professional journals provided that suitable acknowledgement is made and the reproduction is not associated with any form of advertising. Applications for commercial reproduction should be addressed to: NIHR Journals Library, National Institute for Health Research, Evaluation, Trials and Studies Coordinating Centre, Alpha House, University of Southampton Science Park, Southampton SO16 7NS, UK.

For peer review only - http://bmjopen.bmj.com/site/about/guidelines.xhtml

APPENDIX 3

The diagnostic accuracy of hearing tests and cost-effectiveness of school entry hearing screening programmes

SES Study 1 Case child: ID number ☐☐☐☐☐

Research testing

Date of Screen: ☐☐/☐☐/☐☐☐☐ (DD/MM/YYYY)

Location of test: Clinic / Home / NHBRU / Other, please state ____________________
[circle as required]

Background noise level: ☐☐ dB Notes: ____
____

Background information

Location of hospital: ____________________

Date of Birth: ☐☐/☐☐/☐☐☐☐ (DD/MM/YYYY)

Gender [circle as required]: Male / Female

Post code: ☐☐☐☐ ☐

Ethnicity [tick as required]:

White	☐
Mixed / Multiple ethnic groups	☐
Asian / Asian British	☐
Black / African / Caribbean / Black British	☐
Other ethnic group	☐

Known medical conditions [List below]:

Procedural information [pre-randomised]

Order of sweep tests?

Pure Tone then Hearcheck ☐ Hearcheck then Pure Tone ☐

Order of ears for the 2 tests? [record overleaf too]

Left, right ☐ Right, left ☐

Pure Tone Sweep results

	Frequency detected? [tick if heard, cross if not heard]											
**First test** (20db)	1kHz			2kHz			4kHz			500Hz		
Left Ear												
	1kHz			2kHz			4kHz			500Hz		
Right Ear												

Initials of screener:

Time taken (mins):
☐☐

**Analysis:** ***(tick pass or fail)***	***Left Ear***	***Right Ear***
3/3 or 2/3 responses at every frequency	Pass ☐	Pass ☐
1/3 or 0/3 responses for at least one frequency	Fail ☐	Fail ☐

For peer review only - http://bmjopen.bmj.com/site/about/guidelines.xhtml

For peer review only

DOI: 10.3310/hta20360 HEALTH TECHNOLOGY ASSESSMENT 2016 VOL. 20 NO. 36

The diagnostic accuracy of hearing tests and cost-effectiveness of school entry hearing screening programmes

ID Number: ☐☐☐☐☐ DOB: ☐☐/☐☐/☐☐☐☐ Postcode: ☐☐☐☐ ☐

HearCheck Sweep Results

Which ear to test first? Left ☐ Right ☐

	1KHz			3KHz			
	55dB	35dB	20dB	75dB	55dB	35dB	
Left Ear							Total heard /6
Right Ear							Total heard /6

Initials of screener: ______

Time taken (mins): ☐☐

Analysis: ***(tick pass or fail)*** ***Left Ear*** ***Right Ear***

Heard all 6 tones in both ears? Pass ☐ Pass ☐

Heard 0-5 tones in either ear? Fail ☐ Fail ☐

Which test was actually done first? Hearcheck ☐ Pure Tone ☐

Blinding: **Did the second researcher know if the child passed or failed the first screen?**

Yes ☐ **No** ☐

If yes, please explain ____

Hearing History from Pure Tone Audiogram

Initials of researcher completing the table: ________

Date of PTA(dd/mm/yyyy):						
PTA attached?	Y / N					
**RESULTS**	Frequency					
	250Hz	500Hz	1kHz	2kHz	4kHz	8kHz
SF/Binaural AC (dBA)						
Right AC (dBHL)						
Left AC (dBHL)						
Right Unmasked BC (dBHL)						
Left Unmasked BC (dBHL)						
Right Masked BC (dBHL)						
Left Masked BC (dBHL)						
Circle as appropriate *(Required when there is no BC)*	Tymps Right			Tymps Left		
	A	B	C	A	B	C

KEY to read PTA:

○ Right AC
X Left AC
Δ Unmasked BC
[Right BC
] Left BC

0-20 dBHL Normal
21-40 Mild Loss
41-70 Moderate Loss
71-95 Severe Loss
> 95 Profound Loss

SES study 1 - CRF case children - v 1.3 26.09.13

NRES ref: 12/WM/0195, Sponsor ref: 12064, Ethics ref: 106333, NIHR HTA10/63/03

Page **2** of **4**

© Queen's Printer and Controller of HMSO 2016. This work was produced by Fortnum *et al.* under the terms of a commissioning contract issued by the Secretary of State for Health. This issue may be freely reproduced for the purposes of private research and study and extracts (or indeed, the full report) may be included in professional journals provided that suitable acknowledgement is made and the reproduction is not associated with any form of advertising. Applications for commercial reproduction should be addressed to: NIHR Journals Library, National Institute for Health Research, Evaluation, Trials and Studies Coordinating Centre, Alpha House, University of Southampton Science Park, Southampton SO16 7NS, UK.

For peer review only - http://bmjopen.bmj.com/site/about/guidelines.xhtml

APPENDIX 3

The diagnostic accuracy of hearing tests and cost-effectiveness of school entry hearing screening programmes

ID Number: ☐☐☐☐☐ DOB: ☐☐/☐☐/☐☐☐☐ Postcode: ☐☐☐☐ ☐

REPEAT AUDIOGRAM

Date of Repeat Audiogram(dd/mm/yyyy):						
PTA attached?	Y / N					
**RESULTS**	Frequency					
	250Hz	500Hz	1kHz	2kHz	4kHz	8kHz
SF/Binaural AC (dBA)						
Right AC (dBHL)						
Left AC (dBHL)						
Right Unmasked BC (dBHL)						
Left Unmasked BC (dBHL)						
Right Masked BC (dBHL)						
Left Masked BC (dBHL)						
Circle as appropriate *(Required when there is no BC)*	Tymps Right			Tymps Left		
	A	B	C	A	B	C

For peer review only - http://bmjopen.bmj.com/site/about/guidelines.xhtml

DOI: 10.3310/hta20360 HEALTH TECHNOLOGY ASSESSMENT 2016 VOL. 20 NO. 36

The diagnostic accuracy of hearing tests and cost-effectiveness of school entry hearing screening programmes

ID Number: □□□□□ DOB: □□/□□/□□□□ Postcode: □□□□ □

Would parent(s) like a summary of the project to be sent to them? **Y / N**

[Note down preferred contact details]

Is another PTA required (out of date/incomplete?)

Details of the request (BY PHONE) for repeat audiogram:

Name of audiologist: ______________________________

Name of researcher: _________________________ *[signature]*

Date of request: □□/□□/□□□□ (DD/MM/YYYY)

© Queen's Printer and Controller of HMSO 2016. This work was produced by Fortnum *et al.* under the terms of a commissioning contract issued by the Secretary of State for Health. This issue may be freely reproduced for the purposes of private research and study and extracts (or indeed, the full report) may be included in professional journals provided that suitable acknowledgement is made and the reproduction is not associated with any form of advertising. Applications for commercial reproduction should be addressed to: NIHR Journals Library, National Institute for Health Research, Evaluation, Trials and Studies Coordinating Centre, Alpha House, University of Southampton Science Park, Southampton SO16 7NS, UK.

For peer review only - http://bmjopen.bmj.com/site/about/guidelines.xhtml

For peer review only

APPENDIX 3

The diagnostic accuracy of hearing tests and cost-effectiveness of school entry hearing screening programmes

SES Study 1 Control child – ID number ☐☐☐☐☐

Research testing

Date of screen: ☐☐/☐☐/☐☐☐☐ (DD/MM/YYYY)

Location of test [circle as required]: Clinic / NHBRU / Other, please state _______________

Background noise level: ☐☐ dB Notes: ____

____

Background information

Name of School: ______________________ Date of Birth: ☐☐/☐☐/☐☐☐☐ (DD/MM/YYYY)

Gender [circle as required]: Male / Female Post code: ☐☐☐☐ ☐

Ethnicity [tick as required]: Known medical conditions [List below]:

White	☐
Mixed / Multiple ethnic groups	☐
Asian / Asian British	☐
Black / African / Caribbean / Black British	☐
Other ethnic group	☐

<u>Procedural information [pre-randomised]</u>

Order of sweep tests?

Pure Tone then Hearcheck ☐ Hearcheck then Pure Tone ☐

Order of ears for the 3 tests? [Record overleaf too]

Left, right, left ☐ Right, left, right ☐

<u>Pure Tone Sweep results</u>

	Frequency detected? [tick if heard, cross if not heard]											
**First test** (20db)	1kHz			2kHz			4kHz			500Hz		
Left Ear												
	1kHz			2kHz			4kHz			500Hz		
Right Ear												

Time taken (mins): ☐☐

Analysis: ***(tick pass or fail)*** ***Left Ear*** ***Right Ear***

3/3 or 2/3 responses at every frequency Pass ☐ Pass ☐

1/3 or 0/3 responses for at least one frequency Fail ☐ Fail ☐

Initials of screener .

<u>HearCheck Sweep Results</u>

	1KHz			3KHz			
	55dB	35dB	20dB	75dB	55dB	35dB	
Left Ear							Total heard /6
Right Ear							Total heard /6

Time taken (mins): ☐☐

Analysis: ***(tick pass or fail)*** ***Left Ear*** ***Right Ear***

Heard all 6 tones in both ears? Pass ☐ Pass ☐

Heard 0-5 tones in either ear? Fail ☐ Fail ☐

Which test was actually done first? Hearcheck ☐ Pure Tone ☐

SES study 1 - CRF control children - v 1.3 **27.09.13** Page **1** of **3**

NRES ref: 12/WM/0195, Sponsor ref: 12064, Ethics ref: 106333, NIHR HTA10/63/03

For peer review only - http://bmjopen.bmj.com/site/about/guidelines.xhtml

For peer review only

DOI: 10.3310/hta20360 HEALTH TECHNOLOGY ASSESSMENT 2016 VOL. 20 NO. 36

The diagnostic accuracy of hearing tests and cost-effectiveness of school entry hearing screening programmes ID

Number: ☐☐☐☐☐ DOB: ☐☐/☐☐/☐☐☐☐ Postcode: ☐☐☐☐ ☐

PURE TONE AUDIOGRAM (Gold standard)

[2nd researcher to conduct and enter results only after both screening tests have been completed by the 1 researcher]

Which ear to test first? Left ☐ Right ☐

Initials of tester: ________

Time taken (mins): ☐☐

RESULTS	Frequency					
	1kHz	2kHz	4kHz	8kHz	500Hz	250Hz
Right AC (dBHL)						
Left AC (dBHL)						

KEY to read PTA:

0-20 dBHL Normal
21-40 Mild Loss
41-70 Moderate Loss
71-95 Severe Loss
> 95 Profound Loss

PTA results attached (printed out)? Y / N

PTA Result: Normal / Refer [circle as required]

[Refer to Audiology if any frequencies ≥30dBHL are not heard]

Blinding: Did the second researcher know if the child passed or failed the screening tests?

Yes ☐ **No** ☐

If yes, please explain ____

© Queen's Printer and Controller of HMSO 2016. This work was produced by Fortnum *et al.* under the terms of a commissioning contract issued by the Secretary of State for Health. This issue may be freely reproduced for the purposes of private research and study and extracts (or indeed, the full report) may be included in professional journals provided that suitable acknowledgement is made and the reproduction is not associated with any form of advertising. Applications for commercial reproduction should be addressed to: NIHR Journals Library, National Institute for Health Research, Evaluation, Trials and Studies Coordinating Centre, Alpha House, University of Southampton Science Park, Southampton SO16 7NS, UK.

For peer review only - http://bmjopen.bmj.com/site/about/guidelines.xhtml

For peer review only

The diagnostic accuracy of hearing tests and cost-effectiveness of school entry hearing screening programmes ID

Number: ☐☐☐☐☐ DOB: ☐☐/☐☐/☐☐☐☐ Postcode: ☐☐☐☐ ☐

Would parent(s) like a summary of the project to be sent to them? **Y / N**

[Note down preferred contact details]

Referral to Nottingham Audiology Services Required? Y / N

Name of Child: ______________________________ Name of Parent: _____________________________

Address: ____

____

Telephone Number: _________________________ Signature of Parent: _________________________

If referred*, explain to parents and give them a letter and leaflet to take home*

Copy the CRF and pass to Claire or Shelly

For peer review only

For peer review only - http://bmjopen.bmj.com/site/about/guidelines.xhtml

DOI: 10.3310/hta20360 HEALTH TECHNOLOGY ASSESSMENT 2016 VOL. 20 NO. 36

(Form to be printed on NHBRU headed paper)

INFORMATION SHEET
(S1 Cases v1.3 20.09.13)

The diagnostic accuracy of hearing tests and cost-effectiveness of school entry hearing screening programmes

Name of Researcher(s): Dr Heather Fortnum, Ms Sam Catterick and Ms Mara Ozolins

Invitation
We would like to invite your child to take part in a research study. Before you decide whether you want to do that, it is important for you to understand why the research is being done and what it will involve. If you would like, one of our team will go through the information sheet with you and answer any questions you have. Talk to others about the study if you wish. Ask us if there is anything that is not clear or if you would like more information.

Why has my child been chosen?
Your child is being invited to take part because we know they have a hearing loss. We are inviting 80 children like your child to take part. We are also inviting 160 children who do not have a hearing loss to take part. Because we know whether or not the children taking part in this study have a hearing loss we can tell if the screening tests we are assessing are able to correctly identify those with and without a hearing loss.

Who are the researchers?
The research is being led by Dr Heather Fortnum, an Associate Professor and Reader in Hearing Research at the University of Nottingham. She is working with two research fellows, Sam Catterick and Mara Ozolins who will see the children in this study. The research also involves audiologists in Nottingham and Cambridge, and research methods experts in Exeter.

What is the purpose of the study?
Identifying children who have a permanent hearing loss at the earliest possible age is very important. When a hearing loss is detected early, the child's speech and language is usually better and they do better in school. There is now a hearing test at birth for all babies and this means that the vast majority of children born with a hearing loss are identified at birth. However, not all children who will eventually have a hearing loss have that hearing loss at birth.

In most parts of the UK at the moment, children have another hearing test when they start school, using a machine to screen for permanent hearing loss. However, this might not be the best test to use. A new system using a hand-held device to test hearing might be more accurate, and quicker and easier for the children and for the school nurses who do the testing. One aim of this project is to compare two types of hearing tests which can be used in schools to find out which one is better able to identify hearing loss in children.

Does my child have to take part?
It is up to you to decide whether or not he or she takes part. If you do decide that your child will take part, you will be asked to sign a consent form. If you later decide that he/she no longer wishes to take part, please inform us and he/she will be withdrawn from the study. You do not need to give a reason and it will not affect the standard of care your child receives.

What will happen to my child if they take part?
The research will involve just one session of testing with your child. If your child wears hearing aids, they will be asked to remove these before we test them. We will test your child's hearing with the screening system currently used in schools and with the new handheld device. Each gives out sounds at levels up to the equivalent of a noisy room and your child will need to tell us when they hear a

© Queen's Printer and Controller of HMSO 2016. This work was produced by Fortnum *et al.* under the terms of a commissioning contract issued by the Secretary of State for Health. This issue may be freely reproduced for the purposes of private research and study and extracts (or indeed, the full report) may be included in professional journals provided that suitable acknowledgement is made and the reproduction is not associated with any form of advertising. Applications for commercial reproduction should be addressed to: NIHR Journals Library, National Institute for Health Research, Evaluation, Trials and Studies Coordinating Centre, Alpha House, University of Southampton Science Park, Southampton SO16 7NS, UK.

For peer review only - http://bmjopen.bmj.com/site/about/guidelines.xhtml

sound by pressing a button. Each ear will be tested separately. The first system plays the sounds via headphones and the second system plays the sounds from a small machine held next to the child's ear.

We also need to compare the two screening hearing tests with a full test of your child's hearing. This will be done in one of two ways:

1. If your child has had a recent hearing test at your local audiology service, or has an appointment for a hearing test in the next 3 months we will just need to access a copy of these results with your permission.
2. If they have not had a hearing test in the last 12 months and do not have an appointment to have a hearing test in the next 3 months we would need to do a full hearing test in your local audiology clinic or in the research facility in Nottingham.

Where will the tests take place?
We will carry out these tests in your own home or at our research facility at Ropewalk House in the centre of Nottingham. You will be able to choose which is most convenient for you. The research session should take no longer than 30 minutes in total and you can be with your child at all times.

When will my child take part?
The researchers intend to test children just once between December 2012 and October 2014 and your child could be included at any point during this time. Therefore although we would like to know now whether you would like your child to take part, please be aware that you will not be invited for testing until your child is at least 4yrs old.

Expenses and payments
We will pay for all your travel expenses to attend for the hearing tests and each child will be offered a book token to the value of £20 to say thank you for taking part.

What are the possible disadvantages or risks of taking part?
There should be no risk or discomfort for your child. The loudest sound that they will listen to is approximately the equivalent of a noisy room.

What are the advantages of taking part?
We cannot promise the study will help your child but the information we get from this study may help to decide how best to detect hearing loss in children in the future.

What if I have any concerns?
If you have a concern about any aspect of this study, you should ask to speak to the researchers who will do their best to answer your questions. The researchers' contact details are given at the end of this information sheet. If you are still unhappy and wish to complain formally, you can do this by contacting NHS Complaints *<<PALS number for the appropriate hospital to be inserted>>.*

Will my child taking part in this study be kept confidential?
All information about your child will be handled in confidence.

If your child joins the study, some parts of audiology records and the data collected for the study will be looked at by authorised persons from the University of Nottingham who are organising the research. The data may also be looked at by authorised people to check that the study is being carried out correctly. All will have a duty of confidentiality to your child as a research participant and we will do our best to meet this duty.

All information which is collected about your child during the course of the research will be kept **strictly confidential**, stored in a secure and locked office, and on a password protected database. Any information about your child which leaves the hospital will have your child's name and address removed (anonymised) and a unique code will be used so that they cannot be recognised from it.

Your child's personal data (address, telephone number) will be kept for up to 12 months after the end of the study. All research data will be kept securely for 7 years following publication. After this time your child's data will be disposed of securely. During this time all precautions will be taken by

For peer review only

For peer review only - http://bmjopen.bmj.com/site/about/guidelines.xhtml

DOI: 10.3310/hta20360 HEALTH TECHNOLOGY ASSESSMENT 2016 VOL. 20 NO. 36

all those involved to maintain your child's confidentiality; only members of the research team will have access to their personal data.

What will happen if I do not want my child to carry on with the study?
Taking part in the study is voluntary and you are free to withdraw your child at any time, without giving any reason, and without their legal rights being affected. If you withdraw your child, then the information collected so far cannot be deleted and this information may still be used in the project analysis.

What will happen to the results of the study?
The results of the study will be written up into a report for the National Institute for Health Research who are funding the study. We will also publish the results in academic journals and present the results at academic and clinical conferences. The results will feed into government decisions about the best way to screen for hearing loss in children. We will send you a summary of the results if you would like to receive it.

Who is organising and funding the research?
This research is being organised by the University of Nottingham and is being funded by the National Institute for Health Research, Health Technology Assessment Programme.

Who has reviewed this study?
All research in the NHS is looked at by independent group of people, called a Research Ethics Committee, to protect participant's interests. This study has been reviewed and given favourable opinion by the West Midlands, Staffordshire Research Ethics Committee.

Further information and contact details
If you have any questions or would like to talk to someone about this research, please contact either the Chief Investigator, Dr Heather Fortnum or the study researchers, Sam Catterick or Mara Ozolins on 0115 8232600 or email us at SES@nottingham.ac.uk. Alternatively, please write to: Heather Fortnum at the Nottingham Hearing Biomedical Research Unit, Ropewalk House, 113 The Ropewalk, Nottingham, NG1 5DU.

Thank you for reading this

© Queen's Printer and Controller of HMSO 2016. This work was produced by Fortnum *et al.* under the terms of a commissioning contract issued by the Secretary of State for Health. This issue may be freely reproduced for the purposes of private research and study and extracts (or indeed, the full report) may be included in professional journals provided that suitable acknowledgement is made and the reproduction is not associated with any form of advertising. Applications for commercial reproduction should be addressed to: NIHR Journals Library, National Institute for Health Research, Evaluation, Trials and Studies Coordinating Centre, Alpha House, University of Southampton Science Park, Southampton SO16 7NS, UK.

For peer review only - http://bmjopen.bmj.com/site/about/guidelines.xhtml

APPENDIX 3

National Institute for Health Research

Nottingham Hearing
Biomedical Research Unit
Ropewalk House
113 The Ropewalk
Nottingham NG1 5DU

Tel: +44 (0) 115 823 2600
Fax: +44 (0) 115 823 2618
Email: nhbru-enquiries@nottingham.ac.uk
Web: www.hearing.nihr.ac.uk

For peer review only

SUMMARY INFORMATION SHEET

(S1 Cases v1.1 20.09.13)

Are hearing tests at school accurate and cost effective?

What is the study about?

Most children in the UK have a hearing test when they start school. This study will compare different ways of testing hearing to see which is best.

Why has my child been chosen?

We are inviting 80 children with a hearing loss (like your child) and 160 children who do not have a hearing loss to take part. We want to see if the hearing tests used can correctly identify those with and without a hearing loss.

What will my child have to do?

We will either come to your home or you can come to us at Ropewalk House in the centre of Nottingham. Your child's hearing will be tested using the hearing tests. Each test will play sounds and your child will need to tell us when they hear them by pressing a button. Each ear will be tested separately. The first test plays the sounds over headphones and the second test plays the sounds from a small machine (like a telephone) held next to your child's ear. The research session should take no longer than 30 minutes in total.

When will my child take part?

We will test children just once between December 2012 and October 2014.

The National Institute for Health Research Nottingham Hearing Biomedical Research Unit is a partnership between the University of Nottingham, Nottingham University Hospitals NHS Trust and the Medical Research Council (MRC) Institute of Hearing Research

For peer review only - http://bmjopen.bmj.com/site/about/guidelines.xhtml

DOI: 10.3310/hta20360 HEALTH TECHNOLOGY ASSESSMENT 2016 VOL. 20 NO. 36

What are the advantages of taking part?
The study may not help your child, but the information could help to decide how hearing loss should be tested in children in the future.

Are there any risks to taking part?
There should be no risk or discomfortf or your child. The loudest sound that they will listen to is approximately the equivalent of a noisy room.

Will my child's information be kept confidential?
All information about your child will be handled in confidence.

Will we receive any payment?
We will pay for any travel expenses and your child will be given a £20 book token to say thank you.

For further information or contact details of the researchers, please read the detailed information sheet included in this pack.

For peer review only

<u>If you would like to support the research, please return the reply slip to us in the prepaid envelope provided.</u>

The diagnostic accuracy of hearing tests and cost-effectiveness of school entry hearing screening programmes
Summary Information Sheet Study 1 Cases Version 1.1 Date: 20.09.13

The National Institute for Health Research Nottingham Hearing Biomedical Research Unit is a partnership between the University of Nottingham, Nottingham University Hospitals NHS Trust and the Medical Research Council (MRC) Institute of Hearing Research

© Queen's Printer and Controller of HMSO 2016. This work was produced by Fortnum *et al.* under the terms of a commissioning contract issued by the Secretary of State for Health. This issue may be freely reproduced for the purposes of private research and study and extracts (or indeed, the full report) may be included in professional journals provided that suitable acknowledgement is made and the reproduction is not associated with any form of advertising. Applications for commercial reproduction should be addressed to: NIHR Journals Library, National Institute for Health Research, Evaluation, Trials and Studies Coordinating Centre, Alpha House, University of Southampton Science Park, Southampton SO16 7NS, UK.

For peer review only - http://bmjopen.bmj.com/site/about/guidelines.xhtml

APPENDIX 3

Children's pictorial information sheet for children recruited as cases and seen in the research facility

Page 1

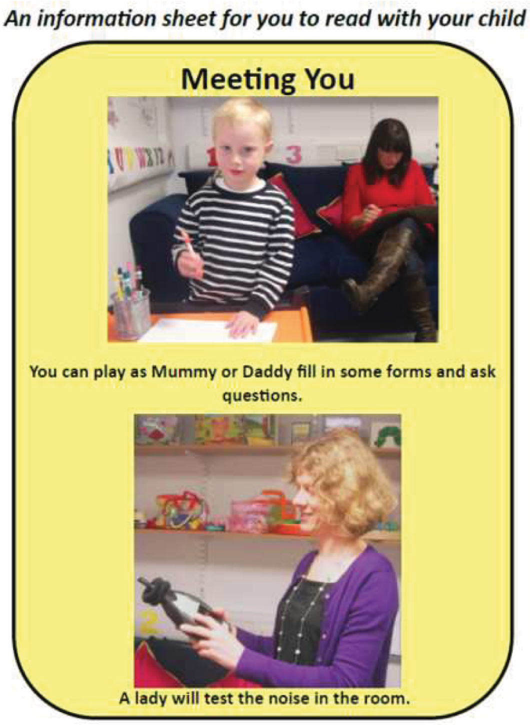

Page 2

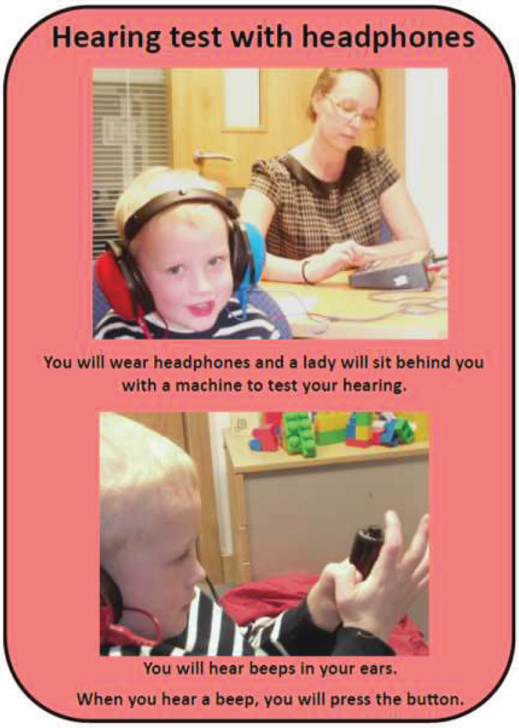

Page 3

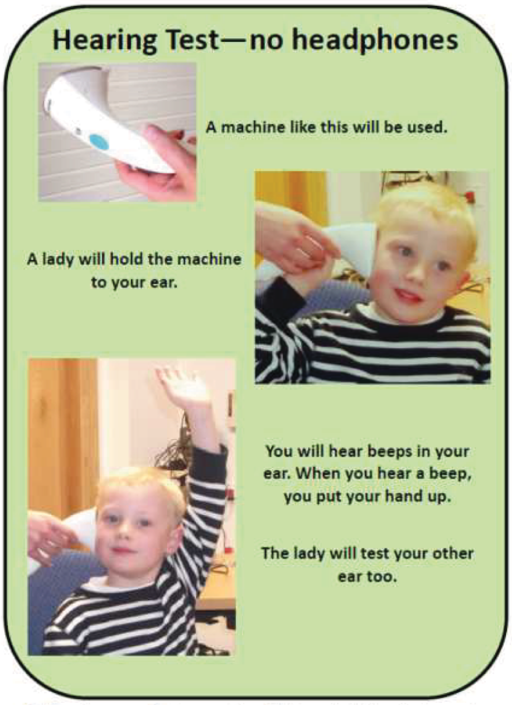

Page 4

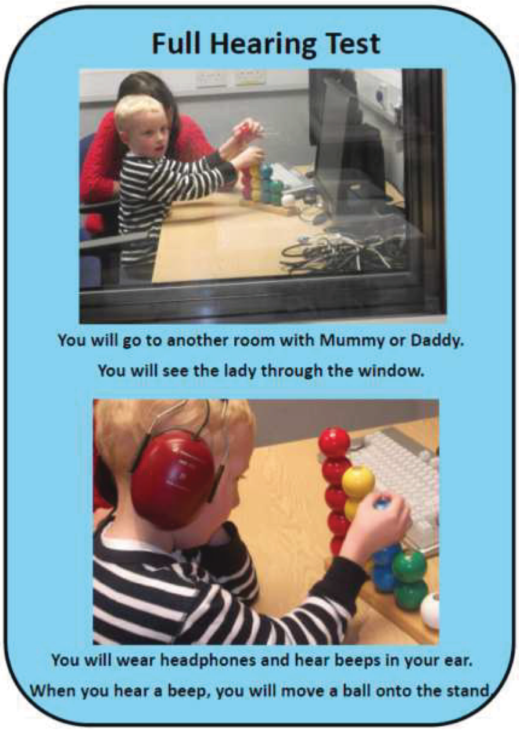

The diagnostic accuracy of hearing tests and cost-effectiveness of school entry hearing screening programmes. Children's information sheet. Study 1 controls v1.1 Date 05.12.12

For peer review only - http://bmjopen.bmj.com/site/about/guidelines.xhtml

DOI: 10.3310/hta20360 HEALTH TECHNOLOGY ASSESSMENT 2016 VOL. 20 NO. 36

The University of Nottingham

NHS
National Institute for Health Research

Nottingham Hearing Biomedical Research Unit

INFORMATION SHEET
(S1 Controls v1.3: 20.09.13)
The diagnostic accuracy of hearing tests and cost-effectiveness of school entry hearing screening programmes

Name of Researcher(s): Dr Heather Fortnum, Ms Sam Catterick and Ms Mara Ozolins

Invitation
We would like to invite your child to take part in a research study. Before you decide whether you want to do that, it is important for you to understand why the research is being done and what it will involve. One of our team will go through the information sheet with you and answer any questions you have. Talk to others about the study if you wish. Ask us if there is anything that is not clear or if you would like more information.

Why has my child been chosen?
Your child is being invited to take part because to the best of our knowledge they have normal hearing. We are inviting 160 children like your child to take part. We are also inviting 80 children who do have a hearing loss to take part. Because we know whether or not the children taking part in this study have a hearing loss we can tell if the screening tests we are assessing are able to correctly identify those with and without a hearing loss.

Who are the researchers?
The research is being led by Dr Heather Fortnum, an Associate Professor and Reader in Hearing Research at the University of Nottingham. She is working with two research fellows, Sam Catterick and Mara Ozolins who will see the children in this study. The research also involves audiologists in Nottingham and Cambridge, and research methods experts in Exeter.

What is the purpose of the study?
Identifying children who have a permanent hearing loss at the earliest possible age is very important. When a hearing loss is detected early, the child's speech and language is usually better and they do better in school. There is now a hearing test at birth for all babies and this means that the vast majority of children born with a hearing loss are identified at birth. However, not all children who will eventually have a hearing loss have that hearing loss at birth.

In most parts of the UK at the moment, children have another hearing test when they start school, using a machine to screen for permanent hearing loss. However, this might not be the best test to use. A new system using a hand-held device to test hearing might be more accurate, and quicker and easier for the children and for the school nurses who do the testing. One aim of this project is to compare two types of hearing tests which can be used in schools to find out which one is better able to identify hearing loss in children.

The National Institute for Health Research Nottingham Hearing Biomedical Research Unit is a partnership between the University of Nottingham, Nottingham University Hospitals NHS Trust and the Medical Research Council (MRC) Institute of Hearing Research

© Queen's Printer and Controller of HMSO 2016. This work was produced by Fortnum *et al.* under the terms of a commissioning contract issued by the Secretary of State for Health. This issue may be freely reproduced for the purposes of private research and study and extracts (or indeed, the full report) may be included in professional journals provided that suitable acknowledgement is made and the reproduction is not associated with any form of advertising. Applications for commercial reproduction should be addressed to: NIHR Journals Library, National Institute for Health Research, Evaluation, Trials and Studies Coordinating Centre, Alpha House, University of Southampton Science Park, Southampton SO16 7NS, UK.

For peer review only - http://bmjopen.bmj.com/site/about/guidelines.xhtml

For peer review only

Does my child have to take part?
It is up to you to decide whether or not he or she takes part. If you do decide that your child will take part, you will be asked to sign a consent form. If you later decide that he/she no longer wishes to take part, please inform us and he/she will be withdrawn from the study. You do not need to give a reason and it will not affect the standard of care your child receives.

What will happen to my child if they take part?
The research will involve just one session of testing with your child. We will test your child's hearing with the screening system currently used in schools and with the new handheld device. Each gives out sounds at levels up to the equivalent of a noisy room and your child will need to tell us when they hear a sound by pressing a button. Each ear will be tested separately. The first system plays the sounds via headphones and the second system plays the sounds from a small machine held next to the child's ear.

We also need to compare the two screening hearing tests with a full test of your child's hearing. This involves your child listening through headphones to a longer series of tones and indicating to the researcher when they can hear something by moving an object.

Where will the tests take place?
We will carry out these tests at our research facility at Ropewalk House in the centre of Nottingham. The research session should take no longer than 45 minutes and you can be with your child at all times.

When will my child take part?
The researchers intend to test children just once between December 2012 and October 2014 and your child could be included at any point during this time. Therefore although we would like to know now whether you would like your child to take part, we may not arrange the research appointment straight away.

What will happen if you find that my child has a hearing loss?
If the hearing tests indicate that your child might have a hearing loss we will give you a letter explaining that we will refer your child for an appointment at the local audiology clinic to have a further test of their hearing and where you can talk to a hearing specialist.

Expenses and payments
We will pay for all your travel expenses to attend for the hearing tests and each child will be offered a book token to the value of £20 to say thank you for taking part.

What are the possible disadvantages or risks of taking part?
There should be no risk or discomfort for your child. The loudest sound that they will listen to is approximately the equivalent of a noisy room.

What are the advantages of taking part?
We cannot promise the study will help your child but the information we get from this study may help to decide how best to detect hearing loss in children in the future.

What if I have any concerns?
If you have a concern about any aspect of this study, you should ask to speak to the researchers who will do their best to answer your questions. The researchers' contact details are given at the end of this information sheet. If you are still unhappy and wish to complain formally, you can do this by contacting NHS Complaints on 0800 0153367.

Will my child taking part in this study be kept confidential?
All information about your child will be handled in confidence.

The diagnostic accuracy of hearing tests and cost-effectiveness of school entry hearing screening programmes
Information Sheet Study 1 controls Version 1.3 Date: 20.09.13

The National Institute for Health Research Nottingham Hearing Biomedical Research Unit is a partnership between the University of Nottingham, Nottingham University Hospitals NHS Trust and the Medical Research Council (MRC) Institute of Hearing Research

For peer review only - http://bmjopen.bmj.com/site/about/guidelines.xhtml

DOI: 10.3310/hta20360 HEALTH TECHNOLOGY ASSESSMENT 2016 VOL. 20 NO. 36

If your child joins the study, some parts of the data collected for the study will be looked at by authorised persons from the University of Nottingham who are organising the research. The data may also be looked at by authorised people to check that the study is being carried out correctly. All will have a duty of confidentiality to your child as a research participant and we will do our best to meet this duty.

All information which is collected about your child during the course of the research will be kept **strictly confidential**, stored in a secure and locked office, and on a password protected database. Any information about your child which leaves the research facility will have your child's name and address removed (anonymised) and a unique code will be used so that they cannot be recognised from it.

Your child's personal data (address, telephone number) will be kept for up to 12 months after the end of the study. All research data will be kept securely for 7 years following publication. After this time your child's data will be disposed of securely. During this time all precautions will be taken by all those involved to maintain your child's confidentiality; only members of the research team will have access to their personal data.

What will happen if I do not want my child to carry on with the study?
Taking part in the study is voluntary and you are free to withdraw your child at any time, without giving any reason, and without their legal rights being affected. If you withdraw your child, then the information collected so far cannot be deleted and this information may still be used in the project analysis.

What will happen to the results of the study?
The results of the study will be written up into a report for the National Institute for Health Research who are funding the study. We will also publish the results in academic journals and present the results at academic and clinical conferences. The results will feed into government decisions about the best way to screen for hearing loss in children. We will send you a summary of the results if you would like to receive it.

Who is organising and funding the research?
This research is being organised by the University of Nottingham and is being funded by the National Institute for Health Research, Health Technology Assessment Programme.

Who has reviewed this study?
All research in the NHS is looked at by independent group of people, called a Research Ethics Committee, to protect participant's interests. This study has been reviewed and given favourable opinion by the West Midlands, Staffordshire Research Ethics Committee.

Further information and contact details
If you have any questions or would like to talk to someone about this research, please contact either the Chief Investigator, Dr Heather Fortnum or the study researchers, Sam Catterick or Mara Ozolins on 0115 8232600 or email us at SES@nottingham.ac.uk. Alternatively, please write to: Heather Fortnum at the Nottingham Hearing Biomedical Research Unit, Ropewalk House, 113 The Ropewalk, Nottingham, NG1 5DU.

Thank you for reading this

© Queen's Printer and Controller of HMSO 2016. This work was produced by Fortnum *et al.* under the terms of a commissioning contract issued by the Secretary of State for Health. This issue may be freely reproduced for the purposes of private research and study and extracts (or indeed, the full report) may be included in professional journals provided that suitable acknowledgement is made and the reproduction is not associated with any form of advertising. Applications for commercial reproduction should be addressed to: NIHR Journals Library, National Institute for Health Research, Evaluation, Trials and Studies Coordinating Centre, Alpha House, University of Southampton Science Park, Southampton SO16 7NS, UK.

For peer review only - http://bmjopen.bmj.com/site/about/guidelines.xhtml

NHS
National Institute for Health Research

Nottingham Hearing Biomedical Research Unit

SUMMARY INFORMATION SHEET (S1 Controls v1.1 20.09.13)

Are hearing tests at school accurate and cost effective?

What is the study about?

Most children in the UK have a hearing test when they start school. This study will compare different ways of testing hearing to see which is best.

Why has my child been chosen?

We are inviting 80 children with a hearing loss and 160 children who do not have a hearing loss (like your child) to take part. We want to see if the hearing tests used can correctly identify those with and without a hearing loss.

What will my child have to do?

Your child's hearing will be tested using two hearing tests. Each test will play sounds and your child will need to tell us when they hear them by pressing a button. Each ear will be tested separately. The first test plays the sounds over headphones and the second test plays the sounds from a small machine (like a telephone) held next to your child's ear.

We also need to compare the two hearing tests with a full test of your child's hearing which involves listening to a longer series of sounds.

The research session should take no longer than 45 minutes in total.

When will my child take part?

We will test children at our research unit at Ropewalk House in the centre of Nottingham just once between December 2012 and October 2014.

What are the advantages of taking part?

The study may not help your child, but the information could help to decide how hearing loss should be tested in children in the future.

Are there any risks to taking part?

There should be no risk or discomfort for your child. The loudest sound that they will listen to is approximately the equivalent of a noisy room.

Will my child's information be kept confidential?

All information about your child will be handled in confidence.

Will we receive any payment?

We will pay for any travel expenses and your child will be given a £20 book token to say thank you.

For further information or contact details of the researchers, please read the detailed information sheet included in this pack.

If you would like to support the research, please return the reply slip to us in the prepaid envelope provided.

The diagnostic accuracy of hearing tests and cost-effectiveness of school entry hearing screening programmes
Summary Information Sheet Study 1 Controls Version 1.1 Date: 20.09.13

The National Institute for Health Research Nottingham Hearing Biomedical Research Unit is a partnership between the University of Nottingham, Nottingham University Hospitals NHS Trust and the Medical Research Council (MRC) Institute of Hearing Research

For peer review only - http://bmjopen.bmj.com/site/about/guidelines.xhtml

DOI: 10.3310/hta20360 HEALTH TECHNOLOGY ASSESSMENT 2016 VOL. 20 NO. 36

Children's pictorial information sheet for children recruited as controls and seen in the research facility

Page 1

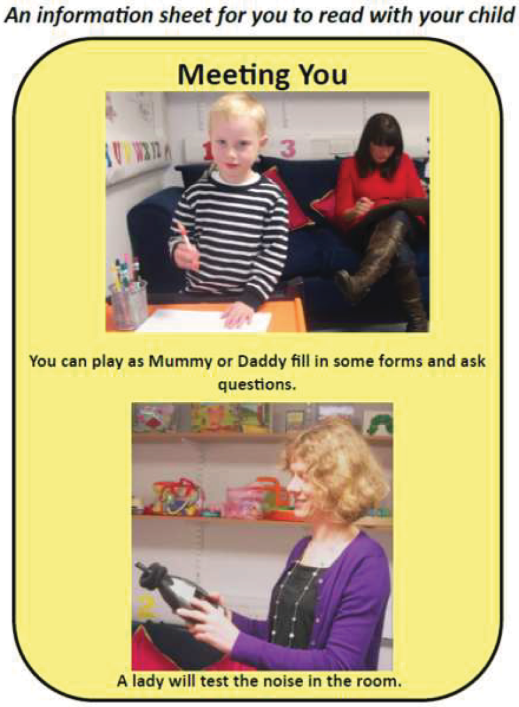

Page 2

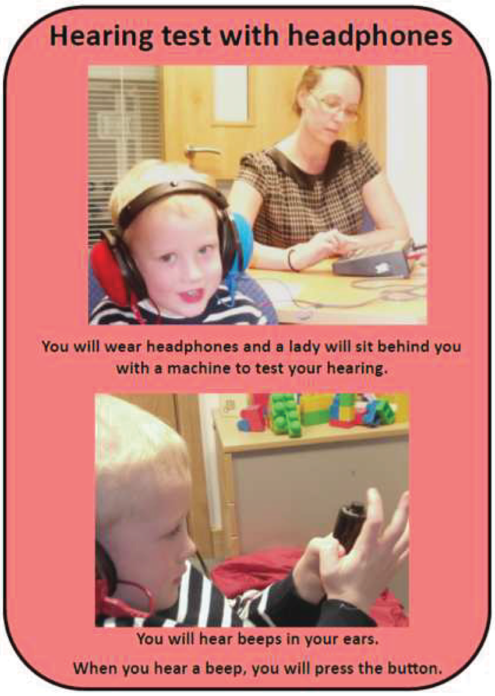

Page 3

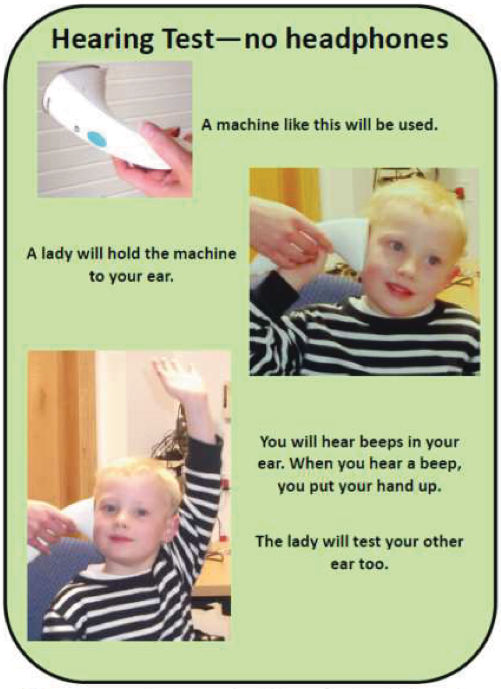

Page 4

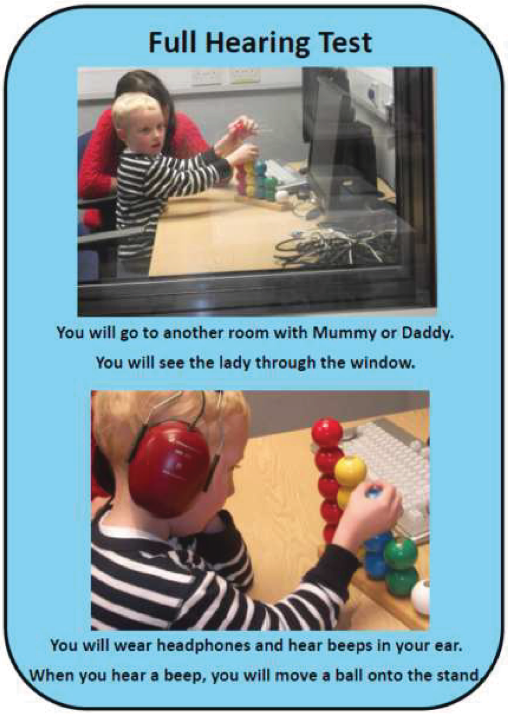

The diagnostic accuracy of hearing tests and cost-effectiveness of school entry hearing screening programmes. Children's information sheet. Study 1 controls v1.1 Date 05.12.12

© Queen's Printer and Controller of HMSO 2016. This work was produced by Fortnum *et al.* under the terms of a commissioning contract issued by the Secretary of State for Health. This issue may be freely reproduced for the purposes of private research and study and extracts (or indeed, the full report) may be included in professional journals provided that suitable acknowledgement is made and the reproduction is not associated with any form of advertising. Applications for commercial reproduction should be addressed to: NIHR Journals Library, National Institute for Health Research, Evaluation, Trials and Studies Coordinating Centre, Alpha House, University of Southampton Science Park, Southampton SO16 7NS, UK.

For peer review only - http://bmjopen.bmj.com/site/about/guidelines.xhtml

For peer review only

DOI: 10.3310/hta20360 HEALTH TECHNOLOGY ASSESSMENT 2016 VOL. 20 NO. 36

Appendix 4 The relationship between the pure-tone screen and HearCheck screener results

TABLE 47 Cross-tabulation of the PTS vs. HC results for ear-level analysis: analyses include only impaired ears that belong to children recruited as cases – impaired ears defined as those with a PTA score of ≥ 30 dB on at least one of the four frequencies

		PTS test results			
		Refer	Pass	Missing	Total
		Impaired (by reference standard)			
HC test results	Refer	104	0	1	105
	Pass	2	1	0	3
	Missing	0	0	0	0
	Total	106	1	1	108
		Hearing (by reference standard)			
HC test results	Refer	34	26	0	60
	Pass	45	340	0	385
	Missing	0	1	0	1
	Total	79	367	0	446
		Missing (no reference standard)			
HC test results	Refer	13	2	1	16
	Pass	3	2	1	6
	Missing	2	0	4	6
	Total	18	4	6	28

For peer review only

© Queen's Printer and Controller of HMSO 2016. This work was produced by Fortnum *et al.* under the terms of a commissioning contract issued by the Secretary of State for Health. This issue may be freely reproduced for the purposes of private research and study and extracts (or indeed, the full report) may be included in professional journals provided that suitable acknowledgement is made and the reproduction is not associated with any form of advertising. Applications for commercial reproduction should be addressed to: NIHR Journals Library, National Institute for Health Research, Evaluation, Trials and Studies Coordinating Centre, Alpha House, University of Southampton Science Park, Southampton SO16 7NS, UK.

For peer review only - http://bmjopen.bmj.com/site/about/guidelines.xhtml

APPENDIX 4

TABLE 48 Cross-tabulation of the PTS vs. HC test results for ear-level analyses: analyses include only impaired ears that belong to children recruited as controls – impaired ears defined as those with a PTA score of ≥ 30 dB on at least one of the four frequencies

		PTS test results			
		Refer	Pass	Missing	Total
		Impaired (by reference standard)			
HC test results	Refer	32	2	0	34
	Pass	8	6	0	14
	Missing	0	0	0	0
	Total	40	8	0	48
		Hearing (by reference standard)			
HC test results	Refer	34	26	0	60
	Pass	45	340	0	385
	Missing	0	1	0	1
	Total	79	367	0	446
		Missing (no reference standard)			
HC test results	Refer	13	2	1	16
	Pass	3	2	1	6
	Missing	2	0	4	6
	Total	18	4	6	28

TABLE 49 Cross-tabulation of the PTS vs. HC test results for ear-level analyses: analyses include all impaired ears regardless of whether belong to children recruited as case or control – impaired ears defined as those with an average PTA score of ≥ 30 dB across the four frequencies

		PTS test results			
		Refer	Pass	Missing	Total
		Impaired (by reference standard)			
HC test results	Refer	109	1	1	111
	Pass	2	4	0	6
	Missing	0	0	0	0
	Total	111	5	1	117
		Hearing (by reference standard)			
HC test results	Refer	61	27	0	88
	Pass	53	343	0	396
	Missing	0	1	0	1
	Total	114	371	0	485
		Missing (no reference standard)			
HC test results	Refer	13	2	1	16
	Pass	3	2	1	6
	Missing	2	0	4	6
	Total	18	4	6	28

For peer review only - http://bmjopen.bmj.com/site/about/guidelines.xhtml

DOI: 10.3310/hta20360 HEALTH TECHNOLOGY ASSESSMENT 2016 VOL. 20 NO. 36

TABLE 50 Cross-tabulation of the PTS vs. HC test results for ear-level analyses: analyses include only impaired ears that belong to children recruited as cases – impaired ears defined as those with an average PTA score of ≥ 30 dB across the four frequencies

		PTS test results			
		Refer	Pass	Missing	Total
		Impaired (by reference standard)			
HC test results	Refer	89	0	1	90
	Pass	1	1	0	2
	Missing	0	0	0	0
	Total	90	1	1	92
		Hearing (by reference standard)			
HC test results	Refer	61	27	0	88
	Pass	53	343	0	396
	Missing	0	1	0	1
	Total	114	371	0	485
		Missing (no reference standard)			
HC test results	Refer	13	2	1	16
	Pass	3	2	1	6
	Missing	2	0	4	6
	Total	18	4	6	28

TABLE 51 Cross-tabulation of the PTS vs. HC test results for ear-level analyses: analyses include only impaired ears that belong to children recruited as controls – impaired ears defined as those with an average PTA score of ≥ 30 dB across the four frequencies

		PTS test results			
		Refer	Pass	Missing	Total
		Impaired (by reference standard)			
HC test results	Refer	20	1	0	21
	Pass	1	3	0	4
	Missing	0	0	0	0
	Total	21	4	0	25
		Hearing (by reference standard)			
HC test results	Refer	61	27	0	88
	Pass	53	343	0	396
	Missing	0	1	0	1
	Total	114	371	0	485
		Missing (no reference standard)			
HC test results	Refer	13	2	1	16
	Pass	3	2	1	6
	Missing	2	0	4	6
	Total	18	4	6	28

© Queen's Printer and Controller of HMSO 2016. This work was produced by Fortnum *et al.* under the terms of a commissioning contract issued by the Secretary of State for Health. This issue may be freely reproduced for the purposes of private research and study and extracts (or indeed, the full report) may be included in professional journals provided that suitable acknowledgement is made and the reproduction is not associated with any form of advertising. Applications for commercial reproduction should be addressed to: NIHR Journals Library, National Institute for Health Research, Evaluation, Trials and Studies Coordinating Centre, Alpha House, University of Southampton Science Park, Southampton SO16 7NS, UK.

For peer review only - http://bmjopen.bmj.com/site/about/guidelines.xhtml

APPENDIX 4

TABLE 52 Cross-tabulation of the PTS vs. HC test results for child-level analyses: analyses include only impaired children who were nominally recruited as cases – impaired children defined as those with an PTA score of ≥ 30 dB on at least one of the four frequencies

		PTS test results			
		Refer	Pass	Missing	Total
		Impaired (by reference standard)			
HC test results	Refer	59	0	0	59
	Pass	0	1	0	1
	Missing	0	0	0	0
	Total	59	1	0	60
		Hearing (by reference standard)			
HC test results	Refer	18	14	0	32
	Pass	22	144	0	166
	Missing	0	0	0	0
	Total	40	158	0	198
		Missing (no reference standard)			
HC test results	Refer	14	0	1	15
	Pass	1	0	0	1
	Missing	1	0	3	4
	Total	16	0	4	20

TABLE 53 Cross-tabulation of the PTS vs. HC test results for child-level analyses: analyses include only impaired children who were nominally recruited as controls – impaired children defined as those with a PTA score of ≥ 30 dB on at least one of the four frequencies

		PTS test results			
		Refer	Pass	Missing	Total
		Impaired (by reference standard)			
HC test results	Refer	26	1	0	27
	Pass	8	2	0	10
	Missing	0	0	0	0
	Total	34	3	0	37
		Hearing (by reference standard)			
HC test results	Refer	18	14	0	32
	Pass	22	144	0	166
	Missing	0	0	0	0
	Total	40	158	0	198
		Missing (no reference standard)			
HC test results	Refer	14	0	1	15
	Pass	1	0	0	1
	Missing	1	0	3	4
	Total	16	0	4	20

For peer review only - http://bmjopen.bmj.com/site/about/guidelines.xhtml

DOI: 10.3310/hta20360 HEALTH TECHNOLOGY ASSESSMENT 2016 VOL. 20 NO. 36

TABLE 54 Cross-tabulation of the PTS vs. HC test results for child-level analyses: analyses include all impaired children regardless of whether recruited as case or control – impaired children defined as those with an average PTA score of ≥ 30 dB across the four frequencies

		PTS test results			
		Refer	Pass	Missing	Total
		Impaired (by reference standard)			
HC test results	Refer	70	0	0	70
	Pass	3	2	0	5
	Missing	0	0	0	0
	Total	73	2	0	75
		Hearing (by reference standard)			
HC test results	Refer	33	15	0	48
	Pass	27	145	0	172
	Missing	0	0	0	0
	Total	60	160	0	220
		Missing (no reference standard)			
HC test results	Refer	14	0	1	15
	Pass	1	0	0	1
	Missing	1	0	3	4
	Total	16	0	4	20

TABLE 55 Cross-tabulation of the PTS vs. HC test results for child-level analyses: analyses include only impaired children who were nominally recruited as cases – impaired ears defined as those with an average PTA score of ≥ 30 dB across the four frequencies

		PTS test results			
		Refer	Pass	Missing	Total
		Impaired (by reference standard)			
HC test results	Refer	53	0	0	53
	Pass	0	1	0	1
	Missing	0	0	0	0
	Total	53	1	0	54
		Hearing (by reference standard)			
HC test results	Refer	33	15	0	48
	Pass	27	145	0	172
	Missing	0	0	0	0
	Total	60	160	0	220
		Missing (no reference standard)			
HC test results	Refer	14	0	1	15
	Pass	1	0	0	1
	Missing	1	0	3	4
	Total	16	0	4	20

© Queen's Printer and Controller of HMSO 2016. This work was produced by Fortnum *et al.* under the terms of a commissioning contract issued by the Secretary of State for Health. This issue may be freely reproduced for the purposes of private research and study and extracts (or indeed, the full report) may be included in professional journals provided that suitable acknowledgement is made and the reproduction is not associated with any form of advertising. Applications for commercial reproduction should be addressed to: NIHR Journals Library, National Institute for Health Research, Evaluation, Trials and Studies Coordinating Centre, Alpha House, University of Southampton Science Park, Southampton SO16 7NS, UK.

For peer review only - http://bmjopen.bmj.com/site/about/guidelines.xhtml

APPENDIX 4

TABLE 56 Cross-tabulation of the PTS vs. HC test results for child-level analyses: analyses include only impaired children who were nominally recruited as controls – impaired ears defined as those with an average PTA score of ≥ 30 dB across the four frequencies

		PTS test results			
		Refer	Pass	Missing	Total
		Impaired (by reference standard)			
HC test results	Refer	17	0	0	17
	Pass	3	1	0	4
	Missing	0	0	0	0
	Total	20	1	0	21
		Hearing (by reference standard)			
HC test results	Refer	33	15	0	48
	Pass	27	145	0	172
	Missing	0	0	0	0
	Total	60	160	0	220
		Missing (no reference standard)			
HC test results	Refer	14	0	1	15
	Pass	1	0	0	1
	Missing	1	0	3	4
	Total	16	0	4	20

For peer review only

For peer review only - http://bmjopen.bmj.com/site/about/guidelines.xhtml

DOI: 10.3310/hta20360 HEALTH TECHNOLOGY ASSESSMENT 2016 VOL. 20 NO. 36

Appendix 5 Parent questionnaire

NHS
National Institute for Health Research

Nottingham Hearing Biomedical Research Unit
Ropewalk House
113 The Ropewalk
Nottingham NG1 5DU

Tel: +44 (0) 115 823 2600
Fax: +44 (0) 115 823 2618
Email: nhbru-enquiries@nottingham.ac.uk
Web: www.hearing.nihr.ac.uk

School Entry Hearing Screening

Questionnaire for parents and carers (version 1.2: 26.09.13)

The University of Nottingham
UNITED KINGDOM · CHINA · MALAYSIA

Participant identifier:
Date returned:

We thank you for completing and returning this questionnaire.

If you would like to be entered into a prize draw for a chance to win a £50 voucher of your choice, please provide us with some contact information on page 5.

The National Institute for Health Research Nottingham Hearing Biomedical Research Unit is a partnership between the University of Nottingham, Nottingham University Hospitals NHS Trust and the Medical Research Council (MRC) Institute of Hearing Research

© Queen's Printer and Controller of HMSO 2016. This work was produced by Fortnum *et al.* under the terms of a commissioning contract issued by the Secretary of State for Health. This issue may be freely reproduced for the purposes of private research and study and extracts (or indeed, the full report) may be included in professional journals provided that suitable acknowledgement is made and the reproduction is not associated with any form of advertising. Applications for commercial reproduction should be addressed to: NIHR Journals Library, National Institute for Health Research, Evaluation, Trials and Studies Coordinating Centre, Alpha House, University of Southampton Science Park, Southampton SO16 7NS, UK.

For peer review only - http://bmjopen.bmj.com/site/about/guidelines.xhtml

The diagnostic accuracy of hearing tests and cost-effectiveness of school entry hearing screening programmes
Study 3 questionnaire – NHBRU V1.2: 26.09.13 NRES ref: 12/WM/0195, Sponsor ref: 12064, Ethics ref: 106333, NIHR HTA10/63/03

A: School Screen

1. Were you aware that your child was having their hearing checked at school?
   Y / N *(please circle one)*

2. How did you find out that your child needed further testing for their hearing? *(please circle one)*

Letter taken home by child / Letter in the post / Telephone / Other, please state ____________

3. When you heard that your child needed further testing for their hearing, how anxious did you feel? Please indicate by circling the appropriate number below:

Not at all anxious ← → Extremely anxious
0 1 2 3 4 5 6 7 8 9 10

4. How many hospital or clinic appointments did your child attend (in total) after being told they needed further testing for their hearing? _______________________

B: Opinion

5. How much do you agree with the following statement; "children should have their hearing checked at school". (*Please circle one)*

   Strongly agree / Agree / No opinion / Disagree / Strongly disagree

6. What are the good things about your child having their hearing checked at school?

____
____
____

7. What are the not so good things about your child having their hearing checked at school?

____
____
____

8. Do you have any further comments?

____
____
____

For peer review only - http://bmjopen.bmj.com/site/about/guidelines.xhtml

DOI: 10.3310/hta20360 HEALTH TECHNOLOGY ASSESSMENT 2016 VOL. 20 NO. 36

C: Impact

9. WHEN COMPLETING THE QUESTIONS BELOW PLEASE CONSIDER THE FIRST FIVE APPOINTMENTS THAT YOUR CHILD MAY HAVE HAD.

**APPOINTMENTS** ➡	1st	2nd	3rd	4th	5th
How long was the journey from home to each appointment? *(please tick)*					
Less than 15 minutes	☐	☐	☐	☐	☐
About 30 minutes	☐	☐	☐	☐	☐
About 1 hour	☐	☐	☐	☐	☐
Between 1 – 2 hours	☐	☐	☐	☐	☐
More than 2 hours	☐	☐	☐	☐	☐
How did you travel to each appointment? *(please tick all that apply)*					
Bus or Tram	☐	☐	☐	☐	☐
Car	☐	☐	☐	☐	☐
Taxi	☐	☐	☐	☐	☐
Train	☐	☐	☐	☐	☐
Other, please state:	________	________	________	________	________
How much did it cost to travel to each appointment? *(please state)*					
Return no. of miles	________	________	________	________	________
Parking (£)	________	________	________	________	________
Tickets/Fares (£)	________	________	________	________	________
Other (£)	________	________	________	________	________
How long were you at each appointment? *(please tick)*					
Less than 30 minutes	☐	☐	☐	☐	☐
Between 30 minutes and 1 hour	☐	☐	☐	☐	☐
About 1 hour	☐	☐	☐	☐	☐
Between 1 – 2 hours	☐	☐	☐	☐	☐
More than 2 hours	☐	☐	☐	☐	☐
How much time was taken off work to attend each appointment? *(please tick)*					
Not working	☐	☐	☐	☐	☐
No time taken off	☐	☐	☐	☐	☐
Part of a day	☐	☐	☐	☐	☐
Full day	☐	☐	☐	☐	☐
How much time did your child have off school to attend each appointment? *(please tick)*					
None	☐	☐	☐	☐	☐
Part of a day	☐	☐	☐	☐	☐

For peer review only

© Queen's Printer and Controller of HMSO 2016. This work was produced by Fortnum *et al.* under the terms of a commissioning contract issued by the Secretary of State for Health. This issue may be freely reproduced for the purposes of private research and study and extracts (or indeed, the full report) may be included in professional journals provided that suitable acknowledgement is made and the reproduction is not associated with any form of advertising. Applications for commercial reproduction should be addressed to: NIHR Journals Library, National Institute for Health Research, Evaluation, Trials and Studies Coordinating Centre, Alpha House, University of Southampton Science Park, Southampton SO16 7NS, UK.

For peer review only - http://bmjopen.bmj.com/site/about/guidelines.xhtml

Full day	☐	☐	☐	☐	☐

10. Did any of the appointments prevent your child from taking part in activities or events that he/she would normally attend? Y / N *(please circle one)*

If yes, please state what. ______________________________

If yes, how many times were these activities or events missed? ______________________

11. Did any of the appointments prevent you from taking part in activities or events that you would normally attend? Y / N *(please circle one)*.

If yes, please state what. ______________________________

If yes, how many times were these activities or events missed? ______________________

12. Did attending appointments cause problems for other members of your family?

Y / N *(please circle one)*. If yes, please state how: ______________________________

---

13. When your child attended hospital or clinic appointments, how anxious did you feel? Please indicate by circling the appropriate number below:

Not at all anxious ← → Extremely anxious

0 1 2 3 4 5 6 7 8 9 10

D: Background Information

14. Your Child's Gender: Male / Female *(please circle one)*

15. Your Child's Ethnicity: *(please tick one)*

White	☐
Mixed / Multiple ethnic groups	☐
Asian / Asian British	☐
Black / African / Caribbean / Black British	☐
Other ethnic group	☐

16. Please provide the name of your child's school: ____________________________

Thank you for taking the time to complete this questionnaire. Your information will remain confidential.
Please return your completed questionnaire in the prepaid envelope provided.

We may conduct a telephone interview to gain some further information about the school hearing screening process from a parent's point of view. While not everyone would be contacted, if you would like to be considered, please supply your details on the next page:

For peer review only - http://bmjopen.bmj.com/site/about/guidelines.xhtml

DOI: 10.3310/hta20360 HEALTH TECHNOLOGY ASSESSMENT 2016 VOL. 20 NO. 36

Please tick any boxes that apply to you:

I would like to be considered for a telephone interview;	Yes ☐	No ☐
I would like to be entered into a prize draw to win a £50 voucher of my choice	Yes ☐	No ☐

Contact Details:

Name: ______________________________ Telephone Number: __________________________

Your contact details will be used if we decide to call you about your responses on the questionnaire or if you win the prize draw.
Your details will not be used for any other reason and they will be destroyed once the study has ended.

© Queen's Printer and Controller of HMSO 2016. This work was produced by Fortnum *et al.* under the terms of a commissioning contract issued by the Secretary of State for Health. This issue may be freely reproduced for the purposes of private research and study and extracts (or indeed, the full report) may be included in professional journals provided that suitable acknowledgement is made and the reproduction is not associated with any form of advertising. Applications for commercial reproduction should be addressed to: NIHR Journals Library, National Institute for Health Research, Evaluation, Trials and Studies Coordinating Centre, Alpha House, University of Southampton Science Park, Southampton SO16 7NS, UK.

For peer review only - http://bmjopen.bmj.com/site/about/guidelines.xhtml

For peer review only

DOI: 10.3310/hta20360 HEALTH TECHNOLOGY ASSESSMENT 2016 VOL. 20 NO. 36

Appendix 6 Practical implementation of screening tests for hearing in schools: information

Participant information sheet

NHS
National Institute for Health Research

Nottingham Hearing
Biomedical Research Unit
Ropewalk House
113 The Ropewalk
Nottingham NG1 5DU

Tel: +44 (0) 115 823 2600
Fax: +44 (0) 115 823 2618
Email: nhbru-enquiries@nottingham.ac.uk
Web: www.hearing.nihr.ac.uk

Information Sheet

Study title: The accuracy of hearing tests and cost-effectiveness of school entry hearing screening programmes

Name of Researcher(s): Dr Heather Fortnum, Ms Sam Catterick and Ms Mara Ozolins

Invitation

We would like to invite your child to take part in a research study. Before you decide whether you agree to their participation it is important for you to understand why the research is being done and what it will involve. If you would like, one of our team will go through the information sheet with you and answer any questions you have. Talk to others about the study if you wish. Ask us if there is anything that is not clear or if you would like more information.

What is the purpose of the study?

Identifying children who have a permanent hearing loss at the earliest possible age is very important. When a hearing loss is detected early, the child's speech and language is usually better and they do better in school. There is now a hearing test at birth for all babies and this means that the vast majority of children born with a hearing loss are identified at birth. However, not all children who will eventually have a hearing loss have that hearing loss at birth.

In most parts of the UK at the moment children have another hearing test when they start school to screen for permanent hearing loss. However, this might not be the best test to use. A new system using a hand-held device to test hearing might be more accurate, and quicker and easier for the children and the school nurses who do the testing.

One aim of this project is to compare the current test with this new way of testing hearing in schools and find out which one is better able to identify hearing loss in children.

© Queen's Printer and Controller of HMSO 2016. This work was produced by Fortnum *et al.* under the terms of a commissioning contract issued by the Secretary of State for Health. This issue may be freely reproduced for the purposes of private research and study and extracts (or indeed, the full report) may be included in professional journals provided that suitable acknowledgement is made and the reproduction is not associated with any form of advertising. Applications for commercial reproduction should be addressed to: NIHR Journals Library, National Institute for Health Research, Evaluation, Trials and Studies Coordinating Centre, Alpha House, University of Southampton Science Park, Southampton SO16 7NS, UK.

For peer review only - http://bmjopen.bmj.com/site/about/guidelines.xhtml

What will happen to my child if they take part?
Your child's hearing will be tested with the system currently used in schools, regardless of whether or not your child takes part in the research. As part of the research we would also like to test your child's hearing with a new handheld device. Each hearing test gives out sounds at levels up to the equivalent of a noisy room and your child will need to say when they hear a sound. Each ear will be tested separately. The first system plays the sounds via headphones and the new system plays the sounds from a small device held next to the child's ear. Your child tells the school nurse when they hear something by raising their hand.

The school nurse will carry out each of the tests in your child's school during the normal school day. A member of the research team will observe the tests and record how long it takes and other general observations about the two tests.

Why has my child been chosen?
Your child is being invited to take part because they have recently started school and are eligible to have a routine hearing test.

Does my child have to take part?
It is up to you to decide whether or not he or she takes part. If you do decide that your child will take part, you don't need to do anything. If you decide that he/she should not take part, please return the reply slip on the invitation letter and return it to the school nurse at your child's school.

What are the possible disadvantages or risks of taking part?
There should be no risk or discomfort for your child. The loudest sound that they will listen to is approximately the equivalent of a noisy room.

What are the advantages of taking part?
We cannot promise the study will help your child but the information we get from this study may help to decide how best to detect hearing loss in children in the future.

What if there is a problem?
If you have a concern about any aspect of this study, you should ask to speak to the researchers who will do their best to answer your questions. The researchers' contact details are given at the end of this information sheet.

Will information about my child be kept confidential?
We will not collect any information about your child in this piece of research, as we are interested only in the methods used to test hearing and not your child's personal details.

What will happen to the results of the study?
The results of the study will be written up into a report for the National Institute for Health Research who are funding the study. We will also publish the results in academic journals and present the results at academic and clinical conferences. The results will feed into government decisions about the best way to screen for hearing loss in children.

Who are the researchers?
The research is being led by Dr Heather Fortnum, an Associate Professor and Reader in Hearing Research at the University of Nottingham. She is working with two research fellows, Sam Catterick and Mara Ozolins who will see the children in this study.

For peer review only - http://bmjopen.bmj.com/site/about/guidelines.xhtml

For peer review only

DOI: 10.3310/hta20360 HEALTH TECHNOLOGY ASSESSMENT 2016 VOL. 20 NO. 36

Who is organising and funding the research?

This research is being organised by the University of Nottingham and is being funded by the National Institute for Health Research, Health Technology Assessment Programme

Who has reviewed this study?

All research in the NHS is looked at by independent group of people, called a Research Ethics Committee, to protect participant's interests. This study has been reviewed and given favourable opinion by West Midlands, Staffordshire Research Ethics Committee.

Further information and contact details

The chief investigator for this study is Dr Heather Fortnum, telephone 0115 8232600 or email heather.fortnum@nottingham.ac.uk. The two researchers working on the study are Sam Catterick, telephone 0115 8232607, email samantha.catterick@nottingham.ac.uk and Mara Ozolins, telephone 0115 8232827, email mara.ozolins@nottingham.ac.uk.

Thank you for reading this

For peer review only

© Queen's Printer and Controller of HMSO 2016. This work was produced by Fortnum *et al.* under the terms of a commissioning contract issued by the Secretary of State for Health. This issue may be freely reproduced for the purposes of private research and study and extracts (or indeed, the full report) may be included in professional journals provided that suitable acknowledgement is made and the reproduction is not associated with any form of advertising. Applications for commercial reproduction should be addressed to: NIHR Journals Library, National Institute for Health Research, Evaluation, Trials and Studies Coordinating Centre, Alpha House, University of Southampton Science Park, Southampton SO16 7NS, UK.

For peer review only - http://bmjopen.bmj.com/site/about/guidelines.xhtml

Case record form

The diagnostic accuracy of hearing tests and cost-effectiveness of school entry hearing screening programmes

CRF: SES Study 4

SCHOOL DETAILS

Name of school

Postcode

Researcher: MO or SC?

No. of pupils on roll

No. of pupils receiving free school meals

Location Urban Rural

SESSION DETAILS

Date of Observation d d / m m / y y y y

Start time h h : m m

End time h h : m m

No. of Hearcheck disposable cups used during the session

Name of Nurse

Is a member of school staff present? Yes No

If yes, state job role

FEEDBACK FROM NURSE AFTER THE SESSION

PURE TONE SCREEN (PTS)

On a scale of 0-10 (with 0 being low), how would you rate the PTS on ease of use?

On a scale of 0-10 (with 0 being low), how would you rate the PTS on accuracy?

On a scale of 0-10 (with 0 being low), how much would you want to keep the PTS in the future?

Additional comments on the PTS:

HEARCHECK (HC)

On a scale of 0-10 (with 0 being low), how would you rate the HC on ease of use?

On a scale of 0-10 (with 0 being low), how would you rate the HC on accuracy?

On a scale of 0-10 (with 0 being low), how much would you want to keep the HC in the future?

Additional comments on the HC:

For peer review only - http://bmjopen.bmj.com/site/about/guidelines.xhtml

DOI: 10.3310/hta20360 HEALTH TECHNOLOGY ASSESSMENT 2016 VOL. 20 NO. 36

INFORMATION ABOUT THE CIRCUMSTANCES OF THE SCREEN

Make comments on items such as; Where did the screening take place? How many children were present? How were explanations done? Were there any interruptions? Any other details that affected the screening.

For peer review only

© Queen's Printer and Controller of HMSO 2016. This work was produced by Fortnum *et al.* under the terms of a commissioning contract issued by the Secretary of State for Health. This issue may be freely reproduced for the purposes of private research and study and extracts (or indeed, the full report) may be included in professional journals provided that suitable acknowledgement is made and the reproduction is not associated with any form of advertising. Applications for commercial reproduction should be addressed to: NIHR Journals Library, National Institute for Health Research, Evaluation, Trials and Studies Coordinating Centre, Alpha House, University of Southampton Science Park, Southampton SO16 7NS, UK.

For peer review only - http://bmjopen.bmj.com/site/about/guidelines.xhtml

APPENDIX 6

Child ID	Test order Enter order number next to each test or ND if not done				Which hearing screen done first? PTS or HC	PURE TONE SCREEN		HEARCHECK		Comments about individual child/screen
						Time taken for screen mins: secs	Pass / Fail / ND / Incomplete?	Time taken for screen mins: secs	Pass / Fail / ND / Incomplete?	
	Vision		Hearing		PTS / HC		Pass / Fail / ND / Incomplete		Pass / Fail / ND / Incomplete	
	Height		Weight							
	Vision		Hearing		PTS / HC		Pass / Fail / ND / Incomplete		Pass / Fail / ND / Incomplete	
	Height		Weight							
	Vision		Hearing		PTS / HC		Pass / Fail / ND / Incomplete		Pass / Fail / ND / Incomplete	
	Height		Weight							
	Vision		Hearing		PTS / HC		Pass / Fail / ND / Incomplete		Pass / Fail / ND / Incomplete	
	Height		Weight							
	Vision		Hearing		PTS / HC		Pass / Fail / ND / Incomplete		Pass / Fail / ND / Incomplete	
	Height		Weight							
	Vision		Hearing		PTS / HC		Pass / Fail / ND / Incomplete		Pass / Fail / ND / Incomplete	
	Height		Weight							
	Vision		Hearing		PTS / HC		Pass / Fail / ND / Incomplete		Pass / Fail / ND / Incomplete	
	Height		Weight							
	Vision		Hearing		PTS / HC		Pass / Fail / ND / Incomplete		Pass / Fail / ND / Incomplete	
	Height		Weight							
	Vision		Hearing		PTS / HC		Pass / Fail / ND / Incomplete		Pass / Fail / ND / Incomplete	
	Height		Weight							
	Vision		Hearing		PTS / HC		Pass / Fail / ND / Incomplete		Pass / Fail / ND / Incomplete	
	Height		Weight							
	Vision		Hearing		PTS / HC		Pass / Fail / ND / Incomplete		Pass / Fail / ND / Incomplete	
	Height		Weight							
	Vision		Hearing		PTS / HC		Pass / Fail / ND / Incomplete		Pass / Fail / ND / Incomplete	
	Height		Weight							

PTS = Pure Tone Screen **HC = HearCheck**

SES study 4 - CRF v 2 28.01.14

NRES ref: 12/WM/0195, Sponsor ref: 12064, Ethics ref: 106333, NIHR HTA10/63/03

3 of 4

For peer review only

For peer review only - http://bmjopen.bmj.com/site/about/guidelines.xhtml

DOI: 10.3310/hta20360 HEALTH TECHNOLOGY ASSESSMENT 2016 VOL. 20 NO. 36

Child ID	Test order<br>Enter order number next to each test or ND if not done				Which hearing screen done first?<br><br>PTS or HC	PURE TONE SCREEN<br>Time taken for screen<br>mins: secs	PURE TONE SCREEN<br>Pass / Fail / ND / Incomplete?	HEARCHECK<br>Time taken for screen<br>mins: secs	HEARCHECK<br>Pass / Fail / ND / Incomplete?	Comments about individual child/screen
	Vision		Hearing		PTS / HC		Pass / Fail / ND / Incomplete		Pass / Fail / ND / Incomplete	
	Height		Weight							
	Vision		Hearing		PTS / HC		Pass / Fail / ND / Incomplete		Pass / Fail / ND / Incomplete	
	Height		Weight							
	Vision		Hearing		PTS / HC		Pass / Fail / ND / Incomplete		Pass / Fail / ND / Incomplete	
	Height		Weight							
	Vision		Hearing		PTS / HC		Pass / Fail / ND / Incomplete		Pass / Fail / ND / Incomplete	
	Height		Weight							
	Vision		Hearing		PTS / HC		Pass / Fail / ND / Incomplete		Pass / Fail / ND / Incomplete	
	Height		Weight							
	Vision		Hearing		PTS / HC		Pass / Fail / ND / Incomplete		Pass / Fail / ND / Incomplete	
	Height		Weight							
	Vision		Hearing		PTS / HC		Pass / Fail / ND / Incomplete		Pass / Fail / ND / Incomplete	
	Height		Weight							
	Vision		Hearing		PTS / HC		Pass / Fail / ND / Incomplete		Pass / Fail / ND / Incomplete	
	Height		Weight							
	Vision		Hearing		PTS / HC		Pass / Fail / ND / Incomplete		Pass / Fail / ND / Incomplete	
	Height		Weight							
	Vision		Hearing		PTS / HC		Pass / Fail / ND / Incomplete		Pass / Fail / ND / Incomplete	
	Height		Weight							
	Vision		Hearing		PTS / HC		Pass / Fail / ND / Incomplete		Pass / Fail / ND / Incomplete	
	Height		Weight							
	Vision		Hearing		PTS / HC		Pass / Fail / ND / Incomplete		Pass / Fail / ND / Incomplete	
	Height		Weight							

PTS = Pure Tone Screen **HC = HearCheck**

SES study 4 - CRF v 2 28.01.14

NRES ref: 12/WM/0195, Sponsor ref: 12064, Ethics ref: 106333, NIHR HTA10/63/03

© Queen's Printer and Controller of HMSO 2016. This work was produced by Fortnum *et al.* under the terms of a commissioning contract issued by the Secretary of State for Health. This issue may be freely reproduced for the purposes of private research and study and extracts (or indeed, the full report) may be included in professional journals provided that suitable acknowledgement is made and the reproduction is not associated with any form of advertising. Applications for commercial reproduction should be addressed to: NIHR Journals Library, National Institute for Health Research, Evaluation, Trials and Studies Coordinating Centre, Alpha House, University of Southampton Science Park, Southampton SO16 7NS, UK.

For peer review only

For peer review only - http://bmjopen.bmj.com/site/about/guidelines.xhtml

For peer review only

For peer review only

EME
HS&DR
HTA
PGfAR
PHR

Part of the NIHR Journals Library
www.journalslibrary.nihr.ac.uk

This report presents independent research funded by the National Institute for Health Research (NIHR). The views expressed are those of the author(s) and not necessarily those of the NHS, the NIHR or the Department of Health

Published by the NIHR Journals Library

For peer review only

For peer review only - http://bmjopen.bmj.com/site/about/guidelines.xhtml

BMJ Open

A directly comparative two-gate case-control diagnostic accuracy study of the pure tone screen and HearCheck Screener tests for identifying hearing impairment in school children

Journal:	*BMJ Open*
Manuscript ID	bmjopen-2017-017258.R1
Article Type:	Research
Date Submitted by the Author:	21-May-2017
Complete List of Authors:	Ukoumunne, Obioha; University of Exeter Medical School, NIHR CLAHRC South West Peninsula (PenCLAHRC)<br>Hyde, Chris; University of Exeter, Exeter Test Group<br>Ozolins, Mara; Nottingham University, NIHR Nottingham Biomedical Research Centre<br>Zhelev, Zhivko; University of Exeter, University of Exeter Medical School<br>Errington, Sam; University of Nottingham, NIHR Nottingham Hearing Biomedical Research Unit<br>Taylor, Rod; University of Exeter, Peninsula Medical School<br>Benton, Claire; Nottingham University Hospitals NHS Trust, Nottingham Audiology Services<br>Moody, Joanne; Ida Darwin Hospital, Community Child Health<br>Cocking, Laura; Plymouth University, Peninsula Clinical Trials Unit at Plymouth University (PenCTU)<br>Watson, Julian; Public Involvement Representative<br>Fortnum, Heather; University of Nottingham, NIHR Nottingham Biomedical Research Centre
**Primary Subject Heading**:	Ear, nose and throat/otolaryngology
Secondary Subject Heading:	Communication, Ear, nose and throat/otolaryngology, Paediatrics
Keywords:	school entry hearing screen, diagnostic accuracy, pure tone screen, HearCheck Screener, pure tone audiogram, hearing impairment

SCHOLARONE™ Manuscripts

A directly comparative two-gate case-control diagnostic accuracy study of the pure tone screen and HearCheck Screener tests for identifying hearing impairment in school children

Author names and institutions

Obioha C Ukoumunne[1], Associate Professor, Medical Statistics

Chris Hyde[2], Professor, Public Health and Clinical Epidemiology

Mara Ozolins[3], Research Fellow, Hearing Research

Zhivko Zhelev[1], Research Fellow, Systematic Reviews

Sam Errington[3], Research Associate, Hearing Research

Rod S Taylor[2], Professor, Health Services Research

Claire Benton[4], Clinical Scientist (Audiology)

Joanne Moody[5], Clinical Scientist (Audiology)

Laura Cocking[6], Senior Data Manager

Julian Watson[7], Public Involvement Representative

Heather Fortnum[3], Associate Professor and Reader, Hearing Research

[1] NIHR CLAHRC South West Peninsula (PenCLAHRC), University of Exeter Medical School, South Cloisters, St Luke's Campus, Heavitree Road, Exeter, EX1 2LU, UK

[2] Institute of Health Research, University of Exeter Medical School, South Cloisters, St Luke's Campus, Heavitree Road, Exeter, EX1 2LU, UK

[3] NIHR Nottingham Biomedical Research Centre, University of Nottingham, Ropewalk House, 113 The Ropewalk, Nottingham, NG1 5DU, UK

[4] Nottingham Audiology Services, Nottingham University Hospitals, Ropewalk House, 113 The Ropewalk, Nottingham, NG1 5DU, UK

[5] Community Child Health, Ida Darwin Hospital, Fulbourn, Cambridge, CB21 5EE, UK

[6] Peninsula Clinical Trials Unit at Plymouth University (PenCTU), Plymouth University, ITTC Building 1, Plymouth Science Park, 1 Davy Road, Plymouth, PL6 8BX

[7] Public Involvement Representative

For peer review only

Corresponding author

Dr Obioha C Ukoumunne

Associate Professor in Medical Statistics

NIHR CLAHRC South West Peninsula

University of Exeter Medical School

South Cloisters

St Luke's Campus

Heavitree Road

Exeter EX1 2LU

UK

O.C.Ukoumunne@exeter.ac.uk

Word count (abstract): 300

Word count (main text): 3424

Number of tables: 4

Number of figures: 4

Key words: school entry hearing screen, diagnostic accuracy, pure tone screen, HearCheck Screener, pure tone audiogram, hearing impairment

For peer review only

For peer review only - http://bmjopen.bmj.com/site/about/guidelines.xhtml

Abstract

Objectives: This study directly compared the accuracy of two audiometry-based tests for screening school children for hearing impairment: the currently used test, pure tone screen, and a device newly applied to children, HearCheck Screener.

Design: Two test two-gate case-control diagnostic test accuracy study.

Setting and Participants: Hearing impaired children ("intended cases") aged 4-6 years were recruited between February 2013 and August 2014 from collaborating audiology services. Children with no previously identified impairment ("intended controls") were recruited from Foundation and Year 1 of schools between February 2013 and June 2014 in central England. The reference standard was pure tone audiometry. Tests were administered at Nottingham Hearing Biomedical Research Unit (NHBRU) or, for some intended cases only, in the participant's home.

Main outcome measures: Sensitivity and specificity of the pure tone screen and HearCheck tests based on pure tone audiometry result as reference standard.

Results: 315 children (630 ears) were recruited; 75 from audiology services and 240 from schools. Full test and reference standard data were obtained for 600 ears; 155 ears were classified as truly impaired and 445 as truly hearing based on the pure tone audiometry assessment. Sensitivity was estimated to be 94.2% (95% CI: 89.0% to 97.0%) for pure tone screen and 89.0% (95% CI: 82.9% to 93.1%) for HearCheck (difference = 5.2% favouring pure tone screen; 95% CI: 0.2% to 10.1%; p=0.02). Estimates for specificity were 82.2% (95% CI: 77.7% to 86.0%) for pure tone screen and 86.5% (95% CI: 82.5% to 89.8%) for HearCheck (difference = 4.3% favouring HearCheck; 95% CI: 0.4% to 8.2%; p = 0.02).

Conclusion: Pure tone screen was better than HearCheck with respect to sensitivity, but inferior with respect to specificity. As avoiding missed cases is arguably of greater importance for school entry screening, pure tone screen is probably preferable in this context.

Study registration: Current Controlled Trials: ISRCTN61668996

For Peer review only

For peer review only - http://bmjopen.bmj.com/site/about/guidelines.xhtml

Strengths and limitations of the study

- A public involvement representative was a full member of the study team and contributed to the development, conduct and interpretation of the study.
- The audiometry-based screening tests, pure tone screen and HearCheck Screener, were directly compared in the same sample of children.
- The two-gate case-control study design used to identify cases and controls is known to be susceptible to bias.
- The estimates of accuracy of each test might be biased but estimates of comparative accuracy are unlikely to be affected by the design.

For peer review only

Introduction

Identification of permanent hearing impairment at the earliest possible age is crucial to the development of speech and language and for ensuring the best opportunities for educational achievement and quality of life (1). The highly sensitive and specific universal new-born hearing screen (UNHS) identifies the vast majority of children born with a hearing impairment (2). Due to acquisition, progression or late-onset of hearing impairment and geographical movement of families, however, a significant number of children remain to be identified with a permanent hearing impairment after the new-born period. The school entry screen (SES), a universal hearing screen when children start school, was established in 1955 and remains in place in many parts of the UK. It is considered a safeguard screen to identify hearing impairment.

The 2007 NIHR Health Technology Assessment (HTA)-funded evaluation of the cost-effectiveness of the school entry hearing screen in the UK (3) included a survey of practice which found that the audiometry-based pure tone screen (PTS) test (4) was used in all cases. The diagnostic accuracy studies identified by a review that was part of this evaluation found PTS to generally have higher sensitivity for minimal, mild and greater hearing impairments than alternative tests (tympanometry, otoscopy, transient-evoked otoacoustic emission tests, parent questionnaires, spoken word tests) for which evidence was identified (3). These comparisons were, however, indirect and highly susceptible to confounding. Furthermore, most of these accuracy studies were undertaken in populations where the prevalence of undetected hearing impairment was considerably greater than that likely to be encountered in a system where a universal new-born hearing screening programme is in place. The estimates of accuracy were also based on small sample sizes. A relatively new device, HearCheck Screener (HC) (5), also audiometry-based, came onto the market in 2005 as a tool for screening for hearing impairment in adults in a general practice setting. It is less comprehensive and flexible than PTS but has the potential to be a quicker test in the school setting. It has not previously been assessed as a tool for screening children in the UK. For further details on how HearCheck Screener is used, refer to: http://www.connevans.co.uk/product/2831233/38SHEARCHECK/Siemens-HearCheck-Screener [accessed 18th May 2017]

The objective of this study was to compare the diagnostic accuracy of PTS and HC tests for hearing impairment of any type at or around school entry using full pure tone audiometry

For peer review only - http://bmjopen.bmj.com/site/about/guidelines.xhtml

(PTA) as the reference standard (6). The study was part of a HTA-funded programme of work with the wider aim of assessing the effectiveness and cost-effectiveness of the school entry hearing screen (7). The full study protocol is available from the authors on request.

Methods

Participants

This diagnostic test accuracy study used a directly comparative two-gate case-control design (8).

Intended cases

Hearing impaired children aged 4-6 years between February 2013 and August 2014 were identified by collaborating audiology services (centres) in central England. They had permanent sensorineural or conductive hearing impairment averaged across the four frequencies 0.5, 1, 2 and 4kHz, either bilaterally (average of 20-60dB HL) or unilaterally (any level ≥20dB HL). Children were identified by the paediatric audiologist in each centre. The reference standard was pure tone audiometry (PTA), and potential recruits were excluded if there was no record of a PTA in the previous 12 months or planned for the following three months, and the family was unwilling to travel to their local service or to Nottingham to undergo the assessment. Eligible children for whom parents provided agreement to take part were invited to undergo the two screening tests (PTS and HC), either in their own homes, or at Nottingham Hearing Biomedical Research Unit (NHBRU), depending on their preference.

Intended controls

Children with no previously identified hearing impairment were recruited from the Foundation Year and Year 1 of schools in the Nottingham area (central England), between February 2013 and June 2014. The study researchers provided an agreed letter of invitation and information packs for the school. Children for whom agreement to take part was provided were invited to undergo the two screening tests and the PTA reference standard assessment at NHBRU.

For peer review only - http://bmjopen.bmj.com/site/about/guidelines.xhtml

Procedures

Written informed consent was obtained from the parent or legal guardian before the child entered the study. Test data for all children and reference standard data for all intended controls were collected specifically for this study. For most of the intended cases the reference standard data were based on previous assessments otherwise unconnected to the study.

Pure tone screen test

Headphones were placed over the child's ears and then pure tones presented across the key frequencies for speech understanding in the order 1kHz, 2kHz, 4kHz and 0.5kHz. Each tone was held for 2 to 3 seconds, with staggered pauses. All four frequencies were tested in one ear before being tested in the other. To pass the screen in a given ear the child needed to respond to 2 out of 3 presentations of each frequency at 20dB HL to pass. The researcher was positioned to ensure they had a clear view of the child without giving any visual cues throughout the test. The child was instructed to place a ball onto a frame every time they heard a sound, however quiet. Hearing aids, glasses, hairbands and earrings were removed where relevant. A familiarisation tone (1kHz at 60dB HL) was presented to ensure the child had understood the instructions.

HearCheck Screener test

The HC screener was placed over the child's ear and an automatic sequence of pure tones played at three levels at each of the frequencies 1 kHz (55dB, 35dB and 20dB) and 3 kHz (75dB, 55dB, 35dB). To pass the screen in a given ear the child needed to respond to all six tones. The child indicated, usually by raising their hand, that they heard each tone. The child was asked to remove hearing aids, and also glasses and earrings if necessary for a good fit. A disposable cardboard ear cover was put onto the HC for each ear. The HC was held against the first ear to be tested, often holding the child's head still with the free hand. The button was pressed and the first three tones were allowed to play for the first frequency. The button was then pressed again for the remaining three tones for the second frequency. The procedure was repeated on the other ear.

For peer review only - http://bmjopen.bmj.com/site/about/guidelines.xhtml

Reference standard

After intended cases had their appointment, the researcher phoned the audiologist, asking them to post their most recent PTA results to NHBRU. For intended controls PTA was carried out in NHBRU at the same session as the screening tests, using the audiometer in a sound-proofed booth with the child sat facing away from the equipment. PTA testing followed standard British Society of Audiology (BSA) recommended procedure (6) without otoscopic examination or masking, for air conduction only. Hearing impairment was considered present when the PTA reference standard threshold was ≥30dB on at least one of the four frequencies (0.5 kHz, 1 kHz, 2 kHz and 4 kHz) and considered absent when the reference threshold was <30dB on all four frequencies.

Other procedural details

Equipment was calibrated as per manufacturer's instructions. There was generally less background noise than would be expected in schools. The researchers were trained in administering the PTA and PTS by the audiologists in the Children's Hearing Assessment Centre in Nottingham, using a mixture of observation, practice on children and feedback.

The order of administering the two screening tests and which researcher undertook them was determined randomly. For intended cases one researcher performed the PTS and another researcher performed the HC. For intended controls one researcher carried out both the screening tests and then another researcher performed the PTA measurement. We sought to blind the second researcher to the results of the first test(s) by asking them to leave the room. The PTA result obtained from the audiologist for intended cases was examined only after the results of the screening tests were known.

Ethical approval was granted by the West Midlands, Staffordshire Research Ethics Committee (Ref: 106333).

Statistical analysis

The target sample size was 80 hearing impaired children and 160 children with no hearing impairment. Eighty impaired children is large enough to estimate a sensitivity of 80% with a margin of error of 10.4% based on the lower bound of the 95% confidence interval and 160 children without impairment is large enough to estimate a specificity of 80% with a margin of error of 7.0%. Accuracy was evaluated using the ear as the unit of analysis. In the main

For peer review only - http://bmjopen.bmj.com/site/about/guidelines.xhtml

analysis, irrespective of intended case or control status, ears were defined as truly hearing impaired or not based on actual PTA reference standard results. Analyses were carried out using Stata statistical software (version 13.1). We reported the absolute difference in percentages between the PTS and HC for each of sensitivity and specificity with 95% confidence intervals and McNemar's test p-value (using the Stata command `mcc`). We used analytical methods that recognise the correlation between results of ears belonging to the same child. Details of further exploratory analyses are provided in the Appendix.

Public involvement

The research question originates from a call from the NIHR HTA funding stream to evaluate the diagnostic accuracy of hearing tests and cost-effectiveness of school entry hearing screening programmes. We recruited Julian Watson, a parent of a child who has experienced conductive hearing impairment, to be a full member of the study team and an author on this paper. His input included comments on information literature for participating parents; development of methodology and the conduct of the study (e.g., addressing recruitment challenges); attending study meetings; and critical comments and suggestions on the final study report and this paper. We also included on our study steering committee a representative from the National Deaf Children's Society. Parents of participating children were offered the opportunity to receive a lay summary of the findings at the end of the study. Almost all parents took up the offer and it was sent to them.

Results

Participants

Intended cases were recruited from 14 audiology services. We received 86 replies from 379 invitations sent by the audiologists. Eight children were ineligible, being outside the required age range. We were unable to contact one of the initial respondents, and we were unable to see a further two children due to researcher illness just before the close of recruitment. We recruited and tested the remaining 75 children (19.8% of those invited) (**Figure 1**). [Figure 1 here]

Intended controls were recruited from 51 of the 164 schools in the Nottingham area that were invited by post to take part. The 51 schools between them gave information packs to the parents of 2787 children, of whom 291 (10.4%) replied, confirming they would like to participate. An additional 11 siblings of children who attended the appointment but who did

For peer review only - http://bmjopen.bmj.com/site/about/guidelines.xhtml

not receive the invitation were in the correct age range and parents agreed for them to take part. Eight of the 302 invited children were subsequently found to be ineligible for the study (one was too old, six already had hearing problems identified, one replied after recruitment closed), 11 changed their minds about taking part, and we were unable to see 43 either because we could not make an appointment (mostly not contactable) or they did not attend the arranged appointment. The remaining 240 children were recruited as intended controls and seen for study appointments (**Figure 2**). [Figure 2 here]

Table 1 summarises the demographic characteristics of participating children by whether they were recruited via audiology services (intended cases) or via schools (intended controls). The groups were similar with respect to gender and age.

Intended controls completed the tests and reference standard on the same day. For intended cases, reference standard data were already available prior to the tests being administered for 65 children and for the remaining 10 children a reference standard assessment took place after administering the PTS and HC. The median time interval between reference standard and test assessment was 16 weeks. There were no adverse events from performing the tests and the reference standard.

Number of ears with impaired or non-impaired hearing

Of the 630 recruited ears, 600 (95.2%) provided full data on the PTS and HC tests and scores for all four frequencies of the PTA reference standard and were included in the main analyses. Two hundred and ninety five children provided full data on both ears and another 10 provided full data on just one ear. There were no indeterminate screening test or PTA results. The PTA reference standard categorised 155 ears as impaired and 445 as not impaired. The mean (SD) hearing level in dB at frequencies 0.5 kHz, 1 kHz, 2 kHz and 4 kHz, was 43.1 (21.0), 45.0 (22.5), 46.2 (25.0) and 49.0 (24.2), respectively, for impaired ears and 9.4 (7.4), 4.7 (7.5), 3.7 (6.6) and 4.9 (8.1), respectively, for hearing ears. One hundred and seven of the impaired ears belonged to children recruited from audiology services and the remaining 48 ears belonging to children with no previously identified hearing loss.

Figures 3 and 4 present flowcharts that describe the number of impaired ears (based on PTA ≥30dB on at least one of the 4 frequencies) and hearing ears that passed and referred on the PTS and HC tests, respectively. [Figure 3 here] [Figure 4 here]

For peer review only - http://bmjopen.bmj.com/site/about/guidelines.xhtml

Sensitivity and specificity

Table 2 summarises the relationship between the PTS and HC test results separately for impaired ears (first panel), hearing ears (second panel) and ears for which information on the reference standard was missing (third panel). The figures highlighted in grey in the first panel indicate the 155 impaired ears that were used in the calculation of sensitivity. The 445 hearing ears used in the calculation of specificity are highlighted in the second panel.

Table 3 reports the sensitivity and specificity of the screening tests. The sensitivity was 94.2% for PTS and 89.0% for HC. The 95% confidence interval for sensitivity indicates that we can be fairly certain that the true sensitivity is no lower than 89% for PTS and 83% for HC. The McNemar's test result (p = 0.02) indicates evidence that the true sensitivity is greater for PTS than for HC. The estimates of specificity were 82.2% for PTS and 86.5% for HC, with evidence provided by McNemar's test that the true specificity is higher for HC than PTS (p = 0.02).

False negatives

The mean hearing level across the 4 test frequencies on the PTA reference standard for the 19 ears that passed one or both of the screening tests but referred by the PTA was 28 (SD = 9) dB compared to 48 (SD = 21) dB for the remaining 136 impaired ears that referred on both PTS and HC. This indicates that impairment was less severe for the false negatives than the true positives.

Discussion

The main finding of our study is that PTS was better than HC with respect to sensitivity (5.2% in favour of PTS; 95% CI: 0.2%, 10.1%; p=0.02), but inferior with respect to specificity (4.3% in favour of HC; 95% CI: 0.4% to 8.2%; p = 0.02).

The two-gate diagnostic test accuracy study design employed is widely acknowledged to be open to bias in the assessment of accuracy (8). However, given the extremely low prevalence of hearing impairment in a school entry population, approximately 0.5% (7), this was felt to be the only feasible design. In a traditional accuracy study where the test and reference standard are administered to all participants identified from a single source ("single-gate") with no advance knowledge of their true disease status (8), 16,000 school children in the UK

For peer review only - http://bmjopen.bmj.com/site/about/guidelines.xhtml

would need to have been recruited to identify our target of 80 cases of hearing impairment and so offer the same precision for measuring sensitivity. The bias might lead to an overestimate of accuracy for each test individually, although we believe that it might have less impact on comparison of accuracy as both tests would be subject to any overestimation. Measuring PTS and HC accuracy in the near to ideal conditions in this study as opposed to the nosier circumstances that would prevail in schools is also likely to lead to inflation of accuracy of the tests individually.

We remain confident that there are no other studies that directly compare PTS with HC. Indeed there are very few directly comparative accuracy studies of any of the potential screening tests for hearing impairment (7). Our findings are consistent with indirect comparison of PTS with other tests which suggest that PTS is superior (3, 7). What this study adds is that when PTS is used in a standard manner and HC is used in the manner designed by the manufacturers, there is a trade-off between sensitivity and specificity and a threshold effect may be part of the apparent difference between the two tests. However, given that thresholds are fixed, particularly for HC, it is reasonable to consider which of PTS and HC in the conventional forms used in the study would be preferable in practice. Some further insight into this is given by reflecting on the absolute numbers of false positives and false negatives when the differences in accuracy are applied to a population with a prevalence of hearing impairment similar to one which might be observed in practice. This is done in **Table 4** where the accuracy estimates are applied to a population of 10,000 with a prevalence of hearing impairment of 0.5% (i.e., 50 with impairment). In most tests used for screening and triage, there is a preference for avoiding false negatives, because it may take many years for "missed" individuals to re-engage with the health system, by which time the opportunity to successfully intervene may have been lost. However, as **Table 4** shows the number of false positives (1771 and 1343 for PTS and HC, respectively) is so much larger than the number of false negatives (3 and 5 for PTS and HC, respectively), that it is reasonable to question whether the cumulative added costs of unnecessary testing in false positives have reached a point where they outweigh the cumulative benefits of avoiding a much smaller number of false negatives. This is particularly true where the nature of the hearing impairment is milder in the missed cases than in those who correctly tested positive, as we found in this study. We did, however, note in another component study of this programme of work that the number of screened children attending for diagnostic evaluation was much less than would be implied by test specificity, suggesting strongly that the number of false positives in a screening

For peer review only - http://bmjopen.bmj.com/site/about/guidelines.xhtml

programme is much less than would be indicated by test specificity in isolation (7). This is because in a screening programme, those initially testing as impaired may have their screening result rechecked or reviewed before being finally sent for diagnostic evaluation. So the impact of false positives is overstated if one relies on test specificity in isolation rather than considering the specificity of the programme as a whole.

On balance, therefore, we retain the view that the reduced number of false negatives associated with PTS use (2 fewer per 10,000 children screened – **Table 4**) does outweigh the advantage in terms of test specificity apparently offered by HC which has 428 fewer false positives per 10,000 screened. The implications for practice are thus that where school entry hearing screening is still being used or is under consideration, PTS would be the better screening tool. We do note, however, that recently concerns have been expressed about the likely cost-effectiveness of SES relative to a system reliant on ad hoc identification of possible hearing impairment and referral for diagnostic evaluation, although this is an early finding needing confirmation (7).

In terms of implications for research, whilst we note that this study gives robust information about the choice between PTS and HC, there are other alternative tests such as automated audiometry-based hearing screening systems installed on laptops or hand-held devices (9-14), otoacoustic emissions (OAEs) (15-19) and Automated Auditory Brainstem Response (AABR) (20). Although they have been the object of direct comparison of accuracy, further research is necessary to provide more robust evidence of their comparative performance, feasibility and cost-effectiveness in different country-specific contexts. Furthermore, we would suggest that if the arguments for the validity of comparative two-gate accuracy studies as used here are accepted this would be an appropriate and efficient means to evaluate relative accuracy in the future. Incorporating such direct comparisons into on-going systematic reviews of single test accuracy studies should also be anticipated. Finally, the work we have done here on accuracy of the hearing screening tests should be extended to estimate the accuracy of the school entry hearing screening programme itself.

For peer review only - http://bmjopen.bmj.com/site/about/guidelines.xhtml

References

1. Yoshinaga-Itano C., Coulter D., Thomson V. The Colorado Newborn Hearing Screening Project: effects on speech and language development for children with hearing loss. J Perinatol. 2000;20:S132–7.

2. Kennedy C., McCann D., Campbell M.J., Kimm L., Thornton R. Universal newborn screening for permanent childhood hearing impairment: an 8-year follow-up of a controlled trial. Lancet. 2005;336:660-2.

3. Bamford J., Fortnum H., Bristow K., Smith J., Vamvakas G., Davies L., et al. Current practice, accuracy, effectiveness and cost-effectiveness of the school entry hearing screen. Health Technol Assess. 2007;11(32).

4. Ewing A. The sweep frequency method of making screening tests of the hearing of school children. BMJ. 1955;4904:41–2.

5. Siemens. HearCheck Screener Userguide. 2012.

6. British Society of Audiology. Pure-tone air-conduction and bone-conduction threshold audiometry with and without masking. 2012.

7. Fortnum H., Ukoumunne O.C., Hyde C., Taylor R.S., Ozolins M., Errington S., et al. A programme of studies including assessment of diagnostic accuracy of school hearing screening tests and a cost-effectiveness model of school entry hearing screening programmes. Health Technol Assess. 2016;20(36).

8. Rutjes A.W.S., Reitsma J.B., Vandenbroucke J.P., Glas A.S., Bossuyt P.M.M. Case–control and two-gate designs in diagnostic accuracy studies. Clin Chem. 2005;51:1335 - 41.

9. Kam A.C., Li L.K., Yeung K.N., Wu W., Huang Z., Wu H., et al. Automated hearing screening for preschool children. Journal of Medical Screening. 2014;21:71-5.

10. Kam A.C.S., Gao H., Li L.K.C., Zhao H., Qiu S., Tong M.C.F. Automated hearing screening for children: A pilot study in China. International Journal of Audiology. 2013;52:855-60.

For peer review only

11. Mahomed-Asmail F., Swanepoel de W., Eikelboom R.H., Myburgh H.C., Hall J. Clinical validity of HearScreen smartphone hearing screening for school children. Ear and Hearing. 2016;37:e11-7.

12. McPherson B., Law M.M.S., Wong M.S.M. Hearing screening for school children: comparison of low-cost, computer-based and conventional audiometry. Child: Care, Health and Development. 2010;36:323-31.

13. Swanepoel D.W., Myburgh H.C., Howe D.M., Mahomed F., Eikelboom R.H. Smartphone hearing screening with integrated quality control and data management. International Journal of Audiology. 2014;53:841-9.

14. Wu H. A new hearing screening system for preschool children. International Journal of Pediatric Otorhinolaryngology. 2014;78:290-5.

15. Cloete T-L., Wilson W.J., Petersen L., Kathard H. Identifying a context-effective school hearing screening test: An emic/etic framework. International Journal of Audiology. 2015;54:605-12.

16. Georgalas C., Xenellis J., Davilis D., Tzangaroulakis A., Ferekidis E. Screening for hearing loss and middle-ear effusion in school-age children, using transient evoked otoacoustic emissions: a feasibility study. Journal of Laryngology and Otology. 2008;122:1299-304.

17. Lyons A., Kei J., Driscoll C. Distortion product otoacoustic emissions in children at school entry: a comparison with pure-tone screening and tympanometry results. Journal of the American Academy of Audiology. 2004;15:702-15.

18. Smiley D.F., Shapley K., Eckl D., Nicholson N. Comparison of pure-tone and distortion product otoacoustic emission screenings in school-age children. Journal of Educational Audiology. 2012;18:32-7.

19. Yin L., Bottrell C., Clarke N., Shacks J., Poulsen M.K. Otoacoustic emissions: a valid, efficient first-line hearing screen for preschool children. Journal of School Health. 2009;79:147-52.

For peer review only

For peer review only - http://bmjopen.bmj.com/site/about/guidelines.xhtml

20. Soares M., Nakazawa M., Ishikawa K., Sato T., Honda K. Hearing screening for Japanese children and young adults using the automated auditory brainstem response. Auris Nasus Larynx. 2014;41:17-21.

For peer review only

For peer review only - http://bmjopen.bmj.com/site/about/guidelines.xhtml

Footnotes

Acknowledgements

We would like to thank: the participating families and schools; the audiologists at each centre for sending out invitations for the study; Venessa Vas for helping to complete the screening; Elliot Carter, Brendan Lane and Paul Williams from PenCTU for developing the data entry websites; Brian Wainman and Mark Warner from PenCTU for entering data and assisting with the data management tasks; former project team member Vasilis Nikolaou; members of and observers on the study steering committee (John Bamford, John Fitzgerald, Vicki Kirwin, Kevin Munro, Kate Northstone, Karen Smith, Eldon Spackman, Maria Koufali, Stacey Arland, Angela Shone); Jon Deeks for critical comments on the conduct and reporting of the study; and Leala Watson for formatting images for the paper.

Author contributions

The protocol was developed and funding obtained by OCU, CH, RST, CB, JM and HF. HF was chief investigator with overall responsibility for the conduct of the study. OCU, CH, MO, ZZ, SE, RST, CB, JM, LC, JW and HF contributed to revisions of the design and conduct of the study. MO and SE managed the study and collected the diagnostic accuracy data. CB invited case children to the study. ZZ conducted an updated systematic review of the diagnostic accuracy of hearing screening tests. LC co-ordinated database design and development, data validation and data export. JW was the PPI representative on the study. OCU developed the statistical analysis plan which was critically revised by all authors. OCU undertook the analyses. OCU drafted the manuscript which was critically revised by all authors. OCU is the guarantor of the manuscript.

Declaration of competing interests

All authors have completed the Unified Competing Interest form at www.icmje.org/coi_disclosure.pdf (available on request from the corresponding author) and declare: no authors have support from any company for the submitted work; no authors have financial relationships with any organisations that might have an interest in the submitted work in the previous three years; no authors have any other relationships or activities that could appear to have influenced the submitted work.

For peer review only

Ethical Approval

Ethical approval was granted by the West Midlands, Staffordshire Research Ethics Committee (Ref: 106333). Written informed consent was obtained from the parent or legal guardian before the child entered the study.

Funding/support

The study was funded by the National Institute for Health Research Health Technology Assessment Programme (project no. 10/63/03). Obioha Ukoumunne, Zhivko Zhelev, Jaime Peters and Rod Taylor are supported by the National Institute for Health Research (NIHR) Collaboration for Leadership in Applied Health Research and Care South West Peninsula (PenCLAHRC). Chris Hyde is supported by the University of Exeter. Mara Ozolins and Sam Errington were supported by a grant from the NIHR Health HTA programme (10/63/03). Claire Benton is funded by Nottingham University Hospitals NHS Trust. Joanne Moody is funded by CCS NHS Trust and supported by Addenbrookes Hospital, Cambridge University Hospitals Foundation Trust. Heather Fortnum was funded by the University of Nottingham and supported by the NIHR NHBRU. The views and opinions expressed therein are those of the authors and do not necessarily reflect those of the HTA programme, the NHS, the NIHR or the Department of Health.

Role of the study sponsor and funder

Neither the funding body (National Institute for Health Research Health Technology Assessment Programme) nor the sponsor (University of Nottingham) had a role in the design of the study; collection, analysis and interpretation of the data; writing of the paper or the decision to submit it for publication.

Independence of researchers from funder

All researchers worked independently from the funder.

Data Access and responsibility

All authors had full access to all of the data (including statistical reports and tables) in the study and can take responsibility for the integrity of the data and the accuracy of the data analysis.

For peer review only

Transparency declaration

The lead author, Obioha Ukoumunne, affirms that the manuscript is an honest, accurate, and transparent account of the study being reported; that no important aspects of the study have been omitted; and that any discrepancies from the study as planned (and, if relevant, registered) have been explained.

Data sharing

Participant level data, the full dataset and statistical code are available from the corresponding author. Consent for this was not obtained but the presented data are anonymised and risk of identification is low.

Copyright

The Corresponding Author has the right to grant on behalf of all authors and does grant on behalf of all authors, a worldwide licence to the Publishers and its licensees in perpetuity, in all forms, formats and media (whether known now or created in the future), to i) publish, reproduce, distribute, display and store the Contribution, ii) translate the Contribution into other languages, create adaptations, reprints, include within collections and create summaries, extracts and/or, abstracts of the Contribution, iii) create any other derivative work(s) based on the Contribution, iv) to exploit all subsidiary rights in the Contribution, v) the inclusion of electronic links from the Contribution to third party material where-ever it may be located, and vi) licence any third party to do any or all of the above.

For peer review only

Table 1: Demographic characteristics of children by recruitment source

Characteristic	Recruited via audiology services (intended cases) (N = 75)	Recruited via schools (intended controls) (N = 240)
Male, n (%)	38 (51)	117 (49)
Age, mean (SD; range)	5.4 (0.9; 3.9 to 7.0)	5.4 (0.6; 4.0 to 6.9)
Ethnicity		
White, n (%)	61 (81)	189 (79)
Black, n (%)	2 (3)	14 (6)
Asian, n (%)	11 (15)	10 (4)
Mixed, n (%)	1 (1)	22 (9)
Other, n (%)	0 (0)	5 (2)

For peer review only

Table 2: Cross tabulation of Pure Tone Screen (PTS) versus HearCheck (HC) test results

		PTS test results			
		Refer	Pass	Missing	Total
		Impaired			
HC test results	Refer	136	2	1	139
	Pass	10	7	0	17
	Missing	0	0	0	0
	Total	146	9	1	156
		Hearing			
HC test results	Refer	34	26	0	60
	Pass	45	340	0	385
	Missing	0	1	0	1
	Total	79	367	0	446
		Missing			
HC test results	Refer	13	2	1	16
	Pass	3	2	1	6
	Missing	2	0	4	6
	Total	18	4	6	28

For peer review only - http://bmjopen.bmj.com/site/about/guidelines.xhtml

Table 3: Accuracy of Pure Tone Screen (PTS) and HearCheck (HC)

Measure	Pure Tone Screen estimate (95% CI)	HearCheck estimate (95% CI)	Difference in accuracy (PTS – HC) estimate (95% CI)	p value
**Sensitivity**	94.2% (89.0% to 97.0%)	89.0% (82.9% to 93.1%)	5.2% (0.2% to 10.1%)	0.02
**Specificity**	82.2% (77.7% to 86.0%)	86.5% (82.5% to 90.0%)	-4.3% (-8.2% to -0.4%)	0.02

For peer review only

For peer review only - http://bmjopen.bmj.com/site/about/guidelines.xhtml

Table 4: Frequency of test results per 10,000 screened in a hypothetical population

Test results	Test		Difference (PTS - HC)
	Pure Tone Screen (PTS)	HearCheck (HC)	
True positives	47	45	2
True negatives	8179	8607	
False positives	1771	1343	428
False negatives	3	5	

For peer review only

For peer review only - http://bmjopen.bmj.com/site/about/guidelines.xhtml

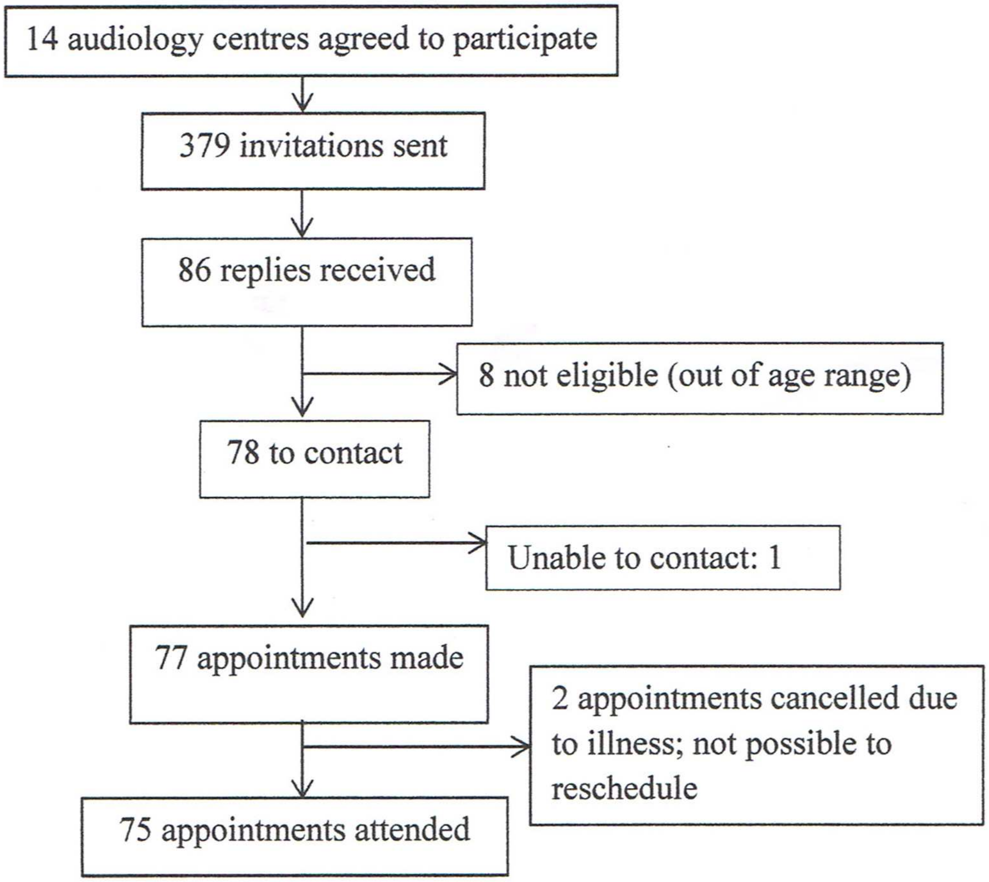

Recruitment of intended case children

123x110mm (300 x 300 DPI)

only

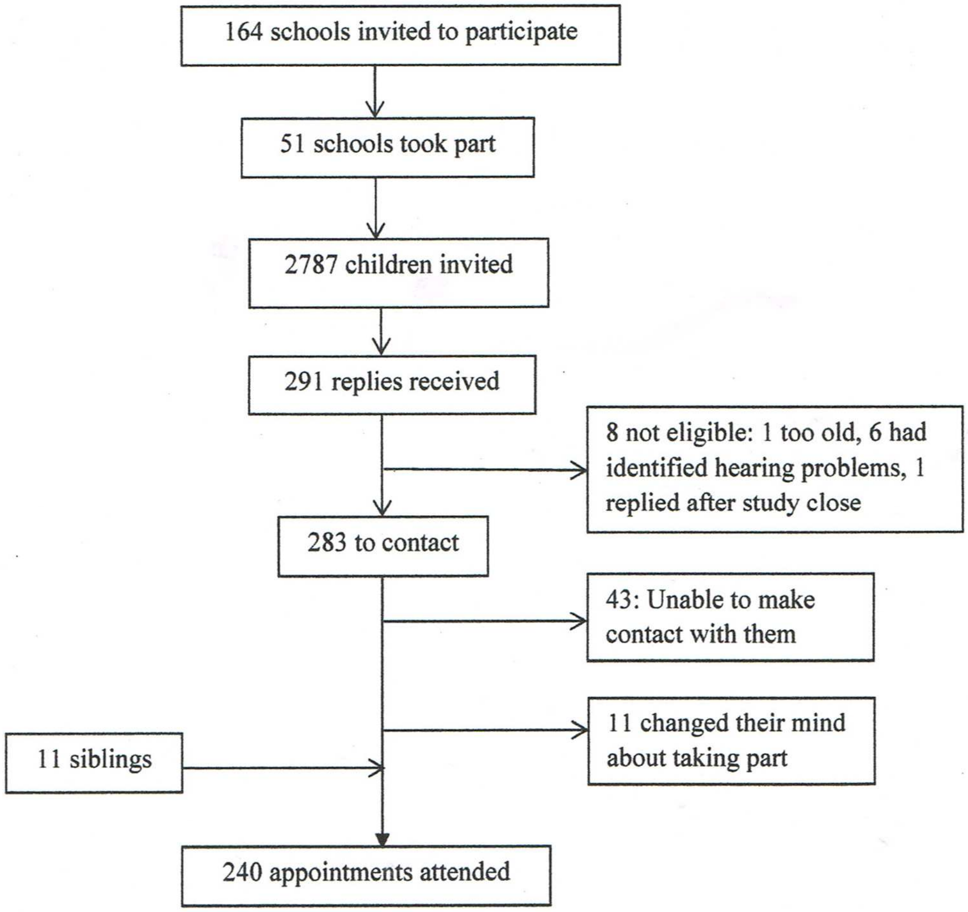

Recruitment of intended control children

145x137mm (300 x 300 DPI)

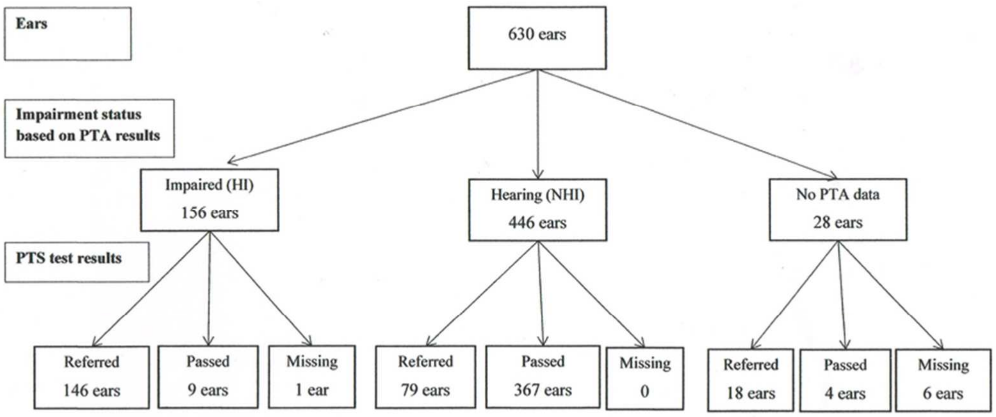

Pure Tone Screen (PTS) test results at ear level by hearing impairment status (PTA)

72x30mm (300 x 300 DPI)

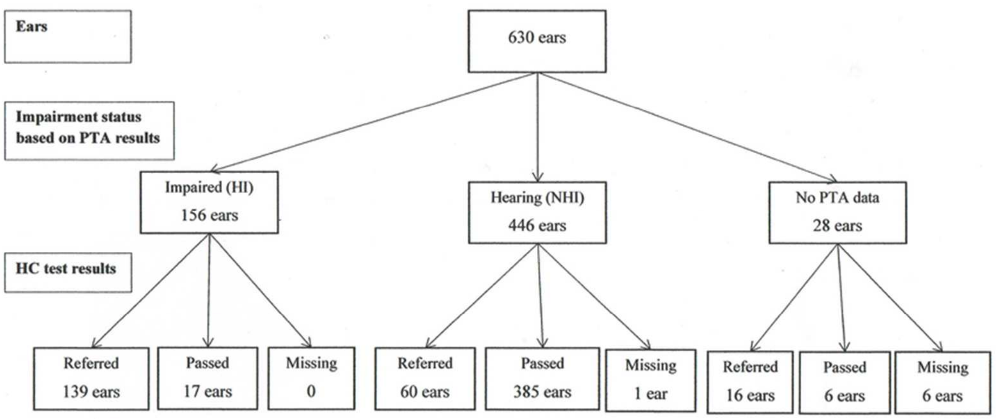

HearCheck (HC) test results at ear level by hearing impairment status (PTA)

72x29mm (300 x 300 DPI)

Appendix

Variability in diagnostic accuracy across different scenarios

The primary analyses reported in the main text are for accuracy at ear level of the pure tone screen (PTS) and HearCheck Screener (HC) tests for distinguishing hearing impaired ears from non-impaired ears. Hearing impairment was defined as present when the PTA reference standard threshold was ≥30dB on at least one of the four frequencies (0.5 kHz, 1 kHz, 2 kHz and 4 kHz) and absent when the reference threshold was <30dB on all four frequencies. All impaired ears were used to calculate test sensitivity regardless of whether belonging to children recruited via audiology services (intended cases) or via schools (intending controls). In addition, we performed further exploratory analyses estimating accuracy of the tests in alternative scenarios:

a) when impairment was defined as present when the mean PTA threshold across the four frequencies was ≥30dB and absent when the mean threshold was <30dB,
b) when only impaired ears (children) that were recruited via audiology services were used to estimate sensitivity and when only impaired ears (children) that were recruited via schools were used to estimate sensitivity,
c) at child level for distinguishing between hearing impaired children and non-impaired children.

The primary definition of hearing impairment is stricter (i.e., the PTA reference standard is harder to pass) than the one based on mean hearing level across the 4 frequencies, since under the former the ear needs to pass on all 4 frequencies to pass overall.

The estimate of sensitivity based on only impaired ears belonging to children recruited via audiology services was carried out to quantify the ability of the tests to identify established hearing impairment. The estimate based on only impaired ears belonging to those recruited via schools was carried out to quantify the ability of the tests to identify impairment that has not previously been established.

The ear-level analyses were presented as primary as this reflects the intrinsic accuracy of the tests. Child-level analyses, however, have practical relevance because, regardless of whether just one ear or both ears are impaired, the child will be referred for diagnostic testing. To be included in the child-level analyses a child needed to provide full data on the tests and reference standard for both ears.

For peer review only - http://bmjopen.bmj.com/site/about/guidelines.xhtml

Table A1 reports the sensitivity and specificity of PTS and HC for each combination of: ear-level versus child-level analyses; definition of impairment (PTA score ≥30dB on at least one frequency versus mean PTA score ≥30dB across all four frequencies); and subset of impaired children used to calculate sensitivity (all children versus only children recruited from audiology services versus only children recruited from schools).

At ear level the sensitivity is generally higher (especially for the HC) when impairment is defined based on average hearing level across the four frequencies. This might be expected as this definition is easier to pass than our primary definition of impairment, thus resulting in only the more severely impaired ears being included in the impaired group and higher sensitivity. For the same reason the specificity is lower for both tests when impairment status is based on average hearing level across the frequencies presented under the PTA.

Restricting impaired ears in the analysis to only those belonging to children recruited via audiology services (intended cases) increased sensitivity relative to inclusion of all impaired ears. Again, this would be expected as ears of such children would be expected to have more severe hearing loss. Restricting impaired ears in the analysis to only those belonging to children recruited via schools (intended controls) results in lower sensitivity. This latter result is notable because the impaired ears of children with no previously identified hearing impairment are likely to be more representative of the spectrum of impairment in the type of child that we would predominantly want to identify in a school-based setting.

The child-level analyses generally provided a similar pattern of results to the ear-level analyses, except that the PTS test was markedly more sensitive in the child-level analyses when including only impaired children recruited via schools.

For peer review only

Table A1: Accuracy of Pure Tone Screen and HearCheck at ear level and child level across different definitions of impairment status and different subsets of impaired children based on whether recruited via audiology services (intended cases) or schools (intended controls)

Reference standard	Subset of impaired children	Pure Tone Screen		HearCheck	
		Sensitivity	Specificity	Sensitivity	Specificity
Ear-level analysis					
PTA score ≥30dB on at least one frequency	All children (primary analysis)	94.2%	82.2%	89.0%	86.5%
	Intended cases only	99.1%		97.2%	
	Intended controls only	83.3%		70.8%	
Average PTA score ≥30dB across frequencies	All children	95.7%	76.4%	94.8%	81.8%
	Intended cases only	98.9%		97.8%	
	Intended controls only	84.0%		84.0%	
Child-level analysis					
PTA score ≥30dB on at least one frequency	All children	95.9%	79.8%	88.7%	83.8%
	Intended cases only	98.3%		98.3%	
	Intended controls only	91.9%		73.0%	
Average PTA score ≥30dB across frequencies	All children	97.3%	72.7%	93.3%	78.2%
	Intended cases only	98.1%		98.1%	
	Intended controls only	95.2%		81.0%	

For peer review only

Section & Topic	No	Item	Reported on page #
TITLE OR ABSTRACT			
	1	Identification as a study of diagnostic accuracy using at least one measure of accuracy (such as sensitivity, specificity, predictive values, or AUC)	1, 3
ABSTRACT			
	2	Structured summary of study design, methods, results, and conclusions (for specific guidance, see STARD for Abstracts)	3
INTRODUCTION			
	3	Scientific and clinical background, including the intended use and clinical role of the index test	5
	4	Study objectives and hypotheses	5
METHODS			
Study design	5	Whether data collection was planned before the index test and reference standard were performed (prospective study) or after (retrospective study)	6
Participants	6	Eligibility criteria	6
	7	On what basis potentially eligible participants were identified (such as symptoms, results from previous tests, inclusion in registry)	6
	8	Where and when potentially eligible participants were identified (setting, location and dates)	6
	9	Whether participants formed a consecutive, random or convenience series	6
Test methods	10a	Index test, in sufficient detail to allow replication	7
	10b	Reference standard, in sufficient detail to allow replication	7, 8
	11	Rationale for choosing the reference standard (if alternatives exist)	no alternatives
	12a	Definition of and rationale for test positivity cut-offs or result categories of the index test, distinguishing pre-specified from exploratory	7
	12b	Definition of and rationale for test positivity cut-offs or result categories of the reference standard, distinguishing pre-specified from exploratory	7, 8
	13a	Whether clinical information and reference standard results were available to the performers/readers of the index test	8
	13b	Whether clinical information and index test results were available to the assessors of the reference standard	8
Analysis	14	Methods for estimating or comparing measures of diagnostic accuracy	8
	15	How indeterminate index test or reference standard results were handled	10
	16	How missing data on the index test and reference standard were handled	10
	17	Any analyses of variability in diagnostic accuracy, distinguishing pre-specified from exploratory	29, 30, 31
	18	Intended sample size and how it was determined	8
RESULTS			
Participants	19	Flow of participants, using a diagram	24, 25
	20	Baseline demographic and clinical characteristics of participants	10, 20
	21a	Distribution of severity of disease in those with the target condition	10
	21b	Distribution of alternative diagnoses in those without the target condition	10
	22	Time interval and any clinical interventions between index test and reference standard	10
Test results	23	Cross tabulation of the index test results (or their distribution) by the results of the reference standard	21, 26, 27
	24	Estimates of diagnostic accuracy and their precision (such as 95% confidence intervals)	10, 11, 22
	25	Any adverse events from performing the index test or the reference standard	10
DISCUSSION			
	26	Study limitations, including sources of potential bias, statistical uncertainty, and generalisability	11, 12, 13
	27	Implications for practice, including the intended use and clinical role of the index test	13
OTHER INFORMATION			
	28	Registration number and name of registry	3
	29	Where the full study protocol can be accessed	6
	30	Sources of funding and other support; role of funders	18

For peer review only

STARD 2015

AIM

STARD stands for "Standards for Reporting Diagnostic accuracy studies". This list of items was developed to contribute to the completeness and transparency of reporting of diagnostic accuracy studies. Authors can use the list to write informative study reports. Editors and peer-reviewers can use it to evaluate whether the information has been included in manuscripts submitted for publication.

EXPLANATION

A **diagnostic accuracy study** evaluates the ability of one or more medical tests to correctly classify study participants as having a **target condition.** This can be a disease, a disease stage, response or benefit from therapy, or an event or condition in the future. A medical test can be an imaging procedure, a laboratory test, elements from history and physical examination, a combination of these, or any other method for collecting information about the current health status of a patient.

The test whose accuracy is evaluated is called **index test.** A study can evaluate the accuracy of one or more index tests. Evaluating the ability of a medical test to correctly classify patients is typically done by comparing the distribution of the index test results with those of the **reference standard**. The reference standard is the best available method for establishing the presence or absence of the target condition. An accuracy study can rely on one or more reference standards.

If test results are categorized as either positive or negative, the cross tabulation of the index test results against those of the reference standard can be used to estimate the **sensitivity** of the index test (the proportion of participants *with* the target condition who have a positive index test), and its **specificity** (the proportion *without* the target condition who have a negative index test). From this cross tabulation (sometimes referred to as the contingency or "2x2" table), several other accuracy statistics can be estimated, such as the positive and negative **predictive values** of the test. Confidence intervals around estimates of accuracy can then be calculated to quantify the statistical **precision** of the measurements.

If the index test results can take more than two values, categorization of test results as positive or negative requires a **test positivity cut-off**. When multiple such cut-offs can be defined, authors can report a receiver operating characteristic (ROC) curve which graphically represents the combination of sensitivity and specificity for each possible test positivity cut-off. The **area under the ROC curve** informs in a single numerical value about the overall diagnostic accuracy of the index test.

The **intended use** of a medical test can be diagnosis, screening, staging, monitoring, surveillance, prediction or prognosis. The **clinical role** of a test explains its position relative to existing tests in the clinical pathway. A replacement test, for example, replaces an existing test. A triage test is used before an existing test; an add-on test is used after an existing test.

Besides diagnostic accuracy, several other outcomes and statistics may be relevant in the evaluation of medical tests. Medical tests can also be used to classify patients for purposes other than diagnosis, such as staging or prognosis. The STARD list was not explicitly developed for these other outcomes, statistics, and study types, although most STARD items would still apply.

DEVELOPMENT

This STARD list was released in 2015. The 30 items were identified by an international expert group of methodologists, researchers, and editors. The guiding principle in the development of STARD was to select items that, when reported, would help readers to judge the potential for bias in the study, to appraise the applicability of the study findings and the validity of conclusions and recommendations. The list represents an update of the first version, which was published in 2003.

More information can be found on http://www.equator-network.org/reporting-guidelines/stard.

For peer review only
